# Identification of Potential Novel Interacting Partners for Coagulation Factor XIII B (FXIII-B) Subunit, a Protein Associated with a Rare Bleeding Disorder

**DOI:** 10.3390/ijms20112682

**Published:** 2019-05-31

**Authors:** Sneha Singh, Mohammad Suhail Akhter, Johannes Dodt, Peter Volkers, Andreas Reuter, Christoph Reinhart, Christoph Krettler, Johannes Oldenburg, Arijit Biswas

**Affiliations:** 1 Institute of Experimental Hematology and Transfusion Medicine, University Clinic Bonn, 53127 Bonn, Germany; Sneha.Gupta@ukbonn.de (S.S.); suhailaiims@gmail.com (M.S.A.); johannes.oldenburg@ukbonn.de (J.O.); 2 College of Applied Medical Sciences, Jazan University, 82911 Jazan, Saudi Arabia; 3 Paul-Ehrich Institute, 63225 Langen, Germany; Johannes.Dodt@pei.de (J.D.); Peter.Volkers@pei.de (P.V.); Andreas.Reuter@pei.de (A.R.); 4 Department of Molecular Membrane Biology, Max-Planck Institute for Biophysics, Max-Von Laue Str 3, 60438 Frankfurt am Main, Germany; christoph.reinhart@biophys.mpg.de (C.R.); christoph.krettler@biophys.mpg.de (C.K.)

**Keywords:** Factor XIII, FXIII deficiency, excipients, pleiotropy, mass spectrometry, complement system

## Abstract

Coagulation factor XIII (FXIII) is a plasma-circulating heterotetrameric pro-transglutaminase complex that is composed of two catalytic FXIII-A and two protective/regulatory FXIII-B subunits. FXIII acts by forming covalent cross-links within a preformed fibrin clots to prevent its premature fibrinolysis. The FXIII-A subunit is known to have pleiotropic roles outside coagulation, but the FXIII-B subunit is a relatively unexplored entity, both structurally as well as functionally. Its discovered roles so far are limited to that of the carrier/regulatory protein of its partner FXIII-A subunit. In the present study, we have explored the co-presence of protein excipients in commercial FXIII plasma concentrate FibrogamminP by combination of protein purification and mass spectrometry-based verification. Complement factor H was one of the co-excipients observed in this analysis. This was followed by performing pull down assays from plasma in order to detect the putative novel interacting partners for the FXIII-B subunit. Complement system proteins, like complement C3 and complement C1q, were amongst the proteins that were pulled down. The only protein that was observed in both experimental set ups was alpha-2-macroglobulin, which might therefore be a putative interacting partner of the FXIII/FXIII-B subunit. Future functional investigations will be needed to understand the physiological significance of this association.

## 1. Introduction

Coagulation Factor XIII (FXIII) is plasma-circulating pro-transglutaminase acting at the terminal phase of the coagulation pathway, which is responsible for cross-linking pre-formed fibrin polymers within it and to anti-fibrinolytic inhibitors to prevent its premature fibrinolysis. In plasma, it circulates as a zymogenic heterotetramer composed of dimeric subunits of catalytic FXIII-A and carrier/regulatory FXIII-B bound to each other non-covalently [1]. The inherited form of FXIII deficiency is a rare autosomal disorder with a prevalence of one in three million, with its clinical manifestations ranging from mild to severe bleeding diathesis [2]. The FXIII deficient patients are presented with severe bleeding tendencies, such as intracranial bleeds, dysmenorrhea, and umbilical cord bleeding, etc. [2]. The catalytic component of FXIII-A_2_B_2_ complex, i.e., FXIII-A subunit, is a structurally and functionally well characterized protein [3,4]. The FXIII-B subunit, which is the regulatory/protective partner, in comparison is a relatively unexplored entity. Homology studies reveal that FXIII-B bears 10 sushi domains (or Complement Control Protein modules), although no biophysical structures for this subunit exist so far [5,6]. The possibility that it might have pleotropic roles outside the coagulation pathway exists similar to its partner FXIII-A subunit, which is known to be involved in roles beyond coagulation, like inflammation, angiogenesis, and wound healing, since FXIII-B is present both in complexed (FXIII-A_2_B_2_) and free form [7]. However, only a select few studies on the FXIII-B subunit have investigated this possibility and have reported mostly negative results [8]. Patients that are severely deficient for FXIII often require a lifelong supplement of plasma derived FXIII concentrates as a majorly available treatment modality in cases of inherited FXIII deficiencies [9]. However, in the case of acquired FXIII deficiency, which may be a secondary effect of immune-mediated inhibition, or defective synthesis and/or consumption of either of FXIII subunits; the treatment involves antifibrinolytic administration, and/or inhibitor eradication, along with replacement therapy [10]. Hence, replenishing the FXIII deficient and/or defective plasma by active and functional FXIII is a leading treatment modality. The two major types of FXIII concentrates that are administered to the patients include virus inactivated fresh frozen plasma (FFP) derived from healthy donors; or the commercially available drug Cortifact (US)/FibrogamminP (Europe & Asia), marketed by CSL Behring [9,11]. These plasma concentrates are suitable for both FXIII-A and FXIII-B subunit deficient states. Recently, a recombinant form of FXIII (Tretten) expressed in yeast has also been commercialized by NovoNordisk and it is only being administered to patients with severe/mild FXIII-A deficiency [12]. The plasma concentrate FibrogamminP is a highly purified, pasteurized, plasma-derived concentrate that has been in use since 1993 and it contains the hetero-tetrameric complex that shows high transglutaminase activity [12]. The other main excipients that are currently indicated in the commercial product are human albumin, glucose, and sodium chloride. In the current study, we performed content characterization of plasma derived FibrogamminP, by gel filtration analyses. Amongst others, we detected complement factor H (CFH) as one of the major proteomic excipients within FibrogamminP. Owing to the structural and sequential complementarity of CFH and FXIII-B [8], we further evaluated whether this co-presence has any functional/physiological implications or not, which is verified by real-time FXIIIa generation assay [13]. However, CFH was not detected in the pull-down assays under the physiological conditions. Additionally, in vitro qualitative assessment of proteins interacting with FXIII-B subunit employing FXIII-B subunits interacting with FXIII-B monoclonal antibodies immobilized to resin, which aided the pull-down of interacting partners in a FXIII deficient background (FXIII-DP) was done. Two complement system proteins i.e., complement C3 and complements C1q were detected in the pull-down assays. When compared to all the detected proteins, only alpha-2-macroglobulin was a common denominator detected in the pull-down assay as well as an excipient in FibrogamminP, which indicates that it might be the true interacting partner of the FXIII/FXIII-B subunit.

## 2. Results

### 2.1. Content Characterization of Plasma FXIII Concentrate Reveals Co-Presence of Complement Factor H and Alpha-2-Macroglobulin Along with FXIII Complex

**Figure 1:**
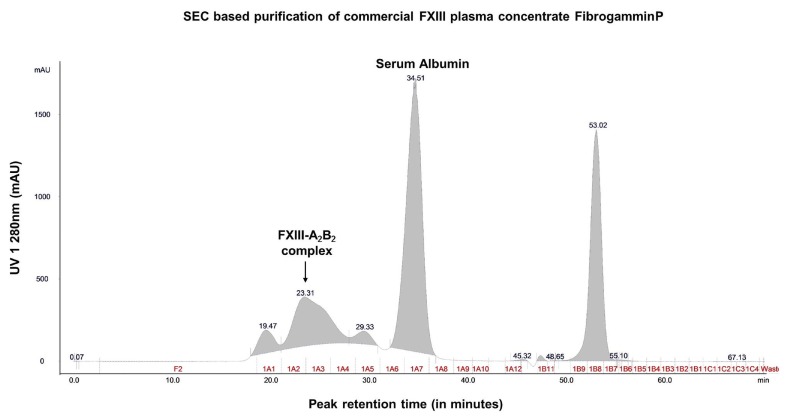
Content characterization of plasma FXIII concentrate (FibrogamminP) by size exclusion chromatography. This image represents the mass-based separation run for commercial plasma FXIII concentrate FibrogamminP on a ÄKTA explorer purifier system. The respective peaks on the chromatograph were detected by UV_280. The arrow indicates the main peak corresponding to the molecular weight of FXIII-A_2_B_2_ complex that was further characterized.

**Figure 2:**
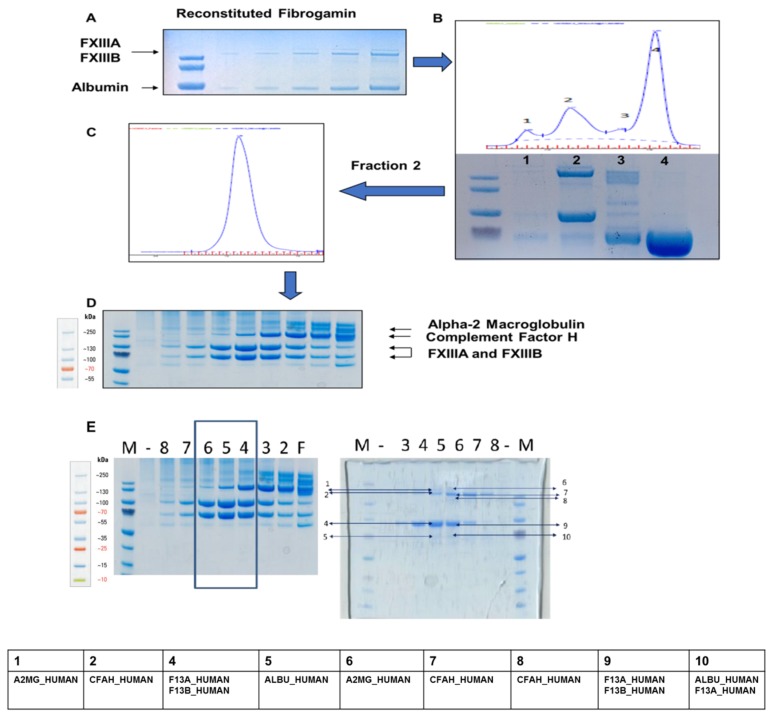
Plasma FXIII concentrate (FibrogamminP) analysis. (**A**) The preliminary SDS-PAGE gel run of the commercial FXIII plasma concentrate “FibrogamminP” reconstituted in water. The fastest running band corresponds to human serum albumin, which is a known constituent of FibrogamminP. (**B**) Mass based separation of reconstituted FibrogamminP run on a SEC column (Superdex 200 PC 3.2/30 column, running buffer: 20 mM Tris HCl, pH 7.4; 120 mM NaCl). The four main peaks of interests were collected separately. Peak 4 corresponds to albumin (size-wise). Peak 2 contained majority of FXIII-A_2_B_2_ heterotetramer complex. The sampled Peak 2 was separately run again, but it still resolved into one peak only shown in (**C**). This single, monodispersed peak was fractioned, sampled and run on an SDS-PAGE, fraction wise as collected from the SEC; which is shown in (**D**) The separated bands (see Figure S3) were excised and evaluated with Mass spectrometry. (**E**) This panel consists of two SDS-PAGE gels, the gel 1 is same as the one in (**D**) depicting fractions corresponding to FXIII complex (boxed lanes). These three fractions were re-pooled and ran on GFC, with fractions separated on gel 2. Marked bands were analysed by mass spectrometry. The labels at the top of both gels are numbered based on fraction ids from the gel filtration runs not to be confused with lane numbers. The “F” represents the crude fraction of FibrogamminP. Table at the bottom represents the major protein hits corresponding to each band (total nine). For detailed peptide summary report please refer Supplementary Materials (Figure S3).

Size exclusion chromatography revealed that the crude fractions of FibrogamminP contain majorly coagulation FXIII-A_2_B_2_ (MW 320 kDa) (retention at 23.31 min), along with albumin (MW 66 kDa) (at 34.51 min), alpha-2-macroglobulin (MW 725 kDa) (at 19.47 min), and complement Factor H (MW 155 kDa) (at 29.33 min) eluted at different retention times (Figure 1, Figure 2 and Figure S1). The peak corresponding to the molecular weight of FXIII-A_2_B_2_ was collected and re-run to generate a single monodispersed homogenous peak. Upon analysis, this peak showed the co-presence of complement Factor H and albumin, in addition to FXIII-A_2_B_2_ (Figure 1, Figure 2, Figures S1 and S3, and attached MS data files (peptide summary reports) in the Supplementary Information).

### 2.2. No Significant Effect of CFH on FXIII-Aa Activation Observed in the FXIIIa Generation Assay

**Figure 3:**
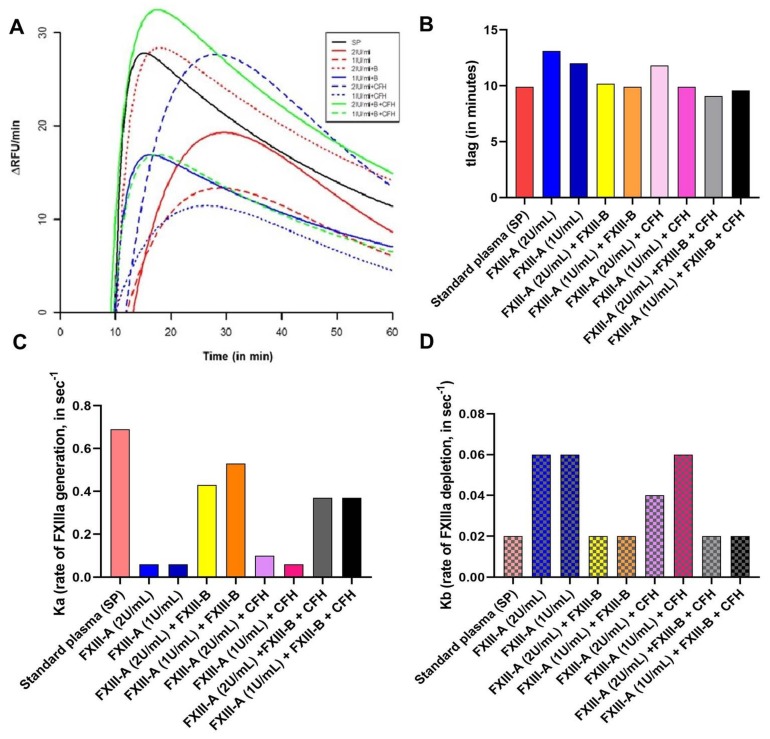
Effect of Complement Factor H (CFH) on FXIIIa generation. FXIIIa generation assay is a real-time, quantitative measurement of generation of active FXIIIa species, in a FXIII deficient background (deficient both for FXIII-A and FXIII-B subunits) [13]. The parameters tlag (time-delay in generation of first signal), Ka (constant of absorption that describes the rate of development of FXIIIa), and Kb (elimination constant) have been represented here. (**A**) Raw data obtained as growth curves representing the generation of active FXIIIa species. X-axis denotes time in minutes; Y-axis denotes the rate of generation of active FXIII-Aa (RFU/min). (**B**–**D**) are comparative bar graph representation of tlag, Ka and Kb observed with different spiking conditions. A tabular representation can be found in Supplementary Information.

The FXIIIa generation assay reveals that the rate of activation of FXIII-A is accelerated in the presence of FXIII-B (Ka (FXIII-DP+ FXIII-A) is 0.12 sec^−1^; Ka (FXIII-DP+ FXIII-A+FXIII-B) is 0.54 sec^−1^) (Figure 3 and Supplementary Table S1). However, the rate of depletion of activated species is reduced in the presence of FXIII-B subunit and a similar effect is observed with CFH. The lag time, which represents thrombin accessibility to FXIII-A molecule, is also mildly influenced (but not significantly so) by both CFH and FXIII-B. However, most of the parameters that were analyzed for these set of experiments showed non-significant association (Figure 3; and Supplementary Table S1).

### 2.3. In a FXIII Deficient Background, FXIII-B Pulls Down Fibrinogen, Few Complement Proteins, and Alpha-2-Macroglobulin

**Figure 4:**
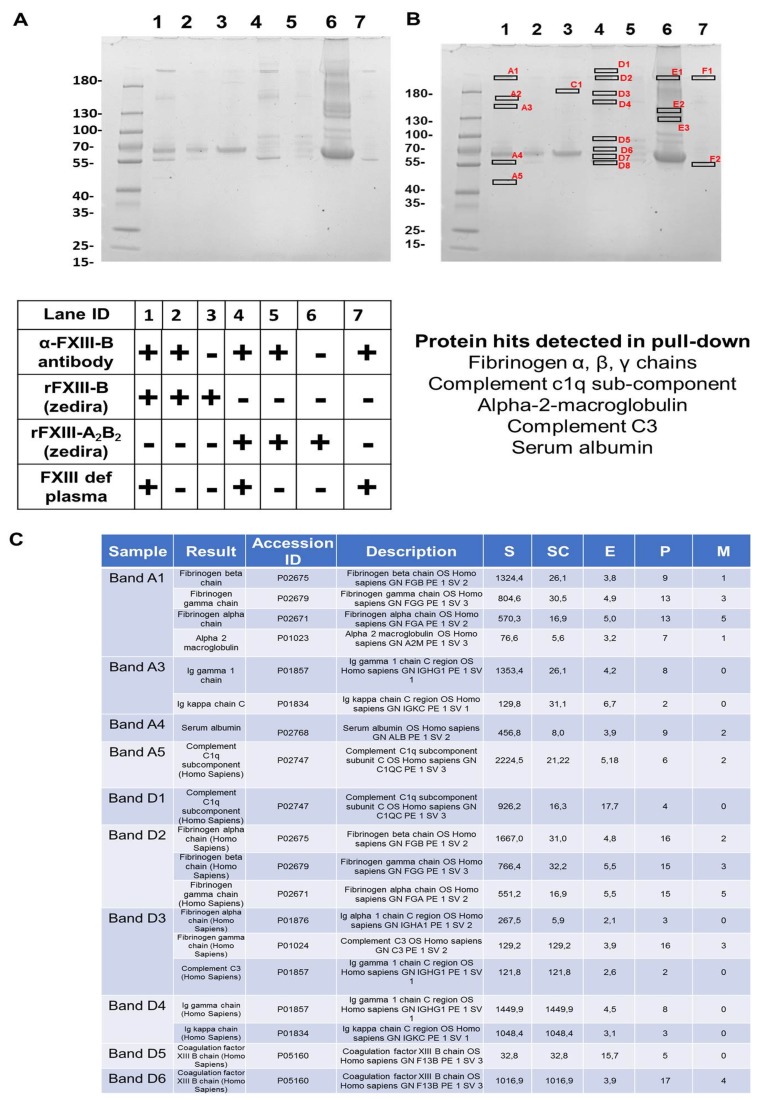
Interactome analysis of FXIII-B in FXIII-DP by Co-immunoprecipitation. (**A**) Coomassie stained SDS-PAGE gel for the proteins pulled down by resin immobilized FXIII-B (bound through amine-linked monoclonal antibody). Table below shows the experimental conditions. (**B**) Shows the same SDS-PAGE as in Panel A, but with those bands that were subsequently analysed for proteomic content by mass spectrometry individually marked (data in Supplementary Figure S2). The major hits are listed at the bottom of this panel, as well as in (**C**). (**C**) Table representing the majority of protein hits obtained by MS analyses of excised bands indicated in B. For a detailed protein-hit summary refer Supplementary Information. Abbrev: S: Protein Score; SC: Sequence coverage in %, E: Mean mass error in ppm; P: number of identified peptides; M; number of modified peptides interactome analyses.

The pull down from rFXIII-B bound resin that had been exposed to FXIII-DP had alpha-2-macroglobulin, complement C3, complement C1q, and fibrinogen-α, -β, -γ chains (Figure 4C lists the most relevant proteins; for complete list see Figure S2) in its proteomic content. Resin bound rFXIII-B exposed to rFXIII-A and FXIII-DP also showed similar content in its pull down. No CFH or alpha-2-macroglobulin that was detected earlier in FibrogamminP was detected in any of these pull downs (Figure 4 and Figure S2).

## 3. Discussion

Amongst the two FXIII subunits, the FXIII-B_2_ dimeric subunit has a unique filamentous structure, with each of its monomers consisting of 10 sushi domains (also known as complement control protein (CCP) modules) that are rich in cysteine bonds and bear specific structure with beta-sandwich arrangement [6,14,15,16]. Other than FXIII-B, a majority of proteins belonging to the complement pathway have such structural domains [17]. The structural arrangement of sushi domains in FXIII-B bear close resemblance to CFH, which might be the result of the co-evolution of complement and coagulation systems. Based on the sequence and structural homology, our group had earlier reported the homology models for the FXIII-B sushi domains [6,15]. In the current report, we find detectable levels of CFH in plasma-derived factor concentrate of FXIII. The peak corresponding to FXIII-A_2_B_2_ appears to be homogenous and monodispersed in gel filtration analyses (obtained after re-analyzing the peak corresponding to FXIII, there appears to be a non-stoichiometric association of proteins within this eluted peak, later detected by MS (Supplementary Information)). Such an association might also be suggestive of its association to albumin instead of the main active drug FXIII-A_2_B_2_. The separated bands of individual fraction, detected by coomassie staining, when evaluated by MS were determined to be FXIII-A_2_ and FXIII-B_2_ subunits, CFH, and alpha-2-macroglobulin in the order, as indicated in Figure 2D. However, the functional analysis reveals that the presence of CFH does not alter FXIII activity, as observed from the FXIIIa generation assay in which no significant change in variables was observed when CFH was added along with FXIII (Figure 3). Their co-presence in FibrogamminP may be attributed to the very nature of sushi domains and/or the similar size of FXIII heterotetramer (320 kDa) and dimeric CFH (155 kDa monomer), as well as a possible association with albumin. Since only 4–15% of total CFH tends to self-assemble in plasma, forming CFH dimers of ~320 kDa, this may explain the reduced amount of CFH detected in FXIII concentrate. Along with CFH, traces of alpha-2-macroglubin (homo-tetrameric acute phase protein, MW 163 kDa) were also detected by size-exclusion chromatography, followed by mass spectrometry in FibrogamminP. Since dimeric forms of alpha-2-macroglubin have also been recently described and these would have a molecular weight that is close to the FXIII heterotetramer, which might also explain their co-presence with FXIII [18,19]. The fact that CFH is merely co-present with FXIII is substantiated by interactome-analysis of FXIII in FXIII-DP (Figure 4). While we found that fibrinogen-α, -β, -γ chains, complement C1q, and complement C3 in the pull downs for both FXIII-B, as well as FXIII-B exposed to FXIII-A, none of the pull downs detected CFH. However, alpha-2-macroglobulin was one protein that was detected in FibrogamminP as well as in the pull-down assays. This suggests that it could be one of the novel proteins interacting with FXIII-B subunit. It is already known that alpha-2-macroglobulin is a substrate for the FXIII-A subunit (although no clear functional role has been discovered in this context), but there are no reports of its direct interaction with the FXIII-B subunit [20]. Alpha-2-macroglobulin is an inhibitor that can inhibit coagulation as well as fibrinolysis by acting on thrombin and plasmin, respectively [18,19]. Interaction with FXIII or FXIII-A/FXIII-B subunit might fine tune its inhibitory roles towards the two different processes. Further functional analysis will be needed to substantiate this idea. The presence of fibrinogen chains (from deficient plasma) in the pull downs of resin bound FXIII-B supports earlier reports that showed that the FXIII-B subunit could be mediating the interaction between FXIII and fibrinogen [21,22]. The presence of complement C1q and complement C3 in the pull-down assays came as a surprise. There has been no report of FXIII or the FXIII-B subunit interacting with complement C1q so far. The complement C3 protein, on the other hand, has been shown to be a substrate for FXIII-A subunit, but again, no interaction with FXIII-B subunit has been reported so far [18]. The interaction possibility of alpha-2-macroglobulin complement C1q and complement C3 with FXIII/FXIII-B subunit, as shown from our study, presents the opportunity to discover newer roles for FXIII/FXIII-B subunit in coagulation, as well as outside the coagulation pathway. These proteins (i.e., alpha-2-macroglobulin, complement C1q and CFH) are related to known physiological and diseased states. Complement C1q deficiency has been known to cause recurrent skin lesions, chronic infections, systemic lupus erythematosus (SLE), and has also been associated with a kidney disease, known as mesangial proliferative glomerulonephritis [23,24]. Complement C3 deficiency manifests itself into recurrent bacterial infections [25]. Elevated plasma levels of alpha-2-macroglobulin, along with Fibrinogen and albumin levels, is commonly seen in nephrotic syndrome [26,27,28]. Therefore, FXIII could play an important role in all these aspects by interacting with these proteins. A significant association of FXIII with a complement system had been discussed by Schroeder et al.; describing FXIII mediated covalent cross-linking of fibrin to complement C3 that could have inflammatory roles in pro-thrombotic states [7,29]. Our study demonstrates that there are a number of proteins, some of which are part of the complement system (like complement C3 and complement C1q), and some of which belong to the coagulation pathway itself (alpha-2-macroglobulin) that might interact with the FXIII/FXIII-B subunits. More work will be needed to further investigate whether these interactions have physiological or pathological implications. The complement factor H protein that shares homology with FXIII-B subunit, on the other hand, is merely co-present with FXIII in FibrogamminP and it appears to have no functional interaction with FXIII/FXIII-B subunit.

## 4. Materials and Methods

### 4.1. Coagulation Factor XIII Complex and FXIII Subunits

Three separate lots of FibrogamminP (CSL Behring; Marburg, Germany) were used as source, to purify FXIII-A_2_B_2_ complex. Recombinant FXIII-A and recombinant FXIII-B were purchased from Zedira GmBH (Darmstadt, Germany). Additionally, the FXIII-B subunit was also expressed and purified in house, as per previously reported protocol [30]. Briefly, the human *HEK293t* cell line purchased from DMSZ (German Collection of Microorganisms and Cell Cultures, Braunschweig, Germany) were cultured in high glucose DMEM, supplemented with 10% *v*/*v* FBS, 1% *v*/*v* Penicillin-Streptomycin antibiotics and 0.1% *v*/*v* Fungisone (all cell culture products bought from Invitrogen, Bleiswijk, Netherlands), at 37 °C at 5% CO_2_. All of the experiments were done on sub-cultured cells in logarithmic phase (below passage 20). Human FXIII-B cDNA, inserted into the cloning site of pReciever-M01 mammalian expression vector was transfected into *HEK293t* cells, as per previously reported protocol [15]. Briefly, 2.7 × 10^5^ cells were transfected with 3 µg of plasmid DNA along with 6 µl of transfection reagent Lipofectamine 2000 (Invitrogen, Bleiswijk, Netherlands). The cultures were harvested 48 h post-transfection, by collecting extracellular fractions containing the secreted rFXIII-B. The extracellular fraction was centrifuged for five minutes, at 14,000 rpm at 4 °C. A negative control of non-transfected cells was used, whereas a plasmid construct with eGFP cloned in pcDNA mammalian expression vector was the positive control for transfection. Secreted protein harvested post transfection of *HEK293t* cells was concentrated and subjected to immuno-affinity-based purification while using the Thermo Scientific Pierce Co-IP kit (Pierce Biotechnology, Rockford, IL, USA), following the manufacturer’s protocol. Briefly, monoclonal antibodies against FXIII-B, raised in mice were immobilized to Amino Link plus coupling resin (Pierce Biotechnology, Rockford, IL, USA) for two hours. The resin was then washed with PBS and incubated with extracellular concentrate overnight in cold-room. The next day, the resin was again washed with PBS and protein bound to anti-FXIII-B antibodies was eluted with acidic elution buffer provided with the kit. The eluted protein was verified on coomassie stained gel. Eluted protein was further subjected to gel filtration chromatography, to ensure the purity and dimeric state of the recombinant protein.

### 4.2. Separation of the FXIII Plasma Concentrate, FibrogamminP into its Constituents

One vial (from three different lots) of FibrogamminP (CSL Behring; Marburg, Germany) i.e., 250 U, was reconstituted with water, as per the manufacturer’s guidelines. The sample was purified in ÄKTA explorer purifier systems (GE Healthcare, Uppsala, Sweden) (all the chromatography experiments were performed in cold-room at 4 °C). Briefly, crude sample was slowly injected (400 µl/min) onto pre-equilibrated column Superdex200 10/300 GL (GE healthcare) (Buffer: 20 mM Tris, 120 mM NaCl, 1 mM EDTA. pH 7.4), and the eluate was collected in 500 µl fractions. SDS-PAGE analyzed the resultant fractions confirm the success of purification. All the fractions were separately pooled (peak-wise), concentrated, and stored.

### 4.3. Mass Spectrometric Analysis

Eluates were first analyzed on pre-cast SDS-PAGE gels (Bio-Rad laboratories, Hercules, CA, USA). The protein bands were analyzed by Coomassie staining (Bio-Rad laboratories, USA). Coomassie-stained protein bands were excised and their identity was confirmed while using mass spectrometric analysis, as reported previously [21]. Briefly, peptides were eluted with 25 mM NH_4_HCO_3_; 10% acetonitrile (ACN) and digestion stopped by adding 5% formic acid. The peptides were resolved on a nano-ultra performance LC system coupled to a nano-ESI-MS (nano Acquity UPLC nanoESI Synapt-MS, Waters, Milford, MA, USA) with a 5 µm symmetry 180 µm × 20 mm C18 pre-column and a 1.7 µm BEH 130 100 µm × 100 mm C18 separation column. A 30-min gradient (3% ACN to 40% CAN at 500 nL/min) after three minutes of trapping (99% water at 5 µL/min) was applied to separate peptides. The MS was operated in V mode, acquiring MSE data and applying standard parameters. Data analysis was performed using ProteinLynx Global Server version 2.4 (Waters corporation, Milford, MA, US), searching an in-house database consisting of the Uniprot database (September 2016 version, restricted to reviewed entries of *Homo sapiens*; taxon identifier 9606. Proteins hits were accepted at a false positive rate of less than 4%, as reported previously. For analyses of peaks retained from size exclusion chromatography of FibrogamminP (Figure 2C), Mascot search engine was utilized, which is based on the probabilistic scoring algorithm for protein identification (a detailed report can be found in the Supplementary Information section).

### 4.4. FXIIIa Generation Assay

The rate of activated FXIII (FXIIIa) generation was monitored by FXIIIa Generation Assay [13,21]. Briefly, the generation of active FXIIIa species was monitored in the background of different plasmatic condition. Compared to standard plasma (SP) (ISTH: SCSP FXIII activity 0.76 U/vial; FXIII antigen A_2_B_2_ 0.74 IU/vial), FXIIIa generation was monitored with FXIII-DP (deficient for both FXIII-A and FXIII-B subunits; Haemochrom Diagnostica GmbH, Essen, Germany). Coagulation was triggered by adding tissue factor/phospholipids TF/PI, and FXIII-A (2 IU/mL of plasma) to 25 µl plasma in order to generate active FXIIIa (subsequently detected by FXIII isopeptidase activity on a chromogenic substrate A101 (Zedira GmBh, Darmstadt, Germany)). The reactions spiked with rFXIII-B (20 µg/mL) (Zedira GmBh, Germany), CFH (20 µg/mL), and both rFXIII-B and CFH with respective controls of standard plasma (SP) were incubated with 35 μL reagent solution (5 μL 100 mM glycine methyl ester, 5 μL 2 mM fluorogenic FXIII-A substrate, 10 μL Innovin (recombinant TF; Dade Behring, Liederbach, Germany) diluted 1:2800 in phospholipids (PTT reagent kit, Roche, Mannheim, Germany) and 15 μL HBS (20 mM HEPES, 150 mM NaCl)/0.1% serum albumin pH 7.5. After pre-incubation of the mixture for five minutes, the reaction was started with 40 μL 25 mM CaCl_2_ pH 7.5. Fluorescence was measured over 1 h at λ ex = 330 nm and λ em = 430 nm in kinetic mode two times per minute. The curve data was evaluated according to a bi-exponential model with first order absorption and elimination. The data were fitted to the equation:

C (t) = c_ka/(ka − kb)_ (exp(−kb_ (t − tlag)) − exp(−ka_(t − tlag)))(1)

where ka: constant of absorption, which describes the development of active FXIIIa and kb—elimination constant. The parameters area under the curve (AUC), peak FXIII-A concentration (CP), and time to peak (TTP) were also evaluated [13].

### 4.5. Isolation and Verification of Plasma Sub-Proteome Interacting with FXIII-B by Immunoaffinity Based Pull-Down Assays

The pierce co-immunoprecipitation kit (ThermoFischer Scientific, Rockford, IL, USA) was used to bind the commercial recombinant FXIII-B (Zedira, Germany) to immobilized anti(α)-FXIII-B monoclonal antibodies (in-house) (similar experiments were also performed with rFXIII-B expressed/purified from *HEK293t* mammalian cell lines in-house). Briefly, 75 µl of mouse-α-human-FXIII-B monoclonal antibody (1 mg/mL) (produced in-house) was coupled to amino-link plus coupling resin, according to the manufacturer’s protocol. Firstly, 100 µl of 1mg/mL rFXIII-B (Zedira, Darmstadt, Germany) (or rFXIII-B in-house) was bound to the immobilized antibodies. The following set of experiments followed: (1) the resin bound rFXIII-B was exposed to FXIII-DP; (2) the immobilized α-FXIII-B antibody exposed to FXIII-DP. Additionally, a resin was prepared with α-FXIII-B antibody that was bound to FXIII-A_2_B_2_ complex purified from FibrogamminP and exposed to FXIII-DP (with a negative control that included a resin support with immobilized-monoclonal FXIII-B antibodies, but with no exposure to either recombinant FXIII-B or FXIII concentrate; to rule out the cross-reactivity of this antibody). The bound complexes were eluted with elution buffer (Primary amine pH 2.8; Pierce, Rockford, IL, USA) and SDS-PAGE analyzed the eluates. The bands observed were characterized by peptide mass fingerprinting, followed by mass spectrometry (Supplementary Materials).

## Supplementary Materials

Supplementary materials can be found at https://www.mdpi.com/1422-0067/20/11/2682/s1.

## Abbreviations

FXIII-DP	Factor XIII deficient plasma
CFH	Complement Factor H
FXIIIa	Activated FXIII-A
SP	Standard Plasma
MS	Mass spectrometry
