# Supplementary material for: Identification of Potential Novel Interacting Partners for Coagulation Factor XIII B (FXIII-B) Subunit, a Protein Associated with a Rare Bleeding Disorder
Source: Int J Mol Sci. 2019 May 31;20(11):2682. doi: 10.3390/ijms20112682 (PMC6600159; doi:10.3390/ijms20112682)
Supplement: Supplementary file 1 [file ijms-20-02682-s001.pdf]

Supplementary information

Identification of potential novel interacting partners for coagulation Factor XIII B (FXIII-B) subunit, a protein associated with a rare bleeding disorder

Sneha Singh [†1], Mohammad Suhail Akhter [† 1, 2], Johannes Dodt [3], Peter Volkers [3], Andreas Reuter [3], Christoph Reinhart [4], Christoph Krettler [4], Johannes Oldenburg [1] and Arijit Biswas [1 ††]

1 *Institute of Experimental Hematology and Transfusion Medicine, University Clinic Bonn, 53127 Bonn, Germany*

2 *College of Applied Medical Sciences, Jazan University, 82911, Jazan, Saudi Arabia*

3 *Paul-Ehrich Institute, 63225 Langen, Germany*

4 *Department of Molecular Membrane Biology, Max-Planck Institute for Biophysics, Max-Von Laue Str 3, 60438, Frankfurt am Main, Germany*

† These authors contributed equally to the manuscript

[††] **Correspondence should be addressed to:**

PD Dr. rer. nat. Arijit Biswas, Institute of Experimental Haematology and Transfusion Medicine,
University Clinic Bonn, Sigmund Freud Str. 25, 53127 Bonn, Germany.

Email: arijit.biswas@ukb.uni-bonn.de   Phone: +49 228 287 19428

Twitter:

Supplementary Table:

Table S1: Table depicting the FXIII Generation assay parameters obtained at different spiking conditions.

	AUC	t_{max} (min)	t_{lag} (min)	K_a	K_b
**Standard plasma (SP)**	925,5	15,1	9,9	0,69	0,02
**FXIIIA (2U/mL)**	671,2	29,5	13,1	0,06	0,06
**FXIIIA (1U/mL)**	476,7	28,9	12	0,06	0,06
**FXIIIA (2U/mL) + FXIIIB**	1047,3	18	10,2	0,43	0,02
**FXIIIA (1U/mL) + FXIIIB**	586,3	16,3	9,9	0,53	0,02
**FXIIIA (2U/mL) + CFH**	1005,2	28,2	11,8	0,10	0,04
**FXIIIA (1U/mL) + CFH**	414	26,4	9,9	0,06	0,06
**FXIIIA (2U/mL) +FXIIIB + CFH**	1192,1	17,6	9,1	0,37	0,02
**FXIIIA (1U/mL) + FXIIIB + CFH**	580,4	17,5	9,6	0,37	0,02

Supplementary figures

Figure S1: Peak table from SEC of Fibrogammin P. Highlighted Peak correspond to Proteomic constituents in the sample at specific retention time (in minutes), and Area under peak (mAU*min).

Peak table: Fibrogammin P Large Scale Purification

140616 Fibrogammin Prep Sup200:10_UV1_280nm@01,PEAK

No	Ret min	Area mAU*min	Area/Peak area (time) %	Height mAU
1	0.07	0.3012	0.00	1.688
2	0.35	0.2807	0.00	1.578
3	19.47	253.5121	3.88	140.487
4	23.31	1177.3246	18.01	299.231
5	29.33	148.7012	2.27	79.121
6	34.51	1598.5213	24.45	1638.463
7	34.67	1456.2326	22.27	1663.050
8	45.32	3.0772	0.05	6.396
9	45.68	4.8692	0.07	9.008
10	47.36	19.3370	0.30	33.259
11	48.65	0.2840	0.00	1.252
12	49.13	0.3210	0.00	1.257
13	53.02	1867.6034	28.56	1408.756
14	55.10	1.0523	0.02	7.242
15	55.29	1.2979	0.02	6.757
16	55.54	1.3947	0.02	6.941
17	55.69	3.1471	0.05	6.650
18	56.33	0.7146	0.01	2.661
19	67.13	0.3679	0.01	1.021
20	68.11	0.2931	0.00	1.340

```
Total number of detected peaks = 141
Total area = 6550.5640  mAU*min
Area in evaluated peaks = 6538.6329  mAU*min
Ratio peak area / total area = 0.998179
Total peak width = 29.50 min
Column height = 30.00 cm
Calculated from : 140616 Fibrogammin Prep Sup200:10_UV1_280nm
Baseline : 140616 Fibrogammin Prep Sup200:10_UV1_280nm@01,BASEM
Peak rejection on:
  Maximum number of peaks: 20
Current peak filter settings:
  Maximum number of peaks: 20
```

Figure S2: MS result showing the protein hits corresponding to the bands on SDS PAGE for interactome analyses of FXIIIB (Figure 4, main manuscript).

Band	A	Description	S	SC	E	P	M
A1	P02675	Fibrinogen beta chain Homo sapiens	1324,4	26,1	3,8	9	1
	P02679	Fibrinogen gamma chain Homo sapiens	804,6	30,5	4,9	13	3
	P02671	Fibrinogen alpha chain Homo sapiens	570,3	16,9	5,0	13	5
	P01023	Alpha 2 macroglobulin Homo sapiens	76,6	5,6	3,2	7	1
A2		negative					
A3	P01857	Ig gamma 1 chain C region Homo sapiens	1353,4	26,1	4,2	8	0
	P01834	Ig kappa chain C region Homo sapiens	129,8	31,1	6,7	2	0
A4	P02768	Serum albumin Homo sapiens	456,8	8,0	3,9	9	2
A5	P02747	Complement C1q subcomponent subunit C Homo sapiens	2224,5	21,2	5,2	6	2
C1		negative					
D1	P02747	Complement C1q subcomponent subunit C Homo sapiens	926,2	16,3	17,7	4	0
D2	P02675	Fibrinogen beta chain Homo sapiens	1667,0	31,0	4,8	16	2
	P02679	Fibrinogen gamma chain Homo sapiens	766,4	32,2	5,5	15	3
	P02671	Fibrinogen alpha chain Homo sapiens	551,2	16,9	5,5	15	5
D3	P01876	Ig alpha 1 chain C region Homo sapiens	267,5	5,9	2,1	3	0
	P01024	Complement C3 Homo sapiens	129,2	11,7	3,9	16	3
	P01857	Ig gamma 1 chain C region Homo sapiens	121,8	7,9	2,6	2	0
D4	P01857	Ig gamma 1 chain C region Homo sapiens	1449,9	26,7	4,5	8	0
	P01834	Ig kappa chain C region Homo sapiens	1048,4	32,1	3,1	3	0
D5	P05160	Coagulation factor XIII B chain Homo sapiens	32,8	4,4	15,7	5	0
D6	P05160	Coagulation factor XIII B chain Homo sapiens	1016,9	31,5	3,9	17	4
D7	P02787	Serotransferrin Homo sapiens	64,8	5,4	2,7	3	0
D8	P02768	Serum albumin Homo sapiens	3085,6	45,3	6,0	28	5
E1	P02768	Serum albumin Homo sapiens	143,5	4,6	5,3	3	1
E2	P02768	Serum albumin Homo sapiens	1165,9	12,5	7,1	8	1
E3	P02768	Serum albumin Homo sapiens	161,7	5,9	3,2	3	1
E4	P02768	Serum albumin Homo sapiens	540,4	2,3	1,8	1	0
F1	Q86VM9	Zinc finger CCCH domain containing protein 18 Homo sapiens	26,3	0,8	8,9	1	0
F2	P02768	Serum albumin Homo sapiens	281,2	2,0	6,5	8	1

A: UniProt Accesion number; S: PLGS Protein Score; SC: Sequence coverage in %, E Mean mass error in ppm; P: number of identified peptides; M; number of modigfied peptides

Figure S3: SDS-PAGE analysis of Fibrogammin P. Gel 1 depicts fraction of eluates obtained after Gel filteration of FibrogamminP, when resuspended in 20mM tris 120mM NaCl, pH 7.4 (Column: Superdex200 100/300 GL (GE healthcare)). The numbers on top represent the fraction number when ran at a constant flow rate of 400uL/min. (F is crude fraction). The marked box for fraction 4-6, was re-ran on GFC, with fractions separated on SDS-PAGE (gel 2 here). The marked bands on gel were cut ant analyzed by Mass spectrometry.

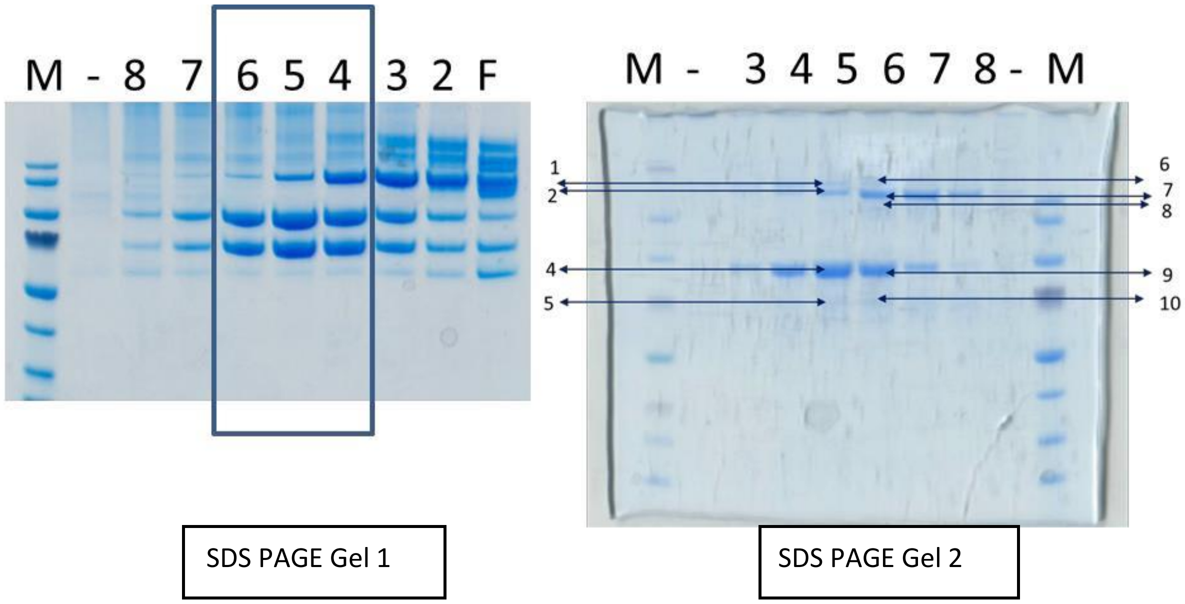

Supplementary Results: The following supplementary attached files are corresponding to the raw data obtained from MS analyses of bands on gel 2 in figure S3. MS analyses were performed based on MASCOT probability based scoring.

Supplementary references

R1. Albrecht M, Alessandri S, Conti A, Reuter A, Lauer I, Vieths S, et al. High level expression, purification and physico- and immunochemical characterization of recombinant Pen a 1: a major allergen of shrimp. Mol. Nutr. Food Res. 2008;52 Suppl 2: S186-S195.

Mascot Search Results

User : Julian
Email : julian.langer@mpibp-frankfurt.mpg.de
Search title : Submitted from 140318 842 ChKrettler 1-10 swissprot full T 0.05Da by Mascot Daemon on CCSW010
MS data file : D:\Data\1403\140318\140314 842 ChReinhard 1_Tray01-C6_01_17682.d\140314 842 ChReinhard 1_Tray01-C6_01_17682.mgf
Database : SwissProt 57.15 (515203 sequences; 181334896 residues)
Timestamp : 19 Mar 2014 at 09:48:02 GMT
Protein hits : A2MG_HUMAN Alpha-2-macroglobulin OS=Homo sapiens GN=A2M PE=1 SV=2
CH60_ECO24 60 kDa chaperonin OS=Escherichia coli O139:H28 (strain E24377A / ETEC) GN=groL PE=3 SV=1
CH60_ENTIT 60 kDa chaperonin (Fragment) OS=Enterobacter intermedius GN=groL PE=3 SV=1
TRYP_PIG Trypsin OS=Sus scrofa PE=1 SV=1
K2C1_HUMAN Keratin, type II cytoskeletal 1 OS=Homo sapiens GN=KRT1 PE=1 SV=6
K1C9_HUMAN Keratin, type I cytoskeletal 9 OS=Homo sapiens GN=KRT9 PE=1 SV=3
CFAH_HUMAN Complement factor H OS=Homo sapiens GN=CFH PE=1 SV=4
K1C10_HUMAN Keratin, type I cytoskeletal 10 OS=Homo sapiens GN=KRT10 PE=1 SV=6
K22E_HUMAN Keratin, type II cytoskeletal 2 epidermal OS=Homo sapiens GN=KRT2 PE=1 SV=2
ALBU_HUMAN Serum albumin OS=Homo sapiens GN=ALB PE=1 SV=2
K2C6B_HUMAN Keratin, type II cytoskeletal 6B OS=Homo sapiens GN=KRT6B PE=1 SV=5
F13B_HUMAN Coagulation factor XIII B chain OS=Homo sapiens GN=F13B PE=1 SV=3
K1C14_HUMAN Keratin, type I cytoskeletal 14 OS=Homo sapiens GN=KRT14 PE=1 SV=4
F13A_HUMAN Coagulation factor XIII A chain OS=Homo sapiens GN=F13A1 PE=1 SV=4
CH602_BURXL 60 kDa chaperonin 2 OS=Burkholderia xenovorans (strain LB400) GN=groL2 PE=3 SV=1

Mascot Score Histogram

Ions score is -10*Log(P), where P is the probability that the observed match is a random event.
Individual ions scores > 38 indicate identity or extensive homology (p<0.05).
Protein scores are derived from ions scores as a non-probabilistic basis for ranking protein hits.

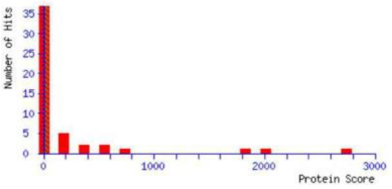

Peptide Summary Report

Format As Peptide Summary Help
Significance threshold p<0.05 Max. number of hits AUTO
Standard scoring MudPIT scoring Ions score or expect cut-off 0 Show sub-sets 0
Show pop-ups Suppress pop-ups Sort unassigned Decreasing Score Require bold red

Select All Select None Search Selected Error tolerant Archive Report

1. A2MG_HUMAN **Mass:** 164614 **Score:** 2744 **Matches:** 130(82) **Sequences:** 56(41) **emPAI:** 2.30
Alpha-2-macroglobulin OS=Homo sapiens GN=A2M PE=1 SV=2
Check to include this hit in error tolerant search or archive report

Query	Observed	Mr(expt)	Mr(calc)	Delta	Miss	Score	Expect	Rank	Unique	Peptide
68	593.3666	592.3593	592.3584	0.0009	0	34	0.19	1	U	K.LSFVK.V
69	593.3673	592.3601	592.3584	0.0016	0	(30)	0.44	1	U	K.LSFVK.V
82	634.3942	633.3869	633.3850	0.0019	0	13	44	1	U	K.VFQLK.R
93	724.4370	723.4297	723.4279	0.0018	0	18	5.2	1	U	K.GVPIPNK.V
96	765.4163	764.4090	764.4068	0.0022	0	25	3	1		R.GEAFTLK.A
101	414.7336	827.4527	827.4501	0.0026	0	63	0.00035	1	U	K.SDIAPVAR.L
102	414.7337	827.4528	827.4501	0.0027	0	(63)	0.00039	1	U	K.SDIAPVAR.L
103	414.7337	827.4529	827.4501	0.0028	0	(48)	0.011	1	U	K.SDIAPVAR.L
112	432.2723	862.5301	862.5276	0.0025	1	24	1.5	1	U	K.TKVFQLK.R
116	442.2850	882.5554	882.5538	0.0015	0	(37)	0.055	1		R.DLKPAIVK.V
117	442.2855	882.5564	882.5538	0.0026	0	(32)	0.19	1		R.DLKPAIVK.V
118	442.2856	882.5566	882.5538	0.0028	0	39	0.035	1		R.DLKPAIVK.V
119	442.2862	882.5578	882.5538	0.0040	0	(33)	0.13	1		R.DLKPAIVK.V
127	463.2787	924.5428	924.5393	0.0036	0	39	0.047	1	U	R.TGTHGLLVK.Q
128	463.2789	924.5432	924.5393	0.0039	0	(18)	5.1	2	U	R.TGTHGLLVK.Q
129	463.2789	924.5433	924.5393	0.0040	0	(28)	0.55	2	U	R.TGTHGLLVK.Q
134	509.8020	1017.5894	1017.5859	0.0035	0	(46)	0.016	1	U	K.ATVLNYLPK.C
135	509.8023	1017.5901	1017.5859	0.0042	0	(31)	0.53	1	U	K.ATVLNYLPK.C
136	509.8024	1017.5902	1017.5859	0.0043	0	48	0.0092	1	U	K.ATVLNYLPK.C
137	509.8027	1017.5908	1017.5859	0.0049	0	(31)	0.55	1	U	K.ATVLNYLPK.C
145	523.7997	1045.5848	1045.5808	0.0040	0	36	0.18	1	U	K.FEVQVTVPK.I
147	523.8002	1045.5859	1045.5808	0.0051	0	(35)	0.25	1	U	K.FEVQVTVPK.I
158	542.8125	1083.6105	1083.6077	0.0028	0	46	0.016	1	U	K.GHFSISIPVK.S
159	542.8133	1083.6119	1083.6077	0.0043	0	(39)	0.065	1	U	K.GHFSISIPVK.S
164	552.3077	1102.6008	1102.5982	0.0026	0	(55)	0.0025	1	U	R.SSGSLLNNAIK.G
165	552.3080	1102.6015	1102.5982	0.0033	0	(51)	0.0067	1	U	R.SSGSLLNNAIK.G
166	552.3083	1102.6020	1102.5982	0.0038	0	59	0.0011	1	U	R.SSGSLLNNAIK.G
170	558.8074	1115.6003	1115.5975	0.0027	0	(77)	1.6e-005	1	U	R.QTVSWAVTPK.S
171	558.8079	1115.6012	1115.5975	0.0036	0	(77)	1.6e-005	1	U	R.QTVSWAVTPK.S
172	558.8082	1115.6019	1115.5975	0.0044	0	77	1.6e-005	1	U	R.QTVSWAVTPK.S
173	560.8235	1119.6324	1119.6288	0.0036	0	(39)	0.074	1	U	K.SIYKPGQTVK.F
174	560.8236	1119.6327	1119.6288	0.0039	0	(32)	0.36	1	U	K.SIYKPGQTVK.F
175	560.8237	1119.6329	1119.6288	0.0041	0	(34)	0.22	1	U	K.SIYKPGQTVK.F
176	560.8239	1119.6332	1119.6288	0.0044	0	42	0.038	1	U	K.SIYKPGQTVK.F
179	567.7944	1133.5742	1133.5750	-0.0008	0	84	2.9e-006	1	U	R.SASNMAIVDVK.M
181	574.8164	1147.6183	1147.6138	0.0044	0	46	0.021	1	U	R.QGIPFFGQVR.L
182	574.8166	1147.6186	1147.6138	0.0048	0	(41)	0.067	1	U	R.QGIPFFGQVR.L
185	575.7933	1149.5720	1149.5700	0.0020	0	(56)	0.0015	1	U	R.SASNMAIVDVK.M + Oxidation (M)
186	575.7935	1149.5725	1149.5700	0.0026	0	(80)	7.4e-006	1	U	R.SASNMAIVDVK.M + Oxidation (M)
187	575.7936	1149.5726	1149.5700	0.0026	0	(74)	3e-005	1	U	R.SASNMAIVDVK.M + Oxidation (M)
202	605.8264	1209.6383	1209.6353	0.0030	0	(50)	0.0053	1	U	K.LPPNVVEESAR.A
203	605.8270	1209.6395	1209.6353	0.0041	0	(50)	0.0052	1	U	K.LPPNVVEESAR.A
204	605.8271	1209.6396	1209.6353	0.0043	0	67	0.00011	1	U	K.LPPNVVEESAR.A
207	613.2816	1224.5487	1224.5445	0.0043	0	50	0.0024	1	U	K.YDVENCLANK.V
208	613.2820	1224.5494	1224.5445	0.0049	0	(39)	0.028	1	U	K.YDVENCLANK.V
220	628.3273	1254.6400	1254.6357	0.0043	0	(63)	0.00026	1	U	K.AIGYLNTGYQR.Q
221	628.3279	1254.6412	1254.6357	0.0056	0	79	7.5e-006	1	U	K.AIGYLNTGYQR.Q
222	630.2920	1258.5694	1258.5652	0.0042	0	(74)	9.2e-006	1	U	R.VGFYESDVMGR.G
223	630.2921	1258.5695	1258.5652	0.0043	0	92	1.4e-007	1	U	R.VGFYESDVMGR.G
224	630.2924	1258.5702	1258.5652	0.0049	0	(83)	1.1e-006	1	U	R.VGFYESDVMGR.G
227	636.8415	1271.6685	1271.6656	0.0029	0	(70)	6.9e-005	1	U	R.VTAAPQSVCALR.A
228	636.8421	1271.6696	1271.6656	0.0040	0	74	2.5e-005	1	U	R.VTAAPQSVCALR.A
229	638.2889	1274.5633	1274.5602	0.0032	0	(48)	0.0028	1	U	R.VGFYESDVMGR.G + Oxidation (M)
230	638.2890	1274.5633	1274.5602	0.0032	0	(54)	0.00072	1	U	R.VGFYESDVMGR.G + Oxidation (M)
242	669.8319	1337.6492	1337.6438	0.0054	0	(63)	0.00028	1	U	R.NALFCLESAWK.T
243	669.8323	1337.6500	1337.6438	0.0062	0	(68)	8.7e-005	1	U	R.NALFCLESAWK.T
244	669.8325	1337.6504	1337.6438	0.0066	0	82	3.3e-006	1	U	R.NALFCLESAWK.T
255	697.8458	1393.6771	1393.6725	0.0046	0	72	3.1e-005	1	U	K.NEDSLVFVQTDK.S
260	471.5446	1411.6119	1411.6085	0.0035	0	(57)	0.00031	1	U	K.EQAPHCICANGR.Q
261	706.8135	1411.6125	1411.6085	0.0040	0	(32)	0.11	1	U	K.EQAPHCICANGR.Q
262	471.5448	1411.6125	1411.6085	0.0040	0	62	0.00011	1	U	K.EQAPHCICANGR.Q
263	709.8044	1417.5943	1417.5899	0.0044	0	(23)	0.35	1	U	K.HYDGSYSTFGER.Y
264	709.8046	1417.5947	1417.5899	0.0048	0	48	0.0012	1	U	K.HYDGSYSTFGER.Y
266	724.8277	1447.6409	1447.6363	0.0046	0	61	0.00014	1	U	K.DMYSFLEDMGLK.A
267	724.8280	1447.6415	1447.6363	0.0052	0	(45)	0.0056	1	U	K.DMYSFLEDMGLK.A
275	756.3885	1510.7625	1510.7627	-0.0002	0	46	0.011	1	U	K.AAQVTIQSSGTFSSK.F
277	515.9404	1544.7993	1544.7947	0.0046	0	(37)	0.12	1	U	R.LVHVEEPHTETVR.K
278	515.9409	1544.8008	1544.7947	0.0061	0	52	0.0042	1	U	R.LVHVEEPHTETVR.K

279	773.4079	1544.8012	1544.7947	0.0065	0	(30)	0.58	1	U	R.LVHVEEPHTETVR.K
280	515.9410	1544.8012	1544.7947	0.0065	0	(38)	0.098	1	U	R.LVHVEEPHTETVR.K
281	519.6093	1555.8062	1555.8028	0.0033	1	(16)	13	1	U	R.TGTHGLLVKQEDMK.G
282	519.6099	1555.8078	1555.8028	0.0050	1	46	0.014	1	U	R.TGTHGLLVKQEDMK.G
283	519.6100	1555.8082	1555.8028	0.0053	1	(34)	0.24	1	U	R.TGTHGLLVKQEDMK.G
284	783.4225	1564.8303	1564.8249	0.0054	0	(66)	0.00012	1	U	K.ALLAYAFALAGNQDK.R
285	783.4227	1564.8309	1564.8249	0.0060	0	87	1e-006	1	U	K.ALLAYAFALAGNQDK.R
286	783.4228	1564.8311	1564.8249	0.0062	0	(67)	0.00011	1	U	K.ALLAYAFALAGNQDK.R
289	524.9412	1571.8018	1571.7977	0.0040	1	(23)	3.3	1	U	R.TGTHGLLVKQEDMK.G + Oxidation (M)
290	802.9288	1603.8430	1603.8358	0.0071	0	82	3.1e-006	1	U	R.IAQWQSFQLEGGLK.Q
293	540.9485	1619.8237	1619.8168	0.0069	0	24	2.3	1	U	K.DNSVHWERPQKPK.A
295	540.9486	1619.8239	1619.8168	0.0071	0	(24)	2.5	1	U	K.DNSVHWERPQKPK.A
304	836.9372	1671.8599	1671.8508	0.0091	0	(38)	0.096	1	U	R.TEHPFTVEEFVLPK.F
305	558.2939	1671.8599	1671.8508	0.0091	0	47	0.011	1	U	R.TEHPFTVEEFVLPK.F
306	558.6395	1672.8966	1672.8897	0.0069	1	28	0.76	1	U	R.LVHVEEPHTETVRK.Y
307	419.2314	1672.8966	1672.8897	0.0069	1	(15)	18	1	U	R.LVHVEEPHTETVRK.Y
310	849.4280	1696.8414	1696.8342	0.0072	0	110	4.7e-009	1	U	K.SSSNEEVMFLTVQVK.G
311	849.4291	1696.8437	1696.8342	0.0095	0	(92)	3.2e-007	1	U	K.SSSNEEVMFLTVQVK.G
314	857.4257	1712.8368	1712.8291	0.0077	0	(108)	6.1e-009	1	U	K.SSSNEEVMFLTVQVK.G + Oxidation (M)
317	577.9650	1730.8730	1730.8662	0.0069	1	11	42	1	U	K.QEDMKGHFSISIPVK.S + Oxidation (M)
322	890.5135	1779.0124	1779.0029	0.0094	0	(26)	0.53	1	U	K.DTVIKPLLVEPEGLEK.E
323	594.0114	1779.0124	1779.0029	0.0094	0	56	0.00054	1	U	K.DTVIKPLLVEPEGLEK.E
324	594.0121	1779.0145	1779.0029	0.0116	0	(39)	0.022	1	U	K.DTVIKPLLVEPEGLEK.E
328	923.0259	1844.0372	1844.0295	0.0077	0	(52)	0.0018	1	U	R.LLIYAVLPTGDVIGDSAK.Y
329	923.0262	1844.0378	1844.0295	0.0083	0	(67)	5.8e-005	1	U	R.LLIYAVLPTGDVIGDSAK.Y
330	923.0268	1844.0391	1844.0295	0.0096	0	69	3.6e-005	1	U	R.LLIYAVLPTGDVIGDSAK.Y
331	924.9489	1847.8833	1847.8730	0.0103	0	66	7.9e-005	1	U	K.QFSFPLSSEPFQGSYK.V
334	942.5313	1883.0481	1883.0404	0.0077	0	72	1.8e-005	1	U	R.VSVQLEASPAFLAVPVEK.E
336	633.9669	1898.8789	1898.8734	0.0055	0	13	12	1	U	K.FSGQLNSHGCFYQQVK.T
337	636.7099	1907.1079	1907.0979	0.0100	1	45	0.0035	1	U	R.KDTVIKPLLVEPEGLEK.E
347	1023.0577	2044.1009	2044.0914	0.0095	0	38	0.063	1	U	R.LLLQQVSLPELPGEYSMK.V
348	683.6917	2048.0532	2048.0439	0.0093	0	19	5.9	1	U	K.VDLSFSPSQSLPASHAHLR.V
351	691.9842	2072.9309	2072.9230	0.0079	0	(45)	0.0034	1	U	K.MCPQLQQYEMHGPEGLR.V
352	691.9845	2072.9315	2072.9230	0.0085	0	(11)	9	1	U	K.MCPQLQQYEMHGPEGLR.V
356	1042.0140	2082.0135	2082.0052	0.0084	0	63	0.0002	1	U	K.AGAFCLSEDAGLGISSTASLR.A
357	1042.0159	2082.0172	2082.0052	0.0121	0	(56)	0.00088	1	U	K.AGAFCLSEDAGLGISSTASLR.A
358	697.3152	2088.9236	2088.9179	0.0057	0	(46)	0.0025	1	U	K.MCPQLQQYEMHGPEGLR.V + Oxidation (M)
359	697.3152	2088.9238	2088.9179	0.0058	0	(22)	0.68	1	U	K.MCPQLQQYEMHGPEGLR.V + Oxidation (M)
360	697.3156	2088.9251	2088.9179	0.0071	0	49	0.0014	1	U	K.MCPQLQQYEMHGPEGLR.V + Oxidation (M)
361	697.3178	2088.9315	2088.9179	0.0135	0	(9)	15	1	U	K.MCPQLQQYEMHGPEGLR.V + Oxidation (M)
362	702.6471	2104.9195	2104.9129	0.0066	0	(26)	0.21	1	U	K.MCPQLQQYEMHGPEGLR.V + 2 Oxidation (M)
363	702.6473	2104.9202	2104.9129	0.0073	0	(41)	0.0077	1	U	K.MCPQLQQYEMHGPEGLR.V + 2 Oxidation (M)
364	702.6477	2104.9212	2104.9129	0.0083	0	(33)	0.045	1	U	K.MCPQLQQYEMHGPEGLR.V + 2 Oxidation (M)
365	704.0334	2109.0784	2109.0702	0.0082	0	(69)	5e-005	1	U	K.LHTEAQIQEEGTVVELTGR.Q
366	704.0339	2109.0798	2109.0702	0.0096	0	94	1.6e-007	1	U	K.LHTEAQIQEEGTVVELTGR.Q
377	745.3002	2232.8787	2232.8688	0.0099	1	30	0.012	1	U	R.KYSDASDCHGEDSQAFCEK.F
392	796.4064	2386.1973	2386.1877	0.0095	0	100	4e-008	1	U	K.QQNAQGGFSSTQDTVVALHALSK.Y
393	796.4069	2386.1988	2386.1877	0.0111	0	(74)	1.4e-005	1	U	K.QQNAQGGFSSTQDTVVALHALSK.Y
394	796.4071	2386.1993	2386.1877	0.0116	0	(91)	2.9e-007	1	U	K.QQNAQGGFSSTQDTVVALHALSK.Y
395	1199.0853	2396.1560	2396.1417	0.0143	0	(12)	22	1	U	K.ETTFNSLLCPSGGEVSEELSLK.L
396	1199.0856	2396.1567	2396.1417	0.0150	0	21	2.4	1	U	K.ETTFNSLLCPSGGEVSEELSLK.L
406	825.7493	2474.2260	2474.2152	0.0109	0	40	0.035	1	U	R.SLFTDLEAENDVLHCVAFAVPK.S
408	832.7745	2495.3016	2495.2908	0.0108	1	16	7.9	1	U	K.NEDSLVFVQTDKSIYKPGQTVK.F
412	1274.5457	2547.0768	2547.0788	-0.0019	0	(44)	0.0012	1	U	K.VYDYYETDEFAIAEYNAPCSK.D
413	1274.5524	2547.0902	2547.0788	0.0115	0	52	0.00021	1	U	K.VYDYYETDEFAIAEYNAPCSK.D
486	942.8207	2825.4402	2825.4310	0.0092	0	(27)	0.64	1	U	R.VVSMDENFHPLNELIPLVYIQDPK.G + Oxidation (M)
487	942.8217	2825.4432	2825.4310	0.0123	0	39	0.037	1	U	R.VVSMDENFHPLNELIPLVYIQDPK.G + Oxidation (M)
488	942.8217	2825.4433	2825.4310	0.0123	0	(24)	1.2	1	U	R.VVSMDENFHPLNELIPLVYIQDPK.G + Oxidation (M)
489	942.8220	2825.4443	2825.4310	0.0133	0	(16)	6.7	1	U	R.VVSMDENFHPLNELIPLVYIQDPK.G + Oxidation (M)
520	1055.1980	3162.5723	3162.5583	0.0140	1	54	0.001	1	U	K.YNILPEKEEFPFALGVQTLPQTCDEPK.A
521	1055.1982	3162.5728	3162.5583	0.0144	1	(39)	0.028	1	U	K.YNILPEKEEFPFALGVQTLPQTCDEPK.A
559	1196.9373	3587.7902	3587.7665	0.0237	1	6	51	1	U	K.ETTFNSLLCPSGGEVSEELSLKLPPNVVEESAR.A

---

2. CH60_ECO24 **Mass:** 57464 **Score:** 2070 **Matches:** 59(45) **Sequences:** 11(11) **emPAI:** 2.84

60 kDa chaperonin OS=Escherichia coli O139:H28 (strain E24377A / ETEC) GN=groL PE=3 SV=1

Check to include this hit in error tolerant search or archive report

Query	Observed	Mr(expt)	Mr(calc)	Delta	Miss	Score	Expect	Rank	Unique	Peptide
287	784.4470	1566.8793	1566.8730	0.0064	0	(70)	3.5e-005	1		R.AAVEEGVVAGGGVALIR.V
288	784.4470	1566.8795	1566.8730	0.0065	0	85	1.2e-006	1		R.AAVEEGVVAGGGVALIR.V
318	880.9573	1759.9001	1759.8952	0.0049	0	(69)	7.1e-005	1		K.AIAQVGTISANSDETVGK.L
319	880.9587	1759.9028	1759.8952	0.0076	0	85	1.9e-006	1		K.AIAQVGTISANSDETVGK.L
342	670.9913	2009.9522	2009.9438	0.0084	1	(28)	0.49	1		R.EIELEDKFENMGAQMVK.E
343	670.9920	2009.9543	2009.9438	0.0105	1	47	0.006	1		R.EIELEDKFENMGAQMVK.E
345	681.6552	2041.9436	2041.9336	0.0100	1	(34)	0.093	1		R.EIELEDKFENMGAQMVK.E + 2 Oxidation (M)
346	681.6552	2041.9439	2041.9336	0.0103	1	(35)	0.072	1		R.EIELEDKFENMGAQMVK.E + 2 Oxidation (M)
398	800.4345	2398.2818	2398.2704	0.0114	1	(85)	8.5e-007	1		R.VVINKDTTTIIDGVGEEAAIQGR.V
399	800.4349	2398.2830	2398.2704	0.0126	1	105	1e-008	1		R.VVINKDTTTIIDGVGEEAAIQGR.V
400	1201.6298	2401.2451	2401.2336	0.0114	0	(61)	0.0003	1		K.ANDAAGDGTTTATVLAQAIITEGLK.A
401	801.4223	2401.2451	2401.2336	0.0114	0	81	2.7e-006	1		K.ANDAAGDGTTTATVLAQAIITEGLK.A
402	801.4224	2401.2455	2401.2336	0.0118	0	(33)	0.17	1		K.ANDAAGDGTTTATVLAQAIITEGLK.A
418	1325.1766	2648.3387	2648.3288	0.0098	0	(64)	0.00013	1		K.AMLQDIATLTGGTVISEEIGMELEK.A
419	883.7868	2648.3387	2648.3288	0.0098	0	(76)	7.8e-006	1		K.AMLQDIATLTGGTVISEEIGMELEK.A
420	883.7870	2648.3393	2648.3288	0.0104	0	(60)	0.0003	1		K.AMLQDIATLTGGTVISEEIGMELEK.A
421	1325.1769	2648.3393	2648.3288	0.0104	0	(73)	1.5e-005	1		K.AMLQDIATLTGGTVISEEIGMELEK.A
422	889.1178	2664.3315	2664.3238	0.0078	0	(34)	0.12	1		K.AMLQDIATLTGGTVISEEIGMELEK.A + Oxidation (M)
423	1333.1731	2664.3315	2664.3238	0.0078	0	(23)	1.5	1		K.AMLQDIATLTGGTVISEEIGMELEK.A + Oxidation (M)
424	1333.1737	2664.3328	2664.3238	0.0090	0	(45)	0.0098	1		K.AMLQDIATLTGGTVISEEIGMELEK.A + Oxidation (M)
425	889.1185	2664.3336	2664.3238	0.0099	0	(91)	2.4e-007	1		K.AMLQDIATLTGGTVISEEIGMELEK.A + Oxidation (M)
426	1333.1741	2664.3336	2664.3238	0.0099	0	(55)	0.00098	1		K.AMLQDIATLTGGTVISEEIGMELEK.A + Oxidation (M)
427	889.1187	2664.3342	2664.3238	0.0104	0	(35)	0.096	1		K.AMLQDIATLTGGTVISEEIGMELEK.A + Oxidation (M)
428	889.1187	2664.3342	2664.3238	0.0105	0	(81)	2.5e-006	1		K.AMLQDIATLTGGTVISEEIGMELEK.A + Oxidation (M)
430	889.1191	2664.3354	2664.3238	0.0117	0	(42)	0.02	1		K.AMLQDIATLTGGTVISEEIGMELEK.A + Oxidation (M)
431	889.1192	2664.3359	2664.3238	0.0121	0	(36)	0.084	1		K.AMLQDIATLTGGTVISEEIGMELEK.A + Oxidation (M)
432	1333.1752	2664.3359	2664.3238	0.0121	0	(77)	5.6e-006	1		K.AMLQDIATLTGGTVISEEIGMELEK.A + Oxidation (M)
433	1333.1753	2664.3359	2664.3238	0.0122	0	(39)	0.04	1		K.AMLQDIATLTGGTVISEEIGMELEK.A + Oxidation (M)
434	889.1193	2664.3360	2664.3238	0.0122	0	109	3.6e-009	1		K.AMLQDIATLTGGTVISEEIGMELEK.A + Oxidation (M)
435	1341.1713	2680.3281	2680.3187	0.0094	0	(26)	0.76	1		K.AMLQDIATLTGGTVISEEIGMELEK.A + 2 Oxidation (M)
436	894.4507	2680.3303	2680.3187	0.0117	0	(61)	0.00025	1		K.AMLQDIATLTGGTVISEEIGMELEK.A + 2 Oxidation (M)
437	894.4508	2680.3307	2680.3187	0.0120	0	(82)	2.2e-006	1		K.AMLQDIATLTGGTVISEEIGMELEK.A + 2 Oxidation (M)
438	1341.1730	2680.3315	2680.3187	0.0128	0	(30)	0.3	1		K.AMLQDIATLTGGTVISEEIGMELEK.A + 2 Oxidation (M)
447	908.5087	2722.5042	2722.4939	0.0103	0	(90)	1.3e-007	1		K.AGKPLLIIAEDVEGEALATLVVNTMR.G
448	908.5089	2722.5047	2722.4939	0.0109	0	109	1.5e-009	1		K.AGKPLLIIAEDVEGEALATLVVNTMR.G
449	908.5092	2722.5057	2722.4939	0.0118	0	(61)	0.0001	1		K.AGKPLLIIAEDVEGEALATLVVNTMR.G
450	908.5093	2722.5060	2722.4939	0.0121	0	(93)	5.5e-008	1		K.AGKPLLIIAEDVEGEALATLVVNTMR.G
453	913.8394	2738.4963	2738.4888	0.0075	0	(67)	2.8e-005	1		K.AGKPLLIIAEDVEGEALATLVVNTMR.G + Oxidation (M)
454	685.6324	2738.5005	2738.4888	0.0117	0	(72)	8.4e-006	1		K.AGKPLLIIAEDVEGEALATLVVNTMR.G + Oxidation (M)
479	931.8177	2792.4313	2792.4187	0.0126	1	(105)	8.8e-009	1		R.KAMLQDIATLTGGTVISEEIGMELEK.A + Oxidation (M)
480	931.8181	2792.4324	2792.4187	0.0137	1	(84)	1.1e-006	1		R.KAMLQDIATLTGGTVISEEIGMELEK.A + Oxidation (M)
481	931.8181	2792.4326	2792.4187	0.0138	1	(84)	1.1e-006	1		R.KAMLQDIATLTGGTVISEEIGMELEK.A + Oxidation (M)
482	937.1476	2808.4209	2808.4136	0.0073	1	111	2.4e-009	1		R.KAMLQDIATLTGGTVISEEIGMELEK.A + 2 Oxidation (M)
483	937.1488	2808.4246	2808.4136	0.0109	1	(96)	7.4e-008	1		R.KAMLQDIATLTGGTVISEEIGMELEK.A + 2 Oxidation (M)
484	937.1491	2808.4254	2808.4136	0.0118	1	(94)	1.2e-007	1		R.KAMLQDIATLTGGTVISEEIGMELEK.A + 2 Oxidation (M)
492	1426.1742	2850.3338	2850.3230	0.0108	0	(4)	63	1	U	K.EGVITVEDGTGLQDELDVVEGMQFDR.G
493	951.1185	2850.3338	2850.3230	0.0108	0	(106)	4.2e-009	1	U	K.EGVITVEDGTGLQDELDVVEGMQFDR.G
494	1426.1748	2850.3351	2850.3230	0.0121	0	(16)	3.7	1	U	K.EGVITVEDGTGLQDELDVVEGMQFDR.G
495	1426.1754	2850.3363	2850.3230	0.0134	0	(40)	0.018	1	U	K.EGVITVEDGTGLQDELDVVEGMQFDR.G
496	951.1194	2850.3363	2850.3230	0.0134	0	112	1.1e-009	1	U	K.EGVITVEDGTGLQDELDVVEGMQFDR.G
498	956.4507	2866.3301	2866.3179	0.0122	0	(100)	1.4e-008	1	U	K.EGVITVEDGTGLQDELDVVEGMQFDR.G + Oxidation (M)
500	956.4510	2866.3311	2866.3179	0.0132	0	(69)	2e-005	1	U	K.EGVITVEDGTGLQDELDVVEGMQFDR.G + Oxidation (M)
515	1037.8861	3110.6365	3110.6216	0.0149	0	(71)	1.5e-005	1		R.GYLSPYFINKPETGAVELESPFILLADK.K
516	1037.8866	3110.6379	3110.6216	0.0163	0	81	1.6e-006	1		R.GYLSPYFINKPETGAVELESPFILLADK.K
517	1037.8868	3110.6385	3110.6216	0.0169	0	(60)	0.00019	1		R.GYLSPYFINKPETGAVELESPFILLADK.K
530	810.6904	3238.7324	3238.7165	0.0159	1	(41)	0.012	1		R.GYLSPYFINKPETGAVELESPFILLADKK.I
531	1080.5847	3238.7324	3238.7165	0.0159	1	(13)	6.3	1		R.GYLSPYFINKPETGAVELESPFILLADKK.I
532	810.6910	3238.7350	3238.7165	0.0185	1	(23)	0.65	1		R.GYLSPYFINKPETGAVELESPFILLADKK.I
534	810.6915	3238.7368	3238.7165	0.0203	1	50	0.0015	1		R.GYLSPYFINKPETGAVELESPFILLADKK.I

Proteins matching the same set of peptides:

CH60_ECO27 **Mass:** 57464 **Score:** 2070 **Matches:** 59(45) **Sequences:** 11(11)

60 kDa chaperonin OS=Escherichia coli O127:H6 (strain E2348/69 / EPEC) GN=groL PE=3 SV=1

CH60_ECO45 **Mass:** 57464 **Score:** 2070 **Matches:** 59(45) **Sequences:** 11(11)

60 kDa chaperonin OS=Escherichia coli O45:K1 (strain S88 / ExPEC) GN=groL PE=3 SV=1

CH60_ECO55 **Mass:** 57464 **Score:** 2070 **Matches:** 59(45) **Sequences:** 11(11)
60 kDa chaperonin OS=Escherichia coli (strain 55989 / EAEC) GN=groL PE=3 SV=1

CH60_ECO57 **Mass:** 57464 **Score:** 2070 **Matches:** 59(45) **Sequences:** 11(11)
60 kDa chaperonin OS=Escherichia coli O157:H7 GN=groL PE=3 SV=2

CH60_ECO5E **Mass:** 57464 **Score:** 2070 **Matches:** 59(45) **Sequences:** 11(11)
60 kDa chaperonin OS=Escherichia coli O157:H7 (strain EC4115 / EHEC) GN=groL PE=3 SV=1

CH60_ECO7I **Mass:** 57464 **Score:** 2070 **Matches:** 59(45) **Sequences:** 11(11)
60 kDa chaperonin OS=Escherichia coli O7:K1 (strain IAI39 / ExPEC) GN=groL PE=3 SV=1

CH60_ECO81 **Mass:** 57464 **Score:** 2070 **Matches:** 59(45) **Sequences:** 11(11)
60 kDa chaperonin OS=Escherichia coli O81 (strain ED1a) GN=groL PE=3 SV=1

CH60_ECO8A **Mass:** 57464 **Score:** 2070 **Matches:** 59(45) **Sequences:** 11(11)
60 kDa chaperonin OS=Escherichia coli O8 (strain IAI1) GN=groL PE=3 SV=1

CH60_ECOBW **Mass:** 57464 **Score:** 2070 **Matches:** 59(45) **Sequences:** 11(11)
60 kDa chaperonin OS=Escherichia coli (strain K12 / BW2952) GN=groL PE=3 SV=1

CH60_ECODH **Mass:** 57464 **Score:** 2070 **Matches:** 59(45) **Sequences:** 11(11)
60 kDa chaperonin OS=Escherichia coli (strain K12 / DH10B) GN=groL PE=3 SV=1

CH60_ECOHS **Mass:** 57464 **Score:** 2070 **Matches:** 59(45) **Sequences:** 11(11)
60 kDa chaperonin OS=Escherichia coli O9:H4 (strain HS) GN=groL PE=3 SV=1

CH60_ECOL5 **Mass:** 57464 **Score:** 2070 **Matches:** 59(45) **Sequences:** 11(11)
60 kDa chaperonin OS=Escherichia coli O6:K15:H31 (strain 536 / UPEC) GN=groL PE=3 SV=1

CH60_ECOL6 **Mass:** 57464 **Score:** 2070 **Matches:** 59(45) **Sequences:** 11(11)
60 kDa chaperonin OS=Escherichia coli O6 GN=groL PE=3 SV=2

CH60_ECOLC **Mass:** 57464 **Score:** 2070 **Matches:** 59(45) **Sequences:** 11(11)
60 kDa chaperonin OS=Escherichia coli (strain ATCC 8739 / DSM 1576 / Crooks) GN=groL PE=3 SV=1

CH60_ECOLI **Mass:** 57464 **Score:** 2070 **Matches:** 59(45) **Sequences:** 11(11)
60 kDa chaperonin OS=Escherichia coli (strain K12) GN=groL PE=1 SV=2

CH60_ECOLU **Mass:** 57464 **Score:** 2070 **Matches:** 59(45) **Sequences:** 11(11)
60 kDa chaperonin OS=Escherichia coli O17:K52:H18 (strain UMN026 / ExPEC) GN=groL PE=3 SV=1

CH60_ECOSE **Mass:** 57464 **Score:** 2070 **Matches:** 59(45) **Sequences:** 11(11)
60 kDa chaperonin OS=Escherichia coli (strain SE11) GN=groL PE=3 SV=1

CH60_ECOSM **Mass:** 57464 **Score:** 2070 **Matches:** 59(45) **Sequences:** 11(11)
60 kDa chaperonin OS=Escherichia coli (strain SMS-3-5 / SECEC) GN=groL PE=3 SV=1

CH60_ECOUT **Mass:** 57464 **Score:** 2070 **Matches:** 59(45) **Sequences:** 11(11)
60 kDa chaperonin OS=Escherichia coli (strain UTI89 / UPEC) GN=groL PE=1 SV=1

CH60_SHIB3 **Mass:** 57464 **Score:** 2070 **Matches:** 59(45) **Sequences:** 11(11)
60 kDa chaperonin OS=Shigella boydii serotype 18 (strain CDC 3083-94 / BS512) GN=groL PE=3 SV=1

CH60_SHIBS **Mass:** 57464 **Score:** 2070 **Matches:** 59(45) **Sequences:** 11(11)
60 kDa chaperonin OS=Shigella boydii serotype 4 (strain Sb227) GN=groL PE=3 SV=1

CH60_SHIDS **Mass:** 57464 **Score:** 2070 **Matches:** 59(45) **Sequences:** 11(11)
60 kDa chaperonin OS=Shigella dysenteriae serotype 1 (strain Sd197) GN=groL PE=3 SV=1

CH60_SHIF8 **Mass:** 57464 **Score:** 2070 **Matches:** 59(45) **Sequences:** 11(11)
60 kDa chaperonin OS=Shigella flexneri serotype 5b (strain 8401) GN=groL PE=3 SV=1

CH60_SHIFL **Mass:** 57464 **Score:** 2070 **Matches:** 59(45) **Sequences:** 11(11)
60 kDa chaperonin OS=Shigella flexneri GN=groL PE=3 SV=2

CH60_SHISS **Mass:** 57464 **Score:** 2070 **Matches:** 59(45) **Sequences:** 11(11)
60 kDa chaperonin OS=Shigella sonnei (strain Ss046) GN=groL PE=3 SV=1

CH601_ECOK1 **Mass:** 57464 **Score:** 2070 **Matches:** 59(45) **Sequences:** 11(11)
60 kDa chaperonin 1 OS=Escherichia coli O1:K1 / APEC GN=groL1 PE=3 SV=1

---

3. CH60_ENTIT **Mass:** 56741 **Score:** 1801 **Matches:** 51(39) **Sequences:** 10(10) **emPAI:** 2.09
60 kDa chaperonin (Fragment) OS=Enterobacter intermedius GN=groL PE=3 SV=1

☐ Check to include this hit in error tolerant search or archive report

Query	Observed	Mr(expt)	Mr(calc)	Delta	Miss	Score	Expect	Rank	Unique	Peptide
287	784.4470	1566.8793	1566.8730	0.0064	0	(70)	3.5e-005	1		R.AAVEEGVVAGGGVALIR.V
288	784.4470	1566.8795	1566.8730	0.0065	0	85	1.2e-006	1		R.AAVEEGVVAGGGVALIR.V
318	880.9573	1759.9001	1759.8952	0.0049	0	(69)	7.1e-005	1		K.AIAQVGTISANSDETVGK.L
319	880.9587	1759.9028	1759.8952	0.0076	0	85	1.9e-006	1		K.AIAQVGTISANSDETVGK.L
342	670.9913	2009.9522	2009.9438	0.0084	1	(28)	0.49	1		R.EIELEDKFENMGAQMVK.E
343	670.9920	2009.9543	2009.9438	0.0105	1	47	0.006	1		R.EIELEDKFENMGAQMVK.E
345	681.6552	2041.9436	2041.9336	0.0100	1	(34)	0.093	1		R.EIELEDKFENMGAQMVK.E + 2 Oxidation (M)
346	681.6552	2041.9439	2041.9336	0.0103	1	(35)	0.072	1		R.EIELEDKFENMGAQMVK.E + 2 Oxidation (M)
398	800.4345	2398.2818	2398.2704	0.0114	1	(85)	8.5e-007	1		R.VVINKDTTTIIDGVGEEAAIQGR.V
399	800.4349	2398.2830	2398.2704	0.0126	1	105	1e-008	1		R.VVINKDTTTIIDGVGEEAAIQGR.V
418	1325.1766	2648.3387	2648.3288	0.0098	0	(64)	0.00013	1		K.AMLQDIATLTGGTVISEEIGMELEK.A
419	883.7868	2648.3387	2648.3288	0.0098	0	(76)	7.8e-006	1		K.AMLQDIATLTGGTVISEEIGMELEK.A
420	883.7870	2648.3393	2648.3288	0.0104	0	(60)	0.0003	1		K.AMLQDIATLTGGTVISEEIGMELEK.A
421	1325.1769	2648.3393	2648.3288	0.0104	0	(73)	1.5e-005	1		K.AMLQDIATLTGGTVISEEIGMELEK.A
422	889.1178	2664.3315	2664.3238	0.0078	0	(34)	0.12	1		K.AMLQDIATLTGGTVISEEIGMELEK.A + Oxidation (M)
423	1333.1731	2664.3315	2664.3238	0.0078	0	(23)	1.5	1		K.AMLQDIATLTGGTVISEEIGMELEK.A + Oxidation (M)
424	1333.1737	2664.3328	2664.3238	0.0090	0	(45)	0.0098	1		K.AMLQDIATLTGGTVISEEIGMELEK.A + Oxidation (M)
425	889.1185	2664.3336	2664.3238	0.0099	0	(91)	2.4e-007	1		K.AMLQDIATLTGGTVISEEIGMELEK.A + Oxidation (M)
426	1333.1741	2664.3336	2664.3238	0.0099	0	(55)	0.00098	1		K.AMLQDIATLTGGTVISEEIGMELEK.A + Oxidation (M)
427	889.1187	2664.3342	2664.3238	0.0104	0	(35)	0.096	1		K.AMLQDIATLTGGTVISEEIGMELEK.A + Oxidation (M)
428	889.1187	2664.3342	2664.3238	0.0105	0	(81)	2.5e-006	1		K.AMLQDIATLTGGTVISEEIGMELEK.A + Oxidation (M)
430	889.1191	2664.3354	2664.3238	0.0117	0	(42)	0.02	1		K.AMLQDIATLTGGTVISEEIGMELEK.A + Oxidation (M)
431	889.1192	2664.3359	2664.3238	0.0121	0	(36)	0.084	1		K.AMLQDIATLTGGTVISEEIGMELEK.A + Oxidation (M)
432	1333.1752	2664.3359	2664.3238	0.0121	0	(77)	5.6e-006	1		K.AMLQDIATLTGGTVISEEIGMELEK.A + Oxidation (M)
433	1333.1753	2664.3359	2664.3238	0.0122	0	(39)	0.04	1		K.AMLQDIATLTGGTVISEEIGMELEK.A + Oxidation (M)
434	889.1193	2664.3360	2664.3238	0.0122	0	109	3.6e-009	1		K.AMLQDIATLTGGTVISEEIGMELEK.A + Oxidation (M)
435	1341.1713	2680.3281	2680.3187	0.0094	0	(26)	0.76	1		K.AMLQDIATLTGGTVISEEIGMELEK.A + 2 Oxidation (M)
436	894.4507	2680.3303	2680.3187	0.0117	0	(61)	0.00025	1		K.AMLQDIATLTGGTVISEEIGMELEK.A + 2 Oxidation (M)
437	894.4508	2680.3307	2680.3187	0.0120	0	(82)	2.2e-006	1		K.AMLQDIATLTGGTVISEEIGMELEK.A + 2 Oxidation (M)
438	1341.1730	2680.3315	2680.3187	0.0128	0	(30)	0.3	1		K.AMLQDIATLTGGTVISEEIGMELEK.A + 2 Oxidation (M)
447	908.5087	2722.5042	2722.4939	0.0103	0	(90)	1.3e-007	1		K.AGKPLLIIAEDVEGEALATLVVNTMR.G
448	908.5089	2722.5047	2722.4939	0.0109	0	109	1.5e-009	1		K.AGKPLLIIAEDVEGEALATLVVNTMR.G
449	908.5092	2722.5057	2722.4939	0.0118	0	(61)	0.0001	1		K.AGKPLLIIAEDVEGEALATLVVNTMR.G
450	908.5093	2722.5060	2722.4939	0.0121	0	(93)	5.5e-008	1		K.AGKPLLIIAEDVEGEALATLVVNTMR.G
453	913.8394	2738.4963	2738.4888	0.0075	0	(67)	2.8e-005	1		K.AGKPLLIIAEDVEGEALATLVVNTMR.G + Oxidation (M)
454	685.6324	2738.5005	2738.4888	0.0117	0	(72)	8.4e-006	1		K.AGKPLLIIAEDVEGEALATLVVNTMR.G + Oxidation (M)
479	931.8177	2792.4313	2792.4187	0.0126	1	(105)	8.8e-009	1		R.KAMLQDIATLTGGTVISEEIGMELEK.A + Oxidation (M)
480	931.8181	2792.4324	2792.4187	0.0137	1	(84)	1.1e-006	1		R.KAMLQDIATLTGGTVISEEIGMELEK.A + Oxidation (M)
481	931.8181	2792.4326	2792.4187	0.0138	1	(84)	1.1e-006	1		R.KAMLQDIATLTGGTVISEEIGMELEK.A + Oxidation (M)
482	937.1476	2808.4209	2808.4136	0.0073	1	111	2.4e-009	1		R.KAMLQDIATLTGGTVISEEIGMELEK.A + 2 Oxidation (M)
483	937.1488	2808.4246	2808.4136	0.0109	1	(96)	7.4e-008	1		R.KAMLQDIATLTGGTVISEEIGMELEK.A + 2 Oxidation (M)
484	937.1491	2808.4254	2808.4136	0.0118	1	(94)	1.2e-007	1		R.KAMLQDIATLTGGTVISEEIGMELEK.A + 2 Oxidation (M)
☑ **501**	**956.7828**	**2867.3267**	**2867.3019**	**0.0248**	**0**	**88**	**2.1e-007**	**1**		**K.EGVITVEDGTGLEDELDVVEGMQFDR.G + Oxidation (M)**
☑ **502**	**1434.6706**	**2867.3267**	**2867.3019**	**0.0248**	**0**	**(12)**	**7.7**	**1**		**K.EGVITVEDGTGLEDELDVVEGMQFDR.G + Oxidation (M)**
515	1037.8861	3110.6365	3110.6216	0.0149	0	(71)	1.5e-005	1		R.GYLSPYFINKPETGAVELESPFILLADK.K
516	1037.8866	3110.6379	3110.6216	0.0163	0	81	1.6e-006	1		R.GYLSPYFINKPETGAVELESPFILLADK.K
517	1037.8868	3110.6385	3110.6216	0.0169	0	(60)	0.00019	1		R.GYLSPYFINKPETGAVELESPFILLADK.K
530	810.6904	3238.7324	3238.7165	0.0159	1	(41)	0.012	1		R.GYLSPYFINKPETGAVELESPFILLADKK.I
531	1080.5847	3238.7324	3238.7165	0.0159	1	(13)	6.3	1		R.GYLSPYFINKPETGAVELESPFILLADKK.I
532	810.6910	3238.7350	3238.7165	0.0185	1	(23)	0.65	1		R.GYLSPYFINKPETGAVELESPFILLADKK.I
534	810.6915	3238.7368	3238.7165	0.0203	1	50	0.0015	1		R.GYLSPYFINKPETGAVELESPFILLADKK.I

---

4. TRYP_PIG **Mass:** 25078 **Score:** 730 **Matches:** 21(20) **Sequences:** 7(7) **emPAI:** 2.29

Trypsin OS=Sus scrofa PE=1 SV=1

Check to include this hit in error tolerant search or archive report

Query	Observed	Mr(expt)	Mr(calc)	Delta	Miss	Score	Expect	Rank	Unique	Peptide
107	421.7597	841.5048	841.5022	0.0027	0	(51)	0.0052	1	U	R.VATVSLPR.S
108	421.7598	841.5049	841.5022	0.0028	0	51	0.005	1	U	R.VATVSLPR.S
149	532.2587	1062.5029	1062.5016	0.0013	0	(47)	0.012	1	U	K.APVLSDSSCK.S
150	532.2596	1062.5047	1062.5016	0.0031	0	(94)	2.2e-007	1	U	K.APVLSDSSCK.S
151	532.2599	1062.5052	1062.5016	0.0037	0	94	2.2e-007	1	U	K.APVLSDSSCK.S
276	763.8817	1525.7488	1525.7446	0.0042	0	54	0.002	1	U	K.SSGSSYPSLLQCLK.A
371	737.7097	2210.1072	2210.0967	0.0105	0	(93)	1.8e-007	1	U	R.LGEHNIDVLEGNEQFINAAK.I
372	737.7098	2210.1077	2210.0967	0.0110	0	(100)	4e-008	1	U	R.LGEHNIDVLEGNEQFINAAK.I
373	737.7105	2210.1096	2210.0967	0.0129	0	110	4.3e-009	1	U	R.LGEHNIDVLEGNEQFINAAK.I
379	761.7355	2282.1847	2282.1729	0.0118	0	(52)	0.0021	1	U	K.IITHPNFNGNTLDNDIMLIK.L
381	761.7357	2282.1852	2282.1729	0.0124	0	70	3.3e-005	1	U	K.IITHPNFNGNTLDNDIMLIK.L
382	761.7361	2282.1865	2282.1729	0.0136	0	(54)	0.0013	1	U	K.IITHPNFNGNTLDNDIMLIK.L
386	767.0664	2298.1774	2298.1678	0.0096	0	(63)	0.0002	1	U	K.IITHPNFNGNTLDNDIMLIK.L + Oxidation (M)
387	767.0670	2298.1793	2298.1678	0.0115	0	(47)	0.0064	1	U	K.IITHPNFNGNTLDNDIMLIK.L + Oxidation (M)
442	903.1482	2706.4228	2706.4089	0.0139	1	(10)	23	1	U	R.IQVRLGEHNIDVLEGNEQFINAAK.I
443	677.6130	2706.4229	2706.4089	0.0140	1	41	0.018	1	U	R.IQVRLGEHNIDVLEGNEQFINAAK.I
543	828.1916	3308.7373	3308.7187	0.0187	1	65	5.5e-005	1	U	K.IITHPNFNGNTLDNDIMLIKLSSPATLNSR.V
545	828.1919	3308.7385	3308.7187	0.0198	1	(59)	0.00022	1	U	K.IITHPNFNGNTLDNDIMLIKLSSPATLNSR.V
546	828.1919	3308.7387	3308.7187	0.0200	1	(61)	0.00012	1	U	K.IITHPNFNGNTLDNDIMLIKLSSPATLNSR.V
548	832.1890	3324.7270	3324.7136	0.0134	1	(36)	0.047	1	U	K.IITHPNFNGNTLDNDIMLIKLSSPATLNSR.V + Oxidation (M)
549	832.1902	3324.7318	3324.7136	0.0182	1	(36)	0.049	1	U	K.IITHPNFNGNTLDNDIMLIKLSSPATLNSR.V + Oxidation (M)

5. K2C1_HUMAN **Mass:** 66170 **Score:** 622 **Matches:** 28(19) **Sequences:** 16(12) **emPAI:** 0.94

Keratin, type II cytoskeletal 1 OS=Homo sapiens GN=KRT1 PE=1 SV=6

Check to include this hit in error tolerant search or archive report

Query	Observed	Mr(expt)	Mr(calc)	Delta	Miss	Score	Expect	Rank	Unique	Peptide
105	416.7490	831.4835	831.4814	0.0021	0	26	2.7	2	U	K.SISISVAR.G
114	437.7546	873.4946	873.4920	0.0027	0	51	0.0083	1	U	R.SLVNLGGSK.S
115	437.7549	873.4952	873.4920	0.0033	0	(51)	0.0084	1	U	R.SLVNLGGSK.S
132	487.2707	972.5268	972.5240	0.0029	0	32	0.6	1		K.IEISELNR.V
156	533.2655	1064.5164	1064.5138	0.0026	0	56	0.0014	1	U	K.AQYEDIAQK.S
157	533.2657	1064.5169	1064.5138	0.0031	0	(27)	1.1	1	U	K.AQYEDIAQK.S
192	590.3040	1178.5935	1178.5931	0.0004	0	54	0.0029	1		K.YEELQITAGR.H
199	401.2358	1200.6856	1200.6826	0.0030	2	30	0.65	1	U	R.LRSEIDNVKK.Q
200	401.2362	1200.6866	1200.6826	0.0040	2	(24)	2.6	1	U	R.LRSEIDNVKK.Q
201	401.2363	1200.6870	1200.6826	0.0044	2	(25)	2	1	U	R.LRSEIDNVKK.Q
226	633.3233	1264.6321	1264.6299	0.0022	0	62	0.00037	1	U	R.TNAENEFVTIK.K
231	639.3608	1276.7071	1276.7027	0.0044	0	(64)	0.0002	1	U	K.LALDLEIATYR.T
232	639.3613	1276.7081	1276.7027	0.0054	0	(61)	0.00047	1	U	K.LALDLEIATYR.T
233	639.3614	1276.7083	1276.7027	0.0057	0	65	0.00019	1	U	K.LALDLEIATYR.T
235	651.8634	1301.7123	1301.7078	0.0045	0	(47)	0.013	1		R.SLDLDSIIAEVK.A
236	651.8637	1301.7128	1301.7078	0.0050	0	97	1.4e-007	1		R.SLDLDSIIAEVK.A
250	692.3522	1382.6899	1382.6830	0.0069	0	61	0.00038	1	U	K.SLNNQFASFIDK.V
251	465.2507	1392.7302	1392.7249	0.0053	1	(68)	8.4e-005	1	U	R.TNAENEFVTIKK.D
252	697.3724	1392.7302	1392.7249	0.0053	1	(73)	3.1e-005	1	U	R.TNAENEFVTIKK.D
253	697.3726	1392.7306	1392.7249	0.0057	1	79	7.6e-006	1	U	R.TNAENEFVTIKK.D
254	465.2508	1392.7306	1392.7249	0.0057	1	(78)	1e-005	1	U	R.TNAENEFVTIKK.D
270	738.3928	1474.7710	1474.7780	-0.0070	0	(26)	1.8	1		R.FLEQQNQVLQTK.W
271	738.3986	1474.7826	1474.7780	0.0046	0	91	5e-007	1		R.FLEQQNQVLQTK.W
315	858.9330	1715.8515	1715.8438	0.0077	0	66	0.00014	1	U	K.QISNLQQSISDAEQR.G
370	728.7165	2183.1277	2183.1182	0.0096	2	53	0.0022	1	U	K.NKLNDLEDALQQAKEDLAR.L
388	777.0660	2328.1761	2328.1669	0.0092	1	42	0.024	1	U	K.QISNLQQSISDAEQRGENALK.D
389	777.0667	2328.1784	2328.1669	0.0115	1	(30)	0.43	1	U	K.QISNLQQSISDAEQRGENALK.D
417	661.5919	2642.3386	2642.3259	0.0126	2	6	84	1	U	K.QISNLQQSISDAEQRGENALKDAK.N

Proteins matching the same set of peptides:

K2C1_PANTR **Mass:** 65621 **Score:** 622 **Matches:** 28(19) **Sequences:** 16(12)

Keratin, type II cytoskeletal 1 OS=Pan troglodytes GN=KRT1 PE=2 SV=1

6. K1C9_HUMAN **Mass:** 62255 **Score:** 488 **Matches:** 17(11) **Sequences:** 10(6) **emPAI:** 0.63

Keratin, type I cytoskeletal 9 OS=Homo sapiens GN=KRT9 PE=1 SV=3

Check to include this hit in error tolerant search or archive report

Query	Observed	Mr(expt)	Mr(calc)	Delta	Miss	Score	Expect	Rank	Unique	Peptide
148	530.7876	1059.5605	1059.5560	0.0045	0	38	0.13	1	U	K.TLLDIDNTR.M
154	533.2552	1064.4958	1064.4920	0.0038	0	(24)	1.8	1	U	K.STMQELNSR.L
155	533.2556	1064.4966	1064.4920	0.0046	0	34	0.16	1	U	K.STMQELNSR.L
189	579.3008	1156.5870	1156.5836	0.0033	0	(63)	0.00027	1	U	R.QGVDADINGLR.Q
190	579.3012	1156.5878	1156.5836	0.0041	0	68	9.5e-005	1	U	R.QGVDADINGLR.Q
209	616.8047	1231.5949	1231.5906	0.0043	0	(99)	6.6e-008	1	U	R.SGGGGGGGLGSGGSIR.S
210	616.8052	1231.5958	1231.5906	0.0053	0	113	2.7e-009	1	U	R.SGGGGGGGLGSGGSIR.S
214	618.2698	1234.5250	1234.5215	0.0036	0	78	2.1e-006	1	U	R.FSSSSGYGGGSSR.V
215	618.2704	1234.5262	1234.5215	0.0048	0	(64)	5.8e-005	1	U	R.FSSSSGYGGGSSR.V
216	618.2704	1234.5263	1234.5215	0.0048	0	(55)	0.00039	1	U	R.FSSSSGYGGGSSR.V
327	613.3296	1836.9668	1836.9581	0.0087	0	49	0.0059	1	U	R.HGVQELEIELQSQLSK.K
332	617.9838	1850.9296	1850.9196	0.0100	1	29	0.62	1	U	K.TLNDMRQEYEQLIAK.N
409	837.3868	2509.1385	2509.1245	0.0140	0	44	0.0043	1	U	K.EIETYHNLLEGGQEDFESSGAGK.I
525	1075.1033	3222.2882	3222.2744	0.0138	0	10	0.43	1	U	R.GGSGGSHGGGSGFGGESGGSYGGGEEASGSGGGYGGGSGK.S
536	1088.8485	3263.5235	3263.5066	0.0170	0	62	8.1e-005	1	U	K.DIENQYETQITQIEHEVSSSGQEVQSSAK.E
537	1088.8486	3263.5240	3263.5066	0.0175	0	(60)	0.00012	1	U	K.DIENQYETQITQIEHEVSSSGQEVQSSAK.E
538	1088.8487	3263.5243	3263.5066	0.0178	0	(22)	0.72	1	U	K.DIENQYETQITQIEHEVSSSGQEVQSSAK.E

7. CFAH_HUMAN **Mass:** 143680 **Score:** 320 **Matches:** 19(11) **Sequences:** 13(8) **emPAI:** 0.30

Complement factor H OS=Homo sapiens GN=CFH PE=1 SV=4

Check to include this hit in error tolerant search or archive report

Query	Observed	Mr(expt)	Mr(calc)	Delta	Miss	Score	Expect	Rank	Unique	Peptide
125	463.2312	924.4477	924.4454	0.0024	0	38	0.077	1	U	R.NGFYPATR.G
126	463.2316	924.4487	924.4454	0.0034	0	(23)	2.2	1	U	R.NGFYPATR.G
152	532.2994	1062.5842	1062.5822	0.0020	0	(51)	0.0064	1	U	K.IDVHLVPDR.K
153	532.2994	1062.5842	1062.5822	0.0020	0	53	0.0034	1	U	K.IDVHLVPDR.K
178	566.2637	1130.5129	1130.5105	0.0024	0	32	0.17	1	U	K.EFDHNSNIR.Y
195	593.3023	1184.5900	1184.5860	0.0041	0	57	0.00096	1	U	K.CLPVTAPENGK.I
196	593.8005	1185.5865	1185.5819	0.0046	1	29	0.69	1	U	R.TKNDFTWFK.L
197	595.2705	1188.5265	1188.5234	0.0031	0	34	0.08	1	U	K.LSYTCEGGFR.I
217	621.7762	1241.5378	1241.5347	0.0031	0	40	0.015	1	U	R.TGDEITYQCR.N
218	623.3082	1244.6018	1244.5972	0.0046	0	64	0.00019	1	U	K.CTSTGWIPAPR.C
225	631.7980	1261.5813	1261.5761	0.0052	0	14	13	1	U	K.DGWSAQPTCIK.S
245	671.3568	1340.6990	1340.6936	0.0054	0	54	0.002	1	U	K.SPDVINGSPISQK.I
248	683.3478	1364.6811	1364.6758	0.0054	0	46	0.015	1	U	R.EIMENYNIALR.W
256	698.3983	1394.7820	1394.7769	0.0052	0	48	0.0071	1	U	K.SSNLIILEEHLK.N
257	698.3984	1394.7822	1394.7769	0.0053	0	(45)	0.013	1	U	K.SSNLIILEEHLK.N
258	698.4002	1394.7858	1394.7769	0.0089	0	(33)	0.19	1	U	K.SSNLIILEEHLK.N
456	914.1059	2739.2959	2739.2844	0.0115	1	64	7.6e-005	1	U	K.VSVLCQENYLIQEGEEITCKDGR.W
457	914.1067	2739.2984	2739.2844	0.0140	1	(46)	0.0048	1	U	K.VSVLCQENYLIQEGEEITCKDGR.W
458	914.1070	2739.2992	2739.2844	0.0148	1	(23)	1.1	1	U	K.VSVLCQENYLIQEGEEITCKDGR.W

8. K1C10_HUMAN **Mass:** 59020 **Score:** 313 **Matches:** 22(11) **Sequences:** 12(7) **emPAI:** 0.67

Keratin, type I cytoskeletal 10 OS=Homo sapiens GN=KRT10 PE=1 SV=6

Check to include this hit in error tolerant search or archive report

Query	Observed	Mr(expt)	Mr(calc)	Delta	Miss	Score	Expect	Rank	Unique	Peptide
98	404.2046	806.3947	806.3923	0.0024	0	(38)	0.15	1		R.LAADDFR.L
99	404.2048	806.3950	806.3923	0.0027	0	(47)	0.016	1		R.LAADDFR.L
100	404.2050	806.3954	806.3923	0.0032	0	62	0.00056	1		R.LAADDFR.L
133	498.2656	994.5166	994.5123	0.0042	1	32	0.35	1		K.IKEWYEK.H
139	516.3046	1030.5946	1030.5910	0.0036	0	27	1.3	1		R.VLDELTLTK.A
160	545.7700	1089.5254	1089.5237	0.0017	0	(34)	0.27	1		K.VTMQNLNDR.L
161	545.7705	1089.5265	1089.5237	0.0029	0	54	0.0022	1		K.VTMQNLNDR.L

Query	Observed	Mr(expt)	Mr(calc)	Delta	Miss	Score	Expect	Rank	Unique	Peptide
162	545.7707	1089.5268	1089.5237	0.0031	0	(54)	0.0022	1		K.VTMQNLNDR.L
168	555.2483	1108.4821	1108.4825	-0.0004	0	47	0.0039	1		K.DAEAWFNEK.S
169	555.2509	1108.4872	1108.4825	0.0047	0	(39)	0.028	1		K.DAEAWFNEK.S
211	617.8466	1233.6786	1233.6717	0.0069	1	40	0.068	1		R.LKYENEVALR.Q
212	617.8467	1233.6788	1233.6717	0.0071	1	(25)	1.9	1		R.LKYENEVALR.Q
213	617.8470	1233.6794	1233.6717	0.0077	1	(8)	98	1		R.LKYENEVALR.Q
247	679.3649	1356.7151	1356.7110	0.0042	1	14	27	1		R.QSVEADINGLRR.V
249	691.3300	1380.6455	1380.6408	0.0047	0	58	0.00055	1		R.ALEESNYELEGK.I
272	747.3735	1492.7324	1492.7270	0.0054	1	(28)	0.85	1	U	R.SQYEQLAEQNRK.D
273	747.3736	1492.7327	1492.7270	0.0057	1	42	0.031	1	U	R.SQYEQLAEQNRK.D
274	747.3742	1492.7339	1492.7270	0.0069	1	(37)	0.11	1	U	R.SQYEQLAEQNRK.D
312	854.3928	1706.7711	1706.7649	0.0062	0	(48)	0.0031	1	U	K.GSLGGGFSSGGFSGGSFSR.G
313	854.3929	1706.7712	1706.7649	0.0063	0	86	5e-007	1	U	K.GSLGGGFSSGGFSGGSFSR.G
325	899.0123	1796.0101	1796.0043	0.0058	0	58	0.00042	1	U	R.NVQALEIELQSQLALK.Q
374	738.0407	2211.1001	2211.0906	0.0095	1	30	0.35	1	U	K.SKELTTEIDNNIEQISSYK.S

9. K22E_HUMAN **Mass:** 65678 **Score:** 255 **Matches:** 10(6) **Sequences:** 7(4) **emPAI:** 0.29
Keratin, type II cytoskeletal 2 epidermal OS=Homo sapiens GN=KRT2 PE=1 SV=2
Check to include this hit in error tolerant search or archive report

Query	Observed	Mr(expt)	Mr(calc)	Delta	Miss	Score	Expect	Rank	Unique	Peptide
104	416.2465	830.4784	830.4862	-0.0078	0	27	2.1	1	U	R.SLVGLGGTK.S
132	487.2707	972.5268	972.5240	0.0029	0	32	0.6	1		K.IEISELNR.V
167	554.2769	1106.5392	1106.5356	0.0036	0	38	0.095	1		K.AQYEEIAQR.S
219	627.8086	1253.6027	1253.6001	0.0027	0	45	0.014	1		R.GFSSGSAVVSGGSR.R
240	665.3693	1328.7241	1328.7187	0.0054	0	(46)	0.015	1		R.NLDLDSIIAEVK.A
241	665.3699	1328.7252	1328.7187	0.0065	0	73	3.3e-005	1		R.NLDLDSIIAEVK.A
268	730.9056	1459.7966	1459.7922	0.0044	0	86	1.7e-006	1	U	K.VDLLNQEIEFLK.V
269	730.9060	1459.7974	1459.7922	0.0052	0	(63)	0.00033	1	U	K.VDLLNQEIEFLK.V
270	738.3928	1474.7710	1474.7780	-0.0070	0	(26)	1.8	1		R.FLEQQNQVLQTK.W
271	738.3986	1474.7826	1474.7780	0.0046	0	91	5e-007	1		R.FLEQQNQVLQTK.W

10. ALBU_HUMAN **Mass:** 71317 **Score:** 147 **Matches:** 6(5) **Sequences:** 3(2) **emPAI:** 0.15
Serum albumin OS=Homo sapiens GN=ALB PE=1 SV=2
Check to include this hit in error tolerant search or archive report

Query	Observed	Mr(expt)	Mr(calc)	Delta	Miss	Score	Expect	Rank	Unique	Peptide
183	575.3129	1148.6112	1148.6077	0.0035	0	64	0.00031	1	U	K.LVNEVTEFAK.T
184	575.3133	1148.6121	1148.6077	0.0043	0	(42)	0.05	1	U	K.LVNEVTEFAK.T
265	722.3264	1442.6383	1442.6347	0.0036	0	28	0.29	1	U	K.YICENQDSISSK.L
296	547.3193	1638.9362	1638.9305	0.0057	1	(48)	0.0045	1		K.KVPQVSTPTLVEVSR.N
297	547.3194	1638.9363	1638.9305	0.0059	1	54	0.001	1		K.KVPQVSTPTLVEVSR.N
298	547.3198	1638.9375	1638.9305	0.0070	1	(44)	0.011	1		K.KVPQVSTPTLVEVSR.N

Proteins matching the same set of peptides:
ALBU_PONAB **Mass:** 71317 **Score:** 147 **Matches:** 6(5) **Sequences:** 3(2)
Serum albumin OS=Pongo abelii GN=ALB PE=2 SV=1

11. K2C6B_HUMAN **Mass:** 60315 **Score:** 140 **Matches:** 5(4) **Sequences:** 4(3) **emPAI:** 0.25
Keratin, type II cytoskeletal 6B OS=Homo sapiens GN=KRT6B PE=1 SV=5
Check to include this hit in error tolerant search or archive report

Query	Observed	Mr(expt)	Mr(calc)	Delta	Miss	Score	Expect	Rank	Unique	Peptide
167	554.2769	1106.5392	1106.5356	0.0036	0	38	0.095	1		K.AQYEEIAQR.S
192	590.3040	1178.5935	1178.5931	0.0004	0	54	0.0029	1		K.YEELQITAGR.H
240	665.3693	1328.7241	1328.7187	0.0054	0	(46)	0.015	1		R.NLDLDSIIAEVK.A
241	665.3699	1328.7252	1328.7187	0.0065	0	73	3.3e-005	1		R.NLDLDSIIAEVK.A
259	704.3626	1406.7106	1406.7041	0.0065	0	75	1.9e-005	1	U	K.ADTLTDEINFLR.A

12. F13B_HUMAN **Mass:** 77742 **Score:** 113 **Matches:** 5(3) **Sequences:** 3(2) **emPAI:** 0.14
Coagulation factor XIII B chain OS=Homo sapiens GN=F13B PE=1 SV=3
Check to include this hit in error tolerant search or archive report

Query	Observed	Mr(expt)	Mr(calc)	Delta	Miss	Score	Expect	Rank	Unique	Peptide
177	565.7718	1129.5291	1129.5226	0.0065	0	30	0.36	1	U	R.CNEYYLLR.G
353	695.0044	2081.9914	2081.9840	0.0074	0	(37)	0.061	1	U	K.VACEEPPFIENGAANLHSK.I
354	695.0047	2081.9923	2081.9840	0.0083	0	(51)	0.0023	1	U	K.VACEEPPFIENGAANLHSK.I
355	695.0059	2081.9959	2081.9840	0.0119	0	55	0.00097	1	U	K.VACEEPPFIENGAANLHSK.I
441	899.7396	2696.1969	2696.1807	0.0162	1	38	0.01	1	U	K.TTGGKDEEVVQCLSDGWSSQPTCR.K

13. K1C14_HUMAN **Mass:** 51872 **Score:** 95 **Matches:** 7(4) **Sequences:** 3(2) **emPAI:** 0.14
Keratin, type I cytoskeletal 14 OS=Homo sapiens GN=KRT14 PE=1 SV=4
Check to include this hit in error tolerant search or archive report

Query	Observed	Mr(expt)	Mr(calc)	Delta	Miss	Score	Expect	Rank	Unique	Peptide
98	404.2046	806.3947	806.3923	0.0024	0	(38)	0.15	1		R.LAADDFR.T
99	404.2048	806.3950	806.3923	0.0027	0	(47)	0.016	1		R.LAADDFR.T
100	404.2050	806.3954	806.3923	0.0032	0	62	0.00056	1		R.LAADDFR.T
138	515.3023	1028.5901	1028.5866	0.0035	0	42	0.06	1		R.VLDELTLAR.A
160	545.7700	1089.5254	1089.5237	0.0017	0	(34)	0.27	1		K.VTMQNLNDR.L
161	545.7705	1089.5265	1089.5237	0.0029	0	54	0.0022	1		K.VTMQNLNDR.L
162	545.7707	1089.5268	1089.5237	0.0031	0	(54)	0.0022	1		K.VTMQNLNDR.L

Proteins matching the same set of peptides:
K1C14_MOUSE **Mass:** 53176 **Score:** 95 **Matches:** 7(4) **Sequences:** 3(2)
Keratin, type I cytoskeletal 14 OS=Mus musculus GN=Krt14 PE=1 SV=2
K1C14_RAT **Mass:** 52936 **Score:** 95 **Matches:** 7(4) **Sequences:** 3(2)
Keratin, type I cytoskeletal 14 OS=Rattus norvegicus GN=Krt14 PE=2 SV=1
K1C15_MOUSE **Mass:** 49278 **Score:** 95 **Matches:** 7(4) **Sequences:** 3(2)
Keratin, type I cytoskeletal 15 OS=Mus musculus GN=Krt15 PE=1 SV=2
K1C15_RAT **Mass:** 49011 **Score:** 95 **Matches:** 7(4) **Sequences:** 3(2)
Keratin, type I cytoskeletal 15 OS=Rattus norvegicus GN=Krt15 PE=1 SV=1
K1C16_HUMAN **Mass:** 51578 **Score:** 95 **Matches:** 7(4) **Sequences:** 3(2)
Keratin, type I cytoskeletal 16 OS=Homo sapiens GN=KRT16 PE=1 SV=4

14. F13A_HUMAN **Mass:** 83728 **Score:** 72 **Matches:** 5(2) **Sequences:** 4(2) **emPAI:** 0.13
Coagulation factor XIII A chain OS=Homo sapiens GN=F13A1 PE=1 SV=4
Check to include this hit in error tolerant search or archive report

Query	Observed	Mr(expt)	Mr(calc)	Delta	Miss	Score	Expect	Rank	Unique	Peptide
234	640.8520	1279.6895	1279.6846	0.0050	0	58	0.00088	1		R.LALETALMYGAK.K
239	663.9182	1325.8219	1325.8170	0.0049	0	29	0.18	1	U	K.STVLTIPEIIIK.V
300	827.4443	1652.8740	1652.8661	0.0078	1	49	0.0056	1	U	K.ETFDVTLEPLSFKK.E
308	844.4697	1686.9249	1686.9192	0.0056	0	30	0.48	1	U	K.GTYIPVPIVSELQSGK.W
309	844.4699	1686.9252	1686.9192	0.0060	0	(25)	1.5	1	U	K.GTYIPVPIVSELQSGK.W

15. CH602_BURXL **Mass:** 56983 **Score:** 21 **Matches:** 6(0) **Sequences:** 1(0) **emPAI:** 0.06
60 kDa chaperonin 2 OS=Burkholderia xenovorans (strain LB400) GN=groL2 PE=3 SV=1
Check to include this hit in error tolerant search or archive report

Query	Observed	Mr(expt)	Mr(calc)	Delta	Miss	Score	Expect	Rank	Unique	Peptide

453	913.8394	2738.4963	2738.4888	0.0075	0	28	0.22	3	U	K.AGKPLLIIAEDVEGEALTTLVVNSMR.G
454	685.6324	2738.5005	2738.4888	0.0117	0	(26)	0.38	3	U	K.AGKPLLIIAEDVEGEALTTLVVNSMR.G
460	919.1723	2754.4950	2754.4837	0.0113	0	(11)	13	1	U	K.AGKPLLIIAEDVEGEALTTLVVNSMR.G + Oxidation (M)
461	919.1726	2754.4960	2754.4837	0.0123	0	(21)	1.4	1	U	K.AGKPLLIIAEDVEGEALTTLVVNSMR.G + Oxidation (M)
462	919.1728	2754.4966	2754.4837	0.0129	0	(16)	3.8	1	U	K.AGKPLLIIAEDVEGEALTTLVVNSMR.G + Oxidation (M)
463	919.1730	2754.4970	2754.4837	0.0133	0	(8)	24	1	U	K.AGKPLLIIAEDVEGEALTTLVVNSMR.G + Oxidation (M)

Peptide matches not assigned to protein hits: (no details means no match)

Query	Observed	Mr(expt)	Mr(calc)	Delta	Miss	Score	Expect	Rank	Unique	Peptide
105	416.7490	831.4835	831.4814	0.0021	0	32	0.66	1		SLSLGSLR
129	463.2789	924.5433	924.5579	-0.0146	1	31	0.32	1		KMHGLLVK
407	414.7330	2482.3546	2482.3656	-0.0109	2	29	0.26	1		SERPALVRQSTFIKEAPSPTLR
128	463.2789	924.5432	924.5644	-0.0212	0	21	2.7	1		NPLLELVK
292	540.9388	1619.7947	1619.8314	-0.0368	1	14	18	1		RMGDNPHANGGILLR
113	432.2728	862.5311	862.5025	0.0287	2	14	15	1		KFVADRK
111	846.4443	845.4370	845.4495	-0.0124	0	13	56	1		DTVVEVGK
109	846.4421	845.4349	845.4607	-0.0258	1	12	66	1		DEKSVIR
110	846.4442	845.4369	845.4495	-0.0125	0	12	78	1		DTVVEVGK
584	4876.5843	4875.5770	4875.5963	-0.0193	2	11	1.9	1		MNALNEPQVVRALYVASQAGVQIDLIVRGACTLRPGVQGISENIR
616	942.5184	7532.0886	7532.1216	-0.0330	2	11	0.28	1		SKQRTMTFRPLSQLLFWTLTANLLILTWVGSQPVEHPFMIIGQLASLTYFTILLVLFPITGALENK + Oxidation (M)
97	802.4321	801.4248	801.3981	0.0268	1	10	1.4e+002	1		VRDGELN
344	678.3584	2032.0534	2032.0598	-0.0063	2	10	50	1		LSHKEVMQKMGELFALR + Oxidation (M)
320	883.8986	1765.7826	1765.8226	-0.0400	1	9	19	1		KMEQESIDQLCELK + Oxidation (M)
385	762.0638	2283.1695	2283.1470	0.0226	0	9	52	1		LQAIEPSGNYHILHAYCGIK
524	530.7850	3178.6666	3178.6339	0.0327	1	7	37	1		GFFGEHGGLFVSETLIPALQELADAYKAAK
472	695.1195	2776.4488	2776.4872	-0.0384	1	6	66	1		AYLTSRAAQQVNIEVLVTIDGVNFR
569	671.3476	4022.0421	4022.0782	-0.0361	2	6	39	1		KGVDSLDDLQNVALMVHYLNNDDPQIQIEILRSLK + Oxidation (M)
446	906.7579	2717.2518	2717.2975	-0.0457	1	5	42	1		EVVEAMTASCKHPMIFPLSNPTSR + Oxidation (M)
513	517.2625	3097.5312	3097.5729	-0.0417	2	5	76	1		VSEMQDRHNAKLITAFIPLSSMFGYVK + Oxidation (M)
397	800.4278	2398.2617	2398.2413	0.0203	1	5	1.2e+002	1		VVGIKNGEIIDMDIDEALAVER
471	695.1186	2776.4453	2776.4290	0.0164	1	4	1.1e+002	1		AGAHVTVVAPRLCSELLEQLNEQSR
468	694.8620	2775.4191	2775.3763	0.0428	1	4	1.2e+002	1		RNQSFCPTVNLDKPWTLVSEQTR
340	646.3287	1935.9644	1935.9845	-0.0202	0	4	1.9e+002	1		ARPFLGICVGMQLMATR + Oxidation (M)
339	646.3287	1935.9644	1935.9169	0.0474	0	3	2.2e+002	1		IVGDDVAIMMVEAEATEK + Oxidation (M)
321	883.8988	1765.7830	1765.7842	-0.0012	0	3	74	1		EHLPEMHQGSFADPR + Oxidation (M)
291	539.3375	1614.9906	1614.9457	0.0449	2	3	25	1		VAIVGPNGSGKKTFIK
586	623.3107	4978.4278	4978.4573	-0.0295	2	3	37	1		SVSVYESFAWFYAVNTGQISIRHGMRGPSSALVAEQAGGLDALGHAR + Oxidation (M)
555	430.2458	3433.9083	3433.8690	0.0393	1	3	24	1		DQKALSVLSPSLNLVVDYGFLWWLAVPIYK
444	903.4259	2707.2559	2707.2500	0.0059	1	3	83	1		FEPPEPSATLNDIKVYTEEEDER
335	630.9678	1889.8816	1889.8499	0.0317	0	2	1.5e+002	1		MSANHMTPGEIVSELDK + 2 Oxidation (M)
403	802.4263	2404.2572	2404.2823	-0.0251	1	2	2.1e+002	1		TAAEKGGVETLGALVHNQQVLNR
503	1435.1738	2868.3331	2868.3699	-0.0368	1	2	97	1		EGVITVEEAKGTETTVEVVEGMQFDR + Oxidation (M)
566	651.3317	3901.9467	3901.9197	0.0271	1	2	1e+002	1		LDGPDSTAVLNTVIFNSFVFCQVFNEINSREIEK
470	694.8625	2775.4210	2775.4048	0.0162	1	2	2e+002	1		SAASDVGRDPMAILPAVQMFVVTGSTR
600	5889.0134	5888.0061	5887.9805	0.0256	2	2	19	1		EFLSDYSQVNCEVLTPHLFDFRQEEAVLHLMKVSAGLDSLVLGEGQILSQVK
451	912.0901	2733.2484	2733.2792	-0.0307	2	2	80	1		SMIISWQEMDRRQFNGPGFQYK + Oxidation (M)
590	887.9477	5321.6425	5321.6564	-0.0139	1	2	40	1		SVPGTIVRHSAYIAPNVILMPSFVNLGAFIDEGTMVDTWATVGSCAQIGK + 2 Oxidation (M)
384	762.0633	2283.1682	2283.1470	0.0212	0	2	2.6e+002	1		LQAIEPSGNYHILHAYCGIK
572	600.3382	4195.3162	4195.3093	0.0069	2	1	12	1		LMRWGASDVHFVRPVHTVTLLLGDKVIPATILGIPSDR + Oxidation (M)
512	500.8024	2998.7705	2998.7477	0.0228	2	1	7.7	1		MTIRLLPPVLVNRIAAGEVVERPASAVK
326	611.6519	1831.9340	1831.9275	0.0065	2	1	3.7e+002	1		KNDKEGSQSASLQEALK
578	763.8837	4577.2586	4577.2425	0.0161	0	1	67	1		LFSEDQGLIISVMTNPTTGGVSASFASLGDYNFAEPGALIGFAGR
180	569.3348	1136.6550	1136.6190	0.0360	1	1	2.9e+002	1		VYDIKLSSGR
485	942.4814	2824.4223	2824.4369	-0.0146	1	1	2.2e+002	1		AAGRTDAGVHASAQVAHVDLAKPWPGDK
80	622.3659	621.3587	621.3235	0.0352	0	1	6.7e+002	1		FGGSVR
130	476.3069	950.5993	950.5522	0.0471	2	1	1.2e+002	1		HQKVRQR
367	539.3069	2153.1985	2153.1965	0.0019	0	1	1.6e+002	1		APPSLVGLILHVALCMGLHR
473	698.8611	2791.4155	2791.3896	0.0259	2	1	2.5e+002	1		QPHRPIERADAMDTSDRPGLRTTR + Oxidation (M)
349	690.6832	2069.0278	2069.0099	0.0179	2	1	3.3e+002	1		KALTETNGDMDKAIDYLR + Oxidation (M)
556	889.4480	3553.7630	3553.7810	-0.0180	1	1	1.7e+002	1		VQEGTYMGHIVEHIALELQELAGMPVGFGRTR + Oxidation (M)
478	698.8616	2791.4174	2791.4273	-0.0099	1	1	2.6e+002	1		DLSTIVTRSAQVMGLAIDSEGATEIAK + Oxidation (M)
570	679.3641	4070.1409	4070.1007	0.0401	2	1	67	1		FIVADTPGHEQYTRNMATGASTADLAIVLIDARQGVLR
539	655.3085	3271.5063	3271.5066	-0.0003	1	0	89	1		LLFTYIMNMESQPYGNPHYERSPSPER + Oxidation (M)
368	719.6879	2156.0420	2156.0597	-0.0177	0	0	3.1e+002	1		APVSPALASPSSEVSDVSEATR
466	922.8450	2765.5130	2765.5489	-0.0359	1	0	1.4e+002	1		MIWIMLATLAVVFVVGFRVLTSGAR + Oxidation (M)
541	823.9199	3291.6505	3291.6664	-0.0159	2	0	2.1e+002	1		MTIMSSLAGAGRGVVNTIGGAAQGINSVKSSADR + Oxidation (M)
1	*402.3567*	*401.3494*								
2	*415.2124*	*414.2051*								
3	*415.2129*	*414.2056*								
4	*415.2131*	*414.2058*								
5	*415.2554*	*414.2481*								
6	*415.3426*	*414.3353*								
7	*415.3432*	*414.3359*								
8	*428.3746*	*427.3674*								
9	*430.2446*	*429.2373*								
10	*430.2453*	*429.2380*								
11	*430.2497*	*429.2424*								
12	*430.3901*	*429.3828*								
13	*432.2388*	*431.2315*								
14	*432.2388*	*431.2315*								
15	*432.2397*	*431.2324*								
16	*432.2812*	*431.2740*								
17	*432.2813*	*431.2741*								
18	*432.2817*	*431.2744*								
19	*435.2724*	*434.2652*								
20	*442.3335*	*441.3262*								
21	*444.3476*	*443.3404*								
22	*444.3483*	*443.3410*								
23	*445.1195*	*444.1122*								
24	*453.3444*	*452.3371*								
25	*455.2255*	*454.2182*								
26	*456.2812*	*455.2739*								
27	*459.2816*	*458.2743*								
28	*459.2818*	*458.2746*								
29	*461.3287*	*460.3214*								
30	*476.3078*	*475.3005*								
31	*476.3084*	*475.3011*								
32	*478.9798*	*477.9725*								
33	*500.3736*	*499.3663*								
34	*503.3075*	*502.3002*								
35	*520.3340*	*519.3268*								
36	*520.3345*	*519.3272*								
37	*525.3215*	*524.3142*								
38	*528.3651*	*527.3578*								
39	*528.3657*	*527.3585*								
40	*531.2804*	*530.2731*								
41	*535.3164*	*534.3091*								
42	*539.3369*	*538.3296*								
43	*540.2980*	*539.2908*								
44	*547.3344*	*546.3271*								
45	*551.5039*	*550.4966*								
46	*551.5040*	*550.4967*								
47	*551.5041*	*550.4968*								
48	*551.5042*	*550.4970*								
49	*551.5042*	*550.4970*								
50	*551.5044*	*550.4971*								
51	*551.5045*	*550.4972*								
52	*551.5045*	*550.4972*								

53	551.5046	550.4973
54	551.5046	550.4973
55	551.5047	550.4974
56	551.5047	550.4974
57	551.5048	550.4975
58	551.5051	550.4978
59	551.5051	550.4979
60	564.3607	563.3535
61	564.3610	563.3537
62	579.5360	578.5287
63	579.5361	578.5288
64	579.5364	578.5291
65	579.5370	578.5297
66	591.3591	590.3518
67	591.5721	590.5649
70	608.3872	607.3799
71	608.3875	607.3802
72	614.5658	613.5586
73	615.4045	614.3972
74	615.4049	614.3976
75	615.4052	614.3979
76	615.4052	614.3979
77	617.4076	616.4003
78	617.4088	616.4015
79	620.3647	619.3575
81	630.4436	629.4363
83	674.4719	673.4646
84	675.6719	674.6647
85	675.6763	674.6690
86	675.6763	674.6691
87	675.6780	674.6707
88	675.6781	674.6709
89	675.6805	674.6732
90	677.6837	676.6765
91	696.4401	695.4328
92	718.4972	717.4899
94	752.5480	751.5408
95	752.5483	751.5410
106	832.4908	831.4835
120	444.1146	886.2147
121	444.1148	886.2150
122	447.1334	892.2522
123	918.6715	917.6643
124	461.3302	920.6458
131	969.9088	968.9015
140	1037.2643	1036.2570
141	1037.2652	1036.2579
	523.2864	1044.5583
	523.7453	1045.4760
	523.7453	1045.4761
146	1046.5932	1045.5859
	550.2257	1098.4369
	578.7679	1155.5212
	582.8355	1163.6563
	591.3486	1180.6826
	591.3497	1180.6849
	596.2141	1190.4137
	608.2571	1214.4996
	608.2579	1214.5012
	436.8914	1307.6525
	442.5349	1324.5829
	678.8228	1355.6311
	405.9633	1619.8239
	550.3360	1647.9860
	552.5381	1654.5925
	552.5381	1654.5925
	553.2606	1656.7599
	574.8157	1721.4252
	934.9224	1867.8302
	636.8432	1907.5078
	647.4261	1939.2564
	415.3390	2071.6587
	728.6481	2182.9225
	444.1117	2215.5222
	558.9748	2231.8699
	755.3486	2263.0239
380	1142.0996	2282.1847
383	1142.1005	2282.1865
	779.3736	2335.0991
	779.3741	2335.1006
	610.7860	2439.1147
	818.0726	2451.1961
	503.3067	2511.4970
	632.3882	2525.5236
	858.9123	2573.7152
	647.4276	2585.6815
	652.4100	2605.6108
429	1333.1750	2664.3354
439	1343.1719	2684.3292
	895.7837	2684.3292
	906.7563	2717.2471
	913.7563	2738.2470
	913.9993	2738.9760
	914.7524	2741.2353
	691.1200	2760.4511
	922.8448	2765.5125
	926.1470	2775.4191
	926.1476	2775.4210
	931.4791	2791.4155
	931.4792	2791.4159
	698.8613	2791.4159
	931.4797	2791.4174
	949.4558	2845.3457
	712.3437	2845.3457
497	408.7863	2854.4534

499	1434.1728	2866.3311
504	412.1694	2878.1349
505	1442.1693	2882.3240
506	968.7578	2903.2517
507	968.7580	2903.2521
508	974.0881	2919.2425
509	974.0893	2919.2461
510	730.8188	2919.2461
511	974.0895	2919.2466
514	1037.2703	3108.7889
518	520.3340	3115.9602
519	1054.5086	3160.5039
522	1055.5244	3163.5513
523	791.8951	3163.5513
526	1079.8515	3236.5328
527	1079.8532	3236.5378
528	1079.8542	3236.5407
529	1079.8542	3236.5408
533	1080.5862	3238.7368
535	652.4031	3256.9789
540	823.9199	3291.6503
542	1103.9197	3308.7373
544	1103.9201	3308.7384
547	830.1166	3316.4373
550	673.3377	3361.6523
551	841.4209	3361.6543
552	842.1644	3364.6285
553	842.1646	3364.6294
554	484.9330	3387.4799
557	447.2910	3570.2699
558	1192.3861	3574.1366
560	904.1412	3612.5357
561	607.9010	3641.3625
562	523.8003	3659.5515
563	528.3601	3691.4700
564	934.8793	3735.4879
565	551.5029	3853.4692
567	500.3081	3994.4068
568	802.9248	4009.5876
571	4117.4670	4116.4597
573	608.3862	4251.6526
574	551.5055	4403.9862
575	630.4464	4406.0739
576	651.8589	4555.9613
577	652.4142	4559.8487
579	4628.6665	4627.6592
580	582.8301	4654.5824
581	594.3630	4746.8456
582	800.4289	4796.5296
583	4859.4724	4858.4651
585	4970.9711	4969.9638
587	5018.5784	5017.5711
588	5230.6058	5229.5985
589	5318.0365	5317.0292
591	5345.0781	5344.0708
592	5399.4136	5398.4063
593	1100.9730	5499.8288
594	5567.0896	5566.0823
595	697.8462	5574.7116
596	5693.8017	5692.7944
597	5724.3392	5723.3319
598	716.9174	5727.2812
599	718.4972	5739.9193
601	5966.2178	5965.2105
602	5993.2904	5992.2831
603	1011.0022	6059.9694
604	6439.5208	6438.5135
605	6464.1858	6463.1785
606	924.9322	6467.4747
607	6492.0414	6491.0341
608	942.5341	6590.6878
609	6734.6303	6733.6230
610	857.4237	6851.3311
611	6919.3963	6918.3890
612	6954.3064	6953.2991
613	7010.1415	7009.1342
614	883.8968	7063.1158
615	914.0821	7304.5987
617	7790.3373	7789.3300
618	7951.0228	7950.0155
619	1326.1777	7951.0228
620	8232.6010	8231.5937
621	8391.5766	8390.5693
622	9756.3243	9755.3170

Search Parameters

```
Type of search           :MS/MS Ion  Search
Enzyme                   :Trypsin
Fixed modifications      :Carbamidomethyl (C)
Variable modifications   :Oxidation  (M)
Mass values              :Monoisotopic
Protein Mass             :Unrestricted
Peptide Mass Tolerance :  ± 0.05 Da
Fragment Mass Tolerance:  ± 0.05 Da
Max Missed Cleavages     :2
Instrument type          :Default
Number of queries        :622
```

Mascot Search Results

**User**	**: Julian**
**Email**	**: julian.langer@mpibp-frankfurt.mpg.de**
**Search title**	**: Submitted from 140318 842 ChKrettler 1-10 swissprot full T 0.05Da by Mascot Daemon on CCSW010**
**MS data file**	**: D:\Data\1403\140318\140314 842 ChReinhard 2_Tray01-C7_01_17683.d\140314 842 ChReinhard 2_Tray01-C7_01_17683.mgf**
**Database**	**: SwissProt 57.15 (515203 sequences; 181334896 residues)**
**Timestamp**	**: 19 Mar 2014 at 09:48:48 GMT**
**Protein hits**	**:** CFAH_HUMAN Complement factor H OS=Homo sapiens GN=CFH PE=1 SV=4
	K2C1_HUMAN Keratin, type II cytoskeletal 1 OS=Homo sapiens GN=KRT1 PE=1 SV=6
	K1C9_HUMAN Keratin, type I cytoskeletal 9 OS=Homo sapiens GN=KRT9 PE=1 SV=3
	TRYP_PIG Trypsin OS=Sus scrofa PE=1 SV=1
	K1C16_HUMAN Keratin, type I cytoskeletal 16 OS=Homo sapiens GN=KRT16 PE=1 SV=4
	K2C1_RAT Keratin, type II cytoskeletal 1 OS=Rattus norvegicus GN=Krt1 PE=2 SV=1
	K1C10_HUMAN Keratin, type I cytoskeletal 10 OS=Homo sapiens GN=KRT10 PE=1 SV=6
	K1C14_HUMAN Keratin, type I cytoskeletal 14 OS=Homo sapiens GN=KRT14 PE=1 SV=4
	CH60_CITK8 60 kDa chaperonin OS=Citrobacter koseri (strain ATCC BAA-895 / CDC 4225-83 / SGSC4696) GN=groL PE=3 SV=1
	K2C6B_HUMAN Keratin, type II cytoskeletal 6B OS=Homo sapiens GN=KRT6B PE=1 SV=5
	K2C5_RAT Keratin, type II cytoskeletal 5 OS=Rattus norvegicus GN=Krt5 PE=1 SV=1
	K2C6C_HUMAN Keratin, type II cytoskeletal 6C OS=Homo sapiens GN=KRT6C PE=1 SV=3
	K2C5_HUMAN Keratin, type II cytoskeletal 5 OS=Homo sapiens GN=KRT5 PE=1 SV=3
	K2C6A_HUMAN Keratin, type II cytoskeletal 6A OS=Homo sapiens GN=KRT6A PE=1 SV=3
	K22E_HUMAN Keratin, type II cytoskeletal 2 epidermal OS=Homo sapiens GN=KRT2 PE=1 SV=2
	K1C17_HUMAN Keratin, type I cytoskeletal 17 OS=Homo sapiens GN=KRT17 PE=1 SV=2
	HORN_HUMAN Hornerin OS=Homo sapiens GN=HRNR PE=1 SV=2
	ALBU_HUMAN Serum albumin OS=Homo sapiens GN=ALB PE=1 SV=2
	K2C4_HUMAN Keratin, type II cytoskeletal 4 OS=Homo sapiens GN=KRT4 PE=1 SV=4
	A1M_RAT Alpha-1-macroglobulin OS=Rattus norvegicus GN=A1m PE=1 SV=1
	A2MG_HUMAN Alpha-2-macroglobulin OS=Homo sapiens GN=A2M PE=1 SV=2

Mascot Score Histogram

Ions score is -10*Log(P), where P is the probability that the observed match is a random event.
Individual ions scores > 38 indicate identity or extensive homology (p<0.05).
Protein scores are derived from ions scores as a non-probabilistic basis for ranking protein hits.

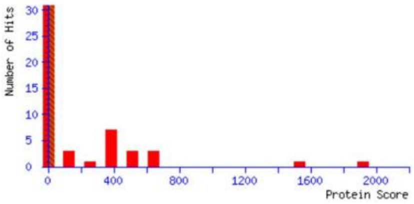

Peptide Summary Report

Format As Peptide Summary | Help

Significance threshold p< 0.05 | Max. number of hits AUTO

Standard scoring | MudPIT scoring | Ions score or expect cut-off 0 | Show sub-sets 0

Show pop-ups | Suppress pop-ups | Sort unassigned Decreasing Score | Require bold red

Select All | Select None | Search Selected | **Error tolerant** | Archive Report

emPAI: 1.

1. CFAH_HUMAN **Mass:** 143680 **Score:** 1917 **Matches:** 84(58) **Sequences:** 42(34) 89

Complement factor H OS=Homo sapiens GN=CFH PE=1 SV=4

Check to include this hit in error tolerant search or archive report

Query	Observed	Mr(expt)	Mr(calc)	Delta	Miss	Score	Expect	Rank	Unique	Peptide
97	463.2317	924.4488	924.4454	0.0034	0	55	0.0015	1	U	R.NGFYPATR.G
98	463.2319	924.4493	924.4454	0.0040	0	(52)	0.0033	1	U	R.NGFYPATR.G
99	463.2320	924.4494	924.4454	0.0040	0	(43)	0.022	1	U	R.NGFYPATR.G
100	463.2324	924.4502	924.4454	0.0049	0	(44)	0.02	1	U	R.NGFYPATR.G
109	487.7253	973.4360	973.4328	0.0033	0	(30)	0.32	1	U	R.FVCNSGYK.I
110	487.7253	973.4360	973.4328	0.0033	0	60	0.00032	1	U	R.FVCNSGYK.I
111	487.7253	973.4360	973.4328	0.0033	0	(45)	0.0084	1	U	R.FVCNSGYK.I
132	521.7529	1041.4912	1041.4879	0.0033	0	(24)	1.3	1	U	R.NGQWSEPPK.C
133	521.7535	1041.4924	1041.4879	0.0044	0	31	0.25	1	U	R.NGQWSEPPK.C
134	521.7536	1041.4925	1041.4879	0.0046	0	(27)	0.64	1	U	R.NGQWSEPPK.C
143	532.3006	1062.5867	1062.5822	0.0045	0	(57)	0.0013	1	U	K.IDVHLVPDR.K
144	532.3009	1062.5873	1062.5822	0.0051	0	61	0.00062	1	U	K.IDVHLVPDR.K
145	532.7920	1063.5694	1063.5662	0.0032	1	(42)	0.042	1	U	K.IIYKENER.F
146	532.7921	1063.5696	1063.5662	0.0035	1	43	0.039	1	U	K.IIYKENER.F
165	552.7755	1103.5364	1103.5281	0.0084	0	50	0.0055	1	U	K.IVSSAMEPDR.E
166	552.7755	1103.5365	1103.5281	0.0085	0	(43)	0.027	1	U	K.IVSSAMEPDR.E
171	556.2729	1110.5313	1110.5280	0.0032	0	18	6.3	1	U	K.WSHPPSCIK.T
176	560.7701	1119.5257	1119.5230	0.0027	0	(17)	11	1	U	K.IVSSAMEPDR.E + Oxidation (M)
181	566.2641	1130.5135	1130.5105	0.0031	0	(29)	0.36	1	U	K.EFDHNSNIR.Y
182	566.2641	1130.5136	1130.5105	0.0032	0	(44)	0.011	1	U	K.EFDHNSNIR.Y
183	566.2647	1130.5148	1130.5105	0.0043	0	53	0.0014	1	U	K.EFDHNSNIR.Y
184	567.3294	1132.6443	1132.6393	0.0050	0	55	0.002	1		R.RPYFPVAVGK.Y
187	571.2852	1140.5558	1140.5532	0.0026	0	38	0.071	1	U	K.CLHPCVISR.E
188	574.8016	1147.5885	1147.5842	0.0044	0	45	0.028	1	U	R.SLGNVIMVCR.K
198	593.3027	1184.5908	1184.5860	0.0048	0	(58)	0.00085	1	U	K.CLPVTAPENGK.I
199	593.3028	1184.5911	1184.5860	0.0052	0	71	4.1e-005	1	U	K.CLPVTAPENGK.I
200	593.8014	1185.5883	1185.5819	0.0064	1	59	0.00062	1	U	R.TKNDFTWFK.L
201	595.2712	1188.5278	1188.5234	0.0045	0	(46)	0.0055	1	U	K.LSYTCEGGFR.I
202	595.2716	1188.5287	1188.5234	0.0054	0	47	0.0047	1	U	K.LSYTCEGGFR.I
215	405.1903	1212.5490	1212.5458	0.0032	0	47	0.0047	1	U	K.HGGLYHENMR.R
216	405.1903	1212.5492	1212.5458	0.0034	0	(40)	0.025	1	U	K.HGGLYHENMR.R
224	621.7758	1241.5370	1241.5347	0.0024	0	73	8e-006	1	U	R.TGDEITYQCR.N
225	621.7767	1241.5387	1241.5347	0.0041	0	(55)	0.00054	1	U	R.TGDEITYQCR.N
227	623.3083	1244.6021	1244.5972	0.0049	0	70	5.5e-005	1	U	K.CTSTGWIPAPR.C
229	630.3285	1258.6424	1258.6380	0.0044	0	37	0.14	1	U	R.WQSIPLCVEK.I
230	630.3295	1258.6444	1258.6380	0.0064	0	(27)	1.5	1	U	R.WQSIPLCVEK.I
234	631.7976	1261.5806	1261.5761	0.0045	0	(37)	0.064	1	U	K.DGWSAQPTCIK.S
235	631.7978	1261.5810	1261.5761	0.0049	0	60	0.0003	1	U	K.DGWSAQPTCIK.S
243	641.8699	1281.7252	1281.7193	0.0058	1	50	0.0036	1	U	R.KGEWVALNPLR.K
257	655.3072	1308.5999	1308.5955	0.0044	0	(34)	0.13	1	U	K.SCDIPVFMNAR.T
258	655.3079	1308.6013	1308.5955	0.0058	0	(36)	0.087	1	U	K.SCDIPVFMNAR.T
260	663.3035	1324.5924	1324.5904	0.0020	0	(28)	0.39	1	U	K.SCDIPVFMNAR.T + Oxidation (M)
261	663.3044	1324.5942	1324.5904	0.0038	0	37	0.045	1	U	K.SCDIPVFMNAR.T + Oxidation (M)
272	671.3542	1340.6939	1340.6936	0.0003	0	53	0.0025	1	U	K.SPDVINGSPISQK.I
284	683.3478	1364.6811	1364.6758	0.0053	0	(59)	0.00068	1	U	R.EIMENYNIALR.W
285	683.3479	1364.6813	1364.6758	0.0055	0	(63)	0.0003	1	U	R.EIMENYNIALR.W
286	683.3485	1364.6825	1364.6758	0.0067	0	(63)	0.00029	1	U	R.EIMENYNIALR.W
288	691.3457	1380.6768	1380.6707	0.0061	0	68	7.5e-005	1	U	R.EIMENYNIALR.W + Oxidation (M)
297	465.9347	1394.7823	1394.7769	0.0054	0	(29)	0.5	1	U	K.SSNLIILEEHLK.N
298	698.3985	1394.7823	1394.7769	0.0055	0	(45)	0.014	1	U	K.SSNLIILEEHLK.N
299	698.3999	1394.7852	1394.7769	0.0083	0	59	0.00044	1	U	K.SSNLIILEEHLK.N

311	481.2407	1440.7003	1440.6932	0.0070	0	38	0.06	1	U	K.EGWIHTVCINGR.W
318	741.3608	1480.7070	1480.7021	0.0050	0	70	4.6e-005	1	U	K.CFEGFGIDGPAIAK.C
323	503.9096	1508.7070	1508.7003	0.0066	0	46	0.0091	1	U	R.CTLKPCDYPDIK.H
324	755.3608	1508.7070	1508.7003	0.0067	0	(19)	5	1	U	R.CTLKPCDYPDIK.H
326	764.3850	1526.7554	1526.7551	0.0003	0	60	0.00043	1	U	K.SIDVACHPGYALPK.A
340	827.8777	1653.7409	1653.7345	0.0064	0	68	3.3e-005	1	U	K.AGEQVTYTCATYYK.M
368	934.4229	1866.8313	1866.8206	0.0106	0	68	2.2e-005	1	U	K.SCDNPYIPNGDYSPLR.I
377	983.4913	1964.9680	1964.9626	0.0055	0	80	4.2e-006	1	U	K.EQVQSCGPPPELLNGNVK.E
378	983.4931	1964.9717	1964.9626	0.0091	0	(77)	8e-006	1	U	K.EQVQSCGPPPELLNGNVK.E
382	1004.9756	2007.9367	2007.9281	0.0086	0	64	9.5e-005	1	U	K.TDCLSLPSFENAIPMGEK.K
383	1004.9758	2007.9371	2007.9281	0.0090	0	(46)	0.0064	1	U	K.TDCLSLPSFENAIPMGEK.K
384	1010.9965	2019.9784	2019.9684	0.0100	0	(80)	4e-006	1	U	R.DTSCVNPPTVQNAYIVSR.Q
385	1010.9969	2019.9792	2019.9684	0.0108	0	94	1.3e-007	1	U	R.DTSCVNPPTVQNAYIVSR.Q
386	1012.9711	2023.9276	2023.9231	0.0045	0	(48)	0.0035	1	U	K.TDCLSLPSFENAIPMGEK.K + Oxidation (M)
387	1012.9728	2023.9310	2023.9231	0.0079	0	(30)	0.2	1	U	K.TDCLSLPSFENAIPMGEK.K + Oxidation (M)
400	1075.9774	2149.9402	2149.9296	0.0105	0	(56)	0.0002	1	U	R.ECDTDGWTNDIPICEVVK.C
401	1075.9876	2149.9607	2149.9296	0.0311	0	66	2.8e-005	1	U	R.ECDTDGWTNDIPICEVVK.C
408	726.6604	2176.9593	2176.9518	0.0076	0	26	0.26	1	U	R.GDAVCTESGWRPLPSCEEK.S
410	731.3595	2191.0567	2191.0480	0.0087	1	(34)	0.13	1	U	K.IVSSAMEPDREYHFGQAVR.F
411	731.3596	2191.0571	2191.0480	0.0090	1	(60)	0.00032	1	U	K.IVSSAMEPDREYHFGQAVR.F
412	552.7706	2207.0533	2207.0429	0.0103	1	64	0.00012	1	U	K.IVSSAMEPDREYHFGQAVR.F + Oxidation (M)
413	736.6920	2207.0543	2207.0429	0.0114	1	(6)	66	3	U	K.IVSSAMEPDREYHFGQAVR.F + Oxidation (M)
414	552.7709	2207.0543	2207.0429	0.0114	1	(33)	0.15	1	U	K.IVSSAMEPDREYHFGQAVR.F + Oxidation (M)
424	754.0081	2259.0026	2258.9902	0.0124	1	84	3.8e-007	1	U	K.TKEEYGHSEVVEYYCNPR.F
427	1133.0532	2264.0917	2264.0848	0.0069	0	(47)	0.0057	1	U	K.SSIDIENGFISESQYTYALK.E
428	1133.0552	2264.0958	2264.0848	0.0109	0	85	9.7e-007	1	U	K.SSIDIENGFISESQYTYALK.E
452	1206.5789	2411.1432	2411.1348	0.0084	0	99	3.3e-008	1	U	K.VSVLCQENYLIQEGEEITCK.D
453	1206.5868	2411.1590	2411.1348	0.0242	0	(12)	16	1	U	K.VSVLCQENYLIQEGEEITCK.D
470	1301.6241	2601.2336	2601.2235	0.0102	0	22	1.4	1	U	R.NTEILTGSWSDQTYPEGTQAIYK.C
488	914.1057	2739.2952	2739.2844	0.0108	1	(50)	0.002	1	U	K.VSVLCQENYLIQEGEEITCKDGR.W
489	914.1059	2739.2957	2739.2844	0.0114	1	(21)	1.6	1	U	K.VSVLCQENYLIQEGEEITCKDGR.W
490	914.1068	2739.2985	2739.2844	0.0141	1	62	0.00013	1	U	K.VSVLCQENYLIQEGEEITCKDGR.W
505	728.3405	2909.3331	2909.3178	0.0153	0	19	1.4	1	U	K.SPPEISHGVVAHMSDSYQYGEEVTYK.C

2. K2C1_HUMAN **Mass:** 66170 **Score:** 1587 **Matches:** 54(42) **Sequences:** 26(21) **emPAI:** 2.57

Keratin, type II cytoskeletal 1 OS=Homo sapiens GN=KRT1 PE=1 SV=6

Check to include this hit in error tolerant search or archive report

Query	Observed	Mr(expt)	Mr(calc)	Delta	Miss	Score	Expect	Rank	Unique	Peptide
80	416.7494	831.4843	831.4814	0.0029	0	31	0.7	2	U	K.SISISVAR.G
88	437.7552	873.4958	873.4920	0.0039	0	(48)	0.017	1	U	R.SLVNLGGSK.S
89	437.7553	873.4960	873.4920	0.0040	0	48	0.016	1	U	R.SLVNLGGSK.S
108	487.2717	972.5288	972.5240	0.0049	0	41	0.08	1		K.IEISELNR.V
117	500.2281	998.4416	998.4379	0.0038	0	(42)	0.014	1	U	K.DVDGAYMTK.V
118	500.2310	998.4474	998.4379	0.0095	0	43	0.014	1	U	K.DVDGAYMTK.V
128	517.2636	1032.5126	1032.5087	0.0039	0	73	3.6e-005	1	U	R.TLLEGEESR.M
129	517.2641	1032.5136	1032.5087	0.0049	0	(68)	0.00011	1	U	R.TLLEGEESR.M
151	533.2646	1064.5146	1064.5138	0.0008	0	(49)	0.0076	1		K.AQYEDIAQK.S
152	533.2659	1064.5173	1064.5138	0.0035	0	(32)	0.38	1		K.AQYEDIAQK.S
153	533.2660	1064.5174	1064.5138	0.0036	0	57	0.0013	1		K.AQYEDIAQK.S
154	537.3022	1072.5899	1072.5876	0.0022	1	35	0.31	1		R.LRSEIDNVK.K
155	537.3026	1072.5907	1072.5876	0.0031	1	(27)	2	1		R.LRSEIDNVK.K
178	563.2755	1124.5364	1124.5349	0.0014	0	(36)	0.11	1	U	K.AEAESLYQSK.Y
179	563.2771	1124.5395	1124.5349	0.0046	0	52	0.0029	1	U	K.AEAESLYQSK.Y
196	590.3059	1178.5972	1178.5931	0.0041	0	63	0.00031	1		K.YEELQITAGR.H
208	401.2361	1200.6866	1200.6826	0.0040	2	21	5.2	1		R.LRSEIDNVKK.Q
237	633.3243	1264.6341	1264.6299	0.0042	0	82	4e-006	1		R.TNAENEFVTIK.K
238	633.3247	1264.6349	1264.6299	0.0050	0	(56)	0.0014	1		R.TNAENEFVTIK.K
239	633.3248	1264.6351	1264.6299	0.0052	0	(74)	2.2e-005	1		R.TNAENEFVTIK.K
242	639.3594	1276.7043	1276.7027	0.0016	0	61	0.00046	1	U	K.LALDLEIATYR.T
246	650.7713	1299.5280	1299.5224	0.0056	0	45	0.002	1	U	K.NMQDMVEDYR.N
249	651.8633	1301.7120	1301.7078	0.0042	0	(77)	1.2e-005	1		R.SLDLDSIIAEVK.A
250	651.8635	1301.7125	1301.7078	0.0047	0	97	1.3e-007	1		R.SLDLDSIIAEVK.A
254	436.8911	1307.6515	1307.6469	0.0045	2	63	0.00031	1		R.NKYEDEINKR.T
255	436.8913	1307.6522	1307.6469	0.0053	2	(56)	0.0014	1		R.NKYEDEINKR.T
256	436.8917	1307.6532	1307.6469	0.0063	2	(60)	0.0006	1		R.NKYEDEINKR.T
267	670.8394	1339.6643	1339.6619	0.0024	1	(71)	4.2e-005	1	U	K.SKAEAESLYQSK.Y
268	670.8403	1339.6660	1339.6619	0.0041	1	91	4.3e-007	1	U	K.SKAEAESLYQSK.Y
269	447.5626	1339.6660	1339.6619	0.0041	1	(21)	4.3	1	U	K.SKAEAESLYQSK.Y
270	670.8407	1339.6668	1339.6619	0.0049	1	(79)	6.3e-006	1	U	K.SKAEAESLYQSK.Y
271	670.8411	1339.6675	1339.6619	0.0056	1	(74)	2.1e-005	1	U	K.SKAEAESLYQSK.Y
289	692.3515	1382.6884	1382.6830	0.0054	0	(88)	7.2e-007	1	U	K.SLNNQFASFIDK.V
290	692.3515	1382.6885	1382.6830	0.0055	0	108	7.5e-009	1	U	K.SLNNQFASFIDK.V
292	465.2509	1392.7307	1392.7249	0.0059	1	(61)	0.00051	1		R.TNAENEFVTIKK.D
293	697.3726	1392.7307	1392.7249	0.0059	1	90	6.3e-007	1		R.TNAENEFVTIKK.D
294	697.3730	1392.7314	1392.7249	0.0066	1	(76)	1.6e-005	1		R.TNAENEFVTIKK.D
295	465.2514	1392.7324	1392.7249	0.0075	1	(45)	0.019	1		R.TNAENEFVTIKK.D
296	697.3735	1392.7324	1392.7249	0.0075	1	(78)	8.4e-006	1		R.TNAENEFVTIKK.D
316	738.3987	1474.7829	1474.7780	0.0049	0	113	3.1e-009	1		R.FLEQQNQVLQTK.W
317	738.3998	1474.7850	1474.7780	0.0070	0	(112)	4.1e-009	1		R.FLEQQNQVLQTK.W
335	546.9602	1637.8587	1637.8525	0.0061	1	(57)	0.001	2	U	K.SLNNQFASFIDKVR.F
336	819.9367	1637.8589	1637.8525	0.0064	1	102	2.8e-008	1	U	K.SLNNQFASFIDKVR.F
337	546.9602	1637.8589	1637.8525	0.0064	1	(54)	0.002	1	U	K.SLNNQFASFIDKVR.F
345	858.9322	1715.8499	1715.8438	0.0061	0	(29)	0.67	1	U	K.QISNLQQSISDAEQR.G
346	858.9343	1715.8541	1715.8438	0.0103	0	92	3.5e-007	1	U	K.QISNLQQSISDAEQR.G
409	728.7191	2183.1355	2183.1182	0.0173	2	11	35	1	U	K.NKLNDLEDALQQAKEDLAR.L
436	762.7171	2285.1296	2285.1175	0.0121	1	56	0.00083	1	U	K.AEAESLYQSKYEELQITAGR.H
446	783.7471	2348.2195	2348.2111	0.0084	1	43	0.02	1	U	R.SLDLDSIIAEVKAQYEDIAQK.S
460	834.4266	2500.2580	2500.2445	0.0136	2	(18)	5.8	1	U	K.SKAEAESLYQSKYEELQITAGR.H
461	626.0718	2500.2581	2500.2445	0.0136	2	(51)	0.0029	1	U	K.SKAEAESLYQSKYEELQITAGR.H
462	834.4269	2500.2588	2500.2445	0.0143	2	(34)	0.15	1	U	K.SKAEAESLYQSKYEELQITAGR.H
463	626.0720	2500.2588	2500.2445	0.0143	2	53	0.0016	1	U	K.SKAEAESLYQSKYEELQITAGR.H
510	978.1831	2931.5274	2931.5090	0.0184	1	49	0.0033	1		R.FLEQQNQVLQTKWELLQQVDTSTR.T

3. K1C9_HUMAN **Mass:** 62255 **Score:** 655 **Matches:** 35(24) **Sequences:** 22(17) **emPAI:** 1.95

Keratin, type I cytoskeletal 9 OS=Homo sapiens GN=KRT9 PE=1 SV=3

Check to include this hit in error tolerant search or archive report

Query	Observed	Mr(expt)	Mr(calc)	Delta	Miss	Score	Expect	Rank	Unique	Peptide
73	809.4437	808.4364	808.4330	0.0033	0	(32)	0.46	1		R.LASYLDK.V
74	405.2255	808.4364	808.4330	0.0033	0	41	0.053	1		R.LASYLDK.V
93	449.2122	896.4099	896.4062	0.0037	0	39	0.043	1	U	R.MTLDDFR.I
138	530.7876	1059.5606	1059.5560	0.0046	0	61	0.00065	1	U	K.TLLDIDNTR.M
139	530.7878	1059.5610	1059.5560	0.0050	0	(56)	0.0024	1	U	K.TLLDIDNTR.M
149	533.2552	1064.4959	1064.4920	0.0039	0	(42)	0.025	1	U	K.STMQELNSR.L
150	533.2554	1064.4962	1064.4920	0.0042	0	42	0.025	1	U	K.STMQELNSR.L
156	537.7672	1073.5198	1073.5142	0.0057	0	49	0.0067	1	U	R.QFSSSYLSR.S
177	561.2979	1120.5812	1120.5764	0.0048	0	60	0.00073	1	U	R.QEYEQLIAK.N
191	579.3017	1156.5889	1156.5836	0.0053	0	44	0.023	1	U	R.QGVDADINGLR.Q
204	595.8101	1189.6056	1189.6013	0.0043	0	59	0.00086	1	U	R.QVLDNLTMEK.S
220	616.8051	1231.5956	1231.5906	0.0051	0	90	6.4e-007	1	U	R.SGGGGGGGLGSGGSIR.S
221	616.8051	1231.5957	1231.5906	0.0052	0	(86)	1.4e-006	1	U	R.SGGGGGGGLGSGGSIR.S
222	618.2698	1234.5249	1234.5215	0.0035	0	(60)	0.00012	1	U	R.FSSSSGYGGGSSR.V
223	618.2700	1234.5254	1234.5215	0.0040	0	64	5.5e-005	1	U	R.FSSSSGYGGGSSR.V
251	654.3450	1306.6755	1306.6703	0.0052	1	(45)	0.022	1	U	R.IKFEMEQNLR.Q
252	654.3455	1306.6764	1306.6703	0.0061	1	50	0.0077	1	U	R.IKFEMEQNLR.Q
253	436.5661	1306.6764	1306.6703	0.0061	1	(36)	0.19	1	U	R.IKFEMEQNLR.Q
349	587.6520	1759.9343	1759.9257	0.0086	2	6	1.2e+002	1	U	K.IQDWYDKKGPAAIQK.N
356	896.3705	1790.7265	1790.7205	0.0060	0	35	0.0081	1	U	R.GGSGGSYGGGGSGGGYGGGSGSR.G

Query	Observed	Mr(expt)	Mr(calc)	Delta	Miss	Score	Expect	Rank	Unique	Peptide
361	613.3292	1836.9658	1836.9581	0.0077	0	37	0.091	1	U	R.HGVQELEIELQSQLSK.K
363	926.4716	1850.9286	1850.9196	0.0090	1	(7)	99	2	U	K.TLNDMRQEYEQLIAK.N
364	617.9835	1850.9286	1850.9196	0.0090	1	32	0.27	1	U	K.TLNDMRQEYEQLIAK.N
366	617.9835	1850.9287	1850.9196	0.0091	1	(30)	0.5	1	U	K.TLNDMRQEYEQLIAK.N
437	767.0477	2298.1212	2298.1123	0.0089	1	42	0.021	1	U	K.SDLEMQYETLQEELMALKK.N
438	767.0490	2298.1253	2298.1123	0.0130	1	(31)	0.27	1	U	K.SDLEMQYETLQEELMALKK.N
449	793.0715	2376.1925	2376.1808	0.0117	1	69	4.8e-005	1	U	R.LASYLDKVQALEEANNDLENK.I
464	837.3832	2509.1278	2509.1245	0.0033	0	48	0.0014	1	U	K.EIETYHNLLEGGQEDFESSGAGK.I
503	968.1425	2901.4056	2901.4032	0.0024	1	10	21	1	U	K.NYSPYYNTIDDLKDQIVDLTVGNNK.T
504	968.1470	2901.4191	2901.4032	0.0159	1	(9)	29	1	U	K.NYSPYYNTIDDLKDQIVDLTVGNNK.T
521	1075.1030	3222.2873	3222.2744	0.0129	0	16	0.089	1	U	R.GGSGGSHGGGSGFGGESGGSYGGGEEASGSGGGYGGGSGK.S
526	1088.8484	3263.5234	3263.5066	0.0169	0	(49)	0.0015	1	U	K.DIENQYETQITQIEHEVSSSGQEVQSSAK.E
527	1088.8486	3263.5240	3263.5066	0.0174	0	(60)	0.00014	1	U	K.DIENQYETQITQIEHEVSSSGQEVQSSAK.E
528	1088.8486	3263.5241	3263.5066	0.0175	0	63	6.8e-005	1	U	K.DIENQYETQITQIEHEVSSSGQEVQSSAK.E
538	848.9115	3391.6169	3391.6015	0.0154	1	42	0.01	1	U	R.KDIENQYETQITQIEHEVSSSGQEVQSSAK.E

---

4. TRYP_PIG **Mass:** 25078 **Score:** 617 **Matches:** 18(15) **Sequences:** 7(7) **emPAI:** 2.29

Trypsin OS=Sus scrofa PE=1 SV=1

☐ Check to include this hit in error tolerant search or archive report

Query	Observed	Mr(expt)	Mr(calc)	Delta	Miss	Score	Expect	Rank	Unique	Peptide
83	421.7601	841.5056	841.5022	0.0035	0	54	0.0027	1	U	R.VATVSLPR.S
84	421.7602	841.5058	841.5022	0.0037	0	(54)	0.0028	1	U	R.VATVSLPR.S
135	523.2868	1044.5590	1044.5564	0.0026	0	53	0.0052	1	U	K.LSSPATLNSR.V
136	523.2873	1044.5601	1044.5564	0.0038	0	(52)	0.0054	1	U	K.LSSPATLNSR.V
141	532.2597	1062.5049	1062.5016	0.0034	0	94	2.2e-007	1	U	K.APVLSDSSCK.S
142	532.2598	1062.5050	1062.5016	0.0034	0	(87)	1.1e-006	1	U	K.APVLSDSSCK.S
370	941.9302	1881.8458	1881.8349	0.0109	0	104	6.1e-009	1	U	R.SCAAAGTECLISGWGNTK.S
416	737.7102	2210.1087	2210.0967	0.0120	0	95	1.1e-007	1	U	R.LGEHNIDVLEGNEQFINAAK.I
417	1106.0616	2210.1087	2210.0967	0.0120	0	(42)	0.023	1	U	R.LGEHNIDVLEGNEQFINAAK.I
418	737.7111	2210.1114	2210.0967	0.0147	0	(88)	6.7e-007	1	U	R.LGEHNIDVLEGNEQFINAAK.I
419	1106.0630	2210.1114	2210.0967	0.0147	0	(20)	3.7	1	U	R.LGEHNIDVLEGNEQFINAAK.I
420	737.7120	2210.1141	2210.0967	0.0174	0	(4)	1.7e+002	7	U	R.LGEHNIDVLEGNEQFINAAK.I
430	761.7346	2282.1821	2282.1729	0.0092	0	(69)	4.5e-005	1	U	K.IITHPNFNGNTLDNDIMLIK.L
432	761.7347	2282.1823	2282.1729	0.0094	0	(56)	0.00085	1	U	K.IITHPNFNGNTLDNDIMLIK.L
433	761.7353	2282.1841	2282.1729	0.0112	0	77	7e-006	1	U	K.IITHPNFNGNTLDNDIMLIK.L
439	767.0669	2298.1789	2298.1678	0.0111	0	(50)	0.0035	1	U	K.IITHPNFNGNTLDNDIMLIK.L + Oxidation (M)
531	828.1922	3308.7396	3308.7187	0.0209	1	73	8.6e-006	1	U	K.IITHPNFNGNTLDNDIMLIKLSSPATLNSR.V
536	832.1870	3324.7189	3324.7136	0.0053	1	(10)	17	1	U	K.IITHPNFNGNTLDNDIMLIKLSSPATLNSR.V + Oxidation (M)

---

5. K1C16_HUMAN **Mass:** 51578 **Score:** 605 **Matches:** 29(18) **Sequences:** 17(13) **emPAI:** 1.66

Keratin, type I cytoskeletal 16 OS=Homo sapiens GN=KRT16 PE=1 SV=4

☐ Check to include this hit in error tolerant search or archive report

Query	Observed	Mr(expt)	Mr(calc)	Delta	Miss	Score	Expect	Rank	Unique	Peptide
71	404.2052	806.3958	806.3923	0.0036	0	56	0.0021	1		R.LAADDFR.T
72	404.2052	806.3959	806.3923	0.0036	0	(51)	0.0076	1		R.LAADDFR.T
73	809.4437	808.4364	808.4330	0.0033	0	(32)	0.46	1		R.LASYLDK.V
74	405.2255	808.4364	808.4330	0.0033	0	41	0.053	1		R.LASYLDK.V
112	495.2739	988.5332	988.5301	0.0030	1	(25)	3	1	U	R.SEVTELRR.V
113	495.2739	988.5332	988.5301	0.0030	1	37	0.2	1	U	R.SEVTELRR.V
114	495.2740	988.5334	988.5301	0.0033	1	(21)	8.1	1	U	R.SEVTELRR.V
124	515.3031	1028.5916	1028.5866	0.0050	0	(43)	0.046	1		R.VLDELTLAR.T
125	515.3033	1028.5921	1028.5866	0.0055	0	43	0.046	1		R.VLDELTLAR.T
130	518.7721	1035.5295	1035.5250	0.0046	1	35	0.24	1		K.IRDWYQR.Q
147	532.8106	1063.6066	1063.6026	0.0041	1	(51)	0.0038	1		R.LASYLDKVR.A
148	532.8110	1063.6074	1063.6026	0.0049	1	56	0.0012	1		R.LASYLDKVR.A
159	545.7705	1089.5264	1089.5237	0.0027	0	56	0.0015	1		K.VTMQNLNDR.L
162	548.7706	1095.5266	1095.5237	0.0030	0	(16)	11	1	U	R.DAETWFLSK.T
163	548.7711	1095.5276	1095.5237	0.0039	0	23	2.7	1	U	R.DAETWFLSK.T
231	630.3429	1258.6712	1258.6669	0.0043	1	(31)	0.69	1	U	R.TKYEHELALR.Q
232	420.5643	1258.6712	1258.6669	0.0043	1	66	0.00021	1	U	R.TKYEHELALR.Q
233	630.7911	1259.5677	1259.5630	0.0047	0	46	0.0052	1	U	R.EVFTSSSSSSSR.Q
247	651.3356	1300.6567	1300.6510	0.0057	0	119	8.2e-010	1		R.ALEEANADLEVK.I
248	651.3360	1300.6575	1300.6510	0.0065	0	(118)	8.5e-010	1		R.ALEEANADLEVK.I
263	669.8391	1337.6636	1337.6575	0.0060	0	81	4.6e-006	1	U	R.APSTYGGGLSVSSR.F
278	679.3653	1356.7161	1356.7110	0.0051	1	47	0.014	1	U	R.QTVEADVNGLRR.V
279	453.2460	1356.7161	1356.7110	0.0051	1	(11)	53	1	U	R.QTVEADVNGLRR.V
280	679.3654	1356.7163	1356.7110	0.0053	1	(31)	0.48	1	U	R.QTVEADVNGLRR.V
287	457.2368	1368.6886	1368.6819	0.0067	1	23	2.3	1	U	R.KNHEEEMLALR.G
301	703.3517	1404.6889	1404.6845	0.0045	0	88	8.6e-007	1	U	K.EVASNSELVQSSR.S
397	1044.4914	2086.9682	2086.9590	0.0092	0	51	0.0018	1	U	R.GQTGGDVNVEMDAAPGVDLSR.I
398	707.3573	2119.0500	2119.0393	0.0108	1	95	1.4e-007	1	U	K.TEELNKEVASNSELVQSSR.S
399	707.3579	2119.0518	2119.0393	0.0125	1	(83)	2.1e-006	1	U	K.TEELNKEVASNSELVQSSR.S

---

6. K2C1_RAT **Mass:** 65190 **Score:** 549 **Matches:** 15(13) **Sequences:** 6(5) **emPAI:** 0.36

Keratin, type II cytoskeletal 1 OS=Rattus norvegicus GN=Krt1 PE=2 SV=1

☐ Check to include this hit in error tolerant search or archive report

Query	Observed	Mr(expt)	Mr(calc)	Delta	Miss	Score	Expect	Rank	Unique	Peptide
76	827.4315	826.4242	826.4225	0.0017	0	27	0.97	1		K.FASFIDK.V
77	414.2201	826.4256	826.4225	0.0031	0	(10)	50	1		K.FASFIDK.V
157	541.8055	1081.5965	1081.5920	0.0044	1	(51)	0.0039	1		K.FASFIDKVR.F
158	541.8060	1081.5973	1081.5920	0.0053	1	61	0.00043	1		K.FASFIDKVR.F
237	633.3243	1264.6341	1264.6299	0.0042	0	82	4e-006	1		R.TNAENEFVTIK.K
238	633.3247	1264.6349	1264.6299	0.0050	0	(56)	0.0014	1		R.TNAENEFVTIK.K
239	633.3248	1264.6351	1264.6299	0.0052	0	(74)	2.2e-005	1		R.TNAENEFVTIK.K
292	465.2509	1392.7307	1392.7249	0.0059	1	(61)	0.00051	1		R.TNAENEFVTIKK.D
293	697.3726	1392.7307	1392.7249	0.0059	1	90	6.3e-007	1		R.TNAENEFVTIKK.D
294	697.3730	1392.7314	1392.7249	0.0066	1	(76)	1.6e-005	1		R.TNAENEFVTIKK.D
295	465.2514	1392.7324	1392.7249	0.0075	1	(45)	0.019	1		R.TNAENEFVTIKK.D
296	697.3735	1392.7324	1392.7249	0.0075	1	(78)	8.4e-006	1		R.TNAENEFVTIKK.D
316	738.3987	1474.7829	1474.7780	0.0049	0	113	3.1e-009	1		R.FLEQQNQVLQTK.W
317	738.3998	1474.7850	1474.7780	0.0070	0	(112)	4.1e-009	1		R.FLEQQNQVLQTK.W
510	978.1831	2931.5274	2931.5090	0.0184	1	49	0.0033	1		R.FLEQQNQVLQTKWELLQQVDTSTR.T

---

7. K1C10_HUMAN **Mass:** 59020 **Score:** 517 **Matches:** 26(20) **Sequences:** 15(13) **emPAI:** 1.35

Keratin, type I cytoskeletal 10 OS=Homo sapiens GN=KRT10 PE=1 SV=6

☐ Check to include this hit in error tolerant search or archive report

Query	Observed	Mr(expt)	Mr(calc)	Delta	Miss	Score	Expect	Rank	Unique	Peptide
71	404.2052	806.3958	806.3923	0.0036	0	56	0.0021	1		R.LAADDFR.L
72	404.2052	806.3959	806.3923	0.0036	0	(51)	0.0076	1		R.LAADDFR.L
73	809.4437	808.4364	808.4330	0.0033	0	(32)	0.46	1		R.LASYLDK.V
74	405.2255	808.4364	808.4330	0.0033	0	41	0.053	1		R.LASYLDK.V
116	498.2650	994.5155	994.5123	0.0032	1	21	4.5	1		K.IKEWYEK.H
120	502.2815	1002.5485	1002.5458	0.0027	1	21	7.1	1		K.SEITELRR.N
121	502.2819	1002.5493	1002.5458	0.0036	1	(20)	9.8	2		K.SEITELRR.N
126	516.3048	1030.5949	1030.5910	0.0039	0	(49)	0.0078	1		R.VLDELTLTK.A
127	516.3049	1030.5952	1030.5910	0.0042	0	52	0.0044	1		R.VLDELTLTK.A

147	532.8106	1063.6066	1063.6026	0.0041	1	(51)	0.0038	1		R.LASYLDKVR.A
148	532.8110	1063.6074	1063.6026	0.0049	1	56	0.0012	1		R.LASYLDKVR.A
159	545.7705	1089.5264	1089.5237	0.0027	0	56	0.0015	1		K.VTMQNLNDR.L
169	555.2506	1108.4867	1108.4825	0.0042	0	(41)	0.016	1		K.DAEAWFNEK.S
170	555.2512	1108.4877	1108.4825	0.0052	0	64	9.8e-005	1		K.DAEAWFNEK.S
236	631.8045	1261.5944	1261.5899	0.0046	0	60	0.00044	1	U	R.SLLEGEGSSGGGGR.G
277	679.3650	1356.7155	1356.7110	0.0046	1	(47)	0.014	1		R.QSVEADINGLRR.V
281	679.3655	1356.7164	1356.7110	0.0055	1	67	0.00014	1		R.QSVEADINGLRR.V
282	453.2461	1356.7164	1356.7110	0.0055	1	(23)	3.1	1		R.QSVEADINGLRR.V
291	695.8428	1389.6711	1389.6736	-0.0024	0	63	0.00023	1	U	K.QSLEASLAETEGR.Y
320	747.3738	1492.7330	1492.7270	0.0061	1	(56)	0.0013	1	U	R.SQYEQLAEQNRK.D
321	498.5852	1492.7337	1492.7270	0.0067	1	(46)	0.014	1	U	R.SQYEQLAEQNRK.D
322	747.3741	1492.7337	1492.7270	0.0067	1	57	0.001	1	U	R.SQYEQLAEQNRK.D
344	854.3934	1706.7723	1706.7649	0.0074	0	128	3.1e-011	1	U	K.GSLGGGFSSGGFSGGSFSR.G
357	899.0140	1796.0135	1796.0043	0.0092	0	90	2.7e-007	1	U	R.NVQALEIELQSQLALK.Q
380	998.9932	1995.9719	1995.9636	0.0083	0	(28)	0.58	1	U	K.ELTTEIDNNIEQISSYK.S
381	998.9932	1995.9719	1995.9636	0.0083	0	42	0.024	1	U	K.ELTTEIDNNIEQISSYK.S

8. K1C14_HUMAN **Mass:** 51872 **Score:** 492 **Matches:** 26(15) **Sequences:** 16(10) **emPAI:** 1.82
Keratin, type I cytoskeletal 14 OS=Homo sapiens GN=KRT14 PE=1 SV=4
Check to include this hit in error tolerant search or archive report

Query	Observed	Mr(expt)	Mr(calc)	Delta	Miss	Score	Expect	Rank	Unique	Peptide
71	404.2052	806.3958	806.3923	0.0036	0	56	0.0021	1		R.LAADDFR.T
72	404.2052	806.3959	806.3923	0.0036	0	(51)	0.0076	1		R.LAADDFR.T
73	809.4437	808.4364	808.4330	0.0033	0	(32)	0.46	1		R.LASYLDK.V
74	405.2255	808.4364	808.4330	0.0033	0	41	0.053	1		R.LASYLDK.V
112	495.2739	988.5332	988.5301	0.0030	1	(13)	48	7		K.SEISELRR.T
113	495.2739	988.5332	988.5301	0.0030	1	22	6.4	5		K.SEISELRR.T
114	495.2740	988.5334	988.5301	0.0033	1	(11)	81	9		K.SEISELRR.T
124	515.3031	1028.5916	1028.5866	0.0050	0	(43)	0.046	1		R.VLDELTLAR.A
125	515.3033	1028.5921	1028.5866	0.0055	0	43	0.046	1		R.VLDELTLAR.A
130	518.7721	1035.5295	1035.5250	0.0046	1	35	0.24	1		K.IRDWYQR.Q
147	532.8106	1063.6066	1063.6026	0.0041	1	(51)	0.0038	1		R.LASYLDKVR.A
148	532.8110	1063.6074	1063.6026	0.0049	1	56	0.0012	1		R.LASYLDKVR.A
159	545.7705	1089.5264	1089.5237	0.0027	0	56	0.0015	1		K.VTMQNLNDR.L
226	621.7820	1241.5495	1241.5458	0.0037	0	23	1.1	1		K.NHEEEMNALR.G
240	633.8407	1265.6669	1265.6615	0.0053	1	27	1.3	1		R.TKYETELNLR.M
247	651.3356	1300.6567	1300.6510	0.0057	0	119	8.2e-010	1		R.ALEEANADLEVK.I
248	651.3360	1300.6575	1300.6510	0.0065	0	(118)	8.5e-010	1		R.ALEEANADLEVK.I
283	681.3514	1360.6882	1360.6834	0.0048	0	70	7.1e-005	1		R.EVATNSELVQSGK.S
304	713.3553	1424.6961	1424.6896	0.0066	0	99	6.8e-008	1	U	R.APSTYGGGLSVSSSR.F
306	719.8567	1437.6988	1437.6922	0.0066	1	58	0.00074	1		R.ILNEMRDQYEK.M
307	480.2402	1437.6988	1437.6922	0.0066	1	(39)	0.065	1		R.ILNEMRDQYEK.M
308	719.8571	1437.6996	1437.6922	0.0074	1	(56)	0.0014	1		R.ILNEMRDQYEK.M
309	480.2405	1437.6996	1437.6922	0.0074	1	(9)	69	1		R.ILNEMRDQYEK.M
396	1043.5028	2084.9909	2084.9797	0.0112	0	70	2.6e-005	1		R.GQVGGDVNVEMDAAPGVDLSR.I
440	770.3644	2308.0713	2308.0567	0.0146	0	28	0.31	1	U	R.LLEGEDAHLSSSQFSSGSQSSR.D
442	778.0790	2331.2151	2331.2030	0.0121	2	31	0.28	1		R.EVATNSELVQSGKSEISELRR.T

9. CH60_CITK8 **Mass:** 57543 **Score:** 418 **Matches:** 16(15) **Sequences:** 5(5) **emPAI:** 0.42
kDa chaperonin OS=Citrobacter koseri (strain ATCC BAA-895 / CDC 4225-83 / SGSC4696) GN=groL PE=3 SV=1
Check to include this hit in error tolerant search or archive report

Query	Observed	Mr(expt)	Mr(calc)	Delta	Miss	Score	Expect	Rank	Unique	Peptide
476	889.1182	2664.3327	2664.3238	0.0089	0	(42)	0.017	1	U	K.AMLQDIATLTGGTVISEEIGMELEK.S + Oxidation (M)
477	889.1188	2664.3346	2664.3238	0.0109	0	53	0.0015	1	U	K.AMLQDIATLTGGTVISEEIGMELEK.S + Oxidation (M)
478	889.1189	2664.3350	2664.3238	0.0112	0	(44)	0.014	1	U	K.AMLQDIATLTGGTVISEEIGMELEK.S + Oxidation (M)
480	908.5092	2722.5057	2722.4939	0.0118	0	(63)	5.5e-005	1		K.AGKPLLIIAEDVEGEALATLVVNTMR.G
481	908.5117	2722.5134	2722.4939	0.0195	0	(39)	0.012	1		K.AGKPLLIIAEDVEGEALATLVVNTMR.G
483	913.8386	2738.4941	2738.4888	0.0053	0	(75)	5.3e-006	1		K.AGKPLLIIAEDVEGEALATLVVNTMR.G + Oxidation (M)
484	913.8389	2738.4948	2738.4888	0.0060	0	81	1.2e-006	1		K.AGKPLLIIAEDVEGEALATLVVNTMR.G + Oxidation (M)
485	913.8390	2738.4953	2738.4888	0.0065	0	(55)	0.00047	1		K.AGKPLLIIAEDVEGEALATLVVNTMR.G + Oxidation (M)
486	913.8400	2738.4982	2738.4888	0.0094	0	(61)	0.00013	1		K.AGKPLLIIAEDVEGEALATLVVNTMR.G + Oxidation (M)
487	913.8411	2738.5015	2738.4888	0.0127	0	(76)	3.9e-006	1		K.AGKPLLIIAEDVEGEALATLVVNTMR.G + Oxidation (M)
492	931.8190	2792.4352	2792.4187	0.0165	1	(49)	0.0035	1	U	R.KAMLQDIATLTGGTVISEEIGMELEK.S + Oxidation (M)
493	931.8222	2792.4448	2792.4187	0.0261	1	75	8.9e-006	1	U	R.KAMLQDIATLTGGTVISEEIGMELEK.S + Oxidation (M)
500	956.4513	2866.3321	2866.3179	0.0142	0	78	2.6e-006	1	U	K.EGVITVEDGTGLQDELDVVEGMQFDR.G + Oxidation (M)
501	956.4526	2866.3361	2866.3179	0.0182	0	(63)	7.3e-005	1	U	K.EGVITVEDGTGLQDELDVVEGMQFDR.G + Oxidation (M)
524	810.6849	3238.7107	3238.7165	-0.0058	1	39	0.02	1	U	R.GYLSPYFINKPETGAVELESPFILLADKK.I
525	810.6931	3238.7432	3238.7165	0.0267	1	(31)	0.095	1	U	R.GYLSPYFINKPETGAVELESPFILLADKK.I

Proteins matching the same set of peptides:

CH60_ECO24 **Mass:** 57464 **Score:** 418 **Matches:** 16(15) **Sequences:** 5(5)
60 kDa chaperonin OS=Escherichia coli O139:H28 (strain E24377A / ETEC) GN=groL PE=3 SV=1

CH60_ECO27 **Mass:** 57464 **Score:** 418 **Matches:** 16(15) **Sequences:** 5(5)
60 kDa chaperonin OS=Escherichia coli O127:H6 (strain E2348/69 / EPEC) GN=groL PE=3 SV=1

CH60_ECO45 **Mass:** 57464 **Score:** 418 **Matches:** 16(15) **Sequences:** 5(5)
60 kDa chaperonin OS=Escherichia coli O45:K1 (strain S88 / ExPEC) GN=groL PE=3 SV=1

CH60_ECO55 **Mass:** 57464 **Score:** 418 **Matches:** 16(15) **Sequences:** 5(5)
60 kDa chaperonin OS=Escherichia coli (strain 55989 / EAEC) GN=groL PE=3 SV=1

CH60_ECO57 **Mass:** 57464 **Score:** 418 **Matches:** 16(15) **Sequences:** 5(5)
60 kDa chaperonin OS=Escherichia coli O157:H7 GN=groL PE=3 SV=2

CH60_ECO5E **Mass:** 57464 **Score:** 418 **Matches:** 16(15) **Sequences:** 5(5)
60 kDa chaperonin OS=Escherichia coli O157:H7 (strain EC4115 / EHEC) GN=groL PE=3 SV=1

CH60_ECO7I **Mass:** 57464 **Score:** 418 **Matches:** 16(15) **Sequences:** 5(5)
60 kDa chaperonin OS=Escherichia coli O7:K1 (strain IAI39 / ExPEC) GN=groL PE=3 SV=1

CH60_ECO81 **Mass:** 57464 **Score:** 418 **Matches:** 16(15) **Sequences:** 5(5)
60 kDa chaperonin OS=Escherichia coli O81 (strain ED1a) GN=groL PE=3 SV=1

CH60_ECO8A **Mass:** 57464 **Score:** 418 **Matches:** 16(15) **Sequences:** 5(5)
60 kDa chaperonin OS=Escherichia coli O8 (strain IAI1) GN=groL PE=3 SV=1

CH60_ECOBW **Mass:** 57464 **Score:** 418 **Matches:** 16(15) **Sequences:** 5(5)
60 kDa chaperonin OS=Escherichia coli (strain K12 / BW2952) GN=groL PE=3 SV=1

CH60_ECODH **Mass:** 57464 **Score:** 418 **Matches:** 16(15) **Sequences:** 5(5)
60 kDa chaperonin OS=Escherichia coli (strain K12 / DH10B) GN=groL PE=3 SV=1

CH60_ECOHS **Mass:** 57464 **Score:** 418 **Matches:** 16(15) **Sequences:** 5(5)
60 kDa chaperonin OS=Escherichia coli O9:H4 (strain HS) GN=groL PE=3 SV=1

CH60_ECOL5 **Mass:** 57464 **Score:** 418 **Matches:** 16(15) **Sequences:** 5(5)
60 kDa chaperonin OS=Escherichia coli O6:K15:H31 (strain 536 / UPEC) GN=groL PE=3 SV=1

CH60_ECOL6 **Mass:** 57464 **Score:** 418 **Matches:** 16(15) **Sequences:** 5(5)
60 kDa chaperonin OS=Escherichia coli O6 GN=groL PE=3 SV=2

CH60_ECOLC **Mass:** 57464 **Score:** 418 **Matches:** 16(15) **Sequences:** 5(5)
60 kDa chaperonin OS=Escherichia coli (strain ATCC 8739 / DSM 1576 / Crooks) GN=groL PE=3 SV=1

CH60_ECOLI **Mass:** 57464 **Score:** 418 **Matches:** 16(15) **Sequences:** 5(5)
60 kDa chaperonin OS=Escherichia coli (strain K12) GN=groL PE=1 SV=2

CH60_ECOLU **Mass:** 57464 **Score:** 418 **Matches:** 16(15) **Sequences:** 5(5)
60 kDa chaperonin OS=Escherichia coli O17:K52:H18 (strain UMN026 / ExPEC) GN=groL PE=3 SV=1
CH60_ECOSE **Mass:** 57464 **Score:** 418 **Matches:** 16(15) **Sequences:** 5(5)
60 kDa chaperonin OS=Escherichia coli (strain SE11) GN=groL PE=3 SV=1
CH60_ECOSM **Mass:** 57464 **Score:** 418 **Matches:** 16(15) **Sequences:** 5(5)
60 kDa chaperonin OS=Escherichia coli (strain SMS-3-5 / SECEC) GN=groL PE=3 SV=1
CH60_ECOUT **Mass:** 57464 **Score:** 418 **Matches:** 16(15) **Sequences:** 5(5)
60 kDa chaperonin OS=Escherichia coli (strain UTI89 / UPEC) GN=groL PE=1 SV=1
CH60_ESCF3 **Mass:** 57522 **Score:** 418 **Matches:** 16(15) **Sequences:** 5(5)
60 kDa chaperonin OS=Escherichia fergusonii (strain ATCC 35469 / DSM 13698 / CDC 0568-73) GN=groL PE=3 SV=1
CH60_KLEPL **Mass:** 56791 **Score:** 418 **Matches:** 16(15) **Sequences:** 5(5)
60 kDa chaperonin (Fragment) OS=Klebsiella planticola GN=groL PE=3 SV=1
CH60_SALA4 **Mass:** 57176 **Score:** 418 **Matches:** 16(15) **Sequences:** 5(5)
60 kDa chaperonin OS=Salmonella agona (strain SL483) GN=groL PE=3 SV=1
CH60_SALAR **Mass:** 57434 **Score:** 418 **Matches:** 16(15) **Sequences:** 5(5)
60 kDa chaperonin OS=Salmonella arizonae (strain ATCC BAA-731 / CDC346-86 / RSK2980) GN=groL PE=3 SV=1
CH60_SALCH **Mass:** 57421 **Score:** 418 **Matches:** 16(15) **Sequences:** 5(5)
60 kDa chaperonin OS=Salmonella choleraesuis GN=groL PE=3 SV=1
CH60_SALDC **Mass:** 57421 **Score:** 418 **Matches:** 16(15) **Sequences:** 5(5)
60 kDa chaperonin OS=Salmonella dublin (strain CT_02021853) GN=groL PE=3 SV=1
CH60_SALEP **Mass:** 57421 **Score:** 418 **Matches:** 16(15) **Sequences:** 5(5)
60 kDa chaperonin OS=Salmonella enteritidis PT4 (strain P125109) GN=groL PE=3 SV=1
CH60_SALG2 **Mass:** 57349 **Score:** 418 **Matches:** 16(15) **Sequences:** 5(5)
60 kDa chaperonin OS=Salmonella gallinarum (strain 287/91 / NCTC 13346) GN=groL PE=3 SV=1
CH60_SALHS **Mass:** 57421 **Score:** 418 **Matches:** 16(15) **Sequences:** 5(5)
60 kDa chaperonin OS=Salmonella heidelberg (strain SL476) GN=groL PE=3 SV=1
CH60_SALPA **Mass:** 57421 **Score:** 418 **Matches:** 16(15) **Sequences:** 5(5)
60 kDa chaperonin OS=Salmonella paratyphi A GN=groL PE=3 SV=1
CH60_SALPB **Mass:** 57421 **Score:** 418 **Matches:** 16(15) **Sequences:** 5(5)
60 kDa chaperonin OS=Salmonella paratyphi B (strain ATCC BAA-1250 / SPB7) GN=groL PE=3 SV=1
CH60_SALPC **Mass:** 57421 **Score:** 418 **Matches:** 16(15) **Sequences:** 5(5)
60 kDa chaperonin OS=Salmonella paratyphi C (strain RKS4594) GN=groL PE=3 SV=1
CH60_SALPK **Mass:** 57421 **Score:** 418 **Matches:** 16(15) **Sequences:** 5(5)
60 kDa chaperonin OS=Salmonella paratyphi A (strain AKU_12601) GN=groL PE=3 SV=1
CH60_SALSV **Mass:** 57421 **Score:** 418 **Matches:** 16(15) **Sequences:** 5(5)
60 kDa chaperonin OS=Salmonella schwarzengrund (strain CVM19633) GN=groL PE=3 SV=1
CH60_SALTI **Mass:** 57421 **Score:** 418 **Matches:** 16(15) **Sequences:** 5(5)
60 kDa chaperonin OS=Salmonella typhi GN=groL PE=3 SV=2
CH60_SALTY **Mass:** 57421 **Score:** 418 **Matches:** 16(15) **Sequences:** 5(5)
60 kDa chaperonin OS=Salmonella typhimurium GN=groL PE=3 SV=2
CH60_SHIB3 **Mass:** 57464 **Score:** 418 **Matches:** 16(15) **Sequences:** 5(5)
60 kDa chaperonin OS=Shigella boydii serotype 18 (strain CDC 3083-94 / BS512) GN=groL PE=3 SV=1
CH60_SHIBS **Mass:** 57464 **Score:** 418 **Matches:** 16(15) **Sequences:** 5(5)
60 kDa chaperonin OS=Shigella boydii serotype 4 (strain Sb227) GN=groL PE=3 SV=1
CH60_SHIDS **Mass:** 57464 **Score:** 418 **Matches:** 16(15) **Sequences:** 5(5)
60 kDa chaperonin OS=Shigella dysenteriae serotype 1 (strain Sd197) GN=groL PE=3 SV=1
CH60_SHIF8 **Mass:** 57464 **Score:** 418 **Matches:** 16(15) **Sequences:** 5(5)
60 kDa chaperonin OS=Shigella flexneri serotype 5b (strain 8401) GN=groL PE=3 SV=1
CH60_SHIFL **Mass:** 57464 **Score:** 418 **Matches:** 16(15) **Sequences:** 5(5)
60 kDa chaperonin OS=Shigella flexneri GN=groL PE=3 SV=2
CH60_SHISS **Mass:** 57464 **Score:** 418 **Matches:** 16(15) **Sequences:** 5(5)
60 kDa chaperonin OS=Shigella sonnei (strain Ss046) GN=groL PE=3 SV=1
CH60_STEMA **Mass:** 57766 **Score:** 418 **Matches:** 16(15) **Sequences:** 5(5)
60 kDa chaperonin OS=Stenotrophomonas maltophilia GN=groL PE=3 SV=1
CH601_ECOK1 **Mass:** 57464 **Score:** 418 **Matches:** 16(15) **Sequences:** 5(5)
60 kDa chaperonin 1 OS=Escherichia coli O1:K1 / APEC GN=groL1 PE=3 SV=1

---

10. K2C6B_HUMAN **Mass:** 60315 **Score:** 406 **Matches:** 21(17) **Sequences:** 12(10) **emPAI:** 0.75
Keratin, type II cytoskeletal 6B OS=Homo sapiens GN=KRT6B PE=1 SV=5
Check to include this hit in error tolerant search or archive report

Query	Observed	Mr(expt)	Mr(calc)	Delta	Miss	Score	Expect	Rank	Unique	Peptide
76	827.4315	826.4242	826.4225	0.0017	0	27	0.97	1		K.FASFIDK.V
77	414.2201	826.4256	826.4225	0.0031	0	(10)	50	1		K.FASFIDK.V
**105**	**473.2607**	**944.5069**	**944.5039**	**0.0030**	**1**	**(36)**	**0.26**	**1**		**R.GRLDSELR.N**
**106**	**473.2612**	**944.5078**	**944.5039**	**0.0038**	**1**	**42**	**0.072**	**1**		**R.GRLDSELR.N**
157	541.8055	1081.5965	1081.5920	0.0044	1	(51)	0.0039	1		K.FASFIDKVR.F
158	541.8060	1081.5973	1081.5920	0.0053	1	61	0.00043	1		K.FASFIDKVR.F
**167**	**554.2748**	**1106.5350**	**1106.5356**	**-0.0006**	**0**	**(43)**	**0.032**	**1**		**K.AQYEEIAQR.S**
**168**	**554.2763**	**1106.5381**	**1106.5356**	**0.0025**	**0**	**55**	**0.0021**	**1**		**K.AQYEEIAQR.S**
196	590.3059	1178.5972	1178.5931	0.0041	0	63	0.00031	1		K.YEELQITAGR.H
**209**	**602.3239**	**1202.6333**	**1202.6295**	**0.0038**	**0**	**(49)**	**0.011**	**1**		**K.WTLLQEQGTK.T**
**210**	**602.3245**	**1202.6344**	**1202.6295**	**0.0048**	**0**	**49**	**0.011**	**1**		**K.WTLLQEQGTK.T**
**214**	**606.7828**	**1211.5511**	**1211.5458**	**0.0052**	**0**	**49**	**0.0034**	**1**		**R.AEAESWYQTK.Y**
254	436.8911	1307.6515	1307.6469	0.0045	2	63	0.00031	1		K.NKYEDEINKR.T
255	436.8913	1307.6522	1307.6469	0.0053	2	(56)	0.0014	1		K.NKYEDEINKR.T
256	436.8917	1307.6532	1307.6469	0.0063	2	(60)	0.0006	1		K.NKYEDEINKR.T
**262**	**665.3687**	**1328.7228**	**1328.7187**	**0.0041**	**0**	**75**	**2.2e-005**	**1**		**R.NLDLDSIIAEVK.A**
**273**	**675.8695**	**1349.7244**	**1349.7191**	**0.0054**	**1**	**46**	**0.017**	**1**		**R.TAAENEFVTLKK.D**
**302**	**704.3618**	**1406.7090**	**1406.7041**	**0.0048**	**0**	**59**	**0.00082**	**1**		**K.ADTLTDEINFLR.A**
**303**	**704.3620**	**1406.7094**	**1406.7041**	**0.0053**	**0**	**(51)**	**0.005**	**1**		**K.ADTLTDEINFLR.A**
**347**	**587.3274**	**1758.9604**	**1758.9516**	**0.0088**	**1**	**57**	**0.00092**	**1**		**K.VLDTKWTLLQEQGTK.T**
**348**	**587.3281**	**1758.9625**	**1758.9516**	**0.0109**	**1**	**(55)**	**0.0014**	**1**		**K.VLDTKWTLLQEQGTK.T**

---

11. K2C5_RAT **Mass:** 61959 **Score:** 406 **Matches:** 21(14) **Sequences:** 11(7) **emPAI:** 0.46
Keratin, type II cytoskeletal 5 OS=Rattus norvegicus GN=Krt5 PE=1 SV=1
Check to include this hit in error tolerant search or archive report

Query	Observed	Mr(expt)	Mr(calc)	Delta	Miss	Score	Expect	Rank	Unique	Peptide
76	827.4315	826.4242	826.4225	0.0017	0	27	0.97	1		K.FASFIDK.V
77	414.2201	826.4256	826.4225	0.0031	0	(10)	50	1		K.FASFIDK.V
105	473.2607	944.5069	944.5039	0.0030	1	(36)	0.26	1		R.GRLDSELR.N
106	473.2612	944.5078	944.5039	0.0038	1	42	0.072	1		R.GRLDSELR.N
154	537.3022	1072.5899	1072.5876	0.0022	1	35	0.31	1		R.LRSEIDNVK.K
155	537.3026	1072.5907	1072.5876	0.0031	1	(27)	2	1		R.LRSEIDNVK.K
157	541.8055	1081.5965	1081.5920	0.0044	1	(51)	0.0039	1		K.FASFIDKVR.F
158	541.8060	1081.5973	1081.5920	0.0053	1	61	0.00043	1		K.FASFIDKVR.F
**206**	**597.7933**	**1193.5720**	**1193.5676**	**0.0043**	**0**	**(39)**	**0.06**	**1**		**K.YEELQQTAGR.H**
**207**	**597.7937**	**1193.5728**	**1193.5676**	**0.0052**	**0**	**54**	**0.0022**	**1**		**K.YEELQQTAGR.H**
208	401.2361	1200.6866	1200.6826	0.0040	2	21	5.2	1		R.LRSEIDNVKK.Q
209	602.3239	1202.6333	1202.6295	0.0038	0	(49)	0.011	1		K.WTLLQEQGTK.T

Query	Observed	Mr(expt)	Mr(calc)	Delta	Miss	Score	Expect	Rank	Unique	Peptide
210	602.3245	1202.6344	1202.6295	0.0048	0	49	0.011	1		K.WTLLQEQGTK.T
249	651.8633	1301.7120	1301.7078	0.0042	0	(77)	1.2e-005	1		R.SLDLDSIIAEVK.A
250	651.8635	1301.7125	1301.7078	0.0047	0	97	1.3e-007	1		R.SLDLDSIIAEVK.A
254	436.8911	1307.6515	1307.6469	0.0045	2	63	0.00031	1		K.NKYEDEINKR.T
255	436.8913	1307.6522	1307.6469	0.0053	2	(56)	0.0014	1		K.NKYEDEINKR.T
256	436.8917	1307.6532	1307.6469	0.0063	2	(60)	0.0006	1		K.NKYEDEINKR.T
347	587.3274	1758.9604	1758.9516	0.0088	1	57	0.00092	1		K.VLDTKWTLLQEQGTK.T
348	587.3281	1758.9625	1758.9516	0.0109	1	(55)	0.0014	1		K.VLDTKWTLLQEQGTK.T
**454**	**806.7160**	**2417.1261**	**2417.1135**	**0.0126**	**1**	**58**	**0.00031**	**1**		**R.TEAESWYQTKYEELQQTAGR.H**

12. K2C6C_HUMAN **Mass:** 60273 **Score:** 395 **Matches:** 21(16) **Sequences:** 12(9) **emPAI:** 0.75
Keratin, type II cytoskeletal 6C OS=Homo sapiens GN=KRT6C PE=1 SV=3
Check to include this hit in error tolerant search or archive report

Query	Observed	Mr(expt)	Mr(calc)	Delta	Miss	Score	Expect	Rank	Unique	Peptide
76	827.4315	826.4242	826.4225	0.0017	0	27	0.97	1		K.FASFIDK.V
77	414.2201	826.4256	826.4225	0.0031	0	(10)	50	1		K.FASFIDK.V
105	473.2607	944.5069	944.5039	0.0030	1	(36)	0.26	1		R.GRLDSELR.N
106	473.2612	944.5078	944.5039	0.0038	1	42	0.072	1		R.GRLDSELR.N
157	541.8055	1081.5965	1081.5920	0.0044	1	(51)	0.0039	1		K.FASFIDKVR.F
158	541.8060	1081.5973	1081.5920	0.0053	1	61	0.00043	1		K.FASFIDKVR.F
167	554.2748	1106.5350	1106.5356	-0.0006	0	(43)	0.032	1		K.AQYEEIAQR.S
168	554.2763	1106.5381	1106.5356	0.0025	0	55	0.0021	1		K.AQYEEIAQR.S
**194**	**583.2980**	**1164.5815**	**1164.5775**	**0.0040**	**0**	**39**	**0.091**	**1**		**K.YEELQVTAGR.H**
209	602.3239	1202.6333	1202.6295	0.0038	0	(49)	0.011	1		K.WTLLQEQGTK.T
210	602.3245	1202.6344	1202.6295	0.0048	0	49	0.011	1		K.WTLLQEQGTK.T
214	606.7828	1211.5511	1211.5458	0.0052	0	49	0.0034	1		R.AEAESWYQTK.Y
254	436.8911	1307.6515	1307.6469	0.0045	2	63	0.00031	1		K.NKYEDEINKR.T
255	436.8913	1307.6522	1307.6469	0.0053	2	(56)	0.0014	1		K.NKYEDEINKR.T
256	436.8917	1307.6532	1307.6469	0.0063	2	(60)	0.0006	1		K.NKYEDEINKR.T
262	665.3687	1328.7228	1328.7187	0.0041	0	75	2.2e-005	1		R.NLDLDSIIAEVK.A
273	675.8695	1349.7244	1349.7191	0.0054	1	46	0.017	1		R.TAAENEFVTLKK.D
302	704.3618	1406.7090	1406.7041	0.0048	0	59	0.00082	1		K.ADTLTDEINFLR.A
303	704.3620	1406.7094	1406.7041	0.0053	0	(51)	0.005	1		K.ADTLTDEINFLR.A
347	587.3274	1758.9604	1758.9516	0.0088	1	57	0.00092	1		K.VLDTKWTLLQEQGTK.T
348	587.3281	1758.9625	1758.9516	0.0109	1	(55)	0.0014	1		K.VLDTKWTLLQEQGTK.T

13. K2C5_HUMAN **Mass:** 62568 **Score:** 365 **Matches:** 21(14) **Sequences:** 12(8) **emPAI:** 0.62
Keratin, type II cytoskeletal 5 OS=Homo sapiens GN=KRT5 PE=1 SV=3
Check to include this hit in error tolerant search or archive report

Query	Observed	Mr(expt)	Mr(calc)	Delta	Miss	Score	Expect	Rank	Unique	Peptide
76	827.4315	826.4242	826.4225	0.0017	0	27	0.97	1		K.FASFIDK.V
77	414.2201	826.4256	826.4225	0.0031	0	(10)	50	1		K.FASFIDK.V
**104**	**469.7528**	**937.4911**	**937.4869**	**0.0043**	**0**	**18**	**9.1**	**1**		**R.SLYNLGGSK.R**
105	473.2607	944.5069	944.5039	0.0030	1	(36)	0.26	1		R.GRLDSELR.N
106	473.2612	944.5078	944.5039	0.0038	1	42	0.072	1		R.GRLDSELR.N
157	541.8055	1081.5965	1081.5920	0.0044	1	(51)	0.0039	1		K.FASFIDKVR.F
158	541.8060	1081.5973	1081.5920	0.0053	1	61	0.00043	1		K.FASFIDKVR.F
**160**	**547.2687**	**1092.5228**	**1092.5199**	**0.0028**	**0**	**36**	**0.14**	**1**		**K.AQYEEIANR.S**
**161**	**547.2693**	**1092.5240**	**1092.5199**	**0.0041**	**0**	**(29)**	**0.76**	**1**		**K.AQYEEIANR.S**
**172**	**556.2934**	**1110.5722**	**1110.5669**	**0.0052**	**0**	**57**	**0.001**	**1**	**U**	**R.ISISTSGGSFR.N**
206	597.7933	1193.5720	1193.5676	0.0043	0	(39)	0.06	1		K.YEELQQTAGR.H
207	597.7937	1193.5728	1193.5676	0.0052	0	54	0.0022	1		K.YEELQQTAGR.H
209	602.3239	1202.6333	1202.6295	0.0038	0	(49)	0.011	1		K.WTLLQEQGTK.T
210	602.3245	1202.6344	1202.6295	0.0048	0	49	0.011	1		K.WTLLQEQGTK.T
254	436.8911	1307.6515	1307.6469	0.0045	2	63	0.00031	1		K.NKYEDEINKR.T
255	436.8913	1307.6522	1307.6469	0.0053	2	(56)	0.0014	1		K.NKYEDEINKR.T
256	436.8917	1307.6532	1307.6469	0.0063	2	(60)	0.0006	1		K.NKYEDEINKR.T
262	665.3687	1328.7228	1328.7187	0.0041	0	75	2.2e-005	1		R.NLDLDSIIAEVK.A
347	587.3274	1758.9604	1758.9516	0.0088	1	57	0.00092	1		K.VLDTKWTLLQEQGTK.T
348	587.3281	1758.9625	1758.9516	0.0109	1	(55)	0.0014	1		K.VLDTKWTLLQEQGTK.T
454	806.7160	2417.1261	2417.1135	0.0126	1	58	0.00031	1		R.TEAESWYQTKYEELQQTAGR.H

14. K2C6A_HUMAN **Mass:** 60293 **Score:** 359 **Matches:** 20(15) **Sequences:** 12(9) **emPAI:** 0.75
Keratin, type II cytoskeletal 6A OS=Homo sapiens GN=KRT6A PE=1 SV=3
Check to include this hit in error tolerant search or archive report

Query	Observed	Mr(expt)	Mr(calc)	Delta	Miss	Score	Expect	Rank	Unique	Peptide
76	827.4315	826.4242	826.4225	0.0017	0	27	0.97	1		K.FASFIDK.V
77	414.2201	826.4256	826.4225	0.0031	0	(10)	50	1		K.FASFIDK.V
105	473.2607	944.5069	944.5039	0.0030	1	(36)	0.26	1		R.GRLDSELR.G
106	473.2612	944.5078	944.5039	0.0038	1	42	0.072	1		R.GRLDSELR.G
**122**	**506.7589**	**1011.5031**	**1011.4985**	**0.0046**	**0**	**66**	**0.00012**	**1**	**U**	**R.SGFSSVSVSR.S**
157	541.8055	1081.5965	1081.5920	0.0044	1	(51)	0.0039	1		K.FASFIDKVR.F
158	541.8060	1081.5973	1081.5920	0.0053	1	61	0.00043	1		K.FASFIDKVR.F
167	554.2748	1106.5350	1106.5356	-0.0006	0	(43)	0.032	1		K.AQYEEIAQR.S
168	554.2763	1106.5381	1106.5356	0.0025	0	55	0.0021	1		K.AQYEEIAQR.S
194	583.2980	1164.5815	1164.5775	0.0040	0	39	0.091	1		K.YEELQVTAGR.H
209	602.3239	1202.6333	1202.6295	0.0038	0	(49)	0.011	1		K.WTLLQEQGTK.T
210	602.3245	1202.6344	1202.6295	0.0048	0	49	0.011	1		K.WTLLQEQGTK.T
214	606.7828	1211.5511	1211.5458	0.0052	0	49	0.0034	1		R.AEAESWYQTK.Y
254	436.8911	1307.6515	1307.6469	0.0045	2	63	0.00031	1		K.NKYEDEINKR.T
255	436.8913	1307.6522	1307.6469	0.0053	2	(56)	0.0014	1		K.NKYEDEINKR.T
256	436.8917	1307.6532	1307.6469	0.0063	2	(60)	0.0006	1		K.NKYEDEINKR.T
262	665.3687	1328.7228	1328.7187	0.0041	0	75	2.2e-005	1		R.NLDLDSIIAEVK.A
273	675.8695	1349.7244	1349.7191	0.0054	1	46	0.017	1		R.TAAENEFVTLKK.D
302	704.3618	1406.7090	1406.7041	0.0048	0	59	0.00082	1		K.ADTLTDEINFLR.A
303	704.3620	1406.7094	1406.7041	0.0053	0	(51)	0.005	1		K.ADTLTDEINFLR.A

15. K22E_HUMAN **Mass:** 65678 **Score:** 351 **Matches:** 13(9) **Sequences:** 8(5) **emPAI:** 0.36
Keratin, type II cytoskeletal 2 epidermal OS=Homo sapiens GN=KRT2 PE=1 SV=2
Check to include this hit in error tolerant search or archive report

Query	Observed	Mr(expt)	Mr(calc)	Delta	Miss	Score	Expect	Rank	Unique	Peptide
76	827.4315	826.4242	826.4225	0.0017	0	27	0.97	1		K.FASFIDK.V

77	414.2201	826.4256	826.4225	0.0031	0	(10)	50	1		K.FASFIDK.V
79	**416.2428**	**830.4711**	**830.4862**	**-0.0150**	**0**	**7**	**2.4e+002**	**1**	**U**	**R.SLVGLGGTK.S**
108	487.2717	972.5288	972.5240	0.0049	0	41	0.08	1		K.IEISELNR.V
157	541.8055	1081.5965	1081.5920	0.0044	1	(51)	0.0039	1		K.FASFIDKVR.F
158	541.8060	1081.5973	1081.5920	0.0053	1	61	0.00043	1		K.FASFIDKVR.F
167	554.2748	1106.5350	1106.5356	-0.0006	0	(43)	0.032	1		K.AQYEEIAQR.S
168	554.2763	1106.5381	1106.5356	0.0025	0	55	0.0021	1		K.AQYEEIAQR.S
262	665.3687	1328.7228	1328.7187	0.0041	0	75	2.2e-005	1		R.NLDLDSIIAEVK.A
313	**730.9065**	**1459.7984**	**1459.7922**	**0.0062**	**0**	**(42)**	**0.041**	**1**	**U**	**K.VDLLNQEIEFLK.V**
314	**730.9071**	**1459.7997**	**1459.7922**	**0.0075**	**0**	**92**	**3.9e-007**	**1**	**U**	**K.VDLLNQEIEFLK.V**
316	738.3987	1474.7829	1474.7780	0.0049	0	113	3.1e-009	1		R.FLEQQNQVLQTK.W
317	738.3998	1474.7850	1474.7780	0.0070	0	(112)	4.1e-009	1		R.FLEQQNQVLQTK.W

16. K1C17_HUMAN **Mass:** 48361 **Score:** 198 **Matches:** 20(10) **Sequences:** 11(6) **emPAI:** 1.15

Keratin, type I cytoskeletal 17 OS=Homo sapiens GN=KRT17 PE=1 SV=2

Check to include this hit in error tolerant search or archive report

Query	Observed	Mr(expt)	Mr(calc)	Delta	Miss	Score	Expect	Rank	Unique	Peptide
71	404.2052	806.3958	806.3923	0.0036	0	56	0.0021	1		R.LAADDFR.T
72	404.2052	806.3959	806.3923	0.0036	0	(51)	0.0076	1		R.LAADDFR.T
73	809.4437	808.4364	808.4330	0.0033	0	(32)	0.46	1		R.LASYLDK.V
74	405.2255	808.4364	808.4330	0.0033	0	41	0.053	1		R.LASYLDK.V
112	495.2739	988.5332	988.5301	0.0030	1	(13)	48	7		K.SEISELRR.T
113	495.2739	988.5332	988.5301	0.0030	1	22	6.4	5		K.SEISELRR.T
114	495.2740	988.5334	988.5301	0.0033	1	(11)	81	9		K.SEISELRR.T
124	515.3031	1028.5916	1028.5866	0.0050	0	(43)	0.046	1		R.VLDELTLAR.A
125	515.3033	1028.5921	1028.5866	0.0055	0	43	0.046	1		R.VLDELTLAR.A
130	518.7721	1035.5295	1035.5250	0.0046	1	35	0.24	1		K.IRDWYQR.Q
140	**531.7561**	**1061.4977**	**1061.4924**	**0.0053**	**0**	**38**	**0.084**	**1**		**K.ATMQNLNDR.L**
147	532.8106	1063.6066	1063.6026	0.0041	1	(51)	0.0038	1		R.LASYLDKVR.A
148	532.8110	1063.6074	1063.6026	0.0049	1	56	0.0012	1		R.LASYLDKVR.A
226	621.7820	1241.5495	1241.5458	0.0037	0	23	1.1	1		K.NHEEEMNALR.G
283	681.3514	1360.6882	1360.6834	0.0048	0	70	7.1e-005	1		R.EVATNSELVQSGK.S
306	719.8567	1437.6988	1437.6922	0.0066	1	58	0.00074	1		R.ILNEMRDQYEK.M
307	480.2402	1437.6988	1437.6922	0.0066	1	(39)	0.065	1		R.ILNEMRDQYEK.M
308	719.8571	1437.6996	1437.6922	0.0074	1	(56)	0.0014	1		R.ILNEMRDQYEK.M
309	480.2405	1437.6996	1437.6922	0.0074	1	(9)	69	1		R.ILNEMRDQYEK.M
442	778.0790	2331.2151	2331.2030	0.0121	2	31	0.28	1		R.EVATNSELVQSGKSEISELRR.T

17. HORN_HUMAN **Mass:** 283140 **Score:** 140 **Matches:** 4(4) **Sequences:** 2(2) **emPAI:** 0.02

Hornerin OS=Homo sapiens GN=HRNR PE=1 SV=2

Check to include this hit in error tolerant search or archive report

Query	Observed	Mr(expt)	Mr(calc)	Delta	Miss	Score	Expect	Rank	Unique	Peptide
331	**528.9113**	**1583.7121**	**1583.7077**	**0.0044**	**0**	**(47)**	**0.0044**	**1**	**U**	**R.GPYESGSGHSSGLGHR.E**
332	**528.9117**	**1583.7132**	**1583.7077**	**0.0055**	**0**	**73**	**1e-005**	**1**	**U**	**R.GPYESGSGHSSGLGHR.E**
333	**528.9122**	**1583.7148**	**1583.7077**	**0.0071**	**0**	**(56)**	**0.00055**	**1**	**U**	**R.GPYESGSGHSSGLGHR.E**
376	**649.9751**	**1946.9035**	**1946.8943**	**0.0091**	**0**	**48**	**0.0036**	**1**	**U**	**R.QSLGHGQHGSGSGQSPSPSR.G**

18. ALBU_HUMAN **Mass:** 71317 **Score:** 97 **Matches:** 6(3) **Sequences:** 6(3) **emPAI:** 0.15

Serum albumin OS=Homo sapiens GN=ALB PE=1 SV=2

Check to include this hit in error tolerant search or archive report

Query	Observed	Mr(expt)	Mr(calc)	Delta	Miss	Score	Expect	Rank	Unique	Peptide
107	**480.7738**	**959.5330**	**959.5552**	**-0.0222**	**0**	**15**	**30**	**1**	**U**	**K.FQNALLVR.Y**
119	**500.8072**	**999.5998**	**999.5964**	**0.0034**	**0**	**52**	**0.0041**	**1**	**U**	**K.QTALVELVK.H**
190	**575.3137**	**1148.6128**	**1148.6077**	**0.0051**	**0**	**42**	**0.055**	**1**	**U**	**K.LVNEVTEFAK.T**
312	**722.3262**	**1442.6379**	**1442.6347**	**0.0032**	**0**	**11**	**14**	**1**	**U**	**K.YICENQDSISSK.L**
329	**516.2728**	**1545.7966**	**1545.7894**	**0.0071**	**1**	**12**	**36**	**1**	**U**	**K.LKECCEKPLLEK.S**
338	**547.3201**	**1638.9385**	**1638.9305**	**0.0080**	**1**	**57**	**0.00055**	**1**	**U**	**K.KVPQVSTPTLVEVSR.N**

Proteins matching the same set of peptides:

ALBU_PONAB **Mass:** 71317 **Score:** 97 **Matches:** 6(3) **Sequences:** 6(3)

Serum albumin OS=Pongo abelii GN=ALB PE=2 SV=1

19. K2C4_HUMAN **Mass:** 57649 **Score:** 91 **Matches:** 6(3) **Sequences:** 4(2) **emPAI:** 0.12

Keratin, type II cytoskeletal 4 OS=Homo sapiens GN=KRT4 PE=1 SV=4

Check to include this hit in error tolerant search or archive report

Query	Observed	Mr(expt)	Mr(calc)	Delta	Miss	Score	Expect	Rank	Unique	Peptide
76	827.4315	826.4242	826.4225	0.0017	0	27	0.97	1		K.FASFIDK.V
77	414.2201	826.4256	826.4225	0.0031	0	(10)	50	1		K.FASFIDK.V
167	554.2748	1106.5350	1106.5356	-0.0006	0	(43)	0.032	1		R.AQYEEIAQR.S
168	554.2763	1106.5381	1106.5356	0.0025	0	55	0.0021	1		R.AQYEEIAQR.S
242	639.3594	1276.7043	1276.7027	0.0016	0	61	0.00046	1		K.LALDIEIATYR.K
375	**648.0098**	**1941.0075**	**1940.9996**	**0.0079**	**1**	**5**	**1.3e+002**	**1**	**U**	**K.FASFIDKVQFLEQQNK.V**

20. A1M_RAT **Mass:** 168388 **Score:** 63 **Matches:** 1(1) **Sequences:** 1(1) **emPAI:** 0.02

Alpha-1-macroglobulin OS=Rattus norvegicus GN=A1m PE=1 SV=1

Check to include this hit in error tolerant search or archive report

Query	Observed	Mr(expt)	Mr(calc)	Delta	Miss	Score	Expect	Rank	Unique	Peptide
173	**558.8049**	**1115.5953**	**1115.6339**	**-0.0386**	**1**	**63**	**0.00047**	**1**	**U**	**R.KTVSWAVTPK.S**

Proteins matching the same set of peptides:

A2M_MOUSE **Mass:** 167144 **Score:** 63 **Matches:** 1(1) **Sequences:** 1(1)

Alpha-2-macroglobulin OS=Mus musculus GN=A2m PE=1 SV=2

21. A2MG_HUMAN **Mass:** 164614 **Score:** 58 **Matches:** 5(1) **Sequences:** 5(1) **emPAI:** 0.06

Alpha-2-macroglobulin OS=Homo sapiens GN=A2M PE=1 SV=2

Check to include this hit in error tolerant search or archive report

Query	Observed	Mr(expt)	Mr(calc)	Delta	Miss	Score	Expect	Rank	Unique	Peptide
173	558.8049	1115.5953	1115.5975	-0.0022	0	63	0.00047	1	U	R.QTVSWAVTPK.S
189	**574.8189**	**1147.6233**	**1147.6138**	**0.0094**	**0**	**27**	**1.5**	**1**	**U**	**R.QGIPFFGQVR.L**
328	**515.9413**	**1544.8020**	**1544.7947**	**0.0073**	**0**	**21**	**4.8**	**1**	**U**	**R.LVHVEEPHTETVR.K**
362	**923.0254**	**1844.0361**	**1844.0295**	**0.0066**	**0**	**27**	**0.54**	**1**	**U**	**R.LLIYAVLPTGDVIGDSAK.Y**
395	**1042.0145**	**2082.0145**	**2082.0052**	**0.0093**	**0**	**13**	**16**	**1**	**U**	**K.AGAFCLSEDAGLGISSTASLR.A**

Peptide matches not assigned to protein hits: (no details means no match)

Query	Observed	Mr(expt)	Mr(calc)	Delta	Miss	Score	Expect	Rank	Unique	Peptide
335	546.9602	1637.8587	1637.8889	-0.0302	2	59	0.00057	1		SLNNKFASFIDKVR
80	416.7494	831.4843	831.4814	0.0029	0	36	0.23	1		SLSLGSLR
392	414.2187	2066.0572	2066.0717	-0.0145	2	32	0.26	1		AAMKAETELGLQAKSFIDK + Oxidation (M)
81	416.7498	831.4851	831.4814	0.0037	0	31	0.79	1		SLSLGSLR
193	581.8088	1161.6031	1161.5666	0.0365	0	25	2.5	1		DGIAQLTYAGPG
115	495.2743	988.5341	988.5077	0.0264	0	25	3	1		TDLGVDIEK
354	887.9516	1773.8886	1773.8647	0.0238	0	25	1.7	1		INDEVLLEYFNFMK
121	502.2819	1002.5493	1002.5458	0.0036	1	22	6.5	1		SELRAVSNK
101	463.7243	925.4340	925.4440	-0.0100	1	20	4.2	1		DGYMIRR + Oxidation (M)
467	517.2572	2581.2497	2581.2732	-0.0235	2	20	3.2	1		TLAVADHNVPTTDRSKGIADEESR
137	523.7811	1045.5475	1045.5404	0.0072	1	19	10	1		LREGTLDDK
420	737.7120	2210.1141	2210.1405	-0.0264	2	19	4.8	1		TFLGIFNEIELKQKECNK
195	588.3230	1174.6314	1174.6016	0.0298	0	17	14	1		NNNTDLMILK
213	605.8032	1209.5918	1209.6030	-0.0112	1	17	11	1		LYPEFGNKDK
543	597.3085	3577.8073	3577.7909	0.0165	1	16	4.6	1		SIKSLSALEELYNSQPVVATTCMGVNHPIFTR + Oxidation (M)
355	887.9557	1773.8968	1773.8857	0.0111	2	13	26	1		GRVEELGEEIGRESSK
87	846.4448	845.4375	845.4681	-0.0306	0	13	61	1		AMLVDVAK
343	567.3282	1698.9628	1698.9377	0.0251	2	12	20	1		AGELVRTAAKTLGGGGGGK
352	883.9005	1765.7864	1765.8226	-0.0362	1	12	11	1		KMEQESIDQLCELK + Oxidation (M)
241	638.3262	1274.6378	1274.6215	0.0163	1	11	55	1		RSLSDDVAEQR
379	665.3710	1993.0911	1993.1255	-0.0343	2	10	27	1		MKGIILAGGSGTRLHPITR + Oxidation (M)
96	456.2814	910.5482	910.5712	-0.0230	2	10	27	1		APRKPSKK
363	926.4716	1850.9286	1850.9414	-0.0128	1	10	51	1		HSDAIFTEEYSKLLAK
369	623.3035	1866.8888	1866.8564	0.0325	1	10	36	1		NRMLESLTSECENIR + Oxidation (M)
351	883.8988	1765.7831	1765.8318	-0.0487	1	10	16	1		SNDPKSQNHPGWLMR
212	605.8031	1209.5916	1209.5924	-0.0008	0	9	61	1		MITASSPHNPR
421	738.3800	2212.1182	2212.1344	-0.0161	1	9	51	1		DAGAAAIMPLAAPIGSNKGLCAK + Oxidation (M)
228	630.2792	1258.5438	1258.5441	-0.0003	0	9	19	1		FDHYCSFVGK
86	846.4442	845.4369	845.4495	-0.0126	0	8	1.7e+002	1		DTVVEVGK
27	538.3075	537.3002	537.2547	0.0455	0	8	1e+002	1		GGYNK
373	639.3045	1914.8918	1914.8683	0.0235	1	8	39	1		QSRFIAWTFMDDDQR
549	617.8450	3701.0264	3701.0080	0.0184	2	8	7.8	1		QVQILAEKLLADNIVPFFPETLTKYGDLELQR
274	678.2943	1354.5741	1354.6187	-0.0446	1	8	20	1		MSRSEGFGEEVK
413	736.6920	2207.0543	2207.0391	0.0152	1	7	64	1		IVKYELGCMVQGNYEYNK
341	561.2965	1680.8678	1680.8934	-0.0257	0	7	1.1e+002	1		AVDIYTSSQTGSIIVK
85	846.4441	845.4369	845.4494	-0.0126	0	6	2.6e+002	1		AEEIGLSK
551	651.8669	3905.1574	3905.1313	0.0262	1	6	6.8	1		EAGVPLLAYGSFITVLINFLILAFIIFMMVKGMNK + 2 Oxidation (M)
315	492.5839	1474.7299	1474.7277	0.0023	1	6	1.4e+002	1		DAYHRSLGVSGASR
266	670.8375	1339.6604	1339.6884	-0.0280	1	6	1.2e+002	1		KDPAAQFVHEAK
26	538.3074	537.3001	537.2547	0.0454	0	6	1.8e+002	1		GGYNK
330	781.3407	1560.6669	1560.6805	-0.0135	1	6	26	1		VYKDEHPNDDSSR
457	825.0732	2472.1978	2472.2141	-0.0163	2	6	79	1		ANYGYLLEMPVSRLTSDEMKR
479	906.3717	2716.0932	2716.1383	-0.0451	1	5	1.8	1		SIRSMAMHPQEMTMCSGDTSGNLK + 3 Oxidation (M)
276	678.3207	1354.6269	1354.6769	-0.0500	0	5	99	1		VVFQEYAGSEVK
564	704.3493	4220.0520	4220.0030	0.0490	1	5	39	1		ALAQFMIDTHATHHGYQEVYAPYMVNAASMRGTGQLPK + Oxidation (M)
327	510.5635	1528.6686	1528.7059	-0.0373	1	5	43	1		TNTAYERFWEGR
372	635.6218	1903.8437	1903.8921	-0.0484	0	5	42	1		VGGTCLNYGCTYITGLR
425	755.3492	2263.0257	2262.9836	0.0420	2	5	46	1		GQGKNEETSGEGGEDKNEPSSK
422	738.3801	2212.1185	2212.1344	-0.0159	1	5	1.4e+002	1		DAGAAAIMPLAAPIGSNKGLCAK + Oxidation (M)
435	762.0632	2283.1677	2283.1470	0.0207	0	5	1.3e+002	1		LQAIEPSGNYHILHAYCGIK
513	602.3247	3006.5870	3006.5635	0.0235	1	4	66	1		ASTARGYLDQARPRPNLTIVTYATTDR
515	623.3083	3111.5049	3111.5250	-0.0201	1	4	78	1		ALMTEMAATGMEEGRLTASTVGQIVGLQSK + 2 Oxidation (M)
359	609.9428	1826.8065	1826.8330	-0.0265	2	4	48	1		MGQSQSGGHGPGGGKKDDK
541	579.3061	3469.7929	3469.7729	0.0201	1	4	68	1		EAGFSAIYLSGAAYTASRGLPDLGIITSAEIAER
592	908.8133	5446.8361	5446.8057	0.0303	1	4	11	1		VLHDILIEHAPGSADLPRDVPHDLPPPPLVLVACSGGADSMALASALAFEAPK
554	671.3573	4022.1003	4022.0717	0.0285	0	4	45	1		GLEVIEAYYLFSLTPEQIDVVVHPQSIVHSSVYYK
534	828.6993	3310.7680	3310.7451	0.0229	0	4	53	1		LLSSFMLLTSAGYVSLFPLLYESQDFLIK + Oxidation (M)
429	756.7030	2267.0872	2267.1216	-0.0343	2	4	1.4e+002	1		ADVTLEEKGCASQVPSHSPKK
573	578.2733	4618.1282	4618.1672	-0.0390	2	3	18	1		NSSSSEGVFQFSGKMEQFDGKPMEYLVITHPLDKGDPPYPK + 2 Oxidation (M)
553	665.3572	3986.0998	3986.1081	-0.0083	2	3	48	1		LNANLASMVTTIRDQMQYNRSVLEGIIVPLAVVDAR + Oxidation (M)
175	560.2247	1118.4348	1118.4588	-0.0241	1	2	20	1		EADGEDDRGR
466	639.3016	2553.1773	2553.1541	0.0232	1	2	91	1		EVLVDSDSDEELEMRNFADNVK
537	673.3466	3361.6966	3361.7030	-0.0064	2	2	1.3e+002	1		MTLTNYVQEVSLADFGKPLHHKAYWNKR + Oxidation (M)
611	854.4000	6827.1418	6827.1250	0.0168	2	2	5.9	1		HDENDMDTFKLLEDIVSSSSSPTITPPQSLHSSQIIRTTIEEDDQTNSELEMFQNEYNR + Oxidation (M)
388	1013.9760	2025.9374	2025.9434	-0.0060	1	2	1.6e+002	1		MMHGDELRVEPHMGVLK + 3 Oxidation (M)
539	679.3671	3391.7990	3391.7558	0.0432	2	1	94	1		MILGIGIDTVEISRFQRFLDEDNQALLNR + Oxidation (M)
560	691.3294	4141.9326	4141.9045	0.0281	1	1	49	1		SPEEGVVEVQVEVNGVQRWGAVCSDHWGLSEAMVACR + Oxidation (M)
275	678.3206	1354.6267	1354.6551	-0.0284	0	1	2.2e+002	1		ELTHNSMPDIAK
472	657.5463	2626.1562	2626.1972	-0.0410	0	1	50	1		ASASGHMSTAQTTAMVSGGMSKPLMR + 2 Oxidation (M)
563	528.3702	4218.9036	4218.9468	-0.0432	1	1	28	1		RAIILNYASMNNSPLTMEDLEACEDEEIENMYISIK
544	521.2849	3641.9432	3641.9446	-0.0014	1	1	69	1		MAATGKPLSELAAAMTVLPQVLINVPVADKSSIMK + 3 Oxidation (M)
588	668.8548	5342.7803	5342.7413	0.0390	1	1	26	1		IYAATNVPYPLVGAMDLTFVLPLGLFNPVMERFAAHAGDLVPAPGHPDPR + Oxidation (M)
523	539.3077	3229.8028	3229.7645	0.0382	2	1	34	1		RVVVIGGGDSAVDWALMLEPIAKEVTLVHR
579	4877.4668	4876.4595	4876.4601	-0.0006	2	1	53	1		EQIYSILHNPDNVVEQTFKNTIIKSMEESMFRPLIPTQQMK
402	719.6877	2156.0412	2156.0824	-0.0412	1	1	2.5e+002	1		FTYKGTAVMVDGGPALTDLGK + Oxidation (M)
403	719.6877	2156.0412	2156.0459	-0.0047	1	1	2.6e+002	1		IPYEESMLKYGSDKPDLR + Oxidation (M)
78	827.4329	826.4256	826.3973	0.0283	0	1	3.8e+002	1		AFEASFR
404	719.6899	2156.0478	2156.0597	-0.0119	0	1	2.9e+002	1		APVSPALASPSSEVSDVSEATR
506	970.7850	2909.3331	2909.3495	-0.0164	1	0	1e+002	1		QEKPGTACLSKESTTSSASASMNSHSVK
95	908.4309	907.4237	907.4399	-0.0163	1	0	4.8e+002	1		FSEERAIG
610	6789.9698	6788.9625	6788.9604	0.0021	2	0	2	1		QGESSTFSLFVSDLTPDVDDGMLYEFFHYHFSSCCSGKIVLDANGHSKCCGFVSFESER
562	695.8469	4169.0379	4169.0139	0.0240	2	0	1.2e+002	1		DQLPTSMHRYPSMIQKIFFAGEYTANAHGWIDSTIK + Oxidation (M)
496	934.4190	2800.2353	2800.2264	0.0089	0	0	65	1		TGSLWPVTFGLACCAVEMMHAGMAR + 3 Oxidation (M)
123	1023.4778	1022.4705	1022.4781	-0.0076	1	0	4.6e+002	1		KGNQEEYR
517	791.1332	3160.5035	3160.5023	0.0012	2	0	1.7e+002	1		LEFELTEVMKDVDFKVFSGPANSDNGR + Oxidation (M)
1	402.3585	401.3513								
2	412.1690	411.1618								
3	415.2122	414.2050								
4	415.2125	414.2052								
5	415.2128	414.2055								
6	415.3424	414.3351								
7	415.3431	414.3358								
8	425.3096	424.3023								
9	432.2387	431.2315								
10	432.2391	431.2318								
11	432.2405	431.2332								
12	432.2816	431.2743								
13	442.3375	441.3303								
14	444.3487	443.3414								
15	447.2925	446.2852								
16	476.3071	475.2998								
17	476.3076	475.3004								
18	500.3073	499.3001								
19	504.3038	503.2965								
20	509.1495	508.1422								
21	520.3353	519.3280								
22	525.3215	524.3142								
23	528.3648	527.3576								
24	528.3653	527.3580								
25	531.2709	530.2636								
28	539.3353	538.3280								
29	544.3350	543.3278								

30	545.7710	544.7637
31	547.3329	546.3257
32	550.6289	549.6216
33	564.3608	563.3535
34	564.3611	563.3538
35	579.3376	578.3303
36	579.5349	578.5276
37	579.5349	578.5276
38	579.5350	578.5277
39	579.5356	578.5283
40	586.4168	585.4095
41	594.3677	593.3604
42	608.3875	607.3802
43	610.3919	609.3846
44	613.3543	612.3470
45	614.5715	613.5642
46	614.5732	613.5660
47	615.4046	614.3973
48	615.4048	614.3975
49	615.4049	614.3976
50	617.4078	616.4005
51	617.4079	616.4006
52	619.5271	618.5198
53	652.4147	651.4074
54	666.3929	665.3856
55	674.4702	673.4629
56	675.6754	674.6681
57	675.6757	674.6684
58	675.6760	674.6687
59	675.6778	674.6705
60	675.6780	674.6707
61	676.4074	675.4002
62	677.6839	676.6766
63	696.4395	695.4322
64	696.4403	695.4330
65	717.6254	716.6181
66	717.6261	716.6189
67	752.5475	751.5402
68	752.5480	751.5407
69	764.6402	763.6329
70	775.4150	774.4078
75	412.7550	823.4955
82	419.7075	837.4004
90	444.1032	886.1918
91	446.2757	890.5368
92	447.1173	892.2200
94	454.3487	906.6829
102	934.8788	933.8716
103	934.8801	933.8728
131	1038.2681	1037.2609
	549.7443	1097.4740
	559.7117	1117.4089
	563.7882	1125.5618
	569.3342	1136.6538
	569.8351	1137.6556
	581.3192	1160.6239
	591.3450	1180.6754
	595.7404	1189.4662
	596.2151	1190.4157
	604.8470	1207.6795
	608.2578	1214.5010
	608.2586	1214.5026
	1216.0386	1215.0313
	644.7266	1287.4386
	646.3224	1290.6302
	658.2206	1314.4267
	447.1560	1338.4463
	447.1565	1338.4476
	467.1910	1398.5512
	718.8093	1435.6039
	721.1681	1440.3216
	746.2958	1490.5771
	506.3173	1515.9301
	545.8758	1634.6055
	827.3381	1652.6617
	848.2857	1694.5569
	587.6528	1759.9367
	591.3683	1771.0830
	607.2400	1818.6980
	609.9449	1826.8128
	926.4716	1850.9287
	622.3644	1864.0713
	949.4472	1896.8798
	971.3784	1940.7422
	682.9684	2045.8835
	687.9578	2060.8515
	688.6257	2062.8553
	691.9719	2072.8938
	691.9724	2072.8953
	433.3554	2161.7407
	724.6616	2170.9629
	725.0199	2172.0378
	737.5059	2209.4959
	444.1196	2215.5618
	755.3492	2263.0257
431	1142.0984	2282.1823

434	1142.0993	2282.1841
	773.3289	2316.9649
	779.3721	2335.0946
	779.3735	2335.0986
	779.6803	2336.0192
	784.7054	2351.0943
	588.7809	2351.0943
450	1192.4839	2382.9532
451	1192.4846	2382.9546
	608.3763	2429.4763
	822.3609	2464.0608
	500.3835	2496.8812
	500.8136	2499.0318
	630.4428	2517.7421
	648.3026	2589.1813
	520.3351	2596.6389
	652.4003	2605.5719
	876.3927	2626.1562
	885.7239	2654.1498
	666.3954	2661.5527
	548.3285	2736.6062
	696.4400	2781.7311
	467.1903	2797.0981
	467.1961	2797.1331
497	1426.1724	2850.3303
498	1426.1729	2850.3312
499	1434.1733	2866.3320
	412.1694	2878.1349
	974.0904	2919.2495
	586.7559	2928.7430
	978.1814	2931.5223
	591.3446	2951.6865
	591.3479	2951.7033
514	1032.5299	3094.5680
516	1054.5084	3160.5035
518	1054.5086	3160.5041
	456.2826	3186.9275
	459.2753	3207.8761
522	1075.1036	3222.2890
	819.8087	3275.2056
	412.1713	3289.3122
532	1103.9205	3308.7396
	414.7524	3309.9612
	830.1173	3316.4400
	575.3058	3445.7909
	579.5369	3471.1779
	456.2738	3642.1318
	456.2844	3642.2173
	614.1829	3679.0536
	614.5668	3681.3571
	630.4422	3776.6096
	653.2532	3913.4755
	582.8159	4072.6605
	582.8511	4072.9071
	586.4174	4097.8711
	586.7509	4100.2050
	4111.6695	4110.6622
561	4143.1864	4142.1791
565	1409.2652	4224.7739
566	1409.2666	4224.7779
567	1070.1788	4276.6860
	718.4952	4304.9277
	740.4641	4436.7410
	564.3586	4506.8108
	650.1770	4544.1882
	658.2195	4600.4858
	578.3552	4618.7838
	800.4220	4796.4882
	692.3296	4839.2559
	970.9689	4849.8082
	608.3868	4859.0366
	613.3712	4898.9114
	4944.0541	4943.0468
	632.3926	5051.0826
	641.8606	5126.8268
	5152.5235	5151.5162
	658.2189	5257.6930
	5324.0079	5323.0006
	762.5265	5330.6346
	674.4697	5387.6992
	899.5089	5391.0100
	5425.1407	5424.1334
593	5459.1206	5458.1133
	910.8607	5459.1205
	5492.3051	5491.2978
	696.4477	5563.5233
	5657.2541	5656.2468
	5666.1156	5665.1083
	5683.2588	5682.2515
	5817.9985	5816.9912
	5921.8264	5920.8191
	5927.9441	5926.9368
	5988.5293	5987.5220
	6073.9444	6072.9371
	763.8793	6102.9765
	763.8802	6102.9835

☑	607	*6267.8791*	*6266.8718*
☑	608	*6383.5031*	*6382.4958*
☑	609	*6476.8002*	*6475.7929*
☑	612	*7168.8553*	*7167.8480*
☑	613	*7297.0286*	*7296.0213*
☑	614	*7329.8450*	*7328.8377*
☑	615	*7394.6331*	*7393.6258*
☑	616	*7402.0573*	*7401.0500*
☑	617	*7628.7776*	*7627.7703*
☑	618	*7753.5011*	*7752.4938*
☑	619	*1005.4948*	*8035.9001*
☑	620	*8131.2861*	*8130.2788*
☑	621	*8440.3326*	*8439.3253*
☑	622	*9496.9094*	*9495.9021*

Search Parameters

**Type of search**	**:MS/MS Ion Search**
**Enzyme**	**:Trypsin**
**Fixed modifications**	**:Carbamidomethyl (C)**
**Variable modifications :**	**Oxidation (M)**
**Mass values**	**:Monoisotopic**
**Protein Mass**	**:Unrestricted**
**Peptide Mass Tolerance :**	**± 0.05 Da**
**Fragment Mass Tolerance:**	**± 0.05 Da**
**Max Missed Cleavages**	**:2**
**Instrument type**	**:Default**
**Number of queries**	**:622**

Mascot: http://www.matrixscience.com/

Mascot Search Results

User	: Julian
Email	: julian.langer@mpibp-frankfurt.mpg.de
Search title	: Submitted from 140318 842 ChKrettler 1-10 swissprot full T 0.05Da by Mascot Daemon on CCSW010
MS data file	: D:\Data\1403\140318\140314 842 ChReinhard 3_Tray01-C8_01_17684.d\140314 842 ChReinhard 3_Tray01-C8_01_17684.mgf
Database	: SwissProt 57.15 (515203 sequences; 181334896 residues)
Timestamp	: 19 Mar 2014 at 09:49:22 GMT
Protein hits	: TRYP_PIG Trypsin OS=Sus scrofa PE=1 SV=1
	K2C1_HUMAN Keratin, type II cytoskeletal 1 OS=Homo sapiens GN=KRT1 PE=1 SV=6
	CFAH_HUMAN Complement factor H OS=Homo sapiens GN=CFH PE=1 SV=4
	K22E_HUMAN Keratin, type II cytoskeletal 2 epidermal OS=Homo sapiens GN=KRT2 PE=1 SV=2
	K1C9_HUMAN Keratin, type I cytoskeletal 9 OS=Homo sapiens GN=KRT9 PE=1 SV=3
	K1C10_HUMAN Keratin, type I cytoskeletal 10 OS=Homo sapiens GN=KRT10 PE=1 SV=6
	ALBU_HUMAN Serum albumin OS=Homo sapiens GN=ALB PE=1 SV=2
	CH60_ACTP2 60 kDa chaperonin OS=Actinobacillus pleuropneumoniae serotype 5b (strain L20) GN=groL PE=3 SV=1

Mascot Score Histogram

Ions score is -10*Log(P), where P is the probability that the observed match is a random event.
Individual ions scores > 38 indicate identity or extensive homology (p<0.05).
Protein scores are derived from ions scores as a non-probabilistic basis for ranking protein hits.

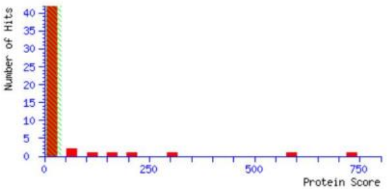

Peptide Summary Report

Format As: Peptide Summary Help

Significance threshold p<0.05 Max. number of hits AUTO

Standard scoring MudPIT scoring Ions score or expect cut-off 0 Show sub-sets 0

Show pop-ups Suppress pop-ups Sort unassigned Decreasing Score Require bold red

Select All Select None Search Selected **Error tolerant** Archive Report

1. TRYP_PIG **Mass:** 25078 **Score:** 732 **Matches:** 33(30) **Sequences:** 9(9) **emPAI:** 3.90
Trypsin OS=Sus scrofa PE=1 SV=1
Check to include this hit in error tolerant search or archive report

Query	Observed	Mr(expt)	Mr(calc)	Delta	Miss	Score	Expect	Rank	Unique	Peptide
88	421.7594	841.5042	841.5022	0.0020	0	54	0.0029	1	U	R.VATVSLPR.S
90	421.7595	841.5045	841.5022	0.0024	0	(49)	0.0096	1	U	R.VATVSLPR.S
91	842.5118	841.5046	841.5022	0.0024	0	(15)	20	1	U	R.VATVSLPR.S
92	421.7596	841.5047	841.5022	0.0025	0	(54)	0.003	1	U	R.VATVSLPR.S
108	523.2865	1044.5585	1044.5564	0.0021	0	65	0.00035	1	U	K.LSSPATLNSR.V
109	523.2866	1044.5587	1044.5564	0.0024	0	(55)	0.003	1	U	K.LSSPATLNSR.V
110	523.2870	1044.5595	1044.5564	0.0031	0	(45)	0.034	1	U	K.LSSPATLNSR.V
111	523.2871	1044.5597	1044.5564	0.0034	0	(56)	0.0024	1	U	K.LSSPATLNSR.V
112	523.2875	1044.5604	1044.5564	0.0040	0	(55)	0.003	1	U	K.LSSPATLNSR.V
113	523.2875	1044.5604	1044.5564	0.0041	0	(53)	0.0044	1	U	K.LSSPATLNSR.V
114	523.2876	1044.5606	1044.5564	0.0043	0	(50)	0.01	1	U	K.LSSPATLNSR.V
116	532.2567	1062.4988	1062.5016	-0.0027	0	94	2.2e-007	1	U	K.APVLSDSSCK.S
156	763.8822	1525.7499	1525.7446	0.0053	0	65	0.00013	1	U	K.SSGSSYPSLLQCLK.A
174	941.9272	1881.8399	1881.8349	0.0050	0	(84)	5.8e-007	1	U	R.SCAAAGTECLISGWGNTK.S
175	941.9279	1881.8412	1881.8349	0.0063	0	116	4e-010	1	U	R.SCAAAGTECLISGWGNTK.S
190	737.7085	2210.1036	2210.0967	0.0069	0	(82)	2.7e-006	1	U	R.LGEHNIDVLEGNEQFINAAK.I
192	737.7093	2210.1060	2210.0967	0.0092	0	(106)	9.3e-009	1	U	R.LGEHNIDVLEGNEQFINAAK.I
193	737.7094	2210.1064	2210.0967	0.0097	0	119	4.5e-010	1	U	R.LGEHNIDVLEGNEQFINAAK.I
198	761.7347	2282.1822	2282.1729	0.0094	0	(52)	0.0021	1	U	K.IITHPNFNGNTLDNDIMLIK.L
200	761.7351	2282.1835	2282.1729	0.0107	0	(63)	0.00018	1	U	K.IITHPNFNGNTLDNDIMLIK.L
201	761.7354	2282.1845	2282.1729	0.0116	0	(48)	0.0054	1	U	K.IITHPNFNGNTLDNDIMLIK.L
206	767.0663	2298.1772	2298.1678	0.0094	0	(27)	0.71	1	U	K.IITHPNFNGNTLDNDIMLIK.L + Oxidation (M)
207	767.0669	2298.1788	2298.1678	0.0110	0	(41)	0.027	1	U	K.IITHPNFNGNTLDNDIMLIK.L + Oxidation (M)
208	767.0675	2298.1806	2298.1678	0.0128	0	67	7.9e-005	1	U	K.IITHPNFNGNTLDNDIMLIK.L + Oxidation (M)
226	903.1476	2706.4211	2706.4089	0.0122	1	(44)	0.0095	1	U	R.IQVRLGEHNIDVLEGNEQFINAAK.I
227	677.6126	2706.4211	2706.4089	0.0122	1	(57)	0.0005	1	U	R.IQVRLGEHNIDVLEGNEQFINAAK.I
228	677.6129	2706.4224	2706.4089	0.0135	1	60	0.00023	1	U	R.IQVRLGEHNIDVLEGNEQFINAAK.I
229	903.1481	2706.4224	2706.4089	0.0135	1	(31)	0.2	1	U	R.IQVRLGEHNIDVLEGNEQFINAAK.I
246	828.1906	3308.7334	3308.7187	0.0147	1	92	1.1e-007	1	U	K.IITHPNFNGNTLDNDIMLIKLSSPATLNSR.V
248	828.1913	3308.7362	3308.7187	0.0175	1	(69)	2.1e-005	1	U	K.IITHPNFNGNTLDNDIMLIKLSSPATLNSR.V
249	828.1914	3308.7366	3308.7187	0.0179	1	(64)	7.2e-005	1	U	K.IITHPNFNGNTLDNDIMLIKLSSPATLNSR.V
251	832.1900	3324.7310	3324.7136	0.0174	1	(41)	0.016	1	U	K.IITHPNFNGNTLDNDIMLIKLSSPATLNSR.V + Oxidation (M)
252	832.1901	3324.7313	3324.7136	0.0177	1	(52)	0.0011	1	U	K.IITHPNFNGNTLDNDIMLIKLSSPATLNSR.V + Oxidation (M)

2. K2C1_HUMAN **Mass:** 66170 **Score:** 590 **Matches:** 18(17) **Sequences:** 9(9) **emPAI:** 0.58
Keratin, type II cytoskeletal 1 OS=Homo sapiens GN=KRT1 PE=1 SV=6
Check to include this hit in error tolerant search or archive report

Query	Observed	Mr(expt)	Mr(calc)	Delta	Miss	Score	Expect	Rank	Unique	Peptide
102	487.2703	972.5261	972.5240	0.0022	0	44	0.043	1		K.IEISELNR.V
103	487.2707	972.5269	972.5240	0.0030	0	(43)	0.047	1		K.IEISELNR.V
104	487.2709	972.5273	972.5240	0.0033	0	(32)	0.66	1		K.IEISELNR.V
134	639.3607	1276.7069	1276.7027	0.0042	0	77	1e-005	1	U	K.LALDLEIATYR.T
135	639.3609	1276.7071	1276.7027	0.0045	0	(65)	0.00018	1	U	K.LALDLEIATYR.T
136	651.8636	1301.7126	1301.7078	0.0048	0	60	0.00065	1		R.SLDLDSIIAEVK.A
137	651.8638	1301.7130	1301.7078	0.0052	0	(49)	0.0093	1		R.SLDLDSIIAEVK.A
149	738.3968	1474.7791	1474.7780	0.0011	0	(70)	6.6e-005	1		R.FLEQQNQVLQTK.W
150	738.3976	1474.7807	1474.7780	0.0027	0	82	4.2e-006	1		R.FLEQQNQVLQTK.W
151	738.3981	1474.7816	1474.7780	0.0037	0	(78)	1.1e-005	1		R.FLEQQNQVLQTK.W
159	800.4229	1598.8313	1598.8264	0.0049	1	63	0.00025	1	U	K.NKLNDLEDALQQAK.E
166	858.9337	1715.8528	1715.8438	0.0090	0	95	1.6e-007	1	U	K.QISNLQQSISDAEQR.G
176	648.0037	1940.9892	1940.9803	0.0089	1	42	0.028	1	U	K.LNDLEDALQQAKEDLAR.L
186	728.7162	2183.1267	2183.1182	0.0085	2	(56)	0.001	1	U	K.NKLNDLEDALQQAKEDLAR.L
187	728.7164	2183.1273	2183.1182	0.0091	2	78	6.9e-006	1	U	K.NKLNDLEDALQQAKEDLAR.L
188	728.7173	2183.1302	2183.1182	0.0120	2	(46)	0.0094	1	U	K.NKLNDLEDALQQAKEDLAR.L
209	777.0661	2328.1764	2328.1669	0.0095	1	(48)	0.0065	1	U	K.QISNLQQSISDAEQRGENALK.D
210	777.0665	2328.1778	2328.1669	0.0108	1	51	0.0033	1	U	K.QISNLQQSISDAEQRGENALK.D

Proteins matching the same set of peptides:
K2C1_PANTR **Mass:** 65621 **Score:** 590 **Matches:** 18(17) **Sequences:** 9(9)
Keratin, type II cytoskeletal 1 OS=Pan troglodytes GN=KRT1 PE=2 SV=1

3. CFAH_HUMAN **Mass:** 143680 **Score:** 323 **Matches:** 15(9) **Sequences:** 7(4) **emPAI:** 0.18
Complement factor H OS=Homo sapiens GN=CFH PE=1 SV=4
Check to include this hit in error tolerant search or archive report

Query	Observed	Mr(expt)	Mr(calc)	Delta	Miss	Score	Expect	Rank	Unique	Peptide

117	532.2997	1062.5848	1062.5822	0.0026	0	60	0.00081	1	U	K.IDVHLVPDR.K
118	532.2997	1062.5849	1062.5822	0.0027	0	(59)	0.00088	1	U	K.IDVHLVPDR.K
119	532.3001	1062.5856	1062.5822	0.0034	0	(59)	0.00081	1	U	K.IDVHLVPDR.K
120	532.3002	1062.5859	1062.5822	0.0037	0	(48)	0.013	1	U	K.IDVHLVPDR.K
130	593.8002	1185.5858	1185.5819	0.0039	1	19	7	1	U	R.TKNDFTWFK.L
133	631.7948	1261.5750	1261.5761	-0.0011	0	21	2.7	1	U	K.DGWSAQPTCIK.S
138	655.3071	1308.5996	1308.5955	0.0041	0	38	0.053	1	U	K.SCDIPVFMNAR.T
139	663.3033	1324.5921	1324.5904	0.0017	0	(18)	3.8	1	U	K.SCDIPVFMNAR.T + Oxidation (M)
143	698.3978	1394.7811	1394.7769	0.0042	0	51	0.003	1	U	K.SSNLIILEEHLK.N
144	465.9343	1394.7811	1394.7769	0.0042	0	(20)	4.5	1	U	K.SSNLIILEEHLK.N
145	698.3983	1394.7821	1394.7769	0.0052	0	(47)	0.0077	1	U	K.SSNLIILEEHLK.N
146	698.3988	1394.7831	1394.7769	0.0062	0	(50)	0.0044	1	U	K.SSNLIILEEHLK.N
153	741.3607	1480.7069	1480.7021	0.0049	0	85	1.6e-006	1	U	K.CFEGFGIDGPAIAK.C
177	1010.9948	2019.9750	2019.9684	0.0066	0	(37)	0.069	1	U	R.DTSCVNPPTVQNAYIVSR.Q
178	1010.9961	2019.9777	2019.9684	0.0093	0	51	0.0029	1	U	R.DTSCVNPPTVQNAYIVSR.Q

4. K22E_HUMAN **Mass:** 65678 **Score:** 187 **Matches:** 8(7) **Sequences:** 3(3) **emPAI:** 0.17

Keratin, type II cytoskeletal 2 epidermal OS=Homo sapiens GN=KRT2 PE=1 SV=2

Check to include this hit in error tolerant search or archive report

Query	Observed	Mr(expt)	Mr(calc)	Delta	Miss	Score	Expect	Rank	Unique	Peptide
102	487.2703	972.5261	972.5240	0.0022	0	44	0.043	1		K.IEISELNR.V
103	487.2707	972.5269	972.5240	0.0030	0	(43)	0.047	1		K.IEISELNR.V
104	487.2709	972.5273	972.5240	0.0033	0	(32)	0.66	1		K.IEISELNR.V
140	665.3682	1328.7219	1328.7187	0.0031	0	61	0.00053	1		R.NLDLDSIIAEVK.A
141	665.3694	1328.7241	1328.7187	0.0054	0	(49)	0.0085	1		R.NLDLDSIIAEVK.A
149	738.3968	1474.7791	1474.7780	0.0011	0	(70)	6.6e-005	1		R.FLEQQNQVLQTK.W
150	738.3976	1474.7807	1474.7780	0.0027	0	82	4.2e-006	1		R.FLEQQNQVLQTK.W
151	738.3981	1474.7816	1474.7780	0.0037	0	(78)	1.1e-005	1		R.FLEQQNQVLQTK.W

5. K1C9_HUMAN **Mass:** 62255 **Score:** 138 **Matches:** 6(4) **Sequences:** 3(2) **emPAI:** 0.18

Keratin, type I cytoskeletal 9 OS=Homo sapiens GN=KRT9 PE=1 SV=3

Check to include this hit in error tolerant search or archive report

Query	Observed	Mr(expt)	Mr(calc)	Delta	Miss	Score	Expect	Rank	Unique	Peptide
115	530.7871	1059.5597	1059.5560	0.0037	0	46	0.021	1	U	K.TLLDIDNTR.M
124	579.3006	1156.5867	1156.5836	0.0030	0	(55)	0.0019	1	U	R.QGVDADINGLR.Q
125	579.3007	1156.5868	1156.5836	0.0031	0	(54)	0.0021	1	U	R.QGVDADINGLR.Q
126	579.3010	1156.5874	1156.5836	0.0038	0	60	0.00054	1	U	R.QGVDADINGLR.Q
243	1088.8476	3263.5210	3263.5066	0.0145	0	31	0.094	1	U	K.DIENQYETQITQIEHEVSSSGQEVQSSAK.E
244	1088.8488	3263.5244	3263.5066	0.0179	0	(22)	0.83	1	U	K.DIENQYETQITQIEHEVSSSGQEVQSSAK.E

6. K1C10_HUMAN **Mass:** 59020 **Score:** 112 **Matches:** 3(2) **Sequences:** 2(1) **emPAI:** 0.12

Keratin, type I cytoskeletal 10 OS=Homo sapiens GN=KRT10 PE=1 SV=6

Check to include this hit in error tolerant search or archive report

Query	Observed	Mr(expt)	Mr(calc)	Delta	Miss	Score	Expect	Rank	Unique	Peptide
106	516.3041	1030.5936	1030.5910	0.0026	0	35	0.23	1	U	R.VLDELTLTK.A
171	899.0122	1796.0098	1796.0043	0.0055	0	(39)	0.034	1	U	R.NVQALEIELQSQLALK.Q
172	899.0135	1796.0124	1796.0043	0.0081	0	77	5e-006	1	U	R.NVQALEIELQSQLALK.Q

7. ALBU_HUMAN **Mass:** 71317 **Score:** 74 **Matches:** 4(1) **Sequences:** 3(1) **emPAI:** 0.10

Serum albumin OS=Homo sapiens GN=ALB PE=1 SV=2

Check to include this hit in error tolerant search or archive report

Query	Observed	Mr(expt)	Mr(calc)	Delta	Miss	Score	Expect	Rank	Unique	Peptide
105	500.8024	999.5903	999.5964	-0.0061	0	6	1.9e+002	1	U	K.QTALVELVK.H
123	575.3114	1148.6082	1148.6077	0.0005	0	47	0.014	1	U	K.LVNEVTEFAK.T
179	682.3727	2044.0963	2044.0881	0.0082	0	(15)	11	1	U	K.VFDEFKPLVEEPQNLIK.Q
180	682.3728	2044.0967	2044.0881	0.0086	0	21	2.8	1	U	K.VFDEFKPLVEEPQNLIK.Q

Proteins matching the same set of peptides:

ALBU_PONAB **Mass:** 71317 **Score:** 74 **Matches:** 4(1) **Sequences:** 3(1)

Serum albumin OS=Pongo abelii GN=ALB PE=2 SV=1

8. CH60_ACTP2 **Mass:** 57781 **Score:** 63 **Matches:** 2(2) **Sequences:** 1(1) **emPAI:** 0.06

60 kDa chaperonin OS=Actinobacillus pleuropneumoniae serotype 5b (strain L20) GN=groL PE=3 SV=1

Check to include this hit in error tolerant search or archive report

Query	Observed	Mr(expt)	Mr(calc)	Delta	Miss	Score	Expect	Rank	Unique	Peptide
230	913.8403	2738.4991	2738.4888	0.0103	0	63	7.6e-005	1	U	K.AGKPLLIVAEDIEGEALATLVVNTMR.G + Oxidation (M)
231	913.8411	2738.5016	2738.4888	0.0128	0	(52)	0.00093	1	U	K.AGKPLLIVAEDIEGEALATLVVNTMR.G + Oxidation (M)

Proteins matching the same set of peptides:

CH60_ACTP7 **Mass:** 57726 **Score:** 63 **Matches:** 2(2) **Sequences:** 1(1)

60 kDa chaperonin OS=Actinobacillus pleuropneumoniae serotype 7 (strain AP76) GN=groL PE=3 SV=1

CH60_ACTPJ **Mass:** 57726 **Score:** 63 **Matches:** 2(2) **Sequences:** 1(1)

60 kDa chaperonin OS=Actinobacillus pleuropneumoniae serotype 3 (strain JL03) GN=groL PE=3 SV=1

CH60_ACTPL **Mass:** 57722 **Score:** 63 **Matches:** 2(2) **Sequences:** 1(1)

60 kDa chaperonin OS=Actinobacillus pleuropneumoniae GN=groL PE=3 SV=3

Peptide matches not assigned to protein hits: (no details means no match)

Query	Observed	Mr(expt)	Mr(calc)	Delta	Miss	Score	Expect	Rank	Unique	Peptide
122	558.8063	1115.5981	1115.6339	-0.0358	1	35	0.26	1		KTVSWAVTPK
127	581.3180	1160.6215	1160.6223	-0.0008	0	33	0.47	1		NQVSLTCLVK
128	581.3200	1160.6254	1160.6401	-0.0147	2	22	5	1		NKVSLSKEEK
132	613.8008	1225.5871	1225.6134	-0.0263	1	22	2.9	1		FICRMMLAGK
170	887.9561	1773.8976	1773.8857	0.0120	2	15	18	1		GRVEELGEEIGRESSK
86	802.4329	801.4256	801.4093	0.0163	0	15	48	1		EASSRPR
95	846.4454	845.4381	845.4607	-0.0226	0	12	75	1		LTTVAADR
241	532.2578	3187.5034	3187.5109	-0.0075	0	10	14	1		TTDYAMLVTMFGWFTMVISYVVYYVR + 2 Oxidation (M)
97	432.2389	862.4632	862.4331	0.0302	0	10	94	1		NMAGSLVR + Oxidation (M)
147	702.3095	1402.6045	1402.6212	-0.0167	0	8	17	1		SESTNETEIHEK
85	802.4327	801.4254	801.4345	-0.0091	0	8	2.2e+002	1		TSVAPTAR
42	540.2999	539.2926	539.3067	-0.0141	0	8	74	1		PQAPK
290	593.8207	4742.5072	4742.5550	-0.0478	2	7	6.4	1		AAVKPYPFYVKKASGATLVTVDGVELIDYVLGYGPLILGHMHPK + Oxidation (M)
163	826.8315	1651.6484	1651.6645	-0.0161	0	6	3.5	1		ACSSHSGEEGGFGSAGR
168	883.8985	1765.7824	1765.8318	-0.0494	1	6	34	1		SNDPKSQNHPGWLMR
96	846.4454	845.4381	845.4130	0.0251	1	6	2.8e+002	1		EPSEEKK
41	534.3148	533.3075	533.3173	-0.0098	2	6	2.1e+002	1		KKTTG
238	530.7833	3178.6561	3178.6339	0.0223	1	6	52	1		GFFGEHGGLFVSETLIPALQELADAYKAAK
165	555.2480	1662.7223	1662.7640	-0.0417	2	6	27	1		VGCMVGTSRYMSRK + 2 Oxidation (M)
204	762.0633	2283.1682	2283.1470	0.0212	0	5	1.1e+002	1		LQAIEPSGNYHILHAYCGIK
162	826.8306	1651.6466	1651.6645	-0.0179	0	5	4.1	1		ACSSHSGEEGGFGSAGR
169	883.8989	1765.7832	1765.7723	0.0109	1	5	51	1		DAVQNCCGISKTEER
269	655.3027	3925.7725	3925.7299	0.0427	2	4	15	1		EQGYDVIGIFMKNWDDTDENGVCTATEDYKDVAK
167	883.8983	1765.7820	1765.8226	-0.0406	1	4	54	1		KMEQESIDQLCELK + Oxidation (M)
259	914.1791	3652.6875	3652.7072	-0.0198	1	4	30	1		TTMTLQAIAQCQKAGGTCAFIDAEHALDPQYAR + Oxidation (M)
321	7706.7262	7705.7189	7705.7641	-0.0451	0	4	4	1		GPMVTVDYAYYLCFCILAITTATTIIYGSYYPVVLTSGFISCITYTWSIDNANVQIMFYGLIPVWGK + 2 Oxidation (M)
233	575.3127	2871.5273	2871.4899	0.0373	2	4	91	1		GVNPKVVEILIDSLNSEKVSVEDCTK
217	802.4317	2404.2732	2404.3226	-0.0494	2	3	1.4e+002	1		LLQAIEAVPRERFVDEALAHK
256	575.3221	3445.8888	3445.8470	0.0418	2	3	27	1		ALLEPLGAGDGVRVDFLEVAPENWIGIGGRLGR
268	651.8617	3905.1267	3905.1313	-0.0046	1	3	22	1		EAGVPLLAYGSFITVLINFLILAFIIFMMVKGMNK + 2 Oxidation (M)
298	665.3631	5314.8468	5314.8248	0.0220	1	3	11	1		GRPLFPGTSTLHQLELILETIPPPSEEDLLALGSGCRASVLHQLGSRPR
257	500.8073	3498.6004	3498.6449	-0.0445	1	3	36	1		DLIAEHEVDCWLVNTGWTGGAYGTGHRMPIK + Oxidation (M)

316	6328.0751	6327.0678	6327.0804	-0.0126	2	3	17	1	GIIGNLDHAFLINVSYDDCSPNAPNIPAAAYAFFMMMFANITPLLMTGAFAERVKFK + 2 Oxidation (M)
185	430.2417	2146.1720	2146.2011	-0.0291	2	3	1.5e+002	1	QSGIPKVPAIPRHSNFTGLK
203	762.0632	2283.1679	2283.1706	-0.0027	1	2	2.2e+002	1	EALRDSAIPADAIQLLEDTSR
94	846.4451	845.4378	845.4494	-0.0116	1	2	7e+002	1	AIEEEKK
279	692.3365	4147.9751	4147.9931	-0.0180	1	2	57	1	ILACKGMDISDTYSEFISSILSALFFASEELTSMLDK + Oxidation (M)
285	738.3927	4424.3126	4424.2979	0.0147	2	2	45	1	LMQKMTIMAFIGNFAESIQMLTPQLHAIIAASVSIKSSQK + 3 Oxidation (M)
157	515.9397	1544.7972	1544.8021	-0.0048	2	2	4e+002	1	KMWDELPEVVRK + Oxidation (M)
295	639.3576	5106.8025	5106.7540	0.0486	2	2	6.8	1	LDGEIRISGAKNSALPILAATLLADGPVTVQNLPHLHDITTMIELFGR + Oxidation (M)
276	824.6312	4118.1198	4118.1562	-0.0364	0	1	72	1	LLLGQSLYVEASVISSDAGEIENSILDDIPIVASPYSIK
287	655.3088	4580.1103	4580.1536	-0.0433	2	1	33	1	LPHCSAAPSHQDHSAMGFGPELWCPKGHTELLRLQDSELR + Oxidation (M)
291	4746.4014	4745.3941	4745.3793	0.0149	2	1	65	1	AVAVAVTVDATDAFLDEIVSAMQPDMLQLHGSEHPERVAELKAR + Oxidation (M)
303	5738.0792	5737.0719	5737.0694	0.0025	2	0	16	1	ISMLMSIPTIIASGVLLGADVAVTSDAQAARDGAIAAAFAFVSALLALSLMMRLLR + 4 Oxidation (M)
292	692.3424	4839.3455	4839.3095	0.0360	2	0	71	1	MDMNRVKQIVSSPADIPVYYNGVSVWIDGYDEENQMATVHLR
1	402.3574	401.3501							
2	413.2665	412.2592							
3	413.2669	412.2596							
4	415.2128	414.2055							
5	415.2131	414.2058							
6	415.2132	414.2059							
7	415.2545	414.2472							
8	415.3424	414.3351							
9	415.3425	414.3352							
10	430.2446	429.2373							
11	430.2449	429.2376							
12	430.3888	429.3815							
13	432.2394	431.2321							
14	432.2395	431.2322							
15	432.2810	431.2737							
16	432.2812	431.2740							
17	432.2819	431.2747							
18	442.3314	441.3242							
19	444.1172	443.1099							
20	444.1180	443.1107							
21	446.2611	445.2538							
22	447.2916	446.2843							
23	453.3446	452.3373							
24	456.2808	455.2736							
25	459.2810	458.2737							
26	467.0942	466.0870							
27	475.2310	474.2237							
28	476.3073	475.3001							
29	476.3081	475.3008							
30	476.3083	475.3010							
31	500.3072	499.2999							
32	500.3076	499.3003							
33	500.3078	499.3005							
34	500.8047	499.7975							
35	506.3167	505.3095							
36	520.3341	519.3268							
37	520.3341	519.3268							
38	520.3343	519.3270							
39	520.3347	519.3274							
40	528.3660	527.3588							
43	544.3343	543.3270							
44	547.3335	546.3262							
45	550.6299	549.6226							
46	564.3606	563.3533							
47	564.3607	563.3534							
48	564.3609	563.3536							
49	564.3609	563.3537							
50	579.5344	578.5272							
51	579.5349	578.5276							
52	579.5366	578.5293							
53	579.5368	578.5295							
54	586.4176	585.4103							
55	591.3608	590.3536							
56	591.5717	590.5644							
57	594.3689	593.3617							
58	608.3862	607.3789							
59	608.3866	607.3793							
60	608.3871	607.3798							
61	614.5703	613.5631							
62	614.5713	613.5640							
63	615.4044	614.3971							
64	615.4051	614.3978							
65	615.4056	614.3983							
66	617.4080	616.4007							
67	622.3662	621.3590							
68	630.4439	629.4366							
69	630.4450	629.4377							
70	632.3871	631.3798							
71	652.4135	651.4063							
72	674.4690	673.4617							
73	674.4702	673.4630							
74	675.6742	674.6670							
75	675.6744	674.6671							
76	675.6770	674.6697							
77	675.6779	674.6706							
78	675.6779	674.6706							
79	675.6782	674.6709							
80	677.6846	676.6774							
81	677.6848	676.6775							
82	718.4981	717.4908							
83	752.5478	751.5405							
84	752.5484	751.5411							
87	412.1717	822.3288							
89	842.5114	841.5042							
93	842.5125	841.5052							
98	448.3545	894.6944							
99	456.2804	910.5463							
100	934.8786	933.8713							
101	934.8815	933.8742							
107	517.2028	1032.3911							
121	555.3482	1108.6819							
129	591.3499	1180.6853							
131	1216.0437	1215.0364							
142	678.3208	1354.6270							

148	721.1677	1440.3209
152	740.1742	1478.3338
154	743.7431	1485.4717
155	754.2789	1506.5432
158	533.3443	1597.0112
160	402.3576	1605.4013
161	550.3422	1648.0047
164	555.2331	1662.6775
173	607.2403	1818.6992
181	415.2542	2071.2344
182	520.3176	2077.2412
183	696.4320	2086.2741
184	525.3248	2097.2700
189	546.9819	2183.8984
191	1106.0603	2210.1059
194	755.3473	2263.0201
195	755.3482	2263.0229
196	566.7630	2263.0229
197	456.2809	2276.3681
199	1142.0984	2282.1822
202	1142.0995	2282.1845
205	763.8636	2288.5689
211	779.3705	2335.0898
212	779.3713	2335.0922
213	779.6754	2336.0045
214	1169.0104	2336.0062
215	779.6760	2336.0063
216	797.3138	2388.9197
218	614.5514	2454.1765
219	412.7522	2470.4693
220	619.5151	2474.0312
221	622.4061	2485.5954
222	516.3053	2576.4902
223	650.2040	2596.7867
224	652.4135	2605.6251
225	652.4145	2605.6288
232	942.4296	2824.2670
234	412.1703	2878.1410
235	413.2660	2885.8111
236	613.3499	3061.7129
237	444.1144	3101.7501
239	456.2620	3186.7829
240	456.2807	3186.9140
242	539.3550	3230.0865
245	412.7557	3293.9872
247	1103.9193	3308.7362
250	1103.9195	3308.7366
253	841.4198	3361.6501
254	564.3545	3380.0835
255	490.2780	3424.8949
258	591.3438	3542.0193
260	611.1658	3660.9510
261	613.3568	3674.0969
262	3705.7018	3704.6945
263	530.7839	3708.4362
264	632.3834	3788.2570
265	633.3391	3793.9910
266	763.8782	3814.3549
267	643.4019	3854.3679
270	503.3087	4018.4118
271	582.8200	4072.6891
272	582.8227	4072.7080
273	680.7944	4078.7224
274	4111.6013	4110.5940
275	588.3088	4111.1107
277	591.3154	4132.1571
278	4143.3234	4142.3161
280	702.3094	4207.8127
281	604.8689	4227.0312
282	539.0868	4304.6360
283	718.4969	4304.9377
284	547.3165	4370.4737
286	643.4306	4496.9630
288	4582.7331	4581.7258
289	579.5347	4628.2195
293	613.6544	4901.1773
294	721.1632	5041.0914
296	5228.3655	5227.3582
297	659.1649	5265.2609
299	691.6078	5524.8044
300	800.4199	5595.8881
301	5683.1304	5682.1231
302	5694.6460	5693.6387
304	5763.3154	5762.3081
305	730.9056	5839.1864
306	983.4824	5894.8509
307	5921.8290	5920.8217
308	5923.4089	5922.4016
309	5923.4202	5922.4129
310	5928.2144	5927.2071
311	858.9308	6005.4644
312	754.2790	6026.1739
313	6037.6235	6036.6162
314	6251.7697	6250.7624
315	6258.8918	6257.8845
317	6954.2853	6953.2780
318	7113.5168	7112.5095
319	7244.5542	7243.5469
320	7394.6099	7393.6026
322	7921.7952	7920.7879
323	7960.6714	7959.6641
324	8082.5252	8081.5179
325	10265.3498	10264.3425
326	11476.0369	11475.0296

Search Parameters

```
Type of search         : MS/MS Ion Search
Enzyme                 : Trypsin
Fixed modifications    : Carbamidomethyl (C)
Variable modifications : Oxidation (M)
Mass values            : Monoisotopic
Protein Mass           : Unrestricted
Peptide Mass Tolerance : ± 0.05 Da
Fragment Mass Tolerance: ± 0.05 Da
Max Missed Cleavages   : 2
Instrument type        : Default
Number of queries      : 326
```

Mascot: http://www.matrixscience.com/

Mascot Search Results

```
User          : Julian
Email         : julian.langer@mpibp-frankfurt.mpg.de
Search title  : Submitted from 140318 842 ChKrettler  1-10 swissprot full T 0.05Da by Mascot Daemon on CCSW010
MS data file  : D:\Data\1403\140318\140314 842 ChReinhard 4_Tray01-C9_01_17685.d\140314 842 ChReinhard 4_Tray01-C9_01_17685.mgf
Database      : SwissProt 57.15 (515203 sequences; 181334896 residues)
Timestamp     : 19 Mar 2014 at 09:49:55 GMT
Protein hits  : F13A_HUMAN  Coagulation factor XIII A chain OS=Homo sapiens GN=F13A1 PE=1 SV=4
                F13B_HUMAN  Coagulation factor XIII B chain OS=Homo sapiens GN=F13B PE=1 SV=3
                F13A_RAT    Coagulation factor XIII A chain OS=Rattus norvegicus GN=F13a1 PE=2 SV=3
                TRYP_PIG    Trypsin OS=Sus scrofa PE=1 SV=1
                K2C1_HUMAN  Keratin, type II cytoskeletal 1 OS=Homo sapiens GN=KRT1 PE=1 SV=6
                F13A_BOVIN  Coagulation factor XIII A chain (Fragment) OS=Bos taurus GN=F13A1 PE=1 SV=2
                K1C9_HUMAN  Keratin, type I cytoskeletal 9 OS=Homo sapiens GN=KRT9 PE=1 SV=3
                K22E_HUMAN  Keratin, type II cytoskeletal 2 epidermal OS=Homo sapiens GN=KRT2 PE=1 SV=2
                ISPH_SYNS3  4-hydroxy-3-methylbut-2-enyl diphosphate reductase OS=Synechococcus sp. (strain CC9311) GN=ispH PE=3 SV=1
```

Mascot Score Histogram

Ions score is -10*Log(P), where P is the probability that the observed match is a random event.
Individual ions scores > 38 indicate identity or extensive homology (p<0.05).
Protein scores are derived from ions scores as a non-probabilistic basis for ranking protein hits.

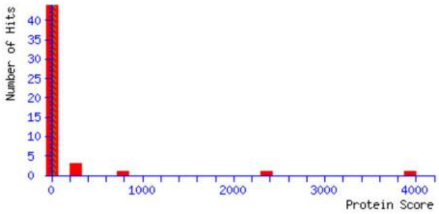

Peptide Summary Report

Format As: Peptide Summary — Help

Significance threshold p< 0.05 — Max. number of hits AUTO

Standard scoring — MudPIT scoring — Ions score or expect cut-off 0 — Show sub-sets 0

Show pop-ups — Suppress pop-ups — Sort unassigned Decreasing Score — Require bold red

Select All — Select None — Search Selected — Error tolerant — Archive Report

1. F13A_HUMAN **Mass:** 83728 **Score:** 3932 **Matches:** 117(91) **Sequences:** 36(29) **emPAI:** 5.15
   Coagulation factor XIII A chain OS=Homo sapiens GN=F13A1 PE=1 SV=4
   Check to include this hit in error tolerant search or archive report

Query	Observed	Mr(expt)	Mr(calc)	Delta	Miss	Score	Expect	Rank	Unique	Peptide
119	418.2390	834.4634	834.4599	0.0034	0	(11)	63	3		R.VEYVIGR.Y
120	418.2390	834.4635	834.4599	0.0036	0	32	0.56	1		R.VEYVIGR.Y
125	430.2476	858.4806	858.4811	-0.0004	0	(58)	0.002	1	U	R.LSIQSSPK.C
126	430.2493	858.4841	858.4811	0.0030	0	63	0.00066	1	U	R.LSIQSSPK.C
127	430.2493	858.4841	858.4811	0.0030	0	(49)	0.016	1	U	R.LSIQSSPK.C
128	430.2494	858.4843	858.4811	0.0033	0	(61)	0.001	1	U	R.LSIQSSPK.C
129	430.2513	858.4879	858.4811	0.0069	0	(54)	0.0048	1	U	R.LSIQSSPK.C
131	438.7282	875.4419	875.4535	-0.0116	0	(35)	0.3	1		R.VGSAMVNAK.D
132	438.7342	875.4539	875.4535	0.0004	0	(36)	0.24	1		R.VGSAMVNAK.D
133	438.7346	875.4546	875.4535	0.0011	0	(54)	0.004	1		R.VGSAMVNAK.D
134	438.7347	875.4549	875.4535	0.0015	0	60	0.001	1		R.VGSAMVNAK.D
135	438.7348	875.4550	875.4535	0.0016	0	(56)	0.0023	1		R.VGSAMVNAK.D
136	439.2146	876.4147	876.4123	0.0024	0	(37)	0.14	1	U	R.AQMDLSGR.G
137	439.2148	876.4150	876.4123	0.0027	0	(38)	0.1	1	U	R.AQMDLSGR.G
138	439.2151	876.4157	876.4123	0.0034	0	39	0.084	1	U	R.AQMDLSGR.G
151	515.7723	1029.5301	1029.5277	0.0024	0	(66)	0.0002	1	U	R.CGPASVQAIK.H
152	515.7725	1029.5305	1029.5277	0.0028	0	66	0.00018	1	U	R.CGPASVQAIK.H
153	515.7726	1029.5307	1029.5277	0.0030	0	(63)	0.00041	1	U	R.CGPASVQAIK.H
154	515.7727	1029.5309	1029.5277	0.0032	0	(63)	0.00042	1	U	R.CGPASVQAIK.H
162	558.8085	1115.6024	1115.6009	0.0016	0	(44)	0.038	1	U	K.KPLNTEGVMK.S
163	558.8088	1115.6029	1115.6009	0.0021	0	(62)	0.00059	1	U	K.KPLNTEGVMK.S
164	558.8088	1115.6030	1115.6009	0.0022	0	67	0.00016	1	U	K.KPLNTEGVMK.S
165	558.8092	1115.6038	1115.6009	0.0029	0	(53)	0.004	1	U	K.KPLNTEGVMK.S
177	566.8062	1131.5979	1131.5958	0.0021	0	(25)	2.6	1	U	K.KPLNTEGVMK.S + Oxidation (M)
178	566.8070	1131.5995	1131.5958	0.0037	0	(57)	0.0018	1	U	K.KPLNTEGVMK.S + Oxidation (M)
182	590.3076	1178.6006	1178.5965	0.0041	0	(70)	6.3e-005	1	U	K.LIASMSSDSLR.H
183	590.3080	1178.6014	1178.5965	0.0049	0	81	5.5e-006	1	U	K.LIASMSSDSLR.H
184	598.3049	1194.5952	1194.5914	0.0038	0	(75)	1.9e-005	1	U	K.LIASMSSDSLR.H + Oxidation (M)
185	598.3049	1194.5952	1194.5914	0.0038	0	(75)	1.8e-005	1	U	K.LIASMSSDSLR.H + Oxidation (M)
186	598.3049	1194.5953	1194.5914	0.0039	0	(78)	9.3e-006	1	U	K.LIASMSSDSLR.H + Oxidation (M)
187	598.3051	1194.5956	1194.5914	0.0042	0	(70)	6.3e-005	1	U	K.LIASMSSDSLR.H + Oxidation (M)
213	640.8524	1279.6903	1279.6846	0.0058	0	(78)	9.2e-006	1		R.LALETALMYGAK.K
215	648.8493	1295.6840	1295.6795	0.0045	0	101	4e-008	1		R.LALETALMYGAK.K + Oxidation (M)
216	648.8498	1295.6851	1295.6795	0.0056	0	(96)	1.3e-007	1		R.LALETALMYGAK.K + Oxidation (M)
217	648.8502	1295.6859	1295.6795	0.0064	0	(77)	1.1e-005	1		R.LALETALMYGAK.K + Oxidation (M)
229	441.9041	1322.6904	1322.6864	0.0040	1	(21)	4.7	1	U	R.KLIASMSSDSLR.H + Oxidation (M)
230	441.9045	1322.6915	1322.6864	0.0052	1	38	0.096	1	U	R.KLIASMSSDSLR.H + Oxidation (M)
231	663.9189	1325.8232	1325.8170	0.0062	0	(44)	0.0059	1	U	K.STVLTIPEIIIK.V
232	663.9190	1325.8234	1325.8170	0.0065	0	50	0.0017	1	U	K.STVLTIPEIIIK.V
245	683.8809	1365.7472	1365.7405	0.0067	1	(8)	67	1		R.DLFRVEYVIGR.Y
246	683.8814	1365.7482	1365.7405	0.0078	1	29	0.57	1		R.DLFRVEYVIGR.Y
268	486.2581	1455.7524	1455.7470	0.0054	0	(68)	9.3e-005	1	U	R.HVYGELDVQIQR.R
269	728.8835	1455.7524	1455.7470	0.0054	0	79	6.7e-006	1	U	R.HVYGELDVQIQR.R
270	728.8841	1455.7537	1455.7470	0.0067	0	(58)	0.00076	1	U	R.HVYGELDVQIQR.R
271	486.2585	1455.7537	1455.7470	0.0067	0	(69)	6.3e-005	1	U	R.HVYGELDVQIQR.R
280	763.3956	1524.7767	1524.7712	0.0055	0	82	3.3e-006	1	U	K.ETFDVTLEPLSFK.K
281	763.3957	1524.7769	1524.7712	0.0057	0	(65)	0.00017	1	U	K.ETFDVTLEPLSFK.K
287	785.9112	1569.8079	1569.8014	0.0065	0	64	0.0002	1	U	R.MYVAVWTPYGVLR.T + Oxidation (M)
294	532.2811	1593.8216	1593.8151	0.0065	1	29	0.66	1		R.VEYVIGRYPQENK.G
295	532.2815	1593.8226	1593.8151	0.0075	1	(25)	1.6	1		R.VEYVIGRYPQENK.G
296	822.8686	1643.7227	1643.7171	0.0056	0	(125)	4e-011	1	U	K.QIGGDGMMDITDTYK.F
301	551.9656	1652.8750	1652.8661	0.0089	1	40	0.049	1	U	K.ETFDVTLEPLSFKK.E
307	830.8668	1659.7191	1659.7120	0.0070	0	(123)	5.1e-011	1	U	K.QIGGDGMMDITDTYK.F + Oxidation (M)
308	830.8671	1659.7196	1659.7120	0.0076	0	(115)	3.5e-010	1	U	K.QIGGDGMMDITDTYK.F + Oxidation (M)
309	830.8675	1659.7205	1659.7120	0.0085	0	142	6.7e-013	1	U	K.QIGGDGMMDITDTYK.F + Oxidation (M)

310	838.8633	1675.7120	1675.7070	0.0051	0	(93)	3.6e-008	1	U	K.QIGGDGMMDITDTYK.F + 2 Oxidation (M)
311	838.8643	1675.7141	1675.7070	0.0071	0	(83)	4.4e-007	1	U	K.QIGGDGMMDITDTYK.F + 2 Oxidation (M)
313	844.4696	1686.9247	1686.9192	0.0055	0	(55)	0.0016	1	U	K.GTYIPVPIVSELQSGK.W
314	844.4700	1686.9255	1686.9192	0.0062	0	(67)	0.0001	1	U	K.GTYIPVPIVSELQSGK.W
315	844.4708	1686.9270	1686.9192	0.0077	0	70	4.7e-005	1	U	K.GTYIPVPIVSELQSGK.W
316	564.6187	1690.8343	1690.8275	0.0068	0	(66)	0.00012	1		K.DGTHVVENVDATHIGK.L
317	564.6188	1690.8346	1690.8275	0.0071	0	95	1.8e-007	1		K.DGTHVVENVDATHIGK.L
318	564.6188	1690.8347	1690.8275	0.0072	0	(70)	5.3e-005	1		K.DGTHVVENVDATHIGK.L
319	564.6196	1690.8369	1690.8275	0.0094	0	(53)	0.0026	1		K.DGTHVVENVDATHIGK.L
340	883.9209	1765.8272	1765.8193	0.0079	0	(87)	5.9e-007	1	U	R.SNVDMDFEVENAVLGK.D
346	594.6638	1780.9697	1780.9611	0.0086	2	(37)	0.068	1	U	K.KETFDVTLEPLSFKK.E
347	594.6640	1780.9702	1780.9611	0.0091	2	(47)	0.0078	1	U	K.KETFDVTLEPLSFKK.E
348	594.6644	1780.9714	1780.9611	0.0103	2	62	0.00021	1	U	K.KETFDVTLEPLSFKK.E
349	891.9180	1781.8214	1781.8142	0.0072	0	103	1.3e-008	1	U	R.SNVDMDFEVENAVLGK.D + Oxidation (M)
350	891.9191	1781.8237	1781.8142	0.0095	0	(99)	3.4e-008	1	U	R.SNVDMDFEVENAVLGK.D + Oxidation (M)
356	602.6565	1804.9476	1804.9407	0.0069	0	(43)	0.026	1	U	R.NVWVHLDGPGVTRPMK.K
357	602.6572	1804.9498	1804.9407	0.0092	0	60	0.00057	1	U	R.NVWVHLDGPGVTRPMK.K
363	607.3167	1818.9282	1818.9224	0.0058	1	(69)	7.6e-005	1		K.KDGTHVVENVDATHIGK.L
364	607.3170	1818.9293	1818.9224	0.0068	1	(64)	0.00022	1		K.KDGTHVVENVDATHIGK.L
365	607.3177	1818.9314	1818.9224	0.0089	1	87	1e-006	1		K.KDGTHVVENVDATHIGK.L
367	607.9879	1820.9420	1820.9356	0.0064	0	(24)	1.9	1	U	R.NVWVHLDGPGVTRPMK.K + Oxidation (M)
368	607.9885	1820.9436	1820.9356	0.0080	0	(28)	0.92	1	U	R.NVWVHLDGPGVTRPMK.K + Oxidation (M)
387	649.0249	1944.0530	1944.0469	0.0061	0	(59)	0.00051	1	U	R.GVNLQEFLNVTSVHLFK.E
388	973.0338	1944.0530	1944.0469	0.0061	0	(44)	0.015	1	U	R.GVNLQEFLNVTSVHLFK.E
389	649.0254	1944.0545	1944.0469	0.0076	0	(63)	0.00018	1	U	R.GVNLQEFLNVTSVHLFK.E
390	973.0345	1944.0545	1944.0469	0.0076	0	72	2.6e-005	1	U	R.GVNLQEFLNVTSVHLFK.E
421	725.0118	2172.0136	2172.0045	0.0091	1	(63)	0.00012	1	U	R.SNVDMDFEVENAVLGKDFK.L + Oxidation (M)
422	725.0123	2172.0150	2172.0045	0.0105	1	63	0.00012	1	U	R.SNVDMDFEVENAVLGKDFK.L + Oxidation (M)
441	1165.0869	2328.1593	2328.1525	0.0068	0	178	5.4e-016	1	U	R.EEYVLNDIGVIFYGEVNDIK.T
442	1165.0881	2328.1617	2328.1525	0.0092	0	(83)	1.8e-006	1	U	R.EEYVLNDIGVIFYGEVNDIK.T
455	805.0649	2412.1730	2412.1631	0.0099	1	(53)	0.0016	1		K.FQEGQEEERLALETALMYGAK.K
456	805.0663	2412.1772	2412.1631	0.0141	1	79	3.9e-006	1		K.FQEGQEEERLALETALMYGAK.K
458	810.3970	2428.1691	2428.1580	0.0111	1	(54)	0.001	1		K.FQEGQEEERLALETALMYGAK.K + Oxidation (M)
459	810.3978	2428.1715	2428.1580	0.0136	1	(37)	0.055	1		K.FQEGQEEERLALETALMYGAK.K + Oxidation (M)
460	810.3984	2428.1732	2428.1580	0.0152	1	(48)	0.0041	1		K.FQEGQEEERLALETALMYGAK.K + Oxidation (M)
474	1278.0681	2554.1217	2554.1145	0.0072	0	6	15	1	U	R.SWSYGQFEDGILDTCLYVMDR.A
475	1278.0687	2554.1229	2554.1145	0.0084	0	(4)	25	1	U	R.SWSYGQFEDGILDTCLYVMDR.A
480	1301.6661	2601.3176	2601.3035	0.0142	0	(11)	27	1	U	R.AVPPNNSNAAEDDLPTVELQGVVPR.G
481	868.1132	2601.3177	2601.3035	0.0142	0	(57)	0.00068	1	U	R.AVPPNNSNAAEDDLPTVELQGVVPR.G
482	1301.6664	2601.3183	2601.3035	0.0149	0	(27)	0.63	1	U	R.AVPPNNSNAAEDDLPTVELQGVVPR.G
483	868.1134	2601.3183	2601.3035	0.0149	0	(62)	0.00022	1	U	R.AVPPNNSNAAEDDLPTVELQGVVPR.G
484	868.1135	2601.3186	2601.3035	0.0152	0	78	5.8e-006	1	U	R.AVPPNNSNAAEDDLPTVELQGVVPR.G
485	1301.6666	2601.3186	2601.3035	0.0152	0	(19)	4	1	U	R.AVPPNNSNAAEDDLPTVELQGVVPR.G
488	872.1097	2613.3074	2613.2962	0.0112	1	(44)	0.011	1	U	K.EREEYVLNDIGVIFYGEVNDIK.T
489	872.1107	2613.3103	2613.2962	0.0141	1	(52)	0.002	1	U	K.EREEYVLNDIGVIFYGEVNDIK.T
490	872.1117	2613.3132	2613.2962	0.0170	1	56	0.00088	1	U	K.EREEYVLNDIGVIFYGEVNDIK.T
517	681.3405	2721.3330	2721.3194	0.0136	0	46	0.0073	1	U	R.EIRPNSTVQWEEVCRPWVSGHR.K
518	681.3410	2721.3349	2721.3194	0.0155	0	(36)	0.064	1	U	R.EIRPNSTVQWEEVCRPWVSGHR.K
519	681.3414	2721.3364	2721.3194	0.0170	0	(45)	0.0096	1	U	R.EIRPNSTVQWEEVCRPWVSGHR.K
524	920.1470	2757.4193	2757.4046	0.0147	1	(70)	2.5e-005	1	U	R.RAVPPNNSNAAEDDLPTVELQGVVPR.G
525	920.1473	2757.4202	2757.4046	0.0157	1	(88)	5e-007	1	U	R.RAVPPNNSNAAEDDLPTVELQGVVPR.G
526	920.1476	2757.4208	2757.4046	0.0163	1	96	7.5e-008	1	U	R.RAVPPNNSNAAEDDLPTVELQGVVPR.G
532	1007.8582	3020.5527	3020.5430	0.0097	0	31	0.19	1	U	K.EAVLIQAGEYMGQLLEQASLHFFVTAR.I
533	1531.1794	3060.3443	3060.3335	0.0108	0	(9)	4.6	1	U	R.NPETDTYILFNPWCEDDAVYLDNEK.E
535	1021.1224	3060.3453	3060.3335	0.0118	0	52	0.00024	1	U	R.NPETDTYILFNPWCEDDAVYLDNEK.E
545	1116.1721	3345.4946	3345.4772	0.0174	1	40	0.005	1	U	R.NPETDTYILFNPWCEDDAVYLDNEKER.E
546	1116.1725	3345.4956	3345.4772	0.0185	1	(25)	0.18	1	U	R.NPETDTYILFNPWCEDDAVYLDNEKER.E
547	1138.5301	3412.5685	3412.5517	0.0167	0	(107)	1.5e-009	1	U	R.IVTNYFSAHDNDANLQMDIFLEEDGNVNSK.L
548	1138.5303	3412.5690	3412.5517	0.0173	0	113	4e-010	1	U	R.IVTNYFSAHDNDANLQMDIFLEEDGNVNSK.L
549	1138.5313	3412.5721	3412.5517	0.0203	0	(89)	1e-007	1	U	R.IVTNYFSAHDNDANLQMDIFLEEDGNVNSK.L
551	1143.8627	3428.5661	3428.5467	0.0195	0	(98)	1.4e-008	1	U	R.IVTNYFSAHDNDANLQMDIFLEEDGNVNSK.L + Oxidation (M)
552	1143.8630	3428.5671	3428.5467	0.0204	0	(62)	5.2e-005	1	U	R.IVTNYFSAHDNDANLQMDIFLEEDGNVNSK.L + Oxidation (M)

2\. F13B_HUMAN **Mass:** 77742 **Score:** 2434 **Matches:** 80(62) **Sequences:** 30(26) **emPAI:** 3.57

Coagulation factor XIII B chain OS=Homo sapiens GN=F13B PE=1 SV=3

Check to include this hit in error tolerant search or archive report

Query	Observed	Mr(expt)	Mr(calc)	Delta	Miss	Score	Expect	Rank	Unique	Peptide
94	714.4540	713.4467	713.4435	0.0031	0	36	0.22	1	U	K.NNLLLK.W
111	405.2029	808.3913	808.3902	0.0012	0	54	0.0027	1	U	K.YPLCTR.K
160	552.2847	1102.5549	1102.5515	0.0034	0	(35)	0.24	1	U	K.GMCTSPPLIK.H
161	552.2850	1102.5554	1102.5515	0.0039	0	(42)	0.05	1	U	K.GMCTSPPLIK.H
168	560.2814	1118.5483	1118.5464	0.0019	0	(29)	0.78	1	U	K.GMCTSPPLIK.H + Oxidation (M)
169	560.2822	1118.5498	1118.5464	0.0034	0	(43)	0.034	1	U	K.GMCTSPPLIK.H + Oxidation (M)
170	560.2823	1118.5500	1118.5464	0.0036	0	(34)	0.28	1	U	K.GMCTSPPLIK.H + Oxidation (M)
171	560.2823	1118.5501	1118.5464	0.0037	0	(55)	0.0024	1	U	K.GMCTSPPLIK.H + Oxidation (M)
172	560.2824	1118.5502	1118.5464	0.0038	0	(40)	0.06	1	U	K.GMCTSPPLIK.H + Oxidation (M)
173	560.2825	1118.5503	1118.5464	0.0039	0	(41)	0.057	1	U	K.GMCTSPPLIK.H + Oxidation (M)
174	560.2829	1118.5512	1118.5464	0.0048	0	64	0.0003	1	U	K.GMCTSPPLIK.H + Oxidation (M)
175	565.7707	1129.5268	1129.5226	0.0042	0	51	0.0031	1	U	R.CNEYYLLR.G
176	565.7707	1129.5269	1129.5226	0.0043	0	(49)	0.0046	1	U	R.CNEYYLLR.G
188	598.8049	1195.5952	1195.5913	0.0039	0	(52)	0.0034	1	U	R.IAQYYYTFK.S
189	598.8056	1195.5966	1195.5913	0.0053	0	(65)	0.00018	1	U	R.IAQYYYTFK.S
190	598.8062	1195.5977	1195.5913	0.0064	0	79	7.8e-006	1	U	R.IAQYYYTFK.S
191	1196.6050	1195.5978	1195.5913	0.0064	0	(18)	8.3	1	U	R.IAQYYYTFK.S
198	610.3313	1218.6481	1218.6431	0.0050	0	(63)	0.00036	1	U	R.CPPPPLPINSK.I
199	610.3314	1218.6483	1218.6431	0.0052	0	(63)	0.00036	1	U	R.CPPPPLPINSK.I
200	610.3316	1218.6486	1218.6431	0.0056	0	64	0.00035	1	U	R.CPPPPLPINSK.I
201	611.8173	1221.6201	1221.6176	0.0025	1	(48)	0.009	1	U	R.GEVKYPLCTR.K
202	611.8179	1221.6212	1221.6176	0.0036	1	(41)	0.048	1	U	R.GEVKYPLCTR.K
203	611.8184	1221.6222	1221.6176	0.0047	1	52	0.0038	1	U	R.GEVKYPLCTR.K
204	617.7956	1233.5767	1233.5740	0.0027	0	(33)	0.19	1	U	K.SFYFPMSIDK.K
205	617.7965	1233.5785	1233.5740	0.0045	0	34	0.15	1	U	K.SFYFPMSIDK.K
208	625.7940	1249.5735	1249.5689	0.0046	0	(27)	0.68	1	U	K.SFYFPMSIDK.K + Oxidation (M)
220	439.5734	1315.6985	1315.6925	0.0060	0	(62)	0.00045	1	U	R.LIENGYFHPVK.Q
221	658.8565	1315.6985	1315.6925	0.0060	0	77	1.4e-005	1	U	R.LIENGYFHPVK.Q
222	439.5736	1315.6988	1315.6925	0.0064	0	(62)	0.00046	1	U	R.LIENGYFHPVK.Q
223	658.8567	1315.6988	1315.6925	0.0064	0	(73)	4e-005	1	U	R.LIENGYFHPVK.Q
224	658.8573	1315.7000	1315.6925	0.0075	0	(72)	5.2e-005	1	U	R.LIENGYFHPVK.Q
225	439.5740	1315.7000	1315.6925	0.0076	0	(49)	0.0091	1	U	R.LIENGYFHPVK.Q
226	661.3424	1320.6703	1320.6674	0.0029	0	64	0.00025	1	U	R.QSTLSYQEPLR.T
227	661.3437	1320.6728	1320.6674	0.0055	0	(63)	0.00035	1	U	R.QSTLSYQEPLR.T
235	673.8072	1345.5999	1345.5972	0.0027	1	(46)	0.0053	1	U	R.CEDGKWTEPPK.C
236	673.8081	1345.6016	1345.5972	0.0044	1	50	0.0022	1	U	R.CEDGKWTEPPK.C
243	681.8442	1361.6739	1361.6689	0.0050	1	36	0.13	1	U	K.SFYFPMSIDKK.L
254	468.8769	1403.6089	1403.6041	0.0049	0	(31)	0.11	1	U	R.CFDHHFLEGSR.E
255	468.8771	1403.6094	1403.6041	0.0054	0	60	0.00012	1	U	R.CFDHHFLEGSR.E
258	708.3741	1414.7336	1414.7279	0.0057	0	(88)	1.1e-006	1	U	K.VLHGDLIDFVCK.Q
259	472.5852	1414.7336	1414.7279	0.0057	0	(51)	0.005	1	U	K.VLHGDLIDFVCK.Q
260	472.5856	1414.7349	1414.7279	0.0070	0	(42)	0.036	1	U	K.VLHGDLIDFVCK.Q
261	708.3747	1414.7349	1414.7279	0.0070	0	99	8.8e-008	1	U	K.VLHGDLIDFVCK.Q
262	711.8676	1421.7206	1421.7150	0.0056	1	62	0.00041	1	U	R.QSTLSYQEPLRT.-
263	711.8683	1421.7220	1421.7150	0.0069	1	(60)	0.00067	1	U	R.QSTLSYQEPLRT.-
292	532.2592	1593.7557	1593.7497	0.0060	1	55	0.0014	1	U	K.IYYNGDKVTYACK.S
293	532.2597	1593.7572	1593.7497	0.0074	1	(36)	0.093	1	U	K.IYYNGDKVTYACK.S
310	838.8633	1675.7120	1675.7446	-0.0326	1	(1)	58	5	U	K.IQENMRYGCASGYK.T

311	838.8643	1675.7141	1675.7446	-0.0306	1	2	54	2	U	K.IQENMRYGCASGYK.T
321	848.9166	1695.8187	1695.8138	0.0049	0	(86)	1e-006	1	U	K.CTKPDLSNGYISDVK.L
322	566.2805	1695.8198	1695.8138	0.0060	0	(66)	0.00011	1	U	K.CTKPDLSNGYISDVK.L
323	848.9172	1695.8198	1695.8138	0.0060	0	(75)	1.2e-005	1	U	K.CTKPDLSNGYISDVK.L
324	566.2806	1695.8201	1695.8138	0.0063	0	(81)	3.2e-006	1	U	K.CTKPDLSNGYISDVK.L
325	848.9173	1695.8201	1695.8138	0.0063	0	98	6.5e-008	1	U	K.CTKPDLSNGYISDVK.L
326	566.2809	1695.8209	1695.8138	0.0072	0	(65)	0.00014	1	U	K.CTKPDLSNGYISDVK.L
334	574.2763	1719.8071	1719.7999	0.0073	0	62	0.00018	1	U	K.SGYLLHGSNEITCNR.G
341	884.9630	1767.9115	1767.9043	0.0072	0	78	8.2e-006	1	U	R.GDTYPAELYITGSILR.M
382	637.6272	1909.8598	1909.8516	0.0082	1	60	0.00017	1	U	K.DKVQYECATGYYTAGGK.K
383	956.9567	1911.8989	1911.8924	0.0065	0	95	9.2e-008	1	U	K.TEEVECLTYGWSLTPK.C
396	1018.4400	2034.8654	2034.8589	0.0065	0	114	2.4e-010	1	U	R.QEEQTTCTTEGWSPEPR.C
397	1018.4414	2034.8682	2034.8589	0.0093	0	(108)	8.5e-010	1	U	R.QEEQTTCTTEGWSPEPR.C
398	681.0058	2039.9956	2039.9874	0.0082	1	60	0.00038	1	U	K.KTEEVECLTYGWSLTPK.C
399	681.0068	2039.9985	2039.9874	0.0112	1	(58)	0.00052	1	U	K.KTEEVECLTYGWSLTPK.C
402	692.3182	2073.9328	2073.9248	0.0080	1	(50)	0.0013	1	U	R.GKWTLPPECVENNENCK.H
403	692.3185	2073.9336	2073.9248	0.0088	1	(51)	0.001	1	U	R.GKWTLPPECVENNENCK.H
404	692.3187	2073.9341	2073.9248	0.0093	1	62	7.8e-005	1	U	R.GKWTLPPECVENNENCK.H
406	695.0050	2081.9932	2081.9840	0.0092	0	(70)	2.8e-005	1	U	K.VACEEPPFIENGAANLHSK.I
407	695.0056	2081.9949	2081.9840	0.0109	0	73	1.6e-005	1	U	K.VACEEPPFIENGAANLHSK.I
408	695.0059	2081.9957	2081.9840	0.0117	0	(66)	7.5e-005	1	U	K.VACEEPPFIENGAANLHSK.I
418	713.3482	2137.0227	2137.0150	0.0077	2	51	0.0023	1	U	K.VKDKVQYECATGYYTAGGK.K
419	713.3489	2137.0249	2137.0150	0.0099	2	(40)	0.036	1	U	K.VKDKVQYECATGYYTAGGK.K
431	1139.0669	2276.1193	2276.1107	0.0087	0	(28)	0.61	1	U	K.QGYDLSPLTPLSELSVQCNR.G
432	1139.0684	2276.1223	2276.1107	0.0116	0	88	5.2e-007	1	U	K.QGYDLSPLTPLSELSVQCNR.G
433	759.7147	2276.1223	2276.1107	0.0116	0	(29)	0.46	1	U	K.QGYDLSPLTPLSELSVQCNR.G
509	899.7385	2696.1936	2696.1807	0.0129	1	(67)	1.4e-005	1	U	K.TTGGKDEEVVQCLSDGWSSQPTCR.K
510	899.7391	2696.1956	2696.1807	0.0149	1	105	2.1e-009	1	U	K.TTGGKDEEVVQCLSDGWSSQPTCR.K
511	899.7393	2696.1961	2696.1807	0.0154	1	(99)	7.8e-009	1	U	K.TTGGKDEEVVQCLSDGWSSQPTCR.K
512	899.7396	2696.1970	2696.1807	0.0164	1	(87)	1.5e-007	1	U	K.TTGGKDEEVVQCLSDGWSSQPTCR.K
527	926.4617	2776.3632	2776.3490	0.0142	0	87	5.5e-007	1	U	K.HPPVVMNGAVADGILASYATGSSVEYR.C + Oxidation (M)
528	926.4618	2776.3635	2776.3490	0.0145	0	(35)	0.084	1	U	K.HPPVVMNGAVADGILASYATGSSVEYR.C + Oxidation (M)

3. F13A_RAT **Mass:** 83120 **Score:** 789 **Matches:** 30(21) **Sequences:** 9(6) **emPAI:** 0.44
Coagulation factor XIII A chain OS=Rattus norvegicus GN=F13a1 PE=2 SV=3
Check to include this hit in error tolerant search or archive report

Query	Observed	Mr(expt)	Mr(calc)	Delta	Miss	Score	Expect	Rank	Unique	Peptide
119	418.2390	834.4634	834.4599	0.0034	0	(11)	63	3		R.VEYVIGR.Y
120	418.2390	834.4635	834.4599	0.0036	0	32	0.56	1		R.VEYVIGR.Y
131	438.7282	875.4419	875.4535	-0.0116	0	(35)	0.3	1		R.VGSAMVNAK.D
132	438.7342	875.4539	875.4535	0.0004	0	(36)	0.24	1		R.VGSAMVNAK.D
133	438.7346	875.4546	875.4535	0.0011	0	(54)	0.004	1		R.VGSAMVNAK.D
134	438.7347	875.4549	875.4535	0.0015	0	60	0.001	1		R.VGSAMVNAK.D
135	438.7348	875.4550	875.4535	0.0016	0	(56)	0.0023	1		R.VGSAMVNAK.D
213	640.8524	1279.6903	1279.6846	0.0058	0	(78)	9.2e-006	1		R.LALETALMYGAK.K
215	648.8493	1295.6840	1295.6795	0.0045	0	101	4e-008	1		R.LALETALMYGAK.K + Oxidation (M)
216	648.8498	1295.6851	1295.6795	0.0056	0	(96)	1.3e-007	1		R.LALETALMYGAK.K + Oxidation (M)
217	648.8502	1295.6859	1295.6795	0.0064	0	(77)	1.1e-005	1		R.LALETALMYGAK.K + Oxidation (M)
245	683.8809	1365.7472	1365.7405	0.0067	1	(8)	67	1		K.DLFRVEYVIGR.Y
246	683.8814	1365.7482	1365.7405	0.0078	1	29	0.57	1		K.DLFRVEYVIGR.Y
294	532.2811	1593.8216	1593.8151	0.0065	1	29	0.66	1		R.VEYVIGRYPQENK.G
295	532.2815	1593.8226	1593.8151	0.0075	1	(25)	1.6	1		R.VEYVIGRYPQENK.G
313	844.4696	1686.9247	1686.9192	0.0055	0	(33)	0.21	3	U	K.GTYIPVPVVTELQSGK.W
314	844.4700	1686.9255	1686.9192	0.0062	0	(40)	0.042	3	U	K.GTYIPVPVVTELQSGK.W
315	844.4708	1686.9270	1686.9192	0.0077	0	46	0.011	3	U	K.GTYIPVPVVTELQSGK.W
316	564.6187	1690.8343	1690.8275	0.0068	0	(66)	0.00012	1		K.DGTHVVENVDATHIGK.L
317	564.6188	1690.8346	1690.8275	0.0071	0	95	1.8e-007	1		K.DGTHVVENVDATHIGK.L
318	564.6188	1690.8347	1690.8275	0.0072	0	(70)	5.3e-005	1		K.DGTHVVENVDATHIGK.L
319	564.6196	1690.8369	1690.8275	0.0094	0	(53)	0.0026	1		K.DGTHVVENVDATHIGK.L
363	607.3167	1818.9282	1818.9224	0.0058	1	(69)	7.6e-005	1		K.KDGTHVVENVDATHIGK.L
364	607.3170	1818.9293	1818.9224	0.0068	1	(64)	0.00022	1		K.KDGTHVVENVDATHIGK.L
365	607.3177	1818.9314	1818.9224	0.0089	1	87	1e-006	1		K.KDGTHVVENVDATHIGK.L
455	805.0649	2412.1730	2412.1631	0.0099	1	(53)	0.0016	1		K.FQEGQEEERLALETALMYGAK.K
456	805.0663	2412.1772	2412.1631	0.0141	1	79	3.9e-006	1		K.FQEGQEEERLALETALMYGAK.K
458	810.3970	2428.1691	2428.1580	0.0111	1	(54)	0.001	1		K.FQEGQEEERLALETALMYGAK.K + Oxidation (M)
459	810.3978	2428.1715	2428.1580	0.0136	1	(37)	0.055	1		K.FQEGQEEERLALETALMYGAK.K + Oxidation (M)
460	810.3984	2428.1732	2428.1580	0.0152	1	(48)	0.0041	1		K.FQEGQEEERLALETALMYGAK.K + Oxidation (M)

4. TRYP_PIG **Mass:** 25078 **Score:** 377 **Matches:** 13(10) **Sequences:** 5(4) **emPAI:** 1.21
Trypsin OS=Sus scrofa PE=1 SV=1
Check to include this hit in error tolerant search or archive report

Query	Observed	Mr(expt)	Mr(calc)	Delta	Miss	Score	Expect	Rank	Unique	Peptide
121	421.7596	841.5047	841.5022	0.0025	0	54	0.0027	1	U	R.VATVSLPR.S
122	421.7600	841.5054	841.5022	0.0032	0	(54)	0.0029	1	U	R.VATVSLPR.S
155	523.2863	1044.5580	1044.5564	0.0016	0	(49)	0.012	1	U	K.LSSPATLNSR.V
156	523.2868	1044.5591	1044.5564	0.0028	0	(41)	0.083	1	U	K.LSSPATLNSR.V
157	523.2869	1044.5592	1044.5564	0.0028	0	56	0.0025	1	U	K.LSSPATLNSR.V
426	737.7100	2210.1081	2210.0967	0.0114	0	(76)	9.5e-006	1	U	R.LGEHNIDVLEGNEQFINAAK.I
427	737.7101	2210.1084	2210.0967	0.0117	0	108	6.6e-009	1	U	R.LGEHNIDVLEGNEQFINAAK.I
428	737.7106	2210.1101	2210.0967	0.0134	0	(91)	3.3e-007	1	U	R.LGEHNIDVLEGNEQFINAAK.I
434	761.7352	2282.1838	2282.1729	0.0109	0	(58)	0.0006	1	U	K.IITHPNFNGNTLDNDIMLIK.L
436	761.7359	2282.1860	2282.1729	0.0131	0	60	0.00035	1	U	K.IITHPNFNGNTLDNDIMLIK.L
437	767.0674	2298.1804	2298.1678	0.0126	0	(31)	0.3	1	U	K.IITHPNFNGNTLDNDIMLIK.L + Oxidation (M)
438	767.0684	2298.1834	2298.1678	0.0156	0	(45)	0.012	1	U	K.IITHPNFNGNTLDNDIMLIK.L + Oxidation (M)
544	828.1912	3308.7359	3308.7187	0.0172	1	35	0.054	1	U	K.IITHPNFNGNTLDNDIMLIKLSSPATLNSR.V

5. K2C1_HUMAN **Mass:** 66170 **Score:** 150 **Matches:** 4(4) **Sequences:** 3(3) **emPAI:** 0.17
Keratin, type II cytoskeletal 1 OS=Homo sapiens GN=KRT1 PE=1 SV=6
Check to include this hit in error tolerant search or archive report

Query	Observed	Mr(expt)	Mr(calc)	Delta	Miss	Score	Expect	Rank	Unique	Peptide
149	487.2714	972.5282	972.5240	0.0042	0	43	0.047	1		K.IEISELNR.V
218	651.8635	1301.7124	1301.7078	0.0046	0	(64)	0.00028	1	U	R.SLDLDSIIAEVK.A
219	651.8639	1301.7133	1301.7078	0.0055	0	64	0.00025	1	U	R.SLDLDSIIAEVK.A
330	858.9317	1715.8489	1715.8438	0.0051	0	84	2.2e-006	1	U	K.QISNLQQSISDAEQR.G

Proteins matching the same set of peptides:
K2C1_PANTR **Mass:** 65621 **Score:** 150 **Matches:** 4(4) **Sequences:** 3(3)

Keratin, type II cytoskeletal 1 OS=Pan troglodytes GN=KRT1 PE=2 SV=1

---

6. F13A_BOVIN **Mass:** 22844 **Score:** 147 **Matches:** 4(4) **Sequences:** 2(2) **emPAI:** 0.34
Coagulation factor XIII A chain (Fragment) OS=Bos taurus GN=F13A1 PE=1 SV=2
Check to include this hit in error tolerant search or archive report

Query	Observed	Mr(expt)	Mr(calc)	Delta	Miss	Score	Expect	Rank	Unique	Peptide
287	785.9112	1569.8079	1569.8014	0.0065	0	64	0.0002	1	U	R.MYVAVWTPYGVIR.T + Oxidation (M)
313	844.4696	1686.9247	1686.9192	0.0055	0	(55)	0.0016	1	U	K.GTYIPVPLVSELQSGK.W
314	844.4700	1686.9255	1686.9192	0.0062	0	(67)	0.0001	1	U	K.GTYIPVPLVSELQSGK.W
315	844.4708	1686.9270	1686.9192	0.0077	0	70	4.7e-005	1	U	K.GTYIPVPLVSELQSGK.W

---

7. K1C9_HUMAN **Mass:** 62255 **Score:** 51 **Matches:** 1(1) **Sequences:** 1(1) **emPAI:** 0.06
Keratin, type I cytoskeletal 9 OS=Homo sapiens GN=KRT9 PE=1 SV=3
Check to include this hit in error tolerant search or archive report

Query	Observed	Mr(expt)	Mr(calc)	Delta	Miss	Score	Expect	Rank	Unique	Peptide
180	579.3004	1156.5862	1156.5836	0.0025	0	51	0.005	1	U	R.QGVDADINGLR.Q

---

8. K22E_HUMAN **Mass:** 65678 **Score:** 40 **Matches:** 2(1) **Sequences:** 2(1) **emPAI:** 0.11
Keratin, type II cytoskeletal 2 epidermal OS=Homo sapiens GN=KRT2 PE=1 SV=2
Check to include this hit in error tolerant search or archive report

Query	Observed	Mr(expt)	Mr(calc)	Delta	Miss	Score	Expect	Rank	Unique	Peptide
149	487.2714	972.5282	972.5240	0.0042	0	43	0.047	1		K.IEISELNR.V
233	665.3706	1328.7267	1328.7187	0.0079	0	27	1.4	1	U	R.NLDLDSIIAEVK.A

---

9. ISPH_SYNS3 **Mass:** 44896 **Score:** 18 **Matches:** 2(0) **Sequences:** 1(0) **emPAI:** 0.08
4-hydroxy-3-methylbut-2-enyl diphosphate reductase OS=Synechococcus sp. (strain CC9311) GN=ispH PE=3 SV=1
Check to include this hit in error tolerant search or archive report

Query	Observed	Mr(expt)	Mr(calc)	Delta	Miss	Score	Expect	Rank	Unique	Peptide
166	559.2928	1116.5710	1116.5928	-0.0218	0	18	13	1	U	K.QSFTSIIHGK.V
167	559.2940	1116.5734	1116.5928	-0.0193	0	(10)	76	1	U	K.QSFTSIIHGK.V

Peptide matches not assigned to protein hits: (no details means no match)

Query	Observed	Mr(expt)	Mr(calc)	Delta	Miss	Score	Expect	Rank	Unique	Peptide
345	887.9568	1773.8990	1773.8567	0.0423	0	26	1.4	1		LNDGASSVILMSESHAK + Oxidation (M)
344	887.9567	1773.8988	1773.8857	0.0131	2	25	1.9	1		GRVEELGEEIGRESSK
119	418.2390	834.4634	834.4236	0.0398	0	20	7.9	1		VEFDLGR
253	463.2020	1386.5841	1386.5730	0.0111	0	14	3.7	1		NPFVMMMDSGAR + 2 Oxidation (M)
123	846.4444	845.4371	845.4495	-0.0124	0	14	50	1		DTVVEVGK
114	418.1656	834.3166	834.3654	-0.0488	0	13	3.8	1		VEGSCQR
234	669.3631	1336.7116	1336.6696	0.0419	1	13	33	1		FAKNLMEEIDK
113	835.3239	834.3166	834.3654	-0.0488	0	12	4.4	1		VEGSCQR
250	690.3762	1378.7378	1378.7707	-0.0329	1	12	35	1		GYSSLKLLEEIK
339	883.8993	1765.7841	1765.8226	-0.0385	1	12	10	1		KMEQESIDQLCELK + Oxidation (M)
370	608.6234	1822.8484	1822.8922	-0.0437	1	11	22	1		SEDNHRNQLENIAVGK
117	835.4705	834.4632	834.4599	0.0033	0	10	86	1		LAAYELR
193	601.8193	1201.6240	1201.6277	-0.0038	0	10	78	1		VNKPMLGHYK + Oxidation (M)
116	835.4701	834.4629	834.4599	0.0029	0	10	88	1		LAAYELR
30	550.2998	549.2925	549.2659	0.0266	0	9	1.1e+002	1		GGNFR
31	550.3003	549.2931	549.2659	0.0271	0	9	1.1e+002	1		GGNFR
29	550.2997	549.2924	549.2659	0.0265	0	9	1.1e+002	1		GGNFR
192	601.8178	1201.6211	1201.6277	-0.0066	0	9	94	1		VNKPMLGHYK + Oxidation (M)
140	463.1873	924.3600	924.3937	-0.0337	0	9	7.9	1		GFGSEEGSR
312	840.4033	1678.7921	1678.8276	-0.0355	0	9	44	1		SGDLDPAIPFFIMEK
145	469.7178	937.4210	937.4658	-0.0448	1	9	47	1		REGIGYPF
566	689.8336	4132.9580	4132.9340	0.0240	2	8	13	1		AYGARIETMHGLSGLGDLTMCCSTPQSRNFSFGVALGR + Oxidation (M)
300	551.9653	1652.8740	1652.8886	-0.0146	2	8	79	1		TDPSYRKIALSFQK
150	1018.4418	1017.4345	1017.4338	0.0007	0	7	30	1		CWSPTDPR
304	552.5937	1654.7592	1654.7872	-0.0281	0	6	57	1		CIGDPEADVENPVLK
251	690.8777	1379.7409	1379.7449	-0.0040	1	6	1.4e+002	1		TKTPDNFFGIIK
124	846.4449	845.4376	845.4680	-0.0304	1	6	2.9e+002	1		MIPTKEK
477	861.0526	2580.1359	2580.1519	-0.0160	2	6	17	1		TNPMDKTIVFCNDIDHADRMR + 2 Oxidation (M)
414	705.0087	2112.0044	2112.0521	-0.0477	2	5	84	1		LGDVMKESVQAAESYVKSR + Oxidation (M)
362	910.3775	1818.7404	1818.7885	-0.0482	1	5	7.9	1		YMKCGIGVCGSCNLGK + Oxidation (M)
115	835.4700	834.4627	834.4599	0.0028	0	5	2.7e+002	1		QVYLANK
257	703.8546	1405.6947	1405.6837	0.0110	1	5	1.9e+002	1		YRETLLDEPDR
565	579.2955	4048.0176	4048.0211	-0.0036	1	5	45	1		LEEFQLLRGESFACHSLVSAASVSGSGTAEKPSLLQDK
338	883.8992	1765.7838	1765.8226	-0.0388	1	4	55	1		KMEQESIDQLCELK + Oxidation (M)
302	552.5928	1654.7567	1654.7699	-0.0133	1	4	93	1		RTNTEPHDSQATWI
247	456.2765	1365.8075	1365.7980	0.0096	1	4	79	1		APALTASLPRLEK
542	808.6618	3230.6181	3230.6016	0.0165	2	4	92	1		ALEGDKEAQEQILKLMDTVDEYIPTPER
386	648.6822	1943.0249	1942.9934	0.0315	1	4	2e+002	1		ELAEMPPPQSRLLQYR + Oxidation (M)
59	588.3596	587.3523	587.3101	0.0422	0	4	1e+003	1		APICK
384	957.4584	1912.9021	1912.9386	-0.0365	1	3	1.3e+002	1		NCLVEYTTSERLIGMK
410	699.6766	2096.0080	2096.0399	-0.0319	2	3	1.4e+002	1		GRSSKSPAHWNQVVSEAEK
596	846.4437	5072.6182	5072.5787	0.0396	2	3	21	1		AQKSQVQHMVQQILKLPAAPQADAADALGVALCHFHTYQSLIAMGGK + 2 Oxidation (M)
550	1139.5649	3415.6729	3415.6460	0.0269	1	3	84	1		SYQVPFADFTLTSTLYDIDVTGYETATKNR
557	625.8102	3748.8175	3748.7997	0.0178	2	3	72	1		YAVPTVWYTCYGVRRYGFHGISHAYVAEEAAR
211	628.7771	1255.5396	1255.5325	0.0070	0	3	75	1		LYCGGPMNNSK + Oxidation (M)
248	686.3290	1370.6434	1370.6904	-0.0470	0	3	1.6e+002	1		VASIMYLYPGNK + Oxidation (M)
249	686.8343	1371.6541	1371.6969	-0.0428	0	3	2e+002	1		MALPGPAVFGPGSR + Oxidation (M)
76	622.3676	621.3603	621.3156	0.0447	0	3	4.4e+002	1		GIMSSK
584	1166.0786	4660.2855	4660.3047	-0.0192	2	3	51	1		TAKKPLTVAGYDMDEDVKYAGPSGHIFDGLNFYILTDSSAPIK + Oxidation (M)
496	880.1218	2637.3435	2637.2938	0.0497	0	2	1.8e+002	1		IAIWGWSYGGYVTSMVLGSGSGVFK + Oxidation (M)
401	1025.4502	2048.8859	2048.9334	-0.0475	1	2	46	1		VGEMEAPNRSTQFNSGGPR + Oxidation (M)
336	580.9540	1739.8403	1739.8625	-0.0222	1	2	2.5e+002	1		SGATGNFTQTRTLAGMK
493	1308.6720	2615.3295	2615.3642	-0.0347	2	2	2.1e+002	1		AVTFLLSQGVMFSNEGRGYVLRR + Oxidation (M)
443	777.3527	2329.0362	2329.0467	-0.0106	0	2	67	1		GTTAVAECMEPTIHAALNGHSY
499	664.5655	2654.2327	2654.2801	-0.0473	1	2	1.1e+002	1		LGEALACRFMITISCCKPLENR + Oxidation (M)
567	692.3577	4148.1025	4148.0625	0.0400	2	2	83	1		AEKSHFLMPGFTHLQYAQPTRLSHHLLAYHDMLSR + Oxidation (M)
501	664.5662	2654.2356	2654.2801	-0.0445	1	1	1.2e+002	1		LGEALACRFMITISCCKPLENR + Oxidation (M)
578	640.8437	4478.8550	4478.9019	-0.0469	1	1	1.8	1		QNTAEFICNALKNVFLYCYTCAHQYDDPLEMMMGCR + Oxidation (M)
411	699.6774	2096.0105	2096.0031	0.0074	1	1	2.3e+002	1		AVVMDSQYLIEPCGSGKSR
99	718.4975	717.4903	717.4497	0.0406	2	1	64	1		SSIRKK
601	911.9228	5465.4933	5465.5328	-0.0395	1	1	16	1		VLFSVANMMHPGTLEHGDPAEFSTKGISFFMTLPCYGEAEQNFNLMLR + 2 Oxidation (M)
278	503.8967	1508.6681	1508.7041	-0.0360	0	1	1.5e+002	1		CQNYLAESKPSGR
491	1308.6720	2615.3294	2615.3517	-0.0222	0	1	2.6e+002	1		LGLDAVDGVLMDLGVSSFQLDNPLK
194	602.2662	1202.5178	1202.5602	-0.0423	0	1	1.2e+002	1		LDLDCPDTVR
405	693.6782	2078.0127	2078.0240	-0.0113	1	1	3.2e+002	1		KTTTVSGTSQTPTSAPSGSQR
413	705.0051	2111.9935	2112.0381	-0.0447	2	0	2.4e+002	1		NVAAPKDNEESQAAPRMLR + Oxidation (M)
502	664.5663	2654.2361	2654.2801	-0.0439	1	0	1.5e+002	1		LGEALACRFMITISCCKPLENR + Oxidation (M)
582	572.7749	4574.1412	4574.1226	0.0186	1	0	52	1		GGTGAIVEYFGSGVESLSCTGMATICNMGAEIGATTSLFPFNKR + Oxidation (M)

299	827.4443	1652.8739	1652.8352	0.0388	2	0	4.6e+002	1	MVHGRMVHGRSVSGK + Oxidation (M)
516	904.4122	2710.2148	2710.2479	-0.0331	2	0	75	1	RYINDLDEMQIDDREISMPWR + Oxidation (M)
449	1185.5327	2369.0507	2369.0747	-0.0239	1	0	92	1	SGFKSTHEFVTCFGDSIDAHK
448	1185.5316	2369.0487	2369.0747	-0.0260	1	0	87	1	SGFKSTHEFVTCFGDSIDAHK
303	552.5936	1654.7589	1654.7872	-0.0284	1	0	2.3e+002	1	GITDDMKSFQSAIDK
1	402.3583	401.3510							
2	413.2507	412.2434							
3	415.2121	414.2048							
4	415.2126	414.2053							
5	415.2127	414.2054							
6	415.3426	414.3353							
7	425.3091	424.3019							
8	432.2391	431.2318							
9	432.2392	431.2319							
10	432.2393	431.2320							
11	432.2819	431.2746							
12	433.3534	432.3461							
13	443.2693	442.2620							
14	444.1173	443.1100							
15	444.1206	443.1133							
16	444.3516	443.3443							
17	446.2614	445.2541							
18	447.2924	446.2852							
19	456.2815	455.2742							
20	500.3068	499.2995							
21	500.3074	499.3001							
22	507.9564	506.9491							
23	520.3339	519.3266							
24	528.3597	527.3525							
25	531.2753	530.2681							
26	539.3347	538.3274							
27	547.3350	546.3277							
28	548.9835	547.9762							
32	551.5020	550.4948							
33	551.5045	550.4972							
34	552.3064	551.2991							
35	564.3606	563.3533							
36	564.3616	563.3543							
37	566.9574	565.9502							
38	579.5336	578.5263							
39	579.5337	578.5265							
40	579.5340	578.5267							
41	579.5341	578.5269							
42	579.5345	578.5273							
43	579.5348	578.5275							
44	579.5352	578.5279							
45	579.5354	578.5281							
46	579.5359	578.5286							
47	579.5360	578.5287							
48	579.5360	578.5287							
49	579.5361	578.5288							
50	579.5362	578.5289							
51	579.5362	578.5289							
52	579.5362	578.5289							
53	579.5363	578.5290							
54	579.5363	578.5290							
55	579.5365	578.5292							
56	579.5365	578.5292							
57	579.5372	578.5299							
58	586.4172	585.4099							
60	597.5438	596.5365							
61	597.5444	596.5372							
62	597.5451	596.5379							
63	614.5705	613.5632							
64	614.5705	613.5632							
65	614.5708	613.5635							
66	614.5711	613.5638							
67	614.5711	613.5638							
68	614.5712	613.5639							
69	615.4045	614.3972							
70	615.4051	614.3979							
71	615.4058	614.3985							
72	617.4077	616.4004							
73	619.5271	618.5199							
74	619.5297	618.5224							
75	622.3659	621.3587							
77	630.4437	629.4365							
78	630.4445	629.4372							
79	650.2052	649.1979							
80	650.2061	649.1988							
81	652.4138	651.4065							
82	661.5631	660.5559							
83	661.5633	660.5561							
84	661.5663	660.5590							
85	661.5681	660.5608							
86	662.2433	661.2360							
87	674.4704	673.4631							
88	674.4710	673.4637							
89	675.6770	674.6697							
90	675.6774	674.6701							
91	675.6795	674.6722							
92	677.6837	676.6764							

93	693.5644	692.5572
95	717.6259	716.6186
96	717.6259	716.6187
97	717.6264	716.6191
98	718.4973	717.4900
100	734.6498	733.6425
101	739.6073	738.6000
102	739.6074	738.6001
103	739.6076	738.6003
104	752.5467	751.5394
105	752.5477	751.5404
106	786.4310	785.4237
107	786.4314	785.4242
108	786.4325	785.4252
109	788.1409	787.1336
110	788.1410	787.1338
112	412.1703	822.3260
118	835.4707	834.4634
130	433.3528	864.6911
139	901.8107	900.8035
141	934.8787	933.8715
142	934.8794	933.8721
143	934.8820	933.8747
144	468.1520	934.2895
146	481.1877	960.3607
147	481.1879	960.3612
148	481.1879	960.3613
	544.1362	1086.2579
	544.1366	1086.2587
	579.2432	1156.4719
	583.2926	1164.5707
195	1216.0683	1215.0610
	610.2518	1218.4889
	610.2519	1218.4893
	625.5586	1249.1026
	625.5590	1249.1035
	628.2837	1254.5528
	628.7195	1255.4245
	640.7225	1279.4304
	641.7692	1281.5238
	661.7541	1321.4936
	674.7664	1347.5182
	674.7674	1347.5202
	680.7450	1359.4755
	680.7455	1359.4765
	680.7456	1359.4766
	680.7458	1359.4770
	682.8925	1363.7705
	694.2993	1386.5841
	703.8006	1405.5866
	721.8052	1441.5959
	721.8055	1441.5964
	722.2978	1442.5810
	481.8676	1442.5810
	492.5528	1474.6365
	743.6925	1485.3705
	743.6930	1485.3715
	751.6921	1501.3696
	752.7912	1503.5679
	502.1966	1503.5679
	506.3144	1515.9214
	771.3220	1540.6294
	519.5425	1555.6056
	523.5045	1567.4916
	523.5051	1567.4934
	784.7540	1567.4934
	526.9046	1577.6920
	528.2241	1581.6504
	791.8328	1581.6511
	528.2243	1581.6512
	551.8536	1652.5389
	827.2767	1652.5389
	552.9147	1655.7222
	552.9152	1655.7237
	846.8630	1691.7115
	566.5533	1696.6381
	566.5536	1696.6391
	854.7821	1707.5496
	574.2257	1719.6552
	860.8351	1719.6557
	574.2258	1719.6557
	870.9274	1739.8403
	580.9544	1739.8414
	885.8165	1769.6184
	885.8177	1769.6209
	595.2096	1782.6070
	893.8149	1785.6153
	893.8150	1785.6154
	897.3235	1792.6324
	602.2670	1803.7793
358	907.4184	1812.8222

	907.4187	1812.8229
	907.4195	1812.8244
	908.4716	1814.9286
	607.9462	1820.8167
	608.2456	1821.7150
	609.9455	1826.8147
	609.9461	1826.8164
	612.3023	1833.8850
	612.5859	1834.7360
	616.1911	1845.5513
	923.8595	1845.7043
	616.2421	1845.7044
	616.9471	1847.8196
	622.9193	1865.7361
	622.9199	1865.7379
	947.9113	1893.8081
	970.3668	1938.7191
	649.9159	1946.7258
	652.4089	1954.2049
	655.9458	1964.8156
	658.2084	1971.6033
	503.3033	2009.1842
400	1025.4478	2048.8810
	696.9354	2087.7843
	703.7982	2108.3729
	711.8679	2132.5819
	712.6430	2134.9071
	712.6436	2134.9089
	722.9732	2165.8976
	544.3340	2173.3068
	551.5031	2201.9833
	442.3561	2206.7441
	745.6699	2233.9880
	755.3494	2263.0263
435	1142.0992	2282.1838
	579.5345	2314.1090
	774.8155	2321.4247
	777.3534	2329.0383
	779.3739	2335.0998
	779.6804	2336.0195
	784.3649	2350.0727
	791.8326	2372.4760
	794.6998	2381.0777
	1202.0150	2402.0154
	1202.0156	2402.0166
	1202.0161	2402.0177
	808.3286	2421.9639
461	1228.0356	2454.0567
462	1228.0370	2454.0595
463	1228.0378	2454.0611
	614.5724	2454.2604
	819.3181	2454.9325
	819.3184	2454.9333
	822.8688	2465.5847
	415.3442	2486.0214
	838.8643	2513.5711
	840.4013	2518.1821
	1264.9622	2527.9099
	1264.9635	2527.9124
	1264.9639	2527.9133
	854.9077	2561.7012
	861.0530	2580.1371
	520.3316	2596.6218
	868.7829	2603.3269
	653.4145	2609.6291
	872.7838	2615.3295
	872.7838	2615.3295
	1308.9309	2615.8473
	884.9614	2651.8623
	885.4171	2653.2294
	885.7522	2654.2349
	890.4221	2668.2445
	890.4225	2668.2456
	890.4227	2668.2463
	891.9125	2672.7156
	897.3161	2688.9264
	539.3331	2691.6291
	676.4114	2701.6165
	904.4112	2710.2118
	904.4119	2710.2140
	911.4208	2731.2405
	688.0353	2748.1119
	917.3773	2749.1101
	688.2848	2749.1101
	704.0925	2812.3409
	947.9096	2840.7069
	573.0495	2860.2110
534	1531.1799	3060.3453
	438.7434	3064.1526
	769.4054	3073.5925
	619.5267	3092.5969

	539	1038.7623	3113.2650
☑	540	1038.7624	3113.2654
☑	541	539.3281	3229.9248
☑	543	651.8634	3254.2809
☑	553	1180.4951	3538.4635
☑	554	1185.8253	3554.4540
☑	555	1185.8271	3554.4594
☑	556	1185.8276	3554.4611
☑	558	628.7207	3766.2807
☑	559	549.1589	3837.0613
☑	560	551.4937	3853.4053
☑	561	551.4940	3853.4073
☑	562	551.5044	3853.4798
☑	563	483.9246	3863.3388
☑	564	560.2766	3914.8851
☑	568	594.3709	4153.5452
☑	569	699.9894	4193.8926
☑	570	534.3145	4266.4576
☑	571	4293.8938	4292.8865
☑	572	549.1580	4385.2056
☑	573	550.3423	4394.6804
☑	574	551.5051	4403.9823
☑	575	743.6948	4456.1249
☑	576	558.8067	4462.3951
☑	577	558.8103	4462.4243
☑	579	1506.6366	4516.8880
☑	580	1506.6372	4516.8898
☑	581	1143.4589	4569.8066
☑	583	774.8170	4642.8586
☑	585	4687.0795	4686.0722
☑	586	1172.7421	4686.9394
☑	587	1172.7436	4686.9454
☑	588	789.8534	4733.0767
☑	589	4755.8017	4754.7944
☑	590	686.8333	4800.7818
☑	591	4902.8537	4901.8464
☑	592	4954.4508	4953.4435
☑	593	4954.4719	4953.4646
☑	594	4971.9984	4970.9911
☑	595	711.7974	4975.5307
☑	597	5159.1706	5158.1633
☑	598	654.3894	5227.0567
☑	599	901.8093	5404.8120
☑	600	910.8922	5459.3098
☑	602	5479.4971	5478.4898
☑	603	5522.1449	5521.1376
☑	604	5536.5956	5535.5883
☑	605	5614.4470	5613.4397
☑	606	947.9137	5681.4383
☑	607	720.4283	5755.3680
☑	608	5914.8628	5913.8555
☑	609	6135.7525	6134.7452
☑	610	1038.0081	6222.0052
☑	611	785.9100	6279.2219
☑	612	791.8334	6326.6087
☑	613	6397.9366	6396.9293
☑	614	6561.9263	6560.9190
☑	615	827.4450	6611.5020
☑	616	6764.8602	6763.8529
☑	617	6871.1341	6870.1268
☑	618	868.6326	6941.0028
☑	619	7212.7363	7211.7290
☑	620	7273.6011	7272.5938
☑	621	7347.7019	7346.6946
☑	622	7394.8418	7393.8345
☑	623	925.3625	7394.8418
☑	624	948.7866	7582.2344
☑	625	1290.9813	7739.8439
☑	626	7790.4618	7789.4545
☑	627	7819.3139	7818.3066
☑	628	8376.6776	8375.6703
☑	629	8391.5646	8390.5573
☑	630	8706.3541	8705.3468
☑	631	9119.6564	9118.6491
☑	632	9592.5715	9591.5642

Search Parameters

Type of search : **MS/MS Ion Search**
Enzyme : **Trypsin**
Fixed modifications : **Carbamidomethyl (C)**
Variable modifications : **Oxidation (M)**
Mass values : **Monoisotopic**
Protein Mass : **Unrestricted**
Peptide Mass Tolerance : **± 0.05 Da**
Fragment Mass Tolerance: **± 0.05 Da**
Max Missed Cleavages : **2**
Instrument type : **Default**
Number of queries : **632**

Mascot: http://www.matrixscience.com/

Mascot Search Results

User	: Julian
Email	: julian.langer@mpibp-frankfurt.mpg.de
Search title	: Submitted from 140318 842 ChKrettler 1-10 swissprot full T 0.05Da by Mascot Daemon on CCSW010
MS data file	: D:\Data\1403\140318\140314 842 ChReinhard 5_Tray01-D1_01_17686.d\140314 842 ChReinhard 5_Tray01-D1_01_17686.mgf
Database	: SwissProt 57.15 (515203 sequences; 181334896 residues)
Timestamp	: 19 Mar 2014 at 09:50:30 GMT
Protein hits	: ALBU_HUMAN Serum albumin OS=Homo sapiens GN=ALB PE=1 SV=2
	K2C1_HUMAN Keratin, type II cytoskeletal 1 OS=Homo sapiens GN=KRT1 PE=1 SV=6
	TRYP_PIG Trypsin OS=Sus scrofa PE=1 SV=1
	K1C9_HUMAN Keratin, type I cytoskeletal 9 OS=Homo sapiens GN=KRT9 PE=1 SV=3
	K1C10_CANFA Keratin, type I cytoskeletal 10 OS=Canis familiaris GN=KRT10 PE=2 SV=1
	F13A_HUMAN Coagulation factor XIII A chain OS=Homo sapiens GN=F13A1 PE=1 SV=4
	F13B_HUMAN Coagulation factor XIII B chain OS=Homo sapiens GN=F13B PE=1 SV=3
	A1BG_HUMAN Alpha-1B-glycoprotein OS=Homo sapiens GN=A1BG PE=1 SV=3

Mascot Score Histogram

Ions score is -10*Log(P), where P is the probability that the observed match is a random event.
Individual ions scores > 39 indicate identity or extensive homology (p<0.05).
Protein scores are derived from ions scores as a non-probabilistic basis for ranking protein hits.

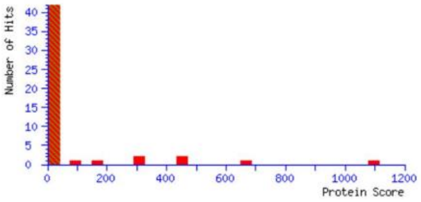

Peptide Summary Report

Format As: Peptide Summary | Help

Significance threshold p< 0.05 | Max. number of hits AUTO

Standard scoring | MudPIT scoring | Ions score or expect cut-off 0 | Show sub-sets 0

Show pop-ups | Suppress pop-ups | Sort unassigned Decreasing Score | Require bold red

Select All | Select None | Search Selected | Error tolerant | Archive Report

1. ALBU_HUMAN **Mass:** 71317 **Score:** 1094 **Matches:** 49(25) **Sequences:** 25(13) **emPAI:** 1.34
Serum albumin OS=Homo sapiens GN=ALB PE=1 SV=2
Check to include this hit in error tolerant search or archive report

Query	Observed	Mr(expt)	Mr(calc)	Delta	Miss	Score	Expect	Rank	Unique	Peptide
73	673.3795	672.3722	672.3707	0.0015	0	33	0.79	1		K.AWAVAR.L
86	772.4411	771.4338	771.4313	0.0025	0	18	20	1	U	K.AACLLPK.L
87	789.4739	788.4667	788.4644	0.0023	0	(14)	35	8		K.LVTDLTK.V
88	789.4741	788.4668	788.4644	0.0025	0	33	0.49	1		K.LVTDLTK.V
105	438.2597	874.5048	874.5025	0.0023	1	23	5.4	1		R.LSQRFPK.A
106	440.7258	879.4370	879.4338	0.0033	0	33	0.35	1	U	K.AEFAEVSK.L
112	927.4963	926.4890	926.4861	0.0029	0	(20)	5.3	1		K.YLYEIAR.R
113	464.2518	926.4890	926.4861	0.0029	0	35	0.15	1		K.YLYEIAR.R
115	464.2521	926.4897	926.4861	0.0035	0	(35)	0.15	1		K.YLYEIAR.R
119	480.7858	959.5570	959.5552	0.0017	0	43	0.044	1	U	K.FQNALLVR.Y
122	492.7505	983.4865	983.4811	0.0054	0	25	1.4	1	U	K.TYETTLEK.C
124	500.8071	999.5997	999.5964	0.0033	0	(43)	0.038	1		K.QTALVELVK.H
125	500.8071	999.5997	999.5964	0.0033	0	53	0.0036	1		K.QTALVELVK.H
126	500.8072	999.5998	999.5964	0.0034	0	(51)	0.0054	1		K.QTALVELVK.H
127	507.3040	1012.5934	1012.5917	0.0017	0	(14)	22	1	U	K.LVAASQAALGL.-
128	1013.6017	1012.5945	1012.5917	0.0028	0	49	0.0068	1	U	K.LVAASQAALGL.-
129	1013.6021	1012.5948	1012.5917	0.0031	0	(29)	0.71	1	U	K.LVAASQAALGL.-
139	528.2997	1054.5848	1054.5811	0.0037	1	18	7.1	1	U	K.KYLYEIAR.R
154	564.8546	1127.6947	1127.6914	0.0033	1	(58)	0.00064	1		K.KQTALVELVK.H
155	564.8553	1127.6961	1127.6914	0.0047	1	72	2.2e-005	1		K.KQTALVELVK.H
157	569.7540	1137.4935	1137.4907	0.0028	0	(49)	0.0026	1		K.CCTESLVNR.R
158	569.7547	1137.4949	1137.4907	0.0042	0	53	0.0011	1		K.CCTESLVNR.R
160	575.3122	1148.6098	1148.6077	0.0021	0	48	0.013	1		K.LVNEVTEFAK.T
173	613.8087	1225.6028	1225.5979	0.0050	1	63	0.00025	1	U	R.FKDLGEENFK.A
174	409.5416	1225.6029	1225.5979	0.0050	1	(28)	0.8	1	U	R.FKDLGEENFK.A
175	613.8090	1225.6035	1225.5979	0.0056	1	(39)	0.064	1	U	R.FKDLGEENFK.A
191	679.8210	1357.6273	1357.6224	0.0049	0	99	3.7e-008	1	U	K.AVMDDFAAFVEK.C + Oxidation (M)
197	722.3270	1442.6395	1442.6347	0.0047	0	(67)	3.5e-005	1	U	K.YICENQDSISSK.L
198	722.3273	1442.6400	1442.6347	0.0053	0	78	2.6e-006	1	U	K.YICENQDSISSK.L
199	722.3284	1442.6423	1442.6347	0.0076	0	(56)	0.0004	1	U	K.YICENQDSISSK.L
202	516.2719	1545.7940	1545.7894	0.0045	1	(48)	0.011	1	U	K.LKECCEKPLLEK.S
203	516.2724	1545.7954	1545.7894	0.0060	1	(55)	0.0022	1	U	K.LKECCEKPLLEK.S
204	516.2725	1545.7956	1545.7894	0.0061	1	62	0.00041	1	U	K.LKECCEKPLLEK.S
205	516.2726	1545.7960	1545.7894	0.0066	1	(56)	0.0018	1	U	K.LKECCEKPLLEK.S
209	547.3191	1638.9355	1638.9305	0.0051	1	(65)	9.2e-005	1		K.KVPQVSTPTLVEVSR.N
210	547.3196	1638.9370	1638.9305	0.0065	1	68	4.1e-005	1		K.KVPQVSTPTLVEVSR.N
225	637.6510	1909.9311	1909.9244	0.0067	0	(46)	0.0084	1	U	R.RPCFSALEVDETYVPK.E
227	637.6513	1909.9321	1909.9244	0.0077	0	54	0.0014	1	U	R.RPCFSALEVDETYVPK.E
233	682.3719	2044.0940	2044.0881	0.0059	0	(44)	0.016	1	U	K.VFDEFKPLVEEPQNLIK.Q
235	682.3723	2044.0951	2044.0881	0.0070	0	52	0.0026	1	U	K.VFDEFKPLVEEPQNLIK.Q
266	647.0378	2584.1221	2584.1105	0.0116	1	(4)	20	1	U	K.VHTECCHGDLLECADDRADLAK.Y
267	647.0379	2584.1225	2584.1105	0.0120	1	10	5.6	1	U	K.VHTECCHGDLLECADDRADLAK.Y
270	659.8151	2635.2315	2635.2197	0.0117	2	(21)	1.3	1	U	K.QEPERNECFLQHKDDNPNLPR.L
271	659.8154	2635.2323	2635.2197	0.0126	2	(16)	4.6	1	U	K.QEPERNECFLQHKDDNPNLPR.L
272	659.8156	2635.2333	2635.2197	0.0135	2	29	0.2	1	U	K.QEPERNECFLQHKDDNPNLPR.L
273	659.8157	2635.2335	2635.2197	0.0138	2	(20)	1.6	1	U	K.QEPERNECFLQHKDDNPNLPR.L
280	699.3466	2793.3572	2793.3466	0.0106	1	(22)	1.6	1	U	R.LVRPEVDVMCTAFHDNEETFLKK.Y + Oxidation (M)
282	699.3466	2793.3573	2793.3466	0.0107	1	27	0.6	1	U	R.LVRPEVDVMCTAFHDNEETFLKK.Y + Oxidation (M)
286	997.4552	2989.3436	2989.3321	0.0115	0	20	0.75	1	U	K.SHCIAEVENDEMPADLPSLAADFVESK.D + Oxidation (M)

Proteins matching the same set of peptides:
ALBU_PONAB **Mass:** 71317 **Score:** 1094 **Matches:** 49(25) **Sequences:** 25(13)
Serum albumin OS=Pongo abelii GN=ALB PE=2 SV=1

---

2. K2C1_HUMAN **Mass:** 66170 **Score:** 679 **Matches:** 22(16) **Sequences:** 14(11) **emPAI:** 0.94
Keratin, type II cytoskeletal 1 OS=Homo sapiens GN=KRT1 PE=1 SV=6
Check to include this hit in error tolerant search or archive report

Query	Observed	Mr(expt)	Mr(calc)	Delta	Miss	Score	Expect	Rank	Unique	Peptide
93	832.4852	831.4779	831.4814	-0.0035	0	8	1.6e+002	5	U	K.SISISVAR.G
103	437.7539	873.4933	873.4920	0.0014	0	(23)	6.1	1	U	R.SLVNLGGSK.S

104	437.7549	873.4952	873.4920	0.0033	0	51	0.0082	1	U	R.SLVNLGGSK.S
120	487.2702	972.5259	972.5240	0.0019	0	43	0.052	1	U	K.IEISELNR.V
121	487.2704	972.5263	972.5240	0.0023	0	(34)	0.44	1	U	K.IEISELNR.V
123	500.2275	998.4405	998.4379	0.0026	0	19	2.8	1	U	K.DVDGAYMTK.V
132	517.2620	1032.5094	1032.5087	0.0006	0	(41)	0.053	1	U	R.TLLEGEESR.M
133	517.2631	1032.5116	1032.5087	0.0029	0	42	0.049	1	U	R.TLLEGEESR.M
134	517.2639	1032.5132	1032.5087	0.0045	0	(42)	0.052	1	U	R.TLLEGEESR.M
146	533.2636	1064.5126	1064.5138	-0.0012	0	41	0.042	1	U	K.AQYEDIAQK.S
162	590.3015	1178.5884	1178.5931	-0.0047	0	53	0.0034	1	U	K.YEELQITAGR.H
163	590.3057	1178.5968	1178.5931	0.0037	0	(39)	0.093	1	U	K.YEELQITAGR.H
181	633.3243	1264.6341	1264.6299	0.0042	0	50	0.0062	1	U	R.TNAENEFVTIK.K
182	639.3588	1276.7031	1276.7027	0.0004	0	60	0.00057	1	U	K.LALDLEIATYR.T
183	651.8570	1301.6994	1301.6939	0.0055	1	48	0.012	1	U	R.NSKIEISELNR.V
184	651.8634	1301.7122	1301.7078	0.0044	0	48	0.011	1		R.SLDLDSIIAEVK.A
185	651.8634	1301.7123	1301.7078	0.0044	0	(47)	0.013	1		R.SLDLDSIIAEVK.A
193	692.3513	1382.6880	1382.6830	0.0050	0	52	0.0034	1	U	K.SLNNQFASFIDK.V
194	697.3719	1392.7292	1392.7249	0.0043	1	(81)	4.8e-006	1	U	R.TNAENEFVTIKK.D
195	465.2504	1392.7295	1392.7249	0.0046	1	(61)	0.00041	1	U	R.TNAENEFVTIKK.D
196	697.3720	1392.7295	1392.7249	0.0046	1	89	7.9e-007	1	U	R.TNAENEFVTIKK.D
200	738.3980	1474.7814	1474.7780	0.0035	0	78	1e-005	1		R.FLEQQNQVLQTK.W

Proteins matching the same set of peptides:
K2C1_PANTR **Mass:** 65621 **Score:** 679 **Matches:** 22(16) **Sequences:** 14(11)
Keratin, type II cytoskeletal 1 OS=Pan troglodytes GN=KRT1 PE=2 SV=1

---

3. TRYP_PIG **Mass:** 25078 **Score:** 465 **Matches:** 24(21) **Sequences:** 7(6) **emPAI:** 1.88
Trypsin OS=Sus scrofa PE=1 SV=1
Check to include this hit in error tolerant search or archive report

Query	Observed	Mr(expt)	Mr(calc)	Delta	Miss	Score	Expect	Rank	Unique	Peptide
94	421.7587	841.5028	841.5022	0.0007	0	(53)	0.0033	1	U	R.VATVSLPR.S
95	421.7598	841.5051	841.5022	0.0030	0	(54)	0.0029	1	U	R.VATVSLPR.S
96	421.7600	841.5054	841.5022	0.0032	0	54	0.0026	1	U	R.VATVSLPR.S
135	523.2868	1044.5590	1044.5564	0.0026	0	(49)	0.011	1	U	K.LSSPATLNSR.V
136	523.2869	1044.5593	1044.5564	0.0029	0	52	0.0056	1	U	K.LSSPATLNSR.V
137	523.2876	1044.5606	1044.5564	0.0043	0	(46)	0.024	1	U	K.LSSPATLNSR.V
140	532.2595	1062.5044	1062.5016	0.0029	0	(94)	2.3e-007	1	U	K.APVLSDSSCK.S
141	532.2597	1062.5049	1062.5016	0.0033	0	(94)	2.2e-007	1	U	K.APVLSDSSCK.S
142	532.2600	1062.5054	1062.5016	0.0039	0	94	2.2e-007	1	U	K.APVLSDSSCK.S
244	737.7078	2210.1015	2210.0967	0.0048	0	(77)	8.2e-006	1	U	R.LGEHNIDVLEGNEQFINAAK.I
245	1106.0601	2210.1057	2210.0967	0.0090	0	(13)	18	1	U	R.LGEHNIDVLEGNEQFINAAK.I
246	737.7092	2210.1057	2210.0967	0.0090	0	103	1.8e-008	1	U	R.LGEHNIDVLEGNEQFINAAK.I
247	737.7092	2210.1059	2210.0967	0.0091	0	(90)	3.5e-007	1	U	R.LGEHNIDVLEGNEQFINAAK.I
251	761.7336	2282.1789	2282.1729	0.0061	0	(69)	4.9e-005	1	U	K.IITHPNFNGNTLDNDIMLIK.L
253	761.7338	2282.1796	2282.1729	0.0067	0	(61)	0.00028	1	U	K.IITHPNFNGNTLDNDIMLIK.L
254	761.7338	2282.1796	2282.1729	0.0067	0	(42)	0.024	1	U	K.IITHPNFNGNTLDNDIMLIK.L
257	767.0661	2298.1764	2298.1678	0.0086	0	(43)	0.018	1	U	K.IITHPNFNGNTLDNDIMLIK.L + Oxidation (M)
258	767.0663	2298.1770	2298.1678	0.0092	0	69	4.3e-005	1	U	K.IITHPNFNGNTLDNDIMLIK.L + Oxidation (M)
259	767.0665	2298.1775	2298.1678	0.0097	0	(68)	5.9e-005	1	U	K.IITHPNFNGNTLDNDIMLIK.L + Oxidation (M)
292	828.1871	3308.7195	3308.7187	0.0008	1	77	3.4e-006	1	U	K.IITHPNFNGNTLDNDIMLIKLSSPATLNSR.V
293	828.1910	3308.7347	3308.7187	0.0160	1	(51)	0.0014	1	U	K.IITHPNFNGNTLDNDIMLIKLSSPATLNSR.V
294	832.1891	3324.7273	3324.7136	0.0137	1	(37)	0.032	1	U	K.IITHPNFNGNTLDNDIMLIKLSSPATLNSR.V + Oxidation (M)
295	832.1906	3324.7335	3324.7136	0.0199	1	(14)	7.6	1	U	K.IITHPNFNGNTLDNDIMLIKLSSPATLNSR.V + Oxidation (M)
367	917.8127	5500.8325	5500.8048	0.0276	2	14	1.6	1	U	R.LGEHNIDVLEGNEQFINAAKIITHPNFNGNTLDNDIMLIKLSSPATLNSR.V

---

4. K1C9_HUMAN **Mass:** 62255 **Score:** 435 **Matches:** 14(9) **Sequences:** 10(6) **emPAI:** 0.54
Keratin, type I cytoskeletal 9 OS=Homo sapiens GN=KRT9 PE=1 SV=3
Check to include this hit in error tolerant search or archive report

Query	Observed	Mr(expt)	Mr(calc)	Delta	Miss	Score	Expect	Rank	Unique	Peptide
108	449.2116	896.4087	896.4062	0.0025	0	45	0.01	1	U	R.MTLDDFR.I
109	449.2117	896.4089	896.4062	0.0027	0	(42)	0.022	1	U	R.MTLDDFR.I
145	533.2543	1064.4940	1064.4920	0.0020	0	37	0.086	1	U	K.STMQELNSR.L
161	579.2998	1156.5851	1156.5836	0.0014	0	63	0.00026	1	U	R.QGVDADINGLR.Q
176	616.8040	1231.5935	1231.5906	0.0029	0	(77)	1.1e-005	1	U	R.SGGGGGGGLGSGGSIR.S
177	616.8042	1231.5938	1231.5906	0.0032	0	(79)	6.5e-006	1	U	R.SGGGGGGGLGSGGSIR.S
178	616.8043	1231.5941	1231.5906	0.0035	0	90	5.8e-007	1	U	R.SGGGGGGGLGSGGSIR.S
180	618.2698	1234.5250	1234.5215	0.0036	0	51	0.00093	1	U	R.FSSSSGYGGGSSR.V
186	654.3447	1306.6749	1306.6703	0.0046	1	45	0.02	1	U	R.IKFEMEQNLR.Q
223	613.3284	1836.9635	1836.9581	0.0054	0	15	14	1	U	R.HGVQELEIELQSQLSK.K
224	617.9831	1850.9274	1850.9196	0.0078	1	19	5.2	1	U	K.TLNDMRQEYEQLIAK.N
288	1075.1069	3222.2989	3222.2744	0.0245	0	19	0.066	1	U	R.GGSGGSHGGGSGFGGESGGSYGGGEEASGSGGGYGGGSGK.S
289	1088.8471	3263.5196	3263.5066	0.0130	0	(13)	6.6	1	U	K.DIENQYETQITQIEHEVSSSGQEVQSSAK.E
290	1088.8483	3263.5231	3263.5066	0.0165	0	49	0.0014	1	U	K.DIENQYETQITQIEHEVSSSGQEVQSSAK.E

---

5. K1C10_CANFA **Mass:** 57847 **Score:** 314 **Matches:** 10(4) **Sequences:** 7(4) **emPAI:** 0.34
Keratin, type I cytoskeletal 10 OS=Canis familiaris GN=KRT10 PE=2 SV=1
Check to include this hit in error tolerant search or archive report

Query	Observed	Mr(expt)	Mr(calc)	Delta	Miss	Score	Expect	Rank	Unique	Peptide
89	404.2046	806.3946	806.3923	0.0024	0	53	0.0046	1		R.LAADDFR.L
131	516.3044	1030.5943	1030.5910	0.0033	0	11	57	2	U	R.VLDELTLTK.A
143	532.8101	1063.6056	1063.6026	0.0031	1	58	0.00073	1		R.LASYLDKVR.A
144	532.8103	1063.6061	1063.6026	0.0035	1	(36)	0.11	1		R.LASYLDKVR.A
148	545.7709	1089.5272	1089.5237	0.0035	0	17	12	1		K.VTMQNLNDR.L
179	617.8457	1233.6768	1233.6717	0.0051	1	(35)	0.22	1	U	R.LKYENEVALR.Q
61	617.8463	1233.6780	1233.6717	0.0063	1	56	0.0014	1	U	R.LKYENEVALR.Q
189	679.3654	1356.7163	1356.7110	0.0053	1	36	0.17	1	U	R.QSVEADINGLRR.V
190	679.3657	1356.7169	1356.7110	0.0060	1	(12)	42	1	U	R.QSVEADINGLRR.V
192	691.3306	1380.6467	1380.6408	0.0058	0	82	2.1e-006	1	U	R.ALEESNYELEGK.I

Proteins matching the same set of peptides:
K1C10_HUMAN **Mass:** 59020 **Score:** 314 **Matches:** 10(4) **Sequences:** 7(4)
Keratin, type I cytoskeletal 10 OS=Homo sapiens GN=KRT10 PE=1 SV=6

---

6. F13A_HUMAN **Mass:** 83728 **Score:** 308 **Matches:** 11(7) **Sequences:** 6(5) **emPAI:** 0.27
Coagulation factor XIII A chain OS=Homo sapiens GN=F13A1 PE=1 SV=4
Check to include this hit in error tolerant search or archive report

Query	Observed	Mr(expt)	Mr(calc)	Delta	Miss	Score	Expect	Rank	Unique	Peptide
100	430.2543	858.4940	858.4811	0.0130	0	46	0.033	1	U	R.LSIQSSPK.C
130	515.7720	1029.5294	1029.5277	0.0017	0	74	2.9e-005	1	U	R.CGPASVQAIK.H
152	558.8084	1115.6022	1115.6009	0.0014	0	(24)	3.3	1	U	K.KPLNTEGVMK.S
153	558.8093	1115.6040	1115.6009	0.0032	0	(24)	3.2	1	U	K.KPLNTEGVMK.S
156	566.8071	1131.5997	1131.5958	0.0039	0	43	0.038	1	U	K.KPLNTEGVMK.S + Oxidation (M)
213	564.6185	1690.8337	1690.8275	0.0062	0	61	0.00043	1	U	K.DGTHVVENVDATHIGK.L
214	564.6191	1690.8354	1690.8275	0.0080	0	(48)	0.0087	1	U	K.DGTHVVENVDATHIGK.L
222	607.3180	1818.9321	1818.9224	0.0097	1	24	2.2	1	U	K.KDGTHVVENVDATHIGK.L
297	1138.5238	3412.5496	3412.5517	-0.0022	0	(32)	0.04	1	U	R.IVTNYFSAHDNDANLQMDIFLEEDGNVNSK.L
298	1138.5248	3412.5524	3412.5517	0.0007	0	60	6.7e-005	1	U	R.IVTNYFSAHDNDANLQMDIFLEEDGNVNSK.L
301	1143.8564	3428.5474	3428.5467	0.0008	0	(11)	4.9	1	U	R.IVTNYFSAHDNDANLQMDIFLEEDGNVNSK.L + Oxidation (M)

---

7. F13B_HUMAN **Mass:** 77742 **Score:** 170 **Matches:** 4(3) **Sequences:** 3(3) **emPAI:** 0.14

Coagulation factor XIII B chain OS=Homo sapiens GN=F13B PE=1 SV=3

Check to include this hit in error tolerant search or archive report

Query	Observed	Mr(expt)	Mr(calc)	Delta	Miss	Score	Expect	Rank	Unique	Peptide
169	610.3305	1218.6463	1218.6431	0.0033	0	46	0.02	1	U	R.CPPPPLPINSK.I
187	439.5731	1315.6976	1315.6925	0.0051	0	62	0.00051	1	U	R.LIENGYFHPVK.Q
239	695.0038	2081.9896	2081.9840	0.0056	0	(32)	0.19	1	U	K.VACEEPPFIENGAANLHSK.I
240	695.0039	2081.9899	2081.9840	0.0059	0	62	0.00018	1	U	K.VACEEPPFIENGAANLHSK.I

8. A1BG_HUMAN **Mass:** 54809 **Score:** 59 **Matches:** 2(2) **Sequences:** 1(1) **emPAI:** 0.06

Alpha-1B-glycoprotein OS=Homo sapiens GN=A1BG PE=1 SV=3

Check to include this hit in error tolerant search or archive report

Query	Observed	Mr(expt)	Mr(calc)	Delta	Miss	Score	Expect	Rank	Unique	Peptide
101	435.7699	869.5253	869.5222	0.0031	0	(49)	0.0065	1	U	K.LLELTGPK.S
102	435.7701	869.5257	869.5222	0.0035	0	59	0.00068	1	U	K.LLELTGPK.S

Peptide matches not assigned to protein hits: (no details means no match)

Query	Observed	Mr(expt)	Mr(calc)	Delta	Miss	Score	Expect	Rank	Unique	Peptide
170	610.8089	1219.6033	1219.5986	0.0048	0	16	18	1		NFPSPVDAAFR
87	789.4739	788.4667	788.4644	0.0023	0	15	28	1		VLTEVTK
218	887.9553	1773.8961	1773.8567	0.0394	0	15	18	1		LNDGASSVILMSESHAK + Oxidation (M)
231	404.2020	2015.9739	2015.9623	0.0116	1	12	20	1		ALDEITVDGFVYRTDMR + Oxidation (M)
215	575.3061	1722.8964	1722.9012	-0.0048	2	12	32	1		RVHELSEDEAAAIRK
131	516.3044	1030.5943	1030.5519	0.0424	1	12	48	1		VIDGSRASAR
219	887.9560	1773.8975	1773.8567	0.0408	0	12	37	1		AEAVALEACPSSNVQTK
138	526.2781	1050.5416	1050.5345	0.0071	1	11	59	1		LEEYVDKR
93	832.4852	831.4779	831.4450	0.0329	0	10	94	1		SLSQAQAK
97	846.4346	845.4274	845.4495	-0.0221	0	10	91	1		DTVVEVGK
374	844.4540	5904.1268	5904.0951	0.0317	0	10	1.4	1		ASVIVPNDPDLIDILAMNGVDISVSEGESSGNDLFTVIGNLIFPLLAFGGLFLLFR
375	844.4561	5904.1419	5904.0951	0.0468	0	10	1.4	1		ASVIVPNDPDLIDILAMNGVDISVSEGESSGNDLFTVIGNLIFPLLAFGGLFLLFR
165	605.8029	1209.5913	1209.5924	-0.0011	0	9	69	1		MITASSPHNPR
211	561.2935	1680.8588	1680.8361	0.0227	1	8	71	1		MSLCQGSAMQKLIAK + Oxidation (M)
164	605.8028	1209.5911	1209.6176	-0.0265	0	8	88	1		LYGAVVQAMSR + Oxidation (M)
216	575.3136	1722.9190	1722.9093	0.0097	1	8	79	1		SFYQPVNTVLNKWK
249	755.3466	2263.0181	2263.0244	-0.0064	0	7	23	1		EEMAMMGFNEMLTLTLLNK + 3 Oxidation (M)
356	5276.6273	5275.6200	5275.6555	-0.0355	1	7	13	1		SIAAFAFPLFGQDMYDTLGYGWGNSLLAFIVLGSSIITCSLLWFGGKR + Oxidation (M)
278	464.2498	2779.4550	2779.4845	-0.0295	1	6	65	1		EKFMYLILASLLLYMGFVAAFAPR + Oxidation (M)
315	651.8613	3905.1238	3905.0873	0.0365	2	6	12	1		VYIPPVCALLGDPTVNVREAAIQTLVEIYKHVGDR
380	6105.2029	6104.1956	6104.1835	0.0121	1	5	4.8	1		VLPALANMNLSARLGVGAAHLLMALIEPQQLLAVGFGETTMCALQHLSGFISSQQIR + 3 Oxidation (M)
314	651.8589	3905.1096	3905.0758	0.0337	2	5	18	1		APVIISHGKSNSKAIQNAIFQAINAASSNLDSIIEQR
287	533.2547	3193.4844	3193.4972	-0.0128	2	5	44	1		AFNIDEIEVKEMEPNENITSEKLEDNK + Oxidation (M)
159	571.8218	1141.6290	1141.6244	0.0046	0	4	2.5e+002	1		QGHNSVFLIK
265	430.2457	2575.4305	2575.4044	0.0262	2	4	41	1		GDVSSQFLTALLMTLPLVKAKDGR + Oxidation (M)
243	540.2887	2157.1255	2157.1476	-0.0221	2	4	1.6e+002	1		GAPAFARLEEAILNRVMQR + Oxidation (M)
275	532.2595	2656.2609	2656.2989	-0.0380	2	4	82	1		MIDAAKKAMHGYISDVYITVDQR + 2 Oxidation (M)
285	579.3008	2891.4677	2891.4988	-0.0311	2	4	1.2e+002	1		RAAEAGVTGILAADLPPEEASDALRAAEK
98	846.4441	845.4369	845.4283	0.0086	0	4	5.1e+002	1		EEAIAWK
362	5404.6831	5403.6758	5403.6869	-0.0111	2	3	24	1		SIPGFANLDLNDQVTLLKYGVYEAIFAMLSSVMNKDGMLVAYGNGFITR + 3 Oxidation (M)
255	762.0613	2283.1621	2283.1569	0.0052	2	3	1.8e+002	1		VQTIEEVMTYIDKWTKQR + Oxidation (M)
91	828.4412	2482.3019	2482.2704	0.0315	1	3	7.3e+002	1		IAPTLDDEDVHTFIERLLTER
237	691.3271	2070.9595	2070.9429	0.0166	1	2	1.2e+002	1		VQDMNYHVEEKDHLGNK + Oxidation (M)
302	579.2994	3469.7526	3469.7478	0.0048	1	2	1.1e+002	1		QTIGTADSVGRYIGGYVAYDNGPLSASLGLAQQK
71	666.3927	665.3854	665.3860	-0.0006	0	2	4.1e+002	1		HAVLIN
283	403.5950	2818.1141	2818.1347	-0.0206	0	2	1.9	1		EQNSPSCEGQQYQQGEYMNSPWR + Oxidation (M)
242	719.6886	2156.0441	2156.0763	-0.0322	2	1	2.4e+002	1		IFENSKPEESSFRARGFR
350	639.3462	5106.7116	5106.6635	0.0481	1	1	18	1		TMATIDRPAIAALWPTLRGESVVLDVGATIGADAHQLIDFAILGTGMAR + 2 Oxidation (M)
348	613.8084	4902.4090	4902.4172	-0.0082	2	1	50	1		LIQNTNSDAELNAFGEVLHRAVNVDTSNLGRFNSSGEPLISFDNK
337	738.3912	4424.3037	4424.2979	0.0058	2	1	61	1		LMQKMTIMAFIGNFAESIQMLTPQLHAIIAASVSIKSSQK + 3 Oxidation (M)
383	6357.2827	6356.2754	6356.2397	0.0357	1	1	17	1		GVTMNEPFFQGHFPAQPVMPGVLILEALAQAAALLATLSMKPEEVNDKITYLMGIDAAR + 2 Oxidation (M)
206	528.3533	1582.0380	1582.0004	0.0377	2	1	2.7	1		LRVINIMKLLLEK
99	846.4443	845.4370	845.4395	-0.0025	0	1	9.7e+002	1		AVNFNGPK
372	5758.6168	5757.6095	5757.6321	-0.0225	1	1	9	1		FSMLNDLVSGNISQSSNTSSNNSTGANTSTNTTNGLGSNSTNYSYINSTHLNKNK
264	500.8238	2499.0828	2499.1199	-0.0371	1	1	41	1		LVAEGKAYYCFCSQADLDAYR
220	594.3526	1780.0359	1780.0206	0.0153	1	1	1.3e+002	1		IAAIRGTTVALPLEAER
347	4767.9803	4766.9730	4766.9402	0.0328	2	0	2	1		DDADYEYSGSEDDEPSPNNRGPSMGIRDDSESSSMIPMDNTLR + Oxidation (M)
57	615.4043	614.3971	614.4115	-0.0145	1	0	1.2e+003	1		GKIIGK
1	*415.2118*	*414.2046*								
2	*415.2122*	*414.2049*								
3	*415.2124*	*414.2051*								
4	*415.2551*	*414.2478*								
5	*415.3422*	*414.3350*								
6	*415.3433*	*414.3360*								
7	*416.2622*	*415.2550*								
8	*418.3894*	*417.3821*								
9	*428.3702*	*427.3629*								
10	*430.2447*	*429.2374*								
11	*430.2447*	*429.2374*								
12	*430.2450*	*429.2377*								
13	*432.2377*	*431.2304*								
14	*432.2389*	*431.2316*								
15	*432.2812*	*431.2739*								
16	*432.2817*	*431.2744*								
17	*444.1170*	*443.1097*								
18	*444.1177*	*443.1105*								
19	*444.1181*	*443.1108*								
20	*446.2602*	*445.2529*								
21	*447.1359*	*446.1287*								
22	*456.2804*	*455.2731*								
23	*456.2812*	*455.2740*								
24	*459.2812*	*458.2739*								
25	*476.3074*	*475.3001*								
26	*476.3078*	*475.3005*								
27	*476.3079*	*475.3006*								
28	*500.3074*	*499.3002*								
29	*500.3076*	*499.3003*								
30	*506.3177*	*505.3104*								
31	*520.3336*	*519.3263*								
32	*520.3342*	*519.3269*								
33	*520.3342*	*519.3269*								
34	*525.3215*	*524.3143*								
35	*534.3135*	*533.3063*								
36	*534.9815*	*533.9742*								
37	*548.3296*	*547.3223*								
38	*550.3431*	*549.3358*								
39	*564.3600*	*563.3527*								
40	*564.3606*	*563.3534*								
41	*564.3607*	*563.3535*								

42	578.3392	577.3320
43	579.5353	578.5281
44	579.5361	578.5288
45	579.5362	578.5290
46	579.5364	578.5291
47	579.5371	578.5298
48	586.4171	585.4099
49	594.3709	593.3636
50	595.3703	594.3630
51	597.5448	596.5375
52	608.3862	607.3789
53	608.3864	607.3791
54	608.3864	607.3791
55	608.3871	607.3798
56	614.5713	613.5641
58	615.4045	614.3972
59	615.4048	614.3976
60	617.4081	616.4008
62	619.5272	618.5199
63	619.5275	618.5202
64	619.5275	618.5202
65	630.4399	629.4327
66	630.4446	629.4373
67	640.5862	639.5789
68	650.2048	649.1976
69	652.4126	651.4053
70	652.4138	651.4065
72	668.6227	667.6154
74	674.4697	673.4624
75	675.6768	674.6695
76	675.6780	674.6707
77	675.6787	674.6714
78	675.6794	674.6721
79	696.4390	695.4317
80	696.4390	695.4317
81	718.4947	717.4874
82	752.5469	751.5396
83	752.5478	751.5405
84	762.5228	761.5155
85	765.4677	764.4605
90	412.7547	823.4948
92	415.2535	828.4925
107	444.3481	886.6817
110	456.2779	910.5412
111	457.2620	912.5094
114	927.4969	926.4897
116	934.8788	933.8715
117	474.8311	947.6476
118	476.3093	950.6041
	534.2642	1066.5139
	549.7446	1097.4746
	1104.2522	1103.2449
	1111.2840	1110.2767
	608.2578	1214.5011
	608.2578	1214.5011
	608.2581	1214.5016
	613.3599	1224.7052
	613.3612	1224.7079
	444.1164	1329.3273
	739.2770	1476.5394
	540.3380	1617.9921
	545.7281	1634.1626
	564.3524	1690.0353
	591.3599	1771.0578
	607.2404	1818.6992
	955.9733	1909.9321
	651.1993	1950.5760
	666.3927	1996.1563
	402.3567	2006.7473
	679.8196	2036.4369
234	1023.0548	2044.0951
	686.3129	2055.9168
	520.3305	2077.2930
	528.9691	2111.8475
248	1106.0602	2210.1059
	755.3479	2263.0219
252	1142.0971	2282.1796
	762.0616	2283.1629
	779.3719	2335.0938
	779.3723	2335.0952
	614.5705	2454.2529
	415.2487	2485.4485
	432.2587	2587.5084
	432.2832	2587.6553
	883.8915	2648.6527
	459.2864	2749.6747
	695.8552	2779.3916
	466.2883	2791.6862
	559.6787	2793.3573
	412.1669	2878.1172
	550.3366	3295.9759
	671.8101	3354.0141
	570.7426	3418.4121
	490.2867	3424.9563
	579.5246	3471.1041
	613.3506	3674.0601
	921.1313	3680.4960
	3700.5419	3699.5346

307	533.7458	3729.1697						
308	934.8803	3735.4922						
309	476.2974	3802.3212						
310	544.8278	3806.7439						
311	3863.1885	3862.1812						
312	3881.0809	3880.0736						
313	650.2066	3895.1959						
316	561.3165	3922.1648						
317	564.8592	3946.9636						
318	666.6047	3993.5847						
319	500.3184	3994.4894						
320	4007.2522	4006.2449						
321	671.8196	4024.8742	4024.9206	-0.0465	2	---	1	EKFPDQWLAYNLSPSFNWPKAMSVDEQHTFIQR + Oxidation (M)
322	579.5374	4049.7108						
323	4117.4703	4116.4630						
324	4143.1475	4142.1402						
325	594.3717	4153.5512						
326	696.4206	4172.4801						
327	525.2883	4194.2482						
328	527.1981	4209.5266						
329	608.3702	4251.5405						
330	534.9907	4271.8673						
331	614.5584	4294.8580						
332	539.3160	4306.4700						
333	619.5404	4329.7317						
334	622.3605	4349.4722						
335	4385.5684	4384.5611						
336	632.4063	4419.7928						
338	555.2476	4433.9224						
339	640.8509	4478.9053						
340	4520.6378	4519.6305						
341	566.7990	4526.3340						
342	569.7537	4549.9712						
343	651.8542	4555.9287						
344	763.9217	4577.4868						
345	4751.3792	4750.3719						
346	679.7935	4751.5033						
349	4964.4531	4963.4458						
351	5193.2507	5192.2434						
352	650.2043	5193.5758						
353	5196.0230	5195.0157						
354	5240.5009	5239.4936						
355	5246.9749	5245.9676						
357	5280.1156	5279.1083						
358	883.8831	5297.2547						
359	5313.2883	5312.2810						
360	763.8495	5339.8952						
361	5343.0446	5342.0373						
363	5428.0298	5427.0225						
364	5487.3537	5486.3464						
365	1101.1728	5500.8276						
366	1101.1738	5500.8325						
368	696.4286	5563.3705						
369	5592.2301	5591.2228						
370	802.4295	5609.9558						
371	5758.4364	5757.4291						
373	5827.5714	5826.5641						
376	741.4618	5923.6364						
377	6038.4185	6037.4112						
378	6061.1975	6060.1902						
379	763.8837	6103.0116						
381	6202.3284	6201.3211						
382	6328.3006	6327.2933						
384	6399.7439	6398.7366						
385	6533.2925	6532.2852						
386	6650.8648	6649.8575						
387	6655.1091	6654.1018						
388	6749.2971	6748.2898						
389	6840.4088	6839.4015						
390	7002.9920	7001.9847						
391	7816.7668	7815.7595						
392	9045.7312	9044.7239						

Search Parameters

Type of search	: MS/MS Ion Search
Enzyme	: Trypsin
Fixed modifications	: Carbamidomethyl (C)
Variable modifications	: Oxidation (M)
Mass values	: Monoisotopic
Protein Mass	: Unrestricted
Peptide Mass Tolerance	: ± 0.05 Da
Fragment Mass Tolerance	: ± 0.05 Da
Max Missed Cleavages	: 2
Instrument type	: Default
Number of queries	: 392

Mascot: http://www.matrixscience.com/

Mascot Search Results

User : Julian
Email : julian.langer@mpibp-frankfurt.mpg.de
Search title : Submitted from 140318 842 ChKrettler 1-10 swissprot full T 0.05Da by Mascot Daemon on CCSW010
MS data file : D:\Data\1403\140318\140314 842 ChReinhard 6_Tray01-D2_01_17687.d\140314 842 ChReinhard 6_Tray01-D2_01_17687.mgf
Database : SwissProt 57.15 (515203 sequences; 181334896 residues)
Timestamp : 19 Mar 2014 at 09:51:01 GMT
Protein hits :

A2MG_HUMAN	Alpha-2-macroglobulin OS=Homo sapiens GN=A2M PE=1 SV=2
TRYP_PIG	Trypsin OS=Sus scrofa PE=1 SV=1
K2C1_HUMAN	Keratin, type II cytoskeletal 1 OS=Homo sapiens GN=KRT1 PE=1 SV=6
CFAH_HUMAN	Complement factor H OS=Homo sapiens GN=CFH PE=1 SV=4
K1C10_HUMAN	Keratin, type I cytoskeletal 10 OS=Homo sapiens GN=KRT10 PE=1 SV=6
K1C14_HUMAN	Keratin, type I cytoskeletal 14 OS=Homo sapiens GN=KRT14 PE=1 SV=4
K22E_HUMAN	Keratin, type II cytoskeletal 2 epidermal OS=Homo sapiens GN=KRT2 PE=1 SV=2
F13B_HUMAN	Coagulation factor XIII B chain OS=Homo sapiens GN=F13B PE=1 SV=3
K1C9_HUMAN	Keratin, type I cytoskeletal 9 OS=Homo sapiens GN=KRT9 PE=1 SV=3
ALBU_FELCA	Serum albumin OS=Felis catus GN=ALB PE=1 SV=1
F13A_HUMAN	Coagulation factor XIII A chain OS=Homo sapiens GN=F13A1 PE=1 SV=4

Mascot Score Histogram

Ions score is -10*Log(P), where P is the probability that the observed match is a random event.
Individual ions scores > 38 indicate identity or extensive homology (p<0.05).
Protein scores are derived from ions scores as a non-probabilistic basis for ranking protein hits.

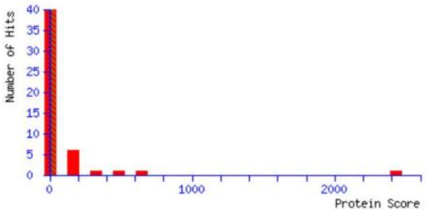

Peptide Summary Report

Format As: Peptide Summary | Help

Significance threshold p< 0.05 | Max. number of hits AUTO

Standard scoring | MudPIT scoring | Ions score or expect cut-off 0 | Show sub-sets 0

Show pop-ups | Suppress pop-ups | Sort unassigned Decreasing Score | Require bold red

Select All | Select None | Search Selected | **Error tolerant** | Archive Report

1. A2MG_HUMAN **Mass:** 164614 **Score:** 2428 **Matches:** 108(78) **Sequences:** 50(37) **emPAI:** 1.98
Alpha-2-macroglobulin OS=Homo sapiens GN=A2M PE=1 SV=2
Check to include this hit in error tolerant search or archive report

Query	Observed	Mr(expt)	Mr(calc)	Delta	Miss	Score	Expect	Rank	Unique	Peptide
86	593.3675	592.3602	592.3584	0.0018	0	33	0.21	1	U	K.LSFVK.V
87	593.3682	592.3609	592.3584	0.0024	0	(32)	0.26	1	U	K.LSFVK.V
100	634.3947	633.3875	633.3850	0.0025	0	23	4.1	4	U	K.VFQLK.R
102	647.4263	646.4190	646.4166	0.0024	0	30	0.36	1	U	K.VIFIR.G
120	724.4377	723.4304	723.4279	0.0025	0	23	1.3	1	U	K.GVPIPNK.V
145	414.7332	827.4518	827.4501	0.0017	0	(62)	0.00039	1	U	K.SDIAPVAR.L
146	414.7337	827.4528	827.4501	0.0027	0	(62)	0.0004	1	U	K.SDIAPVAR.L
147	414.7337	827.4529	827.4501	0.0028	0	63	0.00038	1	U	K.SDIAPVAR.L
148	414.7339	827.4533	827.4501	0.0032	0	(50)	0.0064	1	U	K.SDIAPVAR.L
149	414.7340	827.4534	827.4501	0.0033	0	(62)	0.00039	1	U	K.SDIAPVAR.L
156	438.7471	875.4795	875.4752	0.0043	0	18	15	1	U	K.YNILPEK.E
157	442.2855	882.5565	882.5538	0.0027	0	(31)	0.21	1		R.DLKPAIVK.V
158	442.2860	882.5575	882.5538	0.0036	0	42	0.018	1		R.DLKPAIVK.V
168	463.2781	924.5417	924.5393	0.0025	0	33	0.18	1	U	R.TGTHGLLVK.Q
174	509.8019	1017.5892	1017.5859	0.0034	0	(46)	0.014	1	U	K.ATVLNYLPK.C
175	509.8023	1017.5901	1017.5859	0.0043	0	(43)	0.033	1	U	K.ATVLNYLPK.C
176	509.8027	1017.5909	1017.5859	0.0050	0	51	0.0051	1	U	K.ATVLNYLPK.C
184	523.7994	1045.5843	1045.5808	0.0035	0	(46)	0.021	1	U	K.FEVQVTVPK.I
185	523.7998	1045.5851	1045.5808	0.0043	0	46	0.02	1	U	K.FEVQVTVPK.I
191	542.8138	1083.6131	1083.6077	0.0054	0	35	0.16	1	U	K.GHFSISIPVK.S
192	542.8139	1083.6132	1083.6077	0.0055	0	(35)	0.16	1	U	K.GHFSISIPVK.S
199	552.3076	1102.6006	1102.5982	0.0024	0	(61)	0.00064	1	U	R.SSGSLLNNAIK.G
200	552.3081	1102.6016	1102.5982	0.0034	0	63	0.00041	1	U	R.SSGSLLNNAIK.G
203	558.8072	1115.5999	1115.5975	0.0024	0	77	1.6e-005	1	U	R.QTVSWAVTPK.S
204	558.8081	1115.6016	1115.5975	0.0040	0	(64)	0.00032	1	U	R.QTVSWAVTPK.S
207	560.8234	1119.6323	1119.6288	0.0035	0	18	11	1	U	K.SIYKPGQTVK.F
211	567.7968	1133.5790	1133.5750	0.0040	0	(56)	0.002	1	U	R.SASNMAIVDVK.M
212	567.8010	1133.5874	1133.5750	0.0123	0	(23)	4.3	1	U	R.SASNMAIVDVK.M
213	574.8164	1147.6183	1147.6138	0.0044	0	(34)	0.3	1	U	R.QGIPFFGQVR.L
214	574.8166	1147.6187	1147.6138	0.0049	0	48	0.014	1	U	R.QGIPFFGQVR.L
217	575.7932	1149.5719	1149.5700	0.0019	0	(66)	0.00016	1	U	R.SASNMAIVDVK.M + Oxidation (M)
218	575.7933	1149.5720	1149.5700	0.0020	0	(64)	0.0003	1	U	R.SASNMAIVDVK.M + Oxidation (M)
219	575.7940	1149.5734	1149.5700	0.0035	0	80	6.3e-006	1	U	R.SASNMAIVDVK.M + Oxidation (M)
229	605.8271	1209.6396	1209.6353	0.0043	0	(44)	0.022	1	U	K.LPPNVVEESAR.A
230	605.8280	1209.6414	1209.6353	0.0061	0	67	0.00012	1	U	K.LPPNVVEESAR.A
232	613.2820	1224.5494	1224.5445	0.0049	0	50	0.0022	1	U	K.YDVENCLANK.V
241	628.3274	1254.6403	1254.6357	0.0046	0	65	0.00015	1	U	K.AIGYLNTGYQR.Q
242	630.2919	1258.5693	1258.5652	0.0040	0	79	3.1e-006	1	U	R.VGFYESDVMGR.G
243	630.2923	1258.5701	1258.5652	0.0049	0	(79)	3.3e-006	1	U	R.VGFYESDVMGR.G
247	636.8419	1271.6692	1271.6656	0.0036	0	67	0.00014	1	U	R.VTAAPQSVCALR.A
248	636.8429	1271.6711	1271.6656	0.0055	0	(65)	0.00022	1	U	R.VTAAPQSVCALR.A
249	638.2889	1274.5632	1274.5602	0.0031	0	(55)	0.00058	1	U	R.VGFYESDVMGR.G + Oxidation (M)
250	638.2891	1274.5636	1274.5602	0.0035	0	(75)	5.4e-006	1	U	R.VGFYESDVMGR.G + Oxidation (M)
251	638.2893	1274.5641	1274.5602	0.0040	0	(56)	0.00049	1	U	R.VGFYESDVMGR.G + Oxidation (M)
261	669.8318	1337.6490	1337.6438	0.0053	0	56	0.0013	1	U	R.NALFCLESAWK.T
272	697.8468	1393.6791	1393.6725	0.0066	0	61	0.00042	1	U	K.NEDSLVFVQTDK.S
276	708.9170	1415.8195	1415.8210	-0.0016	0	(48)	0.0062	1		K.MVSGFIPLKPTVK.M
277	472.9471	1415.8195	1415.8210	-0.0015	0	(41)	0.029	1		K.MVSGFIPLKPTVK.M
278	709.8035	1417.5924	1417.5899	0.0026	0	36	0.018	1	U	K.HYDGSYSTFGER.Y
279	716.9181	1431.8216	1431.8160	0.0056	0	68	6.8e-005	1		K.MVSGFIPLKPTVK.M + Oxidation (M)
280	478.2812	1431.8216	1431.8160	0.0057	0	(48)	0.0065	1		K.MVSGFIPLKPTVK.M + Oxidation (M)
284	724.8219	1447.6293	1447.6363	-0.0070	0	(45)	0.0039	1	U	K.DMYSFLEDMGLK.A
285	724.8264	1447.6383	1447.6363	0.0020	0	(48)	0.0028	1	U	K.DMYSFLEDMGLK.A
286	724.8271	1447.6397	1447.6363	0.0034	0	66	3.8e-005	1	U	K.DMYSFLEDMGLK.A
289	746.4066	1490.7987	1490.7882	0.0105	0	62	0.00043	1	U	R.NQGNTWLTAFVLK.T
291	756.3924	1510.7702	1510.7627	0.0075	0	45	0.015	1	U	K.AAQVTIQSSGTFSSK.F
293	515.9406	1544.7999	1544.7947	0.0052	0	(41)	0.051	1	U	R.LVHVEEPHTETVR.K

Query	Observed	Mr(expt)	Mr(calc)	Delta	Miss	Score	Expect	Rank	Unique	Peptide
294	515.9406	1544.8001	1544.7947	0.0054	0	(41)	0.047	1	U	R.LVHVEEPHTETVR.K
295	515.9408	1544.8005	1544.7947	0.0058	0	(44)	0.025	1	U	R.LVHVEEPHTETVR.K
296	515.9408	1544.8006	1544.7947	0.0059	0	(40)	0.067	1	U	R.LVHVEEPHTETVR.K
297	515.9408	1544.8007	1544.7947	0.0060	0	(51)	0.0052	1	U	R.LVHVEEPHTETVR.K
298	515.9409	1544.8009	1544.7947	0.0062	0	(41)	0.048	1	U	R.LVHVEEPHTETVR.K
299	515.9409	1544.8010	1544.7947	0.0063	0	(46)	0.015	1	U	R.LVHVEEPHTETVR.K
300	515.9410	1544.8011	1544.7947	0.0064	0	(40)	0.055	1	U	R.LVHVEEPHTETVR.K
301	515.9410	1544.8012	1544.7947	0.0065	0	56	0.0017	1	U	R.LVHVEEPHTETVR.K
302	802.9274	1603.8403	1603.8358	0.0045	0	(78)	8.1e-006	1	U	R.IAQWQSFQLEGGLK.Q
303	802.9280	1603.8415	1603.8358	0.0057	0	90	5.3e-007	1	U	R.IAQWQSFQLEGGLK.Q
306	540.9480	1619.8221	1619.8168	0.0052	0	(9)	66	1	U	K.DNSVHWERPQKPK.A
307	540.9485	1619.8237	1619.8168	0.0069	0	11	45	1	U	K.DNSVHWERPQKPK.A
308	540.9486	1619.8240	1619.8168	0.0072	0	(2)	3.8e+002	2	U	K.DNSVHWERPQKPK.A
313	836.9364	1671.8583	1671.8508	0.0074	0	51	0.0042	1	U	R.TEHPFTVEEFVLPK.F
314	836.9364	1671.8583	1671.8508	0.0075	0	(23)	2.5	1	U	R.TEHPFTVEEFVLPK.F
315	558.2934	1671.8583	1671.8508	0.0075	0	(41)	0.041	1	U	R.TEHPFTVEEFVLPK.F
320	849.4269	1696.8393	1696.8342	0.0051	0	(84)	1.7e-006	1	U	K.SSSNEEVMFLTVQVK.G
321	857.4255	1712.8365	1712.8291	0.0074	0	92	2.7e-007	1	U	K.SSSNEEVMFLTVQVK.G + Oxidation (M)
322	577.9651	1730.8736	1730.8662	0.0075	1	18	8.5	1	U	K.QEDMKGHFSISIPVK.S + Oxidation (M)
331	594.0108	1779.0106	1779.0029	0.0077	0	60	0.00021	1	U	K.DTVIKPLLVEPEGLEK.E
332	594.0112	1779.0117	1779.0029	0.0088	0	(60)	0.00022	1	U	K.DTVIKPLLVEPEGLEK.E
333	890.5132	1779.0117	1779.0029	0.0088	0	(40)	0.022	1	U	K.DTVIKPLLVEPEGLEK.E
343	923.0253	1844.0360	1844.0295	0.0065	0	(62)	0.00016	1	U	R.LLIYAVLPTGDVIGDSAK.Y
344	923.0265	1844.0385	1844.0295	0.0090	0	68	4.6e-005	1	U	R.LLIYAVLPTGDVIGDSAK.Y
350	942.5318	1883.0490	1883.0404	0.0086	0	97	5.7e-008	1	U	R.VSVQLEASPAFLAVPVEK.E
354	633.9673	1898.8801	1898.8734	0.0067	0	28	0.41	1	U	K.FSGQLNSHGCFYQQVK.T
356	636.7091	1907.1055	1907.0979	0.0076	1	65	3.7e-005	1	U	R.KDTVIKPLLVEPEGLEK.E
364	683.6918	2048.0535	2048.0439	0.0096	0	(30)	0.51	1	U	K.VDLSFSPSQSLPASHAHLR.V
365	683.6921	2048.0544	2048.0439	0.0105	0	55	0.0013	1	U	K.VDLSFSPSQSLPASHAHLR.V
366	513.0209	2048.0545	2048.0439	0.0105	0	(28)	0.69	1	U	K.VDLSFSPSQSLPASHAHLR.V
370	1042.0110	2082.0074	2082.0052	0.0023	0	(65)	0.00011	1	U	K.AGAFCLSEDAGLGISSTASLR.A
371	695.0136	2082.0191	2082.0052	0.0140	0	(49)	0.0044	1	U	K.AGAFCLSEDAGLGISSTASLR.A
372	1042.0168	2082.0191	2082.0052	0.0140	0	95	1.3e-007	1	U	K.AGAFCLSEDAGLGISSTASLR.A
374	697.3161	2088.9266	2088.9179	0.0087	0	(11)	7.9	1	U	K.MCPQLQQYEMHGPEGLR.V + Oxidation (M)
375	697.3164	2088.9275	2088.9179	0.0096	0	(49)	0.0013	1	U	K.MCPQLQQYEMHGPEGLR.V + Oxidation (M)
376	697.3167	2088.9284	2088.9179	0.0105	0	65	3.9e-005	1	U	K.MCPQLQQYEMHGPEGLR.V + Oxidation (M)
377	702.6472	2104.9198	2104.9129	0.0070	0	(40)	0.0088	1	U	K.MCPQLQQYEMHGPEGLR.V + 2 Oxidation (M)
378	704.0331	2109.0775	2109.0702	0.0073	0	90	3.6e-007	1	U	K.LHTEAQIQEEGTVVELTGR.Q
380	713.0271	2136.0595	2136.0487	0.0108	0	47	0.0072	1	U	R.HNVYINGITYTPVSSTNEK.D
411	796.4062	2386.1966	2386.1877	0.0089	0	87	7.9e-007	1	U	K.QQNAQGGFSSTQDTVVALHALSK.Y
412	796.4067	2386.1982	2386.1877	0.0105	0	(85)	1.1e-006	1	U	K.QQNAQGGFSSTQDTVVALHALSK.Y
413	1199.0802	2396.1459	2396.1417	0.0042	0	(13)	14	1	U	K.ETTFNSLLCPSGGEVSEELSLK.L
414	1199.0830	2396.1514	2396.1417	0.0097	0	48	0.0052	1	U	K.ETTFNSLLCPSGGEVSEELSLK.L
425	825.7491	2474.2253	2474.2152	0.0102	0	(34)	0.12	1	U	R.SLFTDLEAENDVLHCVAFAVPK.S
426	825.7492	2474.2257	2474.2152	0.0106	0	(33)	0.15	1	U	R.SLFTDLEAENDVLHCVAFAVPK.S
427	825.7492	2474.2259	2474.2152	0.0107	0	44	0.012	1	U	R.SLFTDLEAENDVLHCVAFAVPK.S
428	825.7497	2474.2272	2474.2152	0.0120	0	(31)	0.26	1	U	R.SLFTDLEAENDVLHCVAFAVPK.S
432	1274.5512	2547.0879	2547.0788	0.0091	0	59	4.7e-005	1	U	K.VYDYYETDEFAIAEYNAPCSK.D
446	942.8214	2825.4424	2825.4310	0.0114	0	(13)	14	1	U	R.VVSMDENFHPLNELIPLVYIQDPK.G + Oxidation (M)
447	942.8222	2825.4447	2825.4310	0.0138	0	17	6.4	1	U	R.VVSMDENFHPLNELIPLVYIQDPK.G + Oxidation (M)
452	978.5149	2932.5228	2932.5103	0.0125	0	23	1.3	1	U	R.AVDQSVLLMKPDAELSASSVYNLLPEK.D + Oxidation (M)

---

2. **TRYP_PIG** **Mass:** 25078 **Score:** 571 **Matches:** 19(15) **Sequences:** 5(5) **emPAI:** 1.88

Trypsin OS=Sus scrofa PE=1 SV=1

Check to include this hit in error tolerant search or archive report

Query	Observed	Mr(expt)	Mr(calc)	Delta	Miss	Score	Expect	Rank	Unique	Peptide
150	421.7595	841.5045	841.5022	0.0024	0	51	0.005	1	U	R.VATVSLPR.S
151	421.7602	841.5059	841.5022	0.0038	0	(51)	0.0049	1	U	R.VATVSLPR.S
388	737.7088	2210.1046	2210.0967	0.0079	0	(102)	2.5e-008	1	U	R.LGEHNIDVLEGNEQFINAAK.I
389	1106.0609	2210.1073	2210.0967	0.0106	0	(33)	0.18	1	U	R.LGEHNIDVLEGNEQFINAAK.I
390	737.7097	2210.1073	2210.0967	0.0106	0	110	4.3e-009	1	U	R.LGEHNIDVLEGNEQFINAAK.I
391	1106.0617	2210.1088	2210.0967	0.0121	0	(47)	0.0073	1	U	R.LGEHNIDVLEGNEQFINAAK.I
392	737.7102	2210.1088	2210.0967	0.0121	0	(108)	6.5e-009	1	U	R.LGEHNIDVLEGNEQFINAAK.I
393	737.7118	2210.1136	2210.0967	0.0169	0	(18)	5.9	1	U	R.LGEHNIDVLEGNEQFINAAK.I
400	761.7348	2282.1825	2282.1729	0.0096	0	68	5.4e-005	1	U	K.IITHPNFNGNTLDNDIMLIK.L
401	761.7350	2282.1833	2282.1729	0.0104	0	(49)	0.0046	1	U	K.IITHPNFNGNTLDNDIMLIK.L
404	761.7358	2282.1855	2282.1729	0.0126	0	(66)	9.1e-005	1	U	K.IITHPNFNGNTLDNDIMLIK.L
407	767.0669	2298.1789	2298.1678	0.0111	0	(55)	0.0011	1	U	K.IITHPNFNGNTLDNDIMLIK.L + Oxidation (M)
408	767.0677	2298.1812	2298.1678	0.0134	0	(52)	0.0025	1	U	K.IITHPNFNGNTLDNDIMLIK.L + Oxidation (M)
439	677.6125	2706.4209	2706.4089	0.0120	1	52	0.0015	1	U	R.IQVRLGEHNIDVLEGNEQFINAAK.I
440	903.1476	2706.4209	2706.4089	0.0121	1	(25)	0.74	1	U	R.IQVRLGEHNIDVLEGNEQFINAAK.I
467	828.1908	3308.7341	3308.7187	0.0155	1	(56)	0.00042	1	U	K.IITHPNFNGNTLDNDIMLIKLSSPATLNSR.V
468	828.1916	3308.7374	3308.7187	0.0187	1	(48)	0.0024	1	U	K.IITHPNFNGNTLDNDIMLIKLSSPATLNSR.V
470	828.1920	3308.7390	3308.7187	0.0203	1	72	9.6e-006	1	U	K.IITHPNFNGNTLDNDIMLIKLSSPATLNSR.V
471	832.1914	3324.7367	3324.7136	0.0231	1	(1)	1.5e+002	1	U	K.IITHPNFNGNTLDNDIMLIKLSSPATLNSR.V + Oxidation (M)

---

3. **K2C1_HUMAN** **Mass:** 66170 **Score:** 507 **Matches:** 16(13) **Sequences:** 9(7) **emPAI:** 0.58

Keratin, type II cytoskeletal 1 OS=Homo sapiens GN=KRT1 PE=1 SV=6

Check to include this hit in error tolerant search or archive report

Query	Observed	Mr(expt)	Mr(calc)	Delta	Miss	Score	Expect	Rank	Unique	Peptide
173	500.2273	998.4399	998.4379	0.0021	0	13	12	1	U	K.DVDGAYMTK.V
178	517.2632	1032.5118	1032.5087	0.0031	0	41	0.055	1	U	R.TLLEGEESR.M
188	533.2652	1064.5158	1064.5138	0.0020	0	(21)	4.7	1		K.AQYEDIAQK.S
189	533.2657	1064.5169	1064.5138	0.0031	0	30	0.63	1		K.AQYEDIAQK.S
221	590.3056	1178.5966	1178.5931	0.0035	0	61	0.00057	1		K.YEELQITAGR.H
222	590.3060	1178.5974	1178.5931	0.0042	0	(55)	0.0021	1		K.YEELQITAGR.H
253	651.8646	1301.7145	1301.7078	0.0067	0	69	9.1e-005	1		R.SLDLDSIIAEVK.A
266	692.3514	1382.6882	1382.6830	0.0052	0	(62)	0.00031	1	U	K.SLNNQFASFIDK.V
267	692.3518	1382.6890	1382.6830	0.0060	0	65	0.00014	1	U	K.SLNNQFASFIDK.V
269	697.3727	1392.7308	1392.7249	0.0059	1	(72)	3.8e-005	1		R.TNAENEFVTIKK.D
270	697.3729	1392.7312	1392.7249	0.0064	1	(78)	8.6e-006	1		R.TNAENEFVTIKK.D
271	465.2510	1392.7313	1392.7249	0.0064	1	(43)	0.026	1		R.TNAENEFVTIKK.D
115	697.3729	1392.7313	1392.7249	0.0065	1	86	3.6e-006	1		R.TNAENEFVTIKK.D
287	738.3929	1474.7712	1474.7780	-0.0067	0	(72)	4.4e-005	1		R.FLEQQNQVLQTK.W
288	738.3988	1474.7831	1474.7780	0.0051	0	92	4.2e-007	1		R.FLEQQNQVLQTK.W
155	858.9336	1715.8526	1715.8438	0.0088	0	106	1.4e-008	1	U	K.QISNLQQSISDAEQR.G

Proteins matching the same set of peptides:

K2C1_PANTR **Mass:** 65621 **Score:** 507 **Matches:** 16(13) **Sequences:** 9(7)

Keratin, type II cytoskeletal 1 OS=Pan troglodytes GN=KRT1 PE=2 SV=1

---

4. **CFAH_HUMAN** **Mass:** 143680 **Score:** 325 **Matches:** 17(11) **Sequences:** 14(10) **emPAI:** 0.36

Complement factor H OS=Homo sapiens GN=CFH PE=1 SV=4

Check to include this hit in error tolerant search or archive report

Query	Observed	Mr(expt)	Mr(calc)	Delta	Miss	Score	Expect	Rank	Unique	Peptide
166	463.2310	924.4475	924.4454	0.0021	0	(33)	0.22	1	U	R.NGFYPATR.G
167	463.2316	924.4486	924.4454	0.0033	0	39	0.059	1	U	R.NGFYPATR.G
187	532.3002	1062.5858	1062.5822	0.0036	0	60	0.00075	1	U	K.IDVHLVPDR.K
223	593.3017	1184.5888	1184.5860	0.0029	0	52	0.0026	1	U	K.CLPVTAPENGK.I
224	593.8001	1185.5857	1185.5819	0.0039	1	45	0.018	1	U	R.TKNDFTWFK.L

225	595.2715	1188.5285	1188.5234	0.0051	0	49	0.003	1	U	K.LSYTCEGGFR.I
239	621.7767	1241.5388	1241.5347	0.0041	0	28	0.25	1	U	R.TGDEITYQCR.N
240	623.3078	1244.6010	1244.5972	0.0038	0	64	0.00018	1	U	K.CTSTGWIPAPR.C
244	630.3283	1258.6421	1258.6380	0.0041	0	47	0.015	1	U	R.WQSIPLCVEK.I
254	655.3075	1308.6004	1308.5955	0.0050	0	38	0.057	1	U	K.SCDIPVFMNAR.T
262	671.3550	1340.6954	1340.6936	0.0018	0	(48)	0.0091	1	U	K.SPDVINGSPISQK.I
263	671.3568	1340.6991	1340.6936	0.0055	0	56	0.0013	1	U	K.SPDVINGSPISQK.I
265	691.3430	1380.6715	1380.6707	0.0008	0	17	10	1	U	R.EIMENYNIALR.W + Oxidation (M)
273	698.3990	1394.7834	1394.7769	0.0066	0	72	2.4e-005	1	U	K.SSNLIILEEHLK.N
363	1010.9986	2019.9826	2019.9684	0.0142	0	51	0.003	1	U	R.DTSCVNPPTVQNAYIVSR.Q
386	552.7683	2207.0440	2207.0429	0.0011	1	(0)	2.5e+002	1	U	K.IVSSAMEPDREYHFGQAVR.F + Oxidation (M)
387	552.7707	2207.0538	2207.0429	0.0108	1	31	0.22	1	U	K.IVSSAMEPDREYHFGQAVR.F + Oxidation (M)

5. K1C10_HUMAN **Mass:** 59020 **Score:** 214 **Matches:** 16(11) **Sequences:** 8(4) **emPAI:** 0.26
Keratin, type I cytoskeletal 10 OS=Homo sapiens GN=KRT10 PE=1 SV=6
Check to include this hit in error tolerant search or archive report

Query	Observed	Mr(expt)	Mr(calc)	Delta	Miss	Score	Expect	Rank	Unique	Peptide
133	404.2046	806.3947	806.3923	0.0024	0	(47)	0.018	1		R.LAADDFR.L
134	404.2046	806.3947	806.3923	0.0024	0	(50)	0.0081	1		R.LAADDFR.L
135	404.2047	806.3948	806.3923	0.0026	0	53	0.0047	1		R.LAADDFR.L
136	405.2248	808.4350	808.4330	0.0020	0	(12)	47	2		R.LASYLDK.V
137	405.2250	808.4354	808.4330	0.0023	0	35	0.22	1		R.LASYLDK.V
177	516.3039	1030.5932	1030.5910	0.0022	0	19	7.9	1		R.VLDELTLTK.A
193	545.7700	1089.5254	1089.5237	0.0017	0	(55)	0.0021	1		K.VTMQNLNDR.L
194	545.7701	1089.5256	1089.5237	0.0019	0	(54)	0.0024	1		K.VTMQNLNDR.L
195	545.7705	1089.5265	1089.5237	0.0028	0	71	4.8e-005	1		K.VTMQNLNDR.L
196	545.7707	1089.5268	1089.5237	0.0031	0	(55)	0.0021	1		K.VTMQNLNDR.L
202	555.2499	1108.4852	1108.4825	0.0027	0	20	2	1	U	K.DAEAWFNEK.S
236	617.8460	1233.6775	1233.6717	0.0058	1	(62)	0.00039	1	U	R.LKYENEVALR.Q
237	617.8461	1233.6777	1233.6717	0.0060	1	(44)	0.025	1	U	R.LKYENEVALR.Q
238	617.8475	1233.6804	1233.6717	0.0087	1	77	1.2e-005	1	U	R.LKYENEVALR.Q
246	631.8044	1261.5942	1261.5899	0.0043	0	50	0.0039	1	U	R.SLLEGEGSSGGGGR.G
281	478.9294	1433.7663	1433.7626	0.0036	1	13	32	1	U	K.IRLENEIQTYR.S

6. K1C14_HUMAN **Mass:** 51872 **Score:** 167 **Matches:** 10(8) **Sequences:** 4(3) **emPAI:** 0.21
Keratin, type I cytoskeletal 14 OS=Homo sapiens GN=KRT14 PE=1 SV=4
Check to include this hit in error tolerant search or archive report

Query	Observed	Mr(expt)	Mr(calc)	Delta	Miss	Score	Expect	Rank	Unique	Peptide
133	404.2046	806.3947	806.3923	0.0024	0	(47)	0.018	1		R.LAADDFR.T
134	404.2046	806.3947	806.3923	0.0024	0	(50)	0.0081	1		R.LAADDFR.T
135	404.2047	806.3948	806.3923	0.0026	0	53	0.0047	1		R.LAADDFR.T
136	405.2248	808.4350	808.4330	0.0020	0	(12)	47	2		R.LASYLDK.V
137	405.2250	808.4354	808.4330	0.0023	0	35	0.22	1		R.LASYLDK.V
193	545.7700	1089.5254	1089.5237	0.0017	0	(55)	0.0021	1		K.VTMQNLNDR.L
194	545.7701	1089.5256	1089.5237	0.0019	0	(54)	0.0024	1		K.VTMQNLNDR.L
195	545.7705	1089.5265	1089.5237	0.0028	0	71	4.8e-005	1		K.VTMQNLNDR.L
196	545.7707	1089.5268	1089.5237	0.0031	0	(55)	0.0021	1		K.VTMQNLNDR.L
252	651.3353	1300.6560	1300.6510	0.0050	0	68	9.2e-005	1		R.ALEEANADLEVK.I

Proteins matching the same set of peptides:
K1C16_HUMAN **Mass:** 51578 **Score:** 167 **Matches:** 10(8) **Sequences:** 4(3)
Keratin, type I cytoskeletal 16 OS=Homo sapiens GN=KRT16 PE=1 SV=4

7. K22E_HUMAN **Mass:** 65678 **Score:** 162 **Matches:** 6(5) **Sequences:** 4(3) **emPAI:** 0.17
Keratin, type II cytoskeletal 2 epidermal OS=Homo sapiens GN=KRT2 PE=1 SV=2
Check to include this hit in error tolerant search or archive report

Query	Observed	Mr(expt)	Mr(calc)	Delta	Miss	Score	Expect	Rank	Unique	Peptide
144	414.2184	826.4222	826.4225	-0.0003	0	14	21	3		K.FASFIDK.V
201	554.2762	1106.5379	1106.5356	0.0023	0	45	0.023	1		K.AQYEEIAQR.S
258	665.3691	1328.7237	1328.7187	0.0050	0	(44)	0.029	1		R.NLDLDSIIAEVK.A
259	665.3692	1328.7238	1328.7187	0.0050	0	64	0.00026	1		R.NLDLDSIIAEVK.A
287	738.3929	1474.7712	1474.7780	-0.0067	0	(72)	4.4e-005	1		R.FLEQQNQVLQTK.W
288	738.3988	1474.7831	1474.7780	0.0051	0	92	4.2e-007	1		R.FLEQQNQVLQTK.W

8. F13B_HUMAN **Mass:** 77742 **Score:** 140 **Matches:** 8(4) **Sequences:** 6(2) **emPAI:** 0.19
Coagulation factor XIII B chain OS=Homo sapiens GN=F13B PE=1 SV=3
Check to include this hit in error tolerant search or archive report

Query	Observed	Mr(expt)	Mr(calc)	Delta	Miss	Score	Expect	Rank	Unique	Peptide
226	598.8054	1195.5963	1195.5913	0.0049	0	63	0.00027	1	U	R.IAQYYYTFK.S
274	468.8771	1403.6096	1403.6041	0.0055	0	28	0.18	1	U	R.CFDHHFLEGSR.E
275	708.3753	1414.7360	1414.7279	0.0081	0	29	0.87	1	U	K.VLHGDLIDFVCK.Q
319	848.9168	1695.8190	1695.8138	0.0052	0	4	1.6e+002	1	U	K.CTKPDLSNGYISDVK.L
367	695.0040	2081.9903	2081.9840	0.0063	0	(50)	0.0033	1	U	K.VACEEPPFIENGAANLHSK.I
368	695.0051	2081.9934	2081.9840	0.0094	0	(40)	0.028	1	U	K.VACEEPPFIENGAANLHSK.I
369	695.0051	2081.9935	2081.9840	0.0095	0	57	0.00066	1	U	K.VACEEPPFIENGAANLHSK.I
438	899.7417	2696.2034	2696.1807	0.0227	1	20	0.77	1	U	K.TTGGKDEEVVQCLSDGWSSQPTCR.K

9. K1C9_HUMAN **Mass:** 62255 **Score:** 136 **Matches:** 9(4) **Sequences:** 6(4) **emPAI:** 0.31
Keratin, type I cytoskeletal 9 OS=Homo sapiens GN=KRT9 PE=1 SV=3
Check to include this hit in error tolerant search or archive report

Query	Observed	Mr(expt)	Mr(calc)	Delta	Miss	Score	Expect	Rank	Unique	Peptide
136	405.2248	808.4350	808.4330	0.0020	0	(12)	47	2		R.LASYLDK.V
137	405.2250	808.4354	808.4330	0.0023	0	35	0.22	1		R.LASYLDK.V
186	530.7868	1059.5590	1059.5560	0.0030	0	68	0.00013	1	U	K.TLLDIDNTR.M
208	561.2975	1120.5805	1120.5764	0.0041	0	(12)	47	1	U	R.QEYEQLIAK.N
209	561.2977	1120.5808	1120.5764	0.0044	0	42	0.048	1	U	R.QEYEQLIAK.N
342	613.3300	1836.9682	1836.9581	0.0101	0	32	0.27	1	U	R.HGVQELEIELQSQLSK.K
430	837.3877	2509.1413	2509.1245	0.0168	0	49	0.0014	1	U	K.EIETYHNLLEGGQEDFESSGAGK.I
463	1088.8475	3263.5206	3263.5066	0.0141	0	43	0.0068	1	U	K.DIENQYETQITQIEHEVSSSGQEVQSSAK.E
464	1088.8481	3263.5224	3263.5066	0.0158	0	(31)	0.092	1	U	K.DIENQYETQITQIEHEVSSSGQEVQSSAK.E

10. ALBU_FELCA **Mass:** 70611 **Score:** 117 **Matches:** 3(3) **Sequences:** 2(2) **emPAI:** 0.10
Serum albumin OS=Felis catus GN=ALB PE=1 SV=1
Check to include this hit in error tolerant search or archive report

Query	Observed	Mr(expt)	Mr(calc)	Delta	Miss	Score	Expect	Rank	Unique	Peptide
215	575.3127	1148.6108	1148.6077	0.0031	0	64	0.00028	1	U	K.LVNEVTEFAK.G

	Query	Observed	Mr(expt)	Mr(calc)	Delta	Miss	Score	Expect	Rank	Unique	Peptide
☑	216	575.3130	1148.6114	1148.6077	0.0037	0	(46)	0.018	1	U	K.LVNEVTEFAK.G
☑	309	547.3196	1638.9370	1638.9305	0.0065	1	63	0.00014	1	U	K.KVPQVSTPTLVEVSR.S

Proteins matching the same set of peptides:

ALBU_MACFA **Mass:** 70825 **Score:** 117 **Matches:** 3(3) **Sequences:** 2(2)
Serum albumin OS=Macaca fascicularis GN=ALB PE=2 SV=1

ALBU_MACMU **Mass:** 69832 **Score:** 117 **Matches:** 3(3) **Sequences:** 2(2)
Serum albumin (Fragment) OS=Macaca mulatta GN=ALB PE=2 SV=1

ALBU_HUMAN **Mass:** 71317 **Score:** 117 **Matches:** 3(3) **Sequences:** 2(2)
Serum albumin OS=Homo sapiens GN=ALB PE=1 SV=2

ALBU_PONAB **Mass:** 71317 **Score:** 117 **Matches:** 3(3) **Sequences:** 2(2)
Serum albumin OS=Pongo abelii GN=ALB PE=2 SV=1

---

11. F13A_HUMAN **Mass:** 83728 **Score:** 66 **Matches:** 5(2) **Sequences:** 3(2) **emPAI:** 0.13
Coagulation factor XIII A chain OS=Homo sapiens GN=F13A1 PE=1 SV=4
☐ Check to include this hit in error tolerant search or archive report

	Query	Observed	Mr(expt)	Mr(calc)	Delta	Miss	Score	Expect	Rank	Unique	Peptide
☑	256	663.9180	1325.8215	1325.8170	0.0045	0	34	0.062	1	U	K.STVLTIPEIIIK.V
☑	257	663.9191	1325.8236	1325.8170	0.0066	0	(33)	0.078	1	U	K.STVLTIPEIIIK.V
☑	316	844.4707	1686.9269	1686.9192	0.0076	0	24	1.8	1	U	K.GTYIPVPIVSELQSGK.W
☑	317	564.6186	1690.8340	1690.8275	0.0065	0	44	0.02	1		K.DGTHVVENVDATHIGK.L
☑	318	564.6189	1690.8348	1690.8275	0.0074	0	(35)	0.17	1		K.DGTHVVENVDATHIGK.L

Peptide matches not assigned to protein hits: (no details means no match)

	Query	Observed	Mr(expt)	Mr(calc)	Delta	Miss	Score	Expect	Rank	Unique	Peptide
☑	100	634.3947	633.3875	633.3850	0.0025	0	28	1.4	1		VFGAIK
☑	182	523.7800	1045.5455	1045.4975	0.0480	0	23	4.8	1		LSSAPCQQR
☑	183	523.7813	1045.5480	1045.5404	0.0076	1	19	10	1		LREGTLDDK
☑	144	414.2184	826.4222	826.4225	-0.0003	0	18	7.3	1		FSAFLDK
☑	136	405.2248	808.4350	808.4443	-0.0093	0	15	24	1		YSAIISR
☑	456	517.2657	3097.5507	3097.5396	0.0110	2	11	17	1		EGVPRFYIRLLSDLDDYLNELWEDK
☑	359	646.3284	1935.9635	1935.9845	-0.0210	0	11	34	1		ARPFLGICVGMQLMATR + Oxidation (M)
☑	153	846.4453	845.4380	845.4495	-0.0115	0	11	90	1		DTVVEVGK
☑	152	846.4444	845.4371	845.4681	-0.0310	0	11	92	1		AMLVDVAK
☑	383	719.6875	2156.0408	2156.0401	0.0007	0	11	29	1		LPGLENWTYLDCLPYFR
☑	292	506.3152	1515.9237	1515.9024	0.0213	2	10	9.9	1		KAPVKLESIASVFK
☑	154	846.4454	845.4382	845.4793	-0.0411	1	10	1.3e+002	1		MAEKVLR
☑	349	628.3278	1881.9616	1881.9627	-0.0011	2	9	50	1		LMSFKMIDDILMRNR
☑	415	800.4239	2398.2500	2398.2413	0.0086	1	9	41	1		VVGIKNGEIIDMDIDEALAVER
☑	339	609.9452	1826.8137	1826.8469	-0.0332	0	9	20	1		QPIDPNQEITEICDR
☑	308	540.9486	1619.8240	1619.8375	-0.0134	2	8	83	1		NMLNSVKAMKVEPK + 2 Oxidation (M)
☑	405	762.0637	2283.1692	2283.1470	0.0222	0	8	57	1		LQAIEPSGNYHILHAYCGIK
☑	361	665.3670	1993.0791	1993.0857	-0.0066	2	8	56	1		RVFALIANLSQRYSAER
☑	510	671.3542	4022.0814	4022.0625	0.0188	2	8	20	1		GELALIESIPLMMKETSTVCSSINVASTKAGINLDAVK + 2 Oxidation (M)
☑	449	579.2959	2891.4429	2891.4196	0.0233	2	7	54	1		GLPYGSGMASSRNKRPSTQVGGLTDPDK + Oxidation (M)
☑	48	544.3339	543.3267	543.3090	0.0176	0	7	3.1e+002	1		APLML
☑	547	887.9597	5321.7147	5321.7614	-0.0467	2	6	12	1		GPTITITAATAAAAATAMVSVDPEELRGLSPSIMQPCHFLTLTPIRIPFR + 2 Oxidation (M)
☑	346	925.4492	1848.8839	1848.8974	-0.0136	2	6	94	1		AYKVYRGMGSLGAMSSR + Oxidation (M)
☑	512	692.3660	4148.1522	4148.1245	0.0277	1	6	25	1		LSPVLEVVTNIDLEHLDYYTDIEHIKETFLSFIDK
☑	327	883.8943	1765.7739	1765.7723	0.0016	1	6	31	1		DAVQNCCGISKTEER
☑	481	605.8194	3628.8729	3628.8950	-0.0221	2	6	46	1		IVGDEKEGISPTGTYATMFLIGELIGEKSSIFVK
☑	329	590.9314	1769.7724	1769.8220	-0.0496	0	6	29	1		LYPEDAESQPLTHDR
☑	493	765.4146	3822.0367	3822.0520	-0.0153	1	5	25	1		FKIIVSFFCDHVFLSVIWLISCVLMIFFTLK
☑	474	579.3000	3469.7566	3469.7625	-0.0060	2	5	54	1		VCNDILSVIDKHLIPSSNAVESTVFFYKMK + Oxidation (M)
☑	532	4862.6215	4861.6142	4861.5787	0.0355	2	5	7.2	1		CGVELSEIDQSGPKLRFSQVITGKPTASLLPTIVEDSLNDLPIPK
☑	382	719.6870	2156.0393	2156.0597	-0.0204	0	5	1e+002	1		APVSPALASPSSEVSDVSEATR
☑	345	923.5281	1845.0416	1845.0036	0.0380	1	5	91	1		GYPPAAPYDKIIVTAAAK
☑	533	697.3425	4874.3464	4874.3783	-0.0319	1	4	26	1		VGAAILGDEYLWSGGVIPYTFAGVSGADQSAILSGMQELEEKTCIR + Oxidation (M)
☑	505	654.3517	3920.0665	3920.0320	0.0345	2	4	36	1		ENISDQDLSLLGFALNSGRSIVIAVNKWDGLSFDVK
☑	492	633.3247	3793.9046	3793.8944	0.0102	1	4	62	1		DQANDGLSSALLILYLDSARSLPNNPLEINHDGMK
☑	358	646.3280	1935.9621	1935.9169	0.0452	0	4	1.8e+002	1		IVGDDVAIMMVEAEATEK + Oxidation (M)
☑	431	844.4792	2530.4158	2530.3795	0.0363	2	4	45	1		QKLVAILDFQPLVQDKEYLDR
☑	524	783.4013	4694.3640	4694.3479	0.0161	2	3	38	1		LAIASIGKLMFAQFSELVNDFYNNGLPSNLTGGRNPSLDYGFK + Oxidation (M)
☑	394	738.0368	2211.0885	2211.1027	-0.0142	2	3	1.6e+002	1		DEIISFMDTIINCSAKAKR
☑	434	515.3032	2571.4796	2571.4366	0.0430	2	3	22	1		VIWSPFIDKYSIPFLGVKLGHR
☑	502	651.8634	3905.1369	3905.0873	0.0496	2	3	22	1		QMVRLAWRDFLGYASLENSLLDLSALAEALIISAR
☑	336	595.2628	1782.7665	1782.7916	-0.0252	0	3	52	1		YSCSTNLPQPAEMASK
☑	459	633.3083	3161.5051	3161.5202	-0.0151	1	3	1.1e+002	1		AFLQATFLSCGSITNPEKMYHLEFDVK + Oxidation (M)
☑	473	670.3431	3346.6791	3346.6656	0.0135	1	2	1.2e+002	1		EMHIELVREEILFDTVHVTFQLTFDNR + Oxidation (M)
☑	442	925.4552	2773.3437	2773.3705	-0.0268	1	2	1.4e+002	1		GGHLVSIESSGEADFVAQLISENMKR
☑	448	578.2759	2886.3434	2886.3090	0.0343	0	2	80	1		VMQHTPDAEGVSQSEQEATADWSLVR + Oxidation (M)
☑	433	854.1200	2559.3381	2559.3366	0.0015	2	2	1.8e+002	1		HISKSMEEILDEVRYLVSQGVK
☑	501	651.8574	3905.1010	3905.0749	0.0261	1	2	38	1		FSAHWWCSWVAIVTGISIAILAPIGGMRHIILSAR + Oxidation (M)
☑	480	515.3016	3600.0601	3600.0775	-0.0175	0	2	7.4	1		IAPFVLPLAVTIFVFILISNWLAVLPVQYTDK
☑	526	593.8032	4742.3673	4742.3984	-0.0311	1	2	58	1		QEMTPMATIITMLSLLSLFFYLRLAYHSTITLPPNSSNHMK + 2 Oxidation (M)
☑	531	691.3244	4832.2201	4832.2455	-0.0254	1	1	23	1		DVDGFHPMNTGNLWNGRPQMVPCTPAGIMEILREYNVELEGK + 3 Oxidation (M)
☑	399	755.3482	2263.0229	2263.0209	0.0020	2	1	96	1		KLSMTCRSHLDEEEEQQK + Oxidation (M)
☑	537	827.8912	4961.3037	4961.3357	-0.0320	1	1	30	1		DAGAPTYMYEFQYRPSFSSDMKPKTVIGDHGDELFSVFGAPFLK + Oxidation (M)
☑	555	5572.8778	5571.8705	5571.8786	-0.0081	0	1	25	1		AVGTNSTLHYVWSSLGPLAVVMVATNTPHSTLSVNWSLLLSPEPDGGLMVLPK + Oxidation (M)
☑	488	746.4005	3726.9663	3726.9702	-0.0038	2	1	82	1		RPMGRILDPLRLMGVEIEATHGDHLPLTLYGPK + 2 Oxidation (M)
☑	441	925.4399	2773.2978	2773.3424	-0.0446	0	1	1.4e+002	1		LCIQLCGMCNTLVLSPSIYVFNR + Oxidation (M)
☑	556	702.9716	5615.7144	5615.7448	-0.0304	2	1	34	1		LDMFEHISLMTLDSLQKCIFSFDSHCQERPSEYIATILELSALVEKR + Oxidation (M)
☑	552	679.3555	5426.7857	5426.7880	-0.0023	1	1	32	1		AGNSTKITELLEINNSVIINTLDCNIFTCLDSIENEILFDIQLYILK
☑	451	418.2357	2920.5989	2920.6183	-0.0194	2	1	91	1		ITSGIYAYLPLMWRVIQKINCIIR
☑	487	617.7829	3700.6539	3700.6688	-0.0149	2	1	34	1		MSAEETASDSTSAEIIEIVGKTGMHGEAMQVKCR + 3 Oxidation (M)
☑	385	721.6717	2161.9934	2161.9797	0.0137	1	1	1.6e+002	1		VTEEGGGEDEMRQAVEDALK
☑	348	941.4914	1880.9682	1880.9535	0.0147	2	1	4.1e+002	1		LLAGFMGVMNNMRKQR + Oxidation (M)
☑	119	718.4965	717.4893	717.4497	0.0396	2	1	72	1		RTKSVK
☑	341	611.6497	1831.9273	1831.9527	-0.0254	1	1	4.3e+002	1		VSQLESLNKQSELETK
☑	381	719.6866	2156.0378	2156.0597	-0.0219	0	0	2.9e+002	1		APVSPALASPSSEVSDVSEATR
☑	529	802.4306	4808.5401	4808.5334	0.0067	1	0	37	1		RLIWTMIGISFCFAALMCIIPFGTTYFIIVLNLSTLQLAER + Oxidation (M)
☑	553	683.3515	5458.7536	5458.7226	0.0309	1	0	41	1		MSLPPHLCVPIDILMIDNFDSFTWNLYQSLCLLGADVTVIRNDAIPR + Oxidation (M)
☑	312	553.2596	1656.7570	1656.7413	0.0157	1	0	2.3e+002	1		YSEAMKATVQGEDGR + Oxidation (M)
☑	1	407.7877	406.7804								
☑	2	408.7870	407.7797								
☑	3	408.7887	407.7814								
☑	4	415.2124	414.2051								
☑	5	415.2129	414.2056								
☑	6	415.2133	414.2060								
☑	7	415.2554	414.2481								
☑	8	415.3426	414.3353								
☑	9	415.3429	414.3356								
☑	10	416.2515	415.2442								
☑	11	430.2446	429.2374								
☑	12	430.2455	429.2382								
☑	13	430.3884	429.3811								
☑	14	432.2394	431.2321								
☑	15	432.2397	431.2325								
☑	16	432.2819	431.2746								

17	433.3536	432.3463
18	433.3537	432.3464
19	435.2728	434.2655
20	442.3377	441.3304
21	444.1167	443.1094
22	444.1176	443.1103
23	444.1191	443.1119
24	456.2813	455.2741
25	458.9448	457.9376
26	458.9449	457.9376
27	459.2812	458.2739
28	459.2820	458.2748
29	476.3074	475.3002
30	476.3082	475.3009
31	476.3084	475.3011
32	476.3087	475.3014
33	478.3145	477.3073
34	500.3074	499.3001
35	500.3075	499.3002
36	500.3725	499.3652
37	504.3030	503.2957
38	518.2830	517.2757
39	520.3342	519.3270
40	520.3347	519.3275
41	520.3348	519.3275
42	525.3216	524.3143
43	528.3648	527.3576
44	531.2788	530.2715
45	534.9819	533.9746
46	539.3373	538.3301
47	539.8208	538.8136
49	548.3295	547.3222
50	549.9541	548.9468
51	549.9555	548.9482
52	550.3436	549.3363
53	554.5514	553.5441
54	564.3609	563.3536
55	568.3423	567.3350
56	578.3406	577.3333
57	579.5336	578.5264
58	579.5345	578.5272
59	579.5345	578.5273
60	579.5352	578.5280
61	579.5360	578.5287
62	579.5361	578.5288
63	579.5362	578.5289
64	579.5363	578.5290
65	579.5363	578.5290
66	579.5363	578.5290
67	579.5363	578.5290
68	579.5363	578.5290
69	579.5363	578.5291
70	579.5364	578.5291
71	579.5364	578.5292
72	579.5365	578.5292
73	579.5366	578.5293
74	579.5366	578.5293
75	579.5366	578.5294
76	579.5367	578.5294
77	579.5367	578.5294
78	579.5368	578.5295
79	579.5368	578.5295
80	579.5370	578.5297
81	579.5371	578.5298
82	579.5372	578.5299
83	579.5375	578.5302
84	580.5383	579.5310
85	586.4179	585.4106
88	594.3716	593.3643
89	608.3876	607.3803
90	608.3881	607.3809
91	614.5718	613.5646
92	615.4046	614.3973
93	615.4049	614.3977
94	615.4050	614.3977
95	617.4078	616.4005
96	619.5265	618.5192
97	623.9144	622.9072
98	630.4424	629.4352
99	630.4448	629.4375
101	638.3985	637.3912
103	661.5589	660.5516
104	661.5611	660.5538
105	661.5658	660.5586
106	674.4692	673.4619
107	674.4712	673.4639
108	675.6750	674.6677
109	675.6772	674.6700
110	675.6781	674.6708
111	675.6782	674.6709
112	676.4079	675.4007
113	677.6846	676.6773
114	691.3203	690.3130
116	709.6426	708.6353

☑	117	717.6259	716.6186
☑	118	717.6270	716.6197
☑	121	739.6037	738.5965
☑	122	739.6040	738.5968
☑	123	739.6060	738.5987
☑	124	739.6078	738.6006
☑	125	739.6119	738.6046
☑	126	739.6159	738.6086
☑	127	740.4663	739.4590
☑	128	752.5484	751.5411
☑	129	752.5484	751.5412
☑	130	762.5230	761.5158
☑	131	402.3619	802.7093
☑	132	806.5498	805.5425
☑	138	407.7941	813.5737
☑	139	407.7962	813.5778
☑	140	407.7966	813.5787
☑	141	408.7904	815.5663
☑	142	412.7550	823.4954
☑	143	412.7550	823.4955
☑	159	884.9651	883.9578
☑	160	444.1150	886.2154
☑	161	444.1153	886.2160
☑	162	450.3816	898.7487
☑	163	459.2792	916.5438
☑	164	463.1566	924.2987
☑	165	463.1575	924.3004
☑	169	467.1945	932.3744
☑	170	934.8824	933.8751
☑	171	934.8826	933.8753
☑	172	934.8829	933.8757
☑	179	523.7459	1045.4773
☑	180	523.7460	1045.4774
☑	181	523.7463	1045.4780
☑	190	538.7002	1075.3859
☑	197	550.2262	1098.4378
☑	198	550.2577	1098.5009
☑	205	560.6762	1119.3379
☑	206	560.6774	1119.3402
☑	210	563.7871	1125.5597
☑	220	585.2675	1168.5205
☑	227	601.2743	1200.5339
☑	228	401.1853	1200.5340
☑	231	608.2578	1214.5010
☑	233	613.3601	1224.7057
☑	234	613.3643	1224.7141
☑	235	412.1646	1233.4719
☑	245	631.8032	1261.5919
☑	255	442.5360	1324.5861
☑	260	444.1278	1329.3615
☑	264	678.8228	1355.6311
☑	268	693.2401	1384.4656
☑	282	717.8904	1433.7663
☑	283	724.3038	1446.5931
☑	290	503.9099	1508.7078
☑	304	540.8062	1619.3968
☑	305	540.8072	1619.3997
☑	310	549.9502	1646.8287
☑	311	550.9430	1649.8073
☑	323	578.3470	1732.0191
☑	324	875.8856	1749.7566
☑	325	589.5913	1765.7522
☑	326	589.5916	1765.7529
☑	328	590.5958	1768.7655
☑	330	591.3636	1771.0690
☑	334	594.3874	1780.1403
☑	335	446.2609	1781.0146
☑	337	607.2382	1818.6927
☑	338	456.2829	1821.1025
☑	340	609.9459	1826.8158
☑	347	619.5278	1855.5617
☑	351	630.9688	1889.8844
☑	352	949.4841	1896.9535
☑	353	633.3251	1896.9536
☑	355	634.6060	1900.7962
☑	357	640.8522	1919.5347
☑	360	973.1472	1944.2799
☑	362	503.3071	2009.1994
☑	373	696.4442	2086.3107
☑	379	1055.5460	2109.0775
☑	384	720.2844	2157.8313
☑	395	446.2311	2226.1192
☑	396	448.2146	2236.0367
☑	397	755.3478	2263.0215
☑	398	755.3479	2263.0218
☑	402	1142.0989	2282.1833
☑	403	1142.1000	2282.1854
☑	406	458.9498	2289.7128
☑	409	579.5341	2314.1073
☑	410	779.3742	2335.1008
☑	416	812.3715	2434.0926
☑	417	610.7851	2439.1111
☑	418	814.0443	2439.1112
☑	419	407.7936	2440.7181

420	407.7966	2440.7360
421	817.3636	2449.0690
422	817.3637	2449.0693
423	614.5681	2454.2435
424	619.5285	2474.0848
429	500.8049	2498.9883
435	647.4208	2585.6539
436	652.4131	2605.6233
437	444.1174	2658.6605
443	467.6988	2800.1490
444	934.4376	2800.2910
445	402.3838	2809.6357
450	579.5354	2892.6407
453	734.6530	2934.5827
454	595.8318	2974.1225
455	442.2821	3088.9240
457	447.2935	3124.0036
458	1054.5085	3160.5036
460	528.3621	3164.1288
461	534.9780	3203.8244
462	534.9781	3203.8246
465	547.3106	3277.8201
466	1103.9187	3308.7341
469	1103.9203	3308.7390
472	669.8290	3344.1088
475	696.4429	3477.1780
476	500.3093	3495.1144
477	439.5602	3508.4230
478	503.3097	3516.1168
479	710.4275	3547.1013
482	456.2782	3642.1675
483	608.3869	3644.2776
484	613.3425	3674.0112
485	528.3631	3691.4906
486	3700.3652	3699.3579
489	746.4070	3726.9988
490	746.4099	3727.0132
491	622.3351	3727.9671
494	550.9270	3849.4380
495	3881.0805	3880.0732
496	3881.4256	3880.4183
497	777.9014	3884.4705
498	650.2020	3895.1683
499	650.7600	3898.5162
500	650.8030	3898.7741
503	652.4098	3908.4153
504	652.4117	3908.4268
506	563.7815	3939.4198
507	563.7880	3939.4647
508	666.3968	3992.3374
509	4007.2259	4006.2186
511	577.0814	4032.5189
513	533.2675	4258.0817
514	534.9743	4271.7360
515	4342.6010	4341.5937
516	730.9144	4379.4429
517	732.8148	4390.8449
518	740.4593	4436.7120
519	4552.8017	4551.7944
520	571.2537	4561.9711
521	579.2962	4626.3116
522	579.5368	4628.2360
523	4661.8314	4660.8241
525	591.3460	4722.7094
527	679.3544	4748.4296
528	683.3381	4776.3157
530	690.3899	4825.6784
534	697.8415	4877.8399
535	4892.9355	4891.9282
536	4949.7633	4948.7560
538	632.3651	5050.8623
539	849.4459	5090.6317
540	639.3595	5106.8176
541	859.4321	5150.5486
542	739.6045	5170.1806
543	5174.7430	5173.7357
544	740.8533	5178.9222
545	661.5642	5284.4552
546	5317.9240	5316.9167
548	5340.4469	5339.4396
549	5366.4906	5365.4833
550	5386.2880	5385.2807
551	675.8592	5398.8152
554	783.4167	5476.8660
557	942.5246	5649.1040
558	943.0312	5652.1435
559	5690.6035	5689.5962
560	5757.2777	5756.2704
561	5758.7497	5757.7424
562	5835.3970	5834.3897
563	5947.8954	5946.8881
564	883.8837	6180.1352
565	6183.5811	6182.5738
566	6212.3048	6211.2975

☑	567	6212.6319	6211.6246
☑	568	783.4302	6259.3833
☑	569	6343.9315	6342.9242
☑	570	6357.0092	6356.0019
☑	571	6383.4144	6382.4071
☑	572	800.4235	6395.3299
☑	573	6407.5465	6406.5392
☑	574	6422.8918	6421.8845
☑	575	6426.6373	6425.6300
☑	576	6493.7280	6492.7207
☑	577	827.4458	6611.5081
☑	578	6629.1251	6628.1178
☑	579	6711.2427	6710.2354
☑	580	857.4227	6851.3235
☑	581	6941.6801	6940.6728
☑	582	7021.6850	7020.6777
☑	583	884.9829	7071.8048
☑	584	7090.1472	7089.1399
☑	585	887.9637	7095.6516
☑	586	7136.2209	7135.2136
☑	587	7141.0884	7140.0811
☑	588	7141.2152	7140.2079
☑	589	7159.1896	7158.1823
☑	590	7319.0049	7317.9976
☑	591	923.0157	7376.0672
☑	592	7452.3625	7451.3552
☑	593	7473.5419	7472.5346
☑	594	7553.7574	7552.7501
☑	595	7635.0024	7633.9951
☑	596	7892.3466	7891.3393
☑	597	8026.1237	8025.1164
☑	598	8283.4087	8282.4014
☑	599	8431.4359	8430.4286
☑	600	8628.6353	8627.6280
☑	601	8699.1790	8698.1717
☑	602	9980.8943	9979.8870
☑	603	10775.9605	10774.9532

Search Parameters

```
Type of search         : MS/MS Ion Search
Enzyme                 : Trypsin
Fixed modifications    : Carbamidomethyl (C)
Variable modifications : Oxidation (M)
Mass values            : Monoisotopic
Protein Mass           : Unrestricted
Peptide Mass Tolerance : ± 0.05 Da
Fragment Mass Tolerance: ± 0.05 Da
Max Missed Cleavages   : 2
Instrument type        : Default
Number of queries      : 603
```

Mascot Search Results

**User**	: Julian
**Email**	: julian.langer@mpibp-frankfurt.mpg.de
**Search title**	: Submitted from 140318 842 ChKrettler 1-10 swissprot full T 0.05Da by Mascot Daemon on CCSW010
**MS data file**	: D:\Data\1403\140318\140314 842 ChReinhard 7_Tray01-D3_01_17688.d\140314 842 ChReinhard 7_Tray01-D3_01_17688.mgf
**Database**	: SwissProt 57.15 (515203 sequences; 181334896 residues)
**Timestamp**	: 19 Mar 2014 at 09:51:38 GMT
**Protein hits**	: CFAH_HUMAN Complement factor H OS=Homo sapiens GN=CFH PE=1 SV=4
	TRYP_PIG Trypsin OS=Sus scrofa PE=1 SV=1
	K2C1_HUMAN Keratin, type II cytoskeletal 1 OS=Homo sapiens GN=KRT1 PE=1 SV=6
	K22E_HUMAN Keratin, type II cytoskeletal 2 epidermal OS=Homo sapiens GN=KRT2 PE=1 SV=2
	K1C9_HUMAN Keratin, type I cytoskeletal 9 OS=Homo sapiens GN=KRT9 PE=1 SV=3
	ALBU_EQUAS Serum albumin OS=Equus asinus GN=ALB PE=1 SV=1
	IGHG1_HUMAN Ig gamma-1 chain C region OS=Homo sapiens GN=IGHG1 PE=1 SV=1
	A2MG_HUMAN Alpha-2-macroglobulin OS=Homo sapiens GN=A2M PE=1 SV=2
	RPON_HALMA DNA-directed RNA polymerase subunit N OS=Haloarcula marismortui GN=rpoN PE=3 SV=1

Mascot Score Histogram

Ions score is -10*Log(P), where P is the probability that the observed match is a random event.
Individual ions scores > 38 indicate identity or extensive homology (p<0.05).
Protein scores are derived from ions scores as a non-probabilistic basis for ranking protein hits.

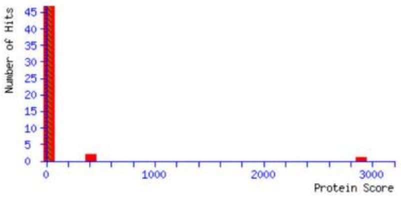

Peptide Summary Report

Format As: Peptide Summary — Help

Significance threshold p< 0.05 — Max. number of hits AUTO

Standard scoring — MudPIT scoring — Ions score or expect cut-off 0 — Show sub-sets 0

Show pop-ups — Suppress pop-ups — Sort unassigned Decreasing Score — Require bold red

Select All — Select None — Search Selected — **Error tolerant** — Archive Report

1. CFAH_HUMAN **Mass:** 143680 **Score:** 2892 **Matches:** 121(92) **Sequences:** 47(40) **emPAI:** 2.75
Complement factor H OS=Homo sapiens GN=CFH PE=1 SV=4
Check to include this hit in error tolerant search or archive report

Query	Observed	Mr(expt)	Mr(calc)	Delta	Miss	Score	Expect	Rank	Unique	Peptide
112	463.2312	924.4479	924.4454	0.0025	0	(37)	0.088	1	U	R.NGFYPATR.G
113	463.2312	924.4479	924.4454	0.0026	0	45	0.015	1	U	R.NGFYPATR.G
114	463.2314	924.4482	924.4454	0.0029	0	(40)	0.045	1	U	R.NGFYPATR.G
115	463.2322	924.4498	924.4454	0.0045	0	(43)	0.023	1	U	R.NGFYPATR.G
122	479.2287	956.4429	956.4392	0.0037	0	11	23	1	U	K.NDFTWFK.L
134	532.3004	1062.5862	1062.5822	0.0040	0	(56)	0.002	1	U	K.IDVHLVPDR.K
135	532.3005	1062.5865	1062.5822	0.0043	0	(53)	0.0036	1	U	K.IDVHLVPDR.K
136	532.3009	1062.5872	1062.5822	0.0050	0	64	0.0003	1	U	K.IDVHLVPDR.K
138	567.3293	1132.6440	1132.6393	0.0047	0	75	2.2e-005	1		R.RPYFPVAVGK.Y
139	567.3293	1132.6441	1132.6393	0.0048	0	(71)	4.7e-005	1		R.RPYFPVAVGK.Y
140	567.3293	1132.6441	1132.6393	0.0048	0	(69)	7.4e-005	1		R.RPYFPVAVGK.Y
141	574.8011	1147.5877	1147.5842	0.0035	0	(47)	0.017	1	U	R.SLGNVIMVCR.K
142	574.8019	1147.5892	1147.5842	0.0050	0	64	0.00031	1	U	R.SLGNVIMVCR.K
144	578.2731	1154.5317	1154.5278	0.0039	0	73	1.6e-005	1	U	R.TGESVEFVCK.R
148	582.7984	1163.5823	1163.5791	0.0032	0	(52)	0.0043	1	U	R.SLGNVIMVCR.K + Oxidation (M)
149	582.7991	1163.5836	1163.5791	0.0045	0	(50)	0.008	1	U	R.SLGNVIMVCR.K + Oxidation (M)
150	593.3017	1184.5889	1184.5860	0.0029	0	(38)	0.077	1	U	K.CLPVTAPENGK.I
151	593.3020	1184.5894	1184.5860	0.0034	0	42	0.032	1	U	K.CLPVTAPENGK.I
152	593.8008	1185.5870	1185.5819	0.0051	1	60	0.0006	1	U	R.TKNDFTWFK.L
153	593.8009	1185.5872	1185.5819	0.0053	1	(57)	0.0011	1	U	R.TKNDFTWFK.L
154	595.2676	1188.5205	1188.5234	-0.0028	0	(40)	0.017	1	U	K.LSYTCEGGFR.I
155	595.2711	1188.5276	1188.5234	0.0043	0	49	0.0027	1	U	K.LSYTCEGGFR.I
156	595.2716	1188.5286	1188.5234	0.0052	0	(47)	0.0045	1	U	K.LSYTCEGGFR.I
157	595.2718	1188.5290	1188.5234	0.0057	0	(43)	0.011	1	U	K.LSYTCEGGFR.I
158	405.1907	1212.5503	1212.5458	0.0045	0	38	0.045	1	U	K.HGGLYHENMR.R
162	621.7763	1241.5381	1241.5347	0.0035	0	(50)	0.0018	1	U	R.TGDEITYQCR.N
163	621.7764	1241.5383	1241.5347	0.0037	0	59	0.0002	1	U	R.TGDEITYQCR.N
164	621.7768	1241.5391	1241.5347	0.0044	0	(52)	0.0011	1	U	R.TGDEITYQCR.N
165	623.3082	1244.6019	1244.5972	0.0047	0	65	0.00016	1	U	K.CTSTGWIPAPR.C
166	623.3084	1244.6022	1244.5972	0.0050	0	(64)	0.00018	1	U	K.CTSTGWIPAPR.C
169	630.3288	1258.6430	1258.6380	0.0050	0	50	0.0071	1	U	R.WQSIPLCVEK.I
170	630.3291	1258.6436	1258.6380	0.0056	0	(42)	0.044	1	U	R.WQSIPLCVEK.I
171	631.7977	1261.5808	1261.5761	0.0047	0	(41)	0.027	1	U	K.DGWSAQPTCIK.S
172	631.7977	1261.5809	1261.5761	0.0048	0	46	0.0081	1	U	K.DGWSAQPTCIK.S
173	428.2491	1281.7254	1281.7193	0.0061	1	(51)	0.0027	1	U	R.KGEWVALNPLR.K
174	641.8700	1281.7254	1281.7193	0.0061	1	80	3.3e-006	1	U	R.KGEWVALNPLR.K
175	428.2492	1281.7258	1281.7193	0.0064	1	(50)	0.0038	1	U	R.KGEWVALNPLR.K
179	655.3067	1308.5988	1308.5955	0.0034	0	53	0.0014	1	U	K.SCDIPVFMNAR.T
185	663.3003	1324.5861	1324.5904	-0.0042	0	(22)	1.4	1	U	K.SCDIPVFMNAR.T + Oxidation (M)
186	663.3050	1324.5955	1324.5904	0.0051	0	(39)	0.033	1	U	K.SCDIPVFMNAR.T + Oxidation (M)
189	671.3554	1340.6963	1340.6936	0.0027	0	(76)	1.5e-005	1	U	K.SPDVINGSPISQK.I
190	671.3558	1340.6971	1340.6936	0.0035	0	(72)	3.5e-005	1	U	K.SPDVINGSPISQK.I
191	671.3559	1340.6973	1340.6936	0.0037	0	(75)	1.7e-005	1	U	K.SPDVINGSPISQK.I
192	671.3562	1340.6979	1340.6936	0.0043	0	(50)	0.0059	1	U	K.SPDVINGSPISQK.I
193	671.3565	1340.6984	1340.6936	0.0049	0	(55)	0.0018	1	U	K.SPDVINGSPISQK.I
194	671.3567	1340.6988	1340.6936	0.0052	0	(60)	0.00051	1	U	K.SPDVINGSPISQK.I
195	671.3569	1340.6992	1340.6936	0.0056	0	(58)	0.00087	1	U	K.SPDVINGSPISQK.I
196	671.3569	1340.6993	1340.6936	0.0057	0	81	4e-006	1	U	K.SPDVINGSPISQK.I
197	671.3572	1340.6999	1340.6936	0.0063	0	(73)	3e-005	1	U	K.SPDVINGSPISQK.I
198	671.3573	1340.6999	1340.6936	0.0064	0	(57)	0.00096	1	U	K.SPDVINGSPISQK.I
199	671.3574	1340.7003	1340.6936	0.0067	0	(57)	0.001	1	U	K.SPDVINGSPISQK.I
200	671.3577	1340.7009	1340.6936	0.0073	0	(73)	2.9e-005	1	U	K.SPDVINGSPISQK.I
205	683.3477	1364.6809	1364.6758	0.0051	0	76	1.4e-005	1	U	R.EIMENYNIALR.W
206	683.3482	1364.6818	1364.6758	0.0060	0	(61)	0.00051	1	U	R.EIMENYNIALR.W
207	691.3453	1380.6760	1380.6707	0.0053	0	(38)	0.082	1	U	R.EIMENYNIALR.W + Oxidation (M)
209	698.3984	1394.7822	1394.7769	0.0053	0	60	0.00045	1	U	K.SSNLIILEEHLK.N
210	465.9347	1394.7822	1394.7769	0.0053	0	(51)	0.0032	1	U	K.SSNLIILEEHLK.N
211	698.3991	1394.7836	1394.7769	0.0068	0	(60)	0.00045	1	U	K.SSNLIILEEHLK.N
212	465.9352	1394.7837	1394.7769	0.0068	0	(22)	2.4	1	U	K.SSNLIILEEHLK.N
213	698.3992	1394.7838	1394.7769	0.0069	0	(55)	0.0013	1	U	K.SSNLIILEEHLK.N
215	470.9475	1409.8206	1409.8143	0.0063	2	(35)	0.076	1	U	R.KGEWVALNPLRK.C
216	470.9475	1409.8208	1409.8143	0.0065	2	36	0.061	1	U	R.KGEWVALNPLRK.C
220	721.3568	1440.6990	1440.6932	0.0058	0	(13)	19	1	U	K.EGWIHTVCINGR.W
221	481.2403	1440.6990	1440.6932	0.0058	0	(31)	0.36	1	U	K.EGWIHTVCINGR.W
222	481.2406	1440.7000	1440.6932	0.0068	0	42	0.023	1	U	K.EGWIHTVCINGR.W
223	721.3573	1440.7000	1440.6932	0.0068	0	(36)	0.098	1	U	K.EGWIHTVCINGR.W
228	741.3621	1480.7096	1480.7021	0.0075	0	93	2.5e-007	1	U	K.CFEGFGIDGPAIAK.C

229	741.3624	1480.7103	1480.7021	0.0083	0	(69)	6e-005	1	U	K.CFEGFGIDGPAIAK.C
230	755.3611	1508.7077	1508.7003	0.0074	0	(17)	7.1	1	U	R.CTLKPCDYPDIK.H
231	503.9099	1508.7078	1508.7003	0.0074	0	50	0.0042	1	U	R.CTLKPCDYPDIK.H
232	503.9099	1508.7078	1508.7003	0.0074	0	(34)	0.15	1	U	R.CTLKPCDYPDIK.H
234	509.9270	1526.7592	1526.7551	0.0041	0	(33)	0.23	1	U	K.SIDVACHPGYALPK.A
235	764.3869	1526.7592	1526.7551	0.0041	0	76	1.2e-005	1	U	K.SIDVACHPGYALPK.A
236	509.9276	1526.7610	1526.7551	0.0059	0	(17)	8.6	1	U	K.SIDVACHPGYALPK.A
240	773.3482	1544.6818	1544.6752	0.0066	0	61	0.00013	1	U	K.WSSPPQCEGLPCK.S
241	542.9456	1625.8150	1625.8096	0.0054	1	(27)	0.99	1	U	R.GKEGWIHTVCINGR.W
242	542.9461	1625.8165	1625.8096	0.0069	1	32	0.34	1	U	R.GKEGWIHTVCINGR.W
246	827.8781	1653.7416	1653.7345	0.0071	0	63	8.3e-005	1	U	K.AGEQVTYTCATYYK.M
248	561.9782	1682.9129	1682.9025	0.0104	1	25	1.4	1	U	K.CKSSNLIILEEHLK.N
256	908.4283	1814.8420	1814.8357	0.0064	0	100	2.5e-008	1	U	K.LGYVTADGETSGSITCGK.D
257	908.4285	1814.8425	1814.8357	0.0069	0	(87)	4.6e-007	1	U	K.LGYVTADGETSGSITCGK.D
258	908.4291	1814.8437	1814.8357	0.0080	0	(83)	1.2e-006	1	U	K.LGYVTADGETSGSITCGK.D
263	934.4219	1866.8293	1866.8206	0.0087	0	83	7.4e-007	1	U	K.SCDNPYIPNGDYSPLR.I
271	976.4378	1950.8609	1950.8564	0.0045	0	40	0.012	1	U	K.AQTTVTCMENGWSPTPR.C + Oxidation (M)
272	983.4924	1964.9702	1964.9626	0.0077	0	103	1.9e-008	1	U	K.EQVQSCGPPPELLNGNVK.E
274	1004.9756	2007.9367	2007.9281	0.0086	0	(60)	0.00025	1	U	K.TDCLSLPSFENAIPMGEK.K
276	1010.9965	2019.9784	2019.9684	0.0100	0	90	3.9e-007	1	U	R.DTSCVNPPTVQNAYIVSR.Q
277	1010.9965	2019.9785	2019.9684	0.0101	0	(82)	2.2e-006	1	U	R.DTSCVNPPTVQNAYIVSR.Q
278	1012.9728	2023.9311	2023.9231	0.0080	0	(55)	0.00077	1	U	K.TDCLSLPSFENAIPMGEK.K + Oxidation (M)
279	1012.9734	2023.9323	2023.9231	0.0092	0	62	0.00014	1	U	K.TDCLSLPSFENAIPMGEK.K + Oxidation (M)
280	1012.9748	2023.9350	2023.9231	0.0119	0	(62)	0.00015	1	U	K.TDCLSLPSFENAIPMGEK.K + Oxidation (M)
282	681.2717	2040.7933	2040.7830	0.0103	0	(11)	0.52	1	U	K.IEGDEEMHCSDDGFWSK.E
283	681.2721	2040.7945	2040.7830	0.0115	0	17	0.13	1	U	K.IEGDEEMHCSDDGFWSK.E
297	713.0187	2136.0344	2136.0231	0.0113	1	37	0.073	1	U	K.TDCLSLPSFENAIPMGEKK.D
299	1075.9776	2149.9406	2149.9296	0.0110	0	112	4.6e-010	1	U	R.ECDTDGWTNDIPICEVVK.C
300	1075.9925	2149.9705	2149.9296	0.0408	0	(57)	0.00029	1	U	R.ECDTDGWTNDIPICEVVK.C
301	718.3496	2152.0270	2152.0180	0.0090	1	(28)	0.49	1	U	K.TDCLSLPSFENAIPMGEKK.D + Oxidation (M)
305	1082.0176	2162.0206	2162.0102	0.0104	0	107	5.4e-009	1	U	K.AVYTCNEGYQLLGEINYR.E
306	726.6612	2176.9616	2176.9518	0.0099	0	(21)	0.82	1	U	R.GDAVCTESGWRPLPSCEEK.S
307	726.6613	2176.9622	2176.9518	0.0104	0	49	0.0013	1	U	R.GDAVCTESGWRPLPSCEEK.S
308	726.6621	2176.9646	2176.9518	0.0128	0	(41)	0.0088	1	U	R.GDAVCTESGWRPLPSCEEK.S
312	736.6919	2207.0538	2207.0429	0.0108	1	(20)	2.7	1	U	K.IVSSAMEPDREYHFGQAVR.F + Oxidation (M)
313	552.7707	2207.0538	2207.0429	0.0108	1	(48)	0.0051	1	U	K.IVSSAMEPDREYHFGQAVR.F + Oxidation (M)
314	736.6922	2207.0547	2207.0429	0.0117	1	(30)	0.28	1	U	K.IVSSAMEPDREYHFGQAVR.F + Oxidation (M)
315	552.7709	2207.0547	2207.0429	0.0118	1	(56)	0.00073	1	U	K.IVSSAMEPDREYHFGQAVR.F + Oxidation (M)
316	552.7712	2207.0557	2207.0429	0.0127	1	62	0.0002	1	U	K.IVSSAMEPDREYHFGQAVR.F + Oxidation (M)
324	754.0073	2259.0001	2258.9902	0.0099	1	42	0.006	1	U	K.TKEEYGHSEVVEYYCNPR.F
325	754.0080	2259.0021	2258.9902	0.0119	1	(41)	0.0085	1	U	K.TKEEYGHSEVVEYYCNPR.F
328	1133.0542	2264.0938	2264.0848	0.0090	0	119	3.9e-010	1	U	K.SSIDIENGFISESQYTYALK.E
344	1206.5794	2411.1441	2411.1348	0.0093	0	90	2.3e-007	1	U	K.VSVLCQENYLIQEGEEITCK.D
347	1301.6260	2601.2374	2601.2235	0.0139	0	(21)	1.9	1	U	R.NTEILTGSWSDQTYPEGTQAIYK.C
348	1301.6279	2601.2413	2601.2235	0.0178	0	76	5.8e-006	1	U	R.NTEILTGSWSDQTYPEGTQAIYK.C
349	868.0877	2601.2413	2601.2235	0.0178	0	(57)	0.00047	1	U	R.NTEILTGSWSDQTYPEGTQAIYK.C
354	914.1063	2739.2972	2739.2844	0.0128	1	69	2.4e-005	1	U	K.VSVLCQENYLIQEGEEITCKDGR.W
355	914.1111	2739.3114	2739.2844	0.0270	1	(45)	0.0067	1	U	K.VSVLCQENYLIQEGEEITCKDGR.W
356	914.1116	2739.3129	2739.2844	0.0285	1	(40)	0.021	1	U	K.VSVLCQENYLIQEGEEITCKDGR.W
358	920.1201	2757.3385	2757.3246	0.0140	1	21	2.1	1	U	R.RNTEILTGSWSDQTYPEGTQAIYK.C
364	728.3408	2909.3343	2909.3178	0.0165	0	(37)	0.026	1	U	K.SPPEISHGVVAHMSDSYQYGEEVTYK.C
365	970.7854	2909.3343	2909.3178	0.0165	0	(11)	10	1	U	K.SPPEISHGVVAHMSDSYQYGEEVTYK.C
366	970.7861	2909.3366	2909.3178	0.0188	0	(8)	20	1	U	K.SPPEISHGVVAHMSDSYQYGEEVTYK.C
367	728.3414	2909.3366	2909.3178	0.0188	0	47	0.0025	1	U	K.SPPEISHGVVAHMSDSYQYGEEVTYK.C

2. TRYP_PIG **Mass:** 25078 **Score:** 372 **Matches:** 26(22) **Sequences:** 5(5) **emPAI:** 1.88

Trypsin OS=Sus scrofa PE=1 SV=1

Check to include this hit in error tolerant search or archive report

Query	Observed	Mr(expt)	Mr(calc)	Delta	Miss	Score	Expect	Rank	Unique	Peptide
98	421.7587	841.5029	841.5022	0.0007	0	(54)	0.0029	1	U	R.VATVSLPR.S
99	421.7602	841.5059	841.5022	0.0038	0	(51)	0.0058	1	U	R.VATVSLPR.S
100	421.7604	841.5063	841.5022	0.0042	0	(48)	0.012	1	U	R.VATVSLPR.S
101	421.7605	841.5065	841.5022	0.0043	0	(49)	0.0096	1	U	R.VATVSLPR.S
102	421.7613	841.5079	841.5022	0.0058	0	54	0.0027	1	U	R.VATVSLPR.S
127	523.2865	1044.5585	1044.5564	0.0021	0	(49)	0.013	1	U	K.LSSPATLNSR.V
128	523.2869	1044.5592	1044.5564	0.0029	0	(58)	0.0016	1	U	K.LSSPATLNSR.V
129	523.2871	1044.5596	1044.5564	0.0033	0	60	0.00088	1	U	K.LSSPATLNSR.V
130	523.2873	1044.5601	1044.5564	0.0038	0	(52)	0.0055	1	U	K.LSSPATLNSR.V
131	523.2875	1044.5605	1044.5564	0.0041	0	(55)	0.0033	1	U	K.LSSPATLNSR.V
132	523.2878	1044.5611	1044.5564	0.0048	0	(47)	0.02	1	U	K.LSSPATLNSR.V
133	523.2882	1044.5619	1044.5564	0.0056	0	(58)	0.0015	1	U	K.LSSPATLNSR.V
317	737.7092	2210.1059	2210.0967	0.0092	0	(100)	4.2e-008	1	U	R.LGEHNIDVLEGNEQFINAAK.I
318	1106.0610	2210.1074	2210.0967	0.0107	0	(8)	59	1	U	R.LGEHNIDVLEGNEQFINAAK.I
319	737.7097	2210.1074	2210.0967	0.0107	0	110	3.8e-009	1	U	R.LGEHNIDVLEGNEQFINAAK.I
320	1106.0614	2210.1083	2210.0967	0.0116	0	(31)	0.33	1	U	R.LGEHNIDVLEGNEQFINAAK.I
321	737.7100	2210.1083	2210.0967	0.0116	0	(103)	2e-008	1	U	R.LGEHNIDVLEGNEQFINAAK.I
322	737.7147	2210.1221	2210.0967	0.0254	0	(7)	89	3	U	R.LGEHNIDVLEGNEQFINAAK.I
330	761.7319	2282.1738	2282.1729	0.0009	0	(47)	0.0074	1	U	K.IITHPNFNGNTLDNDIMLIK.L
331	761.7354	2282.1843	2282.1729	0.0114	0	(56)	0.00087	1	U	K.IITHPNFNGNTLDNDIMLIK.L
334	761.7355	2282.1848	2282.1729	0.0119	0	84	1.3e-006	1	U	K.IITHPNFNGNTLDNDIMLIK.L
338	767.0671	2298.1796	2298.1678	0.0118	0	(45)	0.012	1	U	K.IITHPNFNGNTLDNDIMLIK.L + Oxidation (M)
339	767.0677	2298.1814	2298.1678	0.0136	0	(58)	0.00056	1	U	K.IITHPNFNGNTLDNDIMLIK.L + Oxidation (M)
380	828.1917	3308.7375	3308.7187	0.0189	1	63	9.2e-005	1	U	K.IITHPNFNGNTLDNDIMLIKLSSPATLNSR.V
381	828.1928	3308.7420	3308.7187	0.0233	1	(56)	0.00044	1	U	K.IITHPNFNGNTLDNDIMLIKLSSPATLNSR.V
383	832.1905	3324.7328	3324.7136	0.0192	1	(15)	5.1	1	U	K.IITHPNFNGNTLDNDIMLIKLSSPATLNSR.V + Oxidation (M)

3. K2C1_HUMAN **Mass:** 66170 **Score:** 358 **Matches:** 10(7) **Sequences:** 6(5) **emPAI:** 0.36

Keratin, type II cytoskeletal 1 OS=Homo sapiens GN=KRT1 PE=1 SV=6

Check to include this hit in error tolerant search or archive report

Query	Observed	Mr(expt)	Mr(calc)	Delta	Miss	Score	Expect	Rank	Unique	Peptide
108	437.7547	873.4949	873.4920	0.0029	0	(29)	1.3	1	U	R.SLVNLGGSK.S
109	437.7552	873.4958	873.4920	0.0038	0	(29)	1.3	1	U	R.SLVNLGGSK.S
110	437.7555	873.4964	873.4920	0.0044	0	31	0.87	1	U	R.SLVNLGGSK.S
123	487.2715	972.5283	972.5240	0.0044	0	44	0.043	1		K.IEISELNR.V
176	650.7722	1299.5299	1299.5224	0.0076	0	44	0.0027	1	U	K.NMQDMVEDYR.N
177	651.8638	1301.7131	1301.7078	0.0052	0	(48)	0.012	1	U	R.SLDLDSIIAEVK.A
178	651.8640	1301.7133	1301.7078	0.0055	0	97	1.4e-007	1	U	R.SLDLDSIIAEVK.A
208	692.3520	1382.6894	1382.6830	0.0064	0	57	0.00088	1	U	K.SLNNQFASFIDK.V
251	858.9334	1715.8522	1715.8438	0.0084	0	(76)	1.4e-005	1	U	K.QISNLQQSISDAEQR.G
252	858.9335	1715.8525	1715.8438	0.0087	0	86	1.3e-006	1	U	K.QISNLQQSISDAEQR.G

4. K22E_HUMAN **Mass:** 65678 **Score:** 91 **Matches:** 2(2) **Sequences:** 2(2) **emPAI:** 0.11

Keratin, type II cytoskeletal 2 epidermal OS=Homo sapiens GN=KRT2 PE=1 SV=2

Check to include this hit in error tolerant search or archive report

Query	Observed	Mr(expt)	Mr(calc)	Delta	Miss	Score	Expect	Rank	Unique	Peptide
123	487.2715	972.5283	972.5240	0.0044	0	44	0.043	1		K.IEISELNR.V
187	665.3697	1328.7248	1328.7187	0.0060	0	47	0.012	1	U	R.NLDLDSIIAEVK.A

5. K1C9_HUMAN **Mass:** 62255 **Score:** 89 **Matches:** 4(1) **Sequences:** 4(1) **emPAI:** 0.11

Keratin, type I cytoskeletal 9 OS=Homo sapiens GN=KRT9 PE=1 SV=3

Check to include this hit in error tolerant search or archive report

Query	Observed	Mr(expt)	Mr(calc)	Delta	Miss	Score	Expect	Rank	Unique	Peptide
145	579.3016	1156.5886	1156.5836	0.0049	0	55	0.0018	1	U	R.QGVDADINGLR.Q
260	613.3295	1836.9667	1836.9581	0.0086	0	31	0.33	1	U	R.HGVQELEIELQSQLSK.K
262	617.9842	1850.9309	1850.9196	0.0113	1	1	3.5e+002	3	U	K.TLNDMRQEYEQLIAK.N

Query	Observed	Mr(expt)	Mr(calc)	Delta	Miss	Score	Expect	Rank	Unique	Peptide
379	1088.8494	3263.5263	3263.5066	0.0198	0	1	96	1	U	K.DIENQYETQITQIEHEVSSSGQEVQSSAK.E

6. ALBU_EQUAS **Mass:** 70490 **Score:** 64 **Matches:** 1(1) **Sequences:** 1(1) **emPAI:** 0.05
Serum albumin OS=Equus asinus GN=ALB PE=1 SV=1
Check to include this hit in error tolerant search or archive report

Query	Observed	Mr(expt)	Mr(calc)	Delta	Miss	Score	Expect	Rank	Unique	Peptide
143	575.3137	1148.6128	1148.6077	0.0051	0	64	0.00029	1	U	K.LVNEVTEFAK.K

Proteins matching the same set of peptides:

ALBU_FELCA **Mass:** 70611 **Score:** 64 **Matches:** 1(1) **Sequences:** 1(1)
Serum albumin OS=Felis catus GN=ALB PE=1 SV=1
ALBU_HORSE **Mass:** 70550 **Score:** 64 **Matches:** 1(1) **Sequences:** 1(1)
Serum albumin OS=Equus caballus GN=ALB PE=1 SV=1
ALBU_MACFA **Mass:** 70825 **Score:** 64 **Matches:** 1(1) **Sequences:** 1(1)
Serum albumin OS=Macaca fascicularis GN=ALB PE=2 SV=1
ALBU_MACMU **Mass:** 69832 **Score:** 64 **Matches:** 1(1) **Sequences:** 1(1)
Serum albumin (Fragment) OS=Macaca mulatta GN=ALB PE=2 SV=1
ALBU_HUMAN **Mass:** 71317 **Score:** 64 **Matches:** 1(1) **Sequences:** 1(1)
Serum albumin OS=Homo sapiens GN=ALB PE=1 SV=2
ALBU_PONAB **Mass:** 71317 **Score:** 64 **Matches:** 1(1) **Sequences:** 1(1)
Serum albumin OS=Pongo abelii GN=ALB PE=2 SV=1

7. IGHG1_HUMAN **Mass:** 36596 **Score:** 46 **Matches:** 2(0) **Sequences:** 2(0)
Ig gamma-1 chain C region OS=Homo sapiens GN=IGHG1 PE=1 SV=1
Check to include this hit in error tolerant search or archive report

Query	Observed	Mr(expt)	Mr(calc)	Delta	Miss	Score	Expect	Rank	Unique	Peptide
146	581.3185	1160.6225	1160.6223	0.0002	0	39	0.11	1	U	K.NQVSLTCLVK.G
266	937.4697	1872.9249	1872.9146	0.0104	0	7	87	1	U	K.TTPPVLDSDGSFFLYSK.L

8. A2MG_HUMAN **Mass:** 164614 **Score:** 44 **Matches:** 2(1) **Sequences:** 2(1) **emPAI:** 0.02
Alpha-2-macroglobulin OS=Homo sapiens GN=A2M PE=1 SV=2
Check to include this hit in error tolerant search or archive report

Query	Observed	Mr(expt)	Mr(calc)	Delta	Miss	Score	Expect	Rank	Unique	Peptide
168	630.2925	1258.5705	1258.5652	0.0053	0	3	1.2e+002	7	U	R.VGFYESDVMGR.G
261	923.0259	1844.0373	1844.0295	0.0078	0	41	0.024	1	U	R.LLIYAVLPTGDVIGDSAK.Y

Proteins matching the same set of peptides:

A2MG_PONAB **Mass:** 164585 **Score:** 44 **Matches:** 2(1) **Sequences:** 2(1)
Alpha-2-macroglobulin OS=Pongo abelii GN=A2M PE=2 SV=1

9. RPON_HALMA **Mass:** 7954 **Score:** 42 **Matches:** 1(0) **Sequences:** 1(0)
DNA-directed RNA polymerase subunit N OS=Haloarcula marismortui GN=rpoN PE=3 SV=1
Check to include this hit in error tolerant search or archive report

Query	Observed	Mr(expt)	Mr(calc)	Delta	Miss	Score	Expect	Rank	Unique	Peptide
125	515.3025	1028.5905	1028.5502	0.0403	0	42	0.057	1	U	K.VLDELGVER.H

Peptide matches not assigned to protein hits: (no details means no match)

Query	Observed	Mr(expt)	Mr(calc)	Delta	Miss	Score	Expect	Rank	Unique	Peptide
126	516.3055	1030.5965	1030.5910	0.0055	0	30	0.62	1		VLDELTLTK
214	698.8870	1395.7594	1395.7544	0.0050	1	27	0.98	1		GHEKCALLILDK
117	463.7240	925.4335	925.4440	-0.0105	1	22	2.5	1		DGYMIRR + Oxidation (M)
295	695.0035	2081.9888	2081.9840	0.0048	0	18	4.3	1		VACEEPPFIENGAANLHSK
120	471.7843	941.5540	941.5844	-0.0304	1	17	14	1		MALKPKVR
255	887.9572	1773.8999	1773.8567	0.0432	0	16	15	1		LNDGASSVILMSESHAK + Oxidation (M)
147	581.8089	1161.6032	1161.5666	0.0366	0	16	23	1		DGIAQLTYAGPG
116	463.7195	925.4245	925.4440	-0.0194	1	15	12	1		DGYMIRR + Oxidation (M)
322	737.7147	2210.1221	2210.1080	0.0141	2	13	22	1		DAGRYSRQLLVDDFGVSGQK
227	738.8834	1475.7522	1475.7481	0.0042	1	12	41	1		DAYREAQQLVQR
183	662.3096	1322.6046	1322.6388	-0.0341	1	12	22	1		MGTAEKVTVEDK + Oxidation (M)
286	1029.9511	2057.8876	2057.9265	-0.0389	1	11	5.9	1		EGHFVYSDRSPMQTFNK + Oxidation (M)
168	630.2925	1258.5705	1258.5976	-0.0271	1	10	24	1		SGSMQTTFTRK + Oxidation (M)
264	623.3086	1866.9041	1866.8564	0.0477	1	10	38	1		NRMLESLTSECENIR + Oxidation (M)
378	463.2333	3235.5822	3235.6152	-0.0329	2	10	22	1		LLGISADHEAMTGKDVMNRLQLGYMVGLR + 3 Oxidation (M)
392	593.3036	3553.7782	3553.7731	0.0051	1	9	24	1		ALIYMASLPQGQMTSISQVTEVYGVSRNHMVK + Oxidation (M)
389	589.8026	3532.7722	3532.7655	0.0067	2	9	26	1		LGDFMMKVAQNTLKEVDLILFMINAEEGYGK + Oxidation (M)
96	802.4334	801.4261	801.4596	-0.0335	0	9	1.9e+002	1		IEAAASLK
262	617.9842	1850.9309	1850.9473	-0.0164	1	8	72	1		TLEVSETSQSLKTDVSK
427	4628.2701	4627.2628	4627.2204	0.0424	2	7	19	1		VAESVVSVDSDVECSMSFAIDNQRLAVLNGDAKVTVTLECQR + Oxidation (M)
426	661.3451	4622.3648	4622.3238	0.0409	2	7	15	1		NVWGFLAATSVTFVGVMGMRSYYYGKFMPVGLIAGASLLMAAK + 3 Oxidation (M)
450	5469.7816	5468.7743	5468.8211	-0.0468	0	6	11	1		GILSEVLTTMSQAQASIVTINQNIPIHNWASVVMSFDISALQGTLDDLVTK
103	846.4451	845.4378	845.4130	0.0248	1	6	2.7e+002	1		EPSEEKK
167	630.2786	1258.5427	1258.5441	-0.0014	0	6	36	1		FDHYCSFVGK
253	430.2439	1716.9467	1716.9410	0.0057	1	6	1.1e+002	1		EGGKLQIEAYIIVER
387	432.2391	3449.8548	3449.8275	0.0272	2	6	28	1		MHQSTLLKPVSCYGIGVHTGKRTQLTIEPAK
393	593.8002	3556.7578	3556.7271	0.0307	1	6	55	1		AIMFWIMARPAEYYELFNLLKDNNNEHSK
104	846.4452	845.4379	845.4131	0.0248	0	5	3.5e+002	1		DGELPTSK
390	589.8056	3532.7898	3532.7781	0.0116	2	5	60	1		AMQVALNLGMASRYVLHTPLMWIDKAETWR + 2 Oxidation (M)
335	762.0635	2283.1687	2283.1569	0.0118	2	5	1.2e+002	1		VQTIEEVMTYIDKWTKQR + Oxidation (M)
470	6330.3488	6329.3415	6329.3227	0.0188	2	5	3.6	1		MIKPFSVSELVTEIAPLLRMALPPNIELSFRFDQMQSVIEGSPLELQQVLINICK + 3 Oxidation (M)
302	718.6843	2153.0310	2153.0245	0.0065	0	5	1e+002	1		MQTLSIQHGTLVTMDQYR + 2 Oxidation (M)
137	558.7588	1115.5030	1115.5321	-0.0291	0	5	80	1		FYGLGAVDMK + Oxidation (M)
105	846.4456	845.4383	845.4243	0.0140	1	5	4e+002	1		AEDVERK
460	5768.1430	5767.1357	5767.1278	0.0079	2	4	3.2	1		QLALKAGGFFGVAQMVMPLLGFYLGVQLHDYIGGIHHWVALGVLGFLGLKMIR + Oxidation (M)
414	630.3316	4405.2701	4405.2734	-0.0033	1	4	31	1		TCDIDIEGSNAVVIGRSNIVGKPVAQLLLNENATVTICHSK
303	719.6868	2156.0387	2156.0597	-0.0210	0	4	1.2e+002	1		APVSPALASPSSEVSDVSEATR
64	666.3933	665.3860	665.3748	0.0112	0	4	2.7e+002	1		DIPPPK
239	771.3972	1540.7798	1540.8097	-0.0298	0	4	2.2e+002	1		VLSLAQEQVGGSPEK
265	934.9185	1867.8224	1867.8557	-0.0332	1	4	48	1		EGDFNRDVNLMIGMNK + Oxidation (M)
421	651.8834	4556.1330	4556.1019	0.0311	2	4	24	1		IREEMAELILRYEEVEDVSGEEALDEMELDMLDEAEGIK + Oxidation (M)
436	638.3205	5098.5059	5098.5539	-0.0481	2	4	29	1		GSGQVVTYALNTITNLKVQLIRMAEAEMVIHHHHVQDCDDSTLVR
250	851.3694	1700.7243	1700.7465	-0.0222	0	3	31	1		CFDVGGGLAVDYDGTR
74	676.4129	675.4056	675.3625	0.0431	0	3	3.7e+002	1		APLTMK + Oxidation (M)
428	665.3544	4650.4297	4650.3837	0.0460	2	3	27	1		KVTDPFVDPGLGKNILFMIGVLCGGIIFGTVAGFVSMVPYMMK + 2 Oxidation (M)
486	1207.0735	7236.3970	7236.4072	-0.0102	1	3	4.8	1		SWEGAPAPEVNLSHVSEEFQACVPPQTGLPSPRQTFDDVKPENSPAPHDSPADSSFDRPTEVVLER
337	764.3866	2290.1379	2290.1113	0.0267	0	3	1.9e+002	1		ITGLMAEFYEIVLMVDADTK + 2 Oxidation (M)
329	1133.5503	2265.0860	2265.0738	0.0122	2	3	1.5e+002	1		KAGMKVCCGGIVGMGEEVLDR
398	631.7979	3784.7439	3784.6947	0.0493	2	3	35	1		EKLCSVQSENTMMKSEHSQTMCQLTSQNEALR
298	714.3276	2139.9609	2139.9765	-0.0156	2	3	73	1		EGFQHPSRMGCGAYMRIK + Oxidation (M)
485	7101.9419	7100.9346	7100.8977	0.0369	2	2	2	1		QLMMPLNKAGHKWALILTSLMTFLLLINLLGLLPYTFTPTTQLSMNMALAFPLWLATLLTGLR + 2 Oxidation (M)
386	1133.6142	3397.8207	3397.7924	0.0283	1	2	64	1		ALMGSNMQRQAVPLLFPKPPIVGTGMESVVAK + 2 Oxidation (M)
461	983.9937	5897.9187	5897.9648	-0.0461	2	2	22	1		DLTHLPMPTGKIFVSVYNIQDETGQFKPYPASNFSTAVPQSATAMLVTALKDSR + Oxidation (M)
269	968.9427	1935.8708	1935.8754	-0.0046	1	2	1.1e+002	1		KAPSVMNGGAQMWMQPR + 3 Oxidation (M)
361	934.4161	2800.2265	2800.2264	0.0001	0	2	38	1		TGSLWPVTFGLACCAVEMMHAGMAR + 3 Oxidation (M)
446	764.3732	5343.5618	5343.5130	0.0489	0	2	33	1		NHLIGGFLAGSEGTVDVVDGVNAEGDQVPGFDEVHMVYTEFHSMLSQVPK + Oxidation (M)
422	763.8745	4577.2036	4577.2425	-0.0389	0	1	58	1		LFSEDQGLIISVMTNPTTGGVSASFASLGDYNFAEPGALIGFAGR
463	1017.9988	6101.9493	6101.9447	0.0046	1	1	20	1		TVNSNGNVIFNIANLSINQSNGQITVNSNGTMMNGGSSNNTKSIAGFETLGTFIAPDTR
448	683.3524	5458.7612	5458.7226	0.0385	1	1	33	1		MSLPPHLCVPIDILMIDNFDSFTWNLYQSLCLLGADVTVIRNDAIPR + Oxidation (M)
161	620.7591	1239.5036	1239.5150	-0.0114	0	1	55	1		NESTGSMGTQGR + Oxidation (M)
352	885.7259	2654.1560	2654.1952	-0.0392	1	1	40	1		NTGGKGGDYALAPVSQSSDMSLPDCK

160	614.7927	1227.5708	1227.6095	-0.0387	0	1	2.7e+002	1	SGPVVIDADEAR
469	6330.0739	6329.0666	6329.0727	-0.0061	2	1	22	1	GMPLYAHIAELNGTAGVYSMPLPMMNIINGGEHADNNVDIQEFMIQPVGAKTLKEAVR + 4 Oxidation (M)
360	699.0611	2792.2154	2792.2244	-0.0090	2	1	36	1	TEGCMFEKGPRQFYDDTCVVPEK
357	918.7784	2753.3133	2753.3384	-0.0251	1	1	2e+002	1	ALLTCDVWEHAYYIDYRNARPK
368	728.6078	2910.4021	2910.4002	0.0019	1	1	2.1e+002	1	ATRNSPMALGTAQGDPGEAGTRPGSDAGLR
377	802.4330	3205.7030	3205.6659	0.0371	0	0	1.5e+002	1	VDGSVLPPFIAGGAIYGHSALGEDVNIPELAK
375	1054.5092	3160.5057	3160.5023	0.0034	2	0	1.6e+002	1	LEFTELTEVMKDVDFKVFSGPANSDNGR + Oxidation (M)
415	638.3202	4461.1907	4461.1911	-0.0004	1	0	94	1	MSQYVIAIDQGTTSSRAIIFDHDGNIVSTGQLEHEQIFPK + Oxidation (M)
409	1409.2648	4224.7724	4224.8160	-0.0435	1	0	7.5	1	QMDAGDYYVQESIDILDSDDSGSLFEKMGQLAYSMCK + Oxidation (M)
1	402.3579	401.3507							
2	402.3585	401.3512							
3	415.2116	414.2043							
4	415.2125	414.2052							
5	415.2143	414.2070							
6	415.3426	414.3353							
7	415.3440	414.3367							
8	430.2454	429.2381							
9	432.2389	431.2316							
10	432.2396	431.2324							
11	433.1820	432.1748							
12	444.1166	443.1093							
13	444.1172	443.1099							
14	444.1188	443.1115							
15	444.3478	443.3405							
16	447.2940	446.2867							
17	453.3454	452.3381							
18	459.2814	458.2741							
19	476.3080	475.3008							
20	490.2883	489.2810							
21	500.3069	499.2996							
22	500.3078	499.3005							
23	514.2343	513.2270							
24	520.3344	519.3271							
25	520.3350	519.3277							
26	520.3370	519.3298							
27	525.2902	524.2829							
28	528.3630	527.3558							
29	528.3654	527.3581							
30	531.2759	530.2686							
31	534.3143	533.3071							
32	534.9821	533.9748							
33	539.3339	538.3266							
34	539.3364	538.3292							
35	539.3371	538.3298							
36	550.3491	549.3418							
37	554.3103	553.3030							
38	554.5518	553.5446							
39	564.3611	563.3538							
40	564.3612	563.3539							
41	579.5359	578.5286							
42	586.4175	585.4103							
43	594.3699	593.3626							
44	605.8036	604.7964							
45	608.3871	607.3798							
46	608.3872	607.3799							
47	615.4051	614.3978							
48	615.4054	614.3981							
49	615.4055	614.3982							
50	617.4071	616.3999							
51	617.4085	616.4013							
52	622.3666	621.3594							
53	630.4438	629.4365							
54	630.4448	629.4376							
55	635.3870	634.3797							
56	638.3899	637.3826							
57	650.2071	649.1998							
58	652.4144	651.4072							
59	657.4451	656.4378							
60	661.5572	660.5500							
61	661.5627	660.5554							
62	661.5634	660.5561							
63	661.5688	660.5615							
65	667.3980	666.3907							
66	674.4709	673.4637							
67	675.6759	674.6686							
68	675.6763	674.6691							
69	675.6772	674.6699							
70	675.6772	674.6699							
71	675.6773	674.6700							
72	675.6779	674.6707							
73	675.6804	674.6731							
75	677.6845	676.6773							
76	682.4243	681.4170							
77	691.3439	690.3367							
78	696.4402	695.4329							
79	696.4407	695.4334							
80	717.6228	716.6155							
81	717.6242	716.6169							
82	717.6261	716.6189							
83	717.6263	716.6191							
84	734.6520	733.6447							
85	739.6048	738.5975							
86	739.6055	738.5982							
87	739.6061	738.5988							
88	739.6067	738.5994							
89	739.6087	738.6015							
90	740.4659	739.4586							
91	741.4703	740.4630							
92	752.5464	751.5391							
93	752.5497	751.5424							
94	760.0343	759.0270							
95	762.5239	761.5166							

☑	97	806.5526	805.5453
☑	106	433.3516	864.6886
☑	107	433.3530	864.6915
☑	111	883.8970	882.8897
☑	118	934.8828	933.8755
☑	119	935.8857	934.8785
☑	121	476.3086	950.6026
☑	124	489.8182	977.6218
☑		613.3621	1224.7097
☑		655.7161	1309.4177
☑		658.2197	1314.4249
☑		658.2556	1314.4966
☑		442.3420	1324.0042
☑		445.8876	1334.6409
☑		674.7033	1347.3921
☑		678.3213	1354.6280
☑		682.2656	1362.5166
☑		682.6921	1363.3697
☑		709.8056	1417.5966
☑		478.7073	1433.1001
☑		721.1686	1440.3226
☑		724.8555	1447.6964
☑		724.8567	1447.6988
☑		732.8477	1463.6808
☑		506.3152	1515.9239
☑		767.8201	1533.6257
☑		512.2159	1533.6257
☑		817.3597	1632.7048
☑		545.8757	1634.6053
☑		551.8934	1652.6583
☑		560.5936	1678.7590
☑		564.3572	1690.0497
☑		582.7990	1745.3751
☑		608.3957	1822.1652
☑		474.8363	1895.3160
☑		640.9231	1919.7475
☑		971.3777	1940.7408
☑		500.3089	1997.2067
☑		504.3019	2013.1786
☑		677.9677	2030.8814
☑		682.9687	2045.8842
☑		412.1706	2055.8164
☑		686.9829	2057.9269
☑		687.9579	2060.8518
☑		687.9582	2060.8529
☑		691.9717	2072.8934
☑		691.9720	2072.8941
☑		691.9720	2072.8941
☑		693.2885	2076.8436
☑		520.3342	2077.3077
☑		695.0068	2081.9987
☑		719.6879	2156.0420
☑		550.3446	2197.3493
☑		735.2932	2202.8578
☑		735.9890	2204.9451
☑		740.4635	2218.3686
☑		755.3482	2263.0228
☑		755.3484	2263.0233
☑	332	1142.0994	2282.1843
☑	333	1142.0997	2282.1848
☑		763.8576	2288.5509
☑		579.5371	2314.1192
☑		773.3438	2317.0096
☑		779.3740	2335.1001
☑		779.3744	2335.1013
☑		614.5618	2454.2182
☑		822.3616	2464.0629
☑		885.7253	2654.1541
☑		885.7258	2654.1556
☑		548.3241	2736.5840
☑		696.4422	2781.7396
☑		957.0045	2867.9916
☑		412.1810	2878.2159
☑		739.6068	2954.3979
☑		741.5703	2962.2523
☑		1004.9767	3011.9082
☑		1004.9795	3011.9167
☑		1042.0025	3122.9858
☑		791.1337	3160.5057
☑		641.8692	3204.3098
☑		414.7504	3309.9453
☑		666.3902	3326.9145
☑		675.6694	3373.3105
☑		867.6195	3466.4489
☑		591.3837	3542.2588
☑		520.3346	3635.2914
☑		734.6531	3668.2291
☑		746.8716	3729.3218
☑		630.2797	3775.6347
☑		759.3032	3791.4798
☑		476.3016	3802.3544
☑		641.8684	3845.1667
☑		555.7503	3883.2008
☑		652.4110	3908.4225
☑		665.3677	3986.1624
☑		579.5022	4049.4646
☑		581.8010	4065.5558
☑		4111.6179	4110.6106
☑		4143.3320	4142.3247
☑	410	1409.2663	4224.7770

411	718.4968	4304.9370
412	544.3281	4346.5668
413	732.8679	4391.1636
416	641.9061	4486.2918
417	4498.1346	4497.1273
418	650.1907	4544.2840
419	4552.6804	4551.6731
420	4552.8576	4551.8503
423	763.8979	4577.3437
424	658.2142	4600.4483
425	578.3454	4618.7049
429	4718.6962	4717.6889
430	4747.6071	4746.5998
431	608.3615	4858.8336
432	696.4430	4868.0501
433	614.5874	4908.6413
434	827.8764	4961.2145
435	4989.1836	4988.1763
437	639.3512	5106.7512
438	730.8820	5109.1231
439	732.8329	5122.7792
440	741.3447	5182.3623
441	650.2215	5193.7136
442	652.4102	5211.2232
443	883.9229	5297.4934
444	887.9724	5321.7905
445	5335.3839	5334.3766
447	5432.9901	5431.9828
449	5463.1891	5462.1818
451	919.1003	5508.5580
452	692.4970	5531.9181
453	5643.2946	5642.2873
454	943.0356	5652.1702
455	5657.2366	5656.2293
456	5690.8136	5689.8063
457	5690.8569	5689.8496
458	5738.0775	5737.0702
459	721.1886	5761.4507
462	1010.9925	6059.9113
464	6123.3826	6122.3753
465	6123.4643	6122.4570
466	6137.7248	6136.7175
467	6213.5748	6212.5675
468	890.8091	6228.6129
471	6364.1120	6363.1047
472	6419.8620	6418.8547
473	6492.0167	6491.0094
474	6529.9144	6528.9071
475	817.3341	6530.6144
476	942.5169	6590.5676
477	827.3401	6610.6627
478	6614.4295	6613.4222
479	6668.8001	6667.7928
480	6855.7952	6854.7879
481	6891.6955	6890.6882
482	6900.3039	6899.2966
483	7036.0719	7035.0646
484	7089.7764	7088.7691
487	7284.5022	7283.4949
488	923.5312	7380.1915
489	7408.1019	7407.0946
490	9220.1483	9219.1410

Search Parameters

Type of search : MS/MS Ion Search
Enzyme : Trypsin
Fixed modifications : Carbamidomethyl (C)
Variable modifications : Oxidation (M)
Mass values : Monoisotopic
Protein Mass : Unrestricted
Peptide Mass Tolerance : ± 0.05 Da
Fragment Mass Tolerance: ± 0.05 Da
Max Missed Cleavages : 2
Instrument type : Default
Number of queries : 490

Mascot Search Results

User : Julian
Email : julian.langer@mpibp-frankfurt.mpg.de
Search title : Submitted from 140318 842 ChKrettler 1-10 swissprot full T 0.05Da by Mascot Daemon on CCSW010
MS data file : D:\Data\1403\140318\140314 842 ChReinhard 8_Tray01-D4_01_17689.d\140314 842 ChReinhard 8_Tray01-D4_01_17689.mgf
Database : SwissProt 57.15 (515203 sequences; 181334896 residues)
Timestamp : 19 Mar 2014 at 09:52:08 GMT
Protein hits :

CFAH_HUMAN	Complement factor H OS=Homo sapiens GN=CFH PE=1 SV=4
TRYP_PIG	Trypsin OS=Sus scrofa PE=1 SV=1
K2C1_HUMAN	Keratin, type II cytoskeletal 1 OS=Homo sapiens GN=KRT1 PE=1 SV=6
K1C10_HUMAN	Keratin, type I cytoskeletal 10 OS=Homo sapiens GN=KRT10 PE=1 SV=6
K1C9_HUMAN	Keratin, type I cytoskeletal 9 OS=Homo sapiens GN=KRT9 PE=1 SV=3
K2C1_RAT	Keratin, type II cytoskeletal 1 OS=Rattus norvegicus GN=Krt1 PE=2 SV=1
K22E_HUMAN	Keratin, type II cytoskeletal 2 epidermal OS=Homo sapiens GN=KRT2 PE=1 SV=2
ALBU_HUMAN	Serum albumin OS=Homo sapiens GN=ALB PE=1 SV=2
K2C6B_HUMAN	Keratin, type II cytoskeletal 6B OS=Homo sapiens GN=KRT6B PE=1 SV=5
IGHG1_HUMAN	Ig gamma-1 chain C region OS=Homo sapiens GN=IGHG1 PE=1 SV=1
A2MG_HUMAN	Alpha-2-macroglobulin OS=Homo sapiens GN=A2M PE=1 SV=2
K1C14_HUMAN	Keratin, type I cytoskeletal 14 OS=Homo sapiens GN=KRT14 PE=1 SV=4
F13B_HUMAN	Coagulation factor XIII B chain OS=Homo sapiens GN=F13B PE=1 SV=3
F13A_HUMAN	Coagulation factor XIII A chain OS=Homo sapiens GN=F13A1 PE=1 SV=4
CO4A_HUMAN	Complement C4-A OS=Homo sapiens GN=C4A PE=1 SV=1

Mascot Score Histogram

Ions score is -10*Log(P), where P is the probability that the observed match is a random event.
Individual ions scores > 38 indicate identity or extensive homology (p<0.05).
Protein scores are derived from ions scores as a non-probabilistic basis for ranking protein hits.

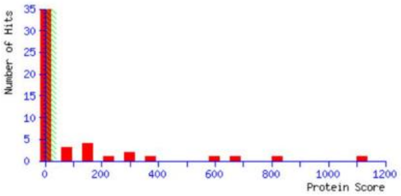

Peptide Summary Report

Format As: Peptide Summary — Help
Significance threshold p< 0.05 — Max. number of hits AUTO
Standard scoring — MudPIT scoring — Ions score or expect cut-off 0 — Show sub-sets 0
Show pop-ups — Suppress pop-ups — Sort unassigned Decreasing Score — Require bold red

Select All | Select None | Search Selected | Error tolerant | Archive Report

1. CFAH_HUMAN **Mass:** 143680 **Score:** 1117 **Matches:** 53(35) **Sequences:** 32(24) **emPAI:** 0.98
Complement factor H OS=Homo sapiens GN=CFH PE=1 SV=4
Check to include this hit in error tolerant search or archive report

Query	Observed	Mr(expt)	Mr(calc)	Delta	Miss	Score	Expect	Rank	Unique	Peptide
132	463.2317	924.4488	924.4454	0.0035	0	(33)	0.25	1	U	R.NGFYPATR.G
133	463.2319	924.4492	924.4454	0.0038	0	(33)	0.26	1	U	R.NGFYPATR.G
134	463.2334	924.4522	924.4454	0.0069	0	41	0.039	1	U	R.NGFYPATR.G
160	532.3011	1062.5876	1062.5822	0.0054	0	63	0.00036	1	U	K.IDVHLVPDR.K
161	532.3013	1062.5880	1062.5822	0.0058	0	(56)	0.0019	1	U	K.IDVHLVPDR.K
180	556.2727	1110.5308	1110.5280	0.0028	0	(18)	6.8	1	U	K.WSHPPSCIK.T
181	556.2731	1110.5317	1110.5280	0.0036	0	32	0.26	1	U	K.WSHPPSCIK.T
187	566.2647	1130.5149	1130.5105	0.0044	0	52	0.002	1	U	K.EFDHNSNIR.Y
188	566.2648	1130.5151	1130.5105	0.0046	0	(37)	0.064	1	U	K.EFDHNSNIR.Y
189	566.2655	1130.5165	1130.5105	0.0061	0	(25)	0.9	1	U	K.EFDHNSNIR.Y
190	567.3293	1132.6440	1132.6393	0.0047	0	47	0.013	1		R.RPYFPVAVGK.Y
192	578.2740	1154.5334	1154.5278	0.0056	0	65	0.00012	1	U	R.TGESVEFVCK.R
198	593.3025	1184.5905	1184.5860	0.0045	0	39	0.067	1	U	K.CLPVTAPENGK.I
199	593.8004	1185.5862	1185.5819	0.0044	1	(46)	0.014	1	U	R.TKNDFTWFK.L
200	593.8014	1185.5883	1185.5819	0.0064	1	55	0.0016	1	U	R.TKNDFTWFK.L
202	595.2713	1188.5280	1188.5234	0.0046	0	47	0.0045	1	U	K.LSYTCEGGFR.I
203	595.2718	1188.5291	1188.5234	0.0057	0	(43)	0.011	1	U	K.LSYTCEGGFR.I
211	405.1911	1212.5515	1212.5458	0.0057	0	(24)	1.2	1	U	K.HGGLYHENMR.R
217	410.5183	1228.5331	1228.5407	-0.0076	0	24	0.64	1	U	K.HGGLYHENMR.R + Oxidation (M)
225	621.7764	1241.5382	1241.5347	0.0036	0	36	0.038	1	U	R.TGDEITYQCR.N
226	623.3088	1244.6030	1244.5972	0.0058	0	(64)	0.00021	1	U	K.CTSTGWIPAPR.C
227	623.3092	1244.6038	1244.5972	0.0066	0	65	0.00017	1	U	K.CTSTGWIPAPR.C
230	630.3284	1258.6423	1258.6380	0.0044	0	29	0.81	1	U	R.WQSIPLCVEK.I
241	655.3074	1308.6002	1308.5955	0.0047	0	(41)	0.025	1	U	K.SCDIPVFMNAR.T
242	655.3074	1308.6002	1308.5955	0.0048	0	49	0.0042	1	U	K.SCDIPVFMNAR.T
249	663.3043	1324.5940	1324.5904	0.0037	0	(42)	0.016	1	U	K.SCDIPVFMNAR.T + Oxidation (M)
257	671.3577	1340.7008	1340.6936	0.0072	0	62	0.00037	1	U	K.SPDVINGSPISQK.I
265	691.3452	1380.6758	1380.6707	0.0051	0	61	0.00043	1	U	R.EIMENYNIALR.W + Oxidation (M)
271	465.9346	1394.7819	1394.7769	0.0050	0	(17)	8.5	1	U	K.SSNLIIILEEHLK.N
272	698.3982	1394.7819	1394.7769	0.0050	0	60	0.00044	1	U	K.SSNLIIILEEHLK.N
273	481.2406	1440.7000	1440.6932	0.0068	0	33	0.22	1	U	K.EGWIHTVCINGR.W
278	741.3608	1480.7069	1480.7021	0.0049	0	79	5.9e-006	1	U	K.CFEGFGIDGPAIAK.C
280	503.9099	1508.7080	1508.7003	0.0077	0	10	39	1	U	R.CTLKPCDYPDIK.H
289	827.8778	1653.7410	1653.7345	0.0066	0	59	0.00023	1	U	K.AGEQVTYTCATYYK.M
290	827.8797	1653.7449	1653.7345	0.0105	0	(17)	4.2	1	U	K.AGEQVTYTCATYYK.M
302	908.4298	1814.8451	1814.8357	0.0095	0	87	4.8e-007	1	U	K.LGYVTADGETSGSITCGK.D
306	934.4218	1866.8290	1866.8206	0.0084	0	6	35	1	U	K.SCDNPYIPNGDYSPLR.I
311	983.4920	1964.9694	1964.9626	0.0069	0	78	6e-006	1	U	K.EQVQSCGPPPELLNGNVK.E
312	983.4923	1964.9699	1964.9626	0.0074	0	(69)	5.5e-005	1	U	K.EQVQSCGPPPELLNGNVK.E
314	1004.9758	2007.9370	2007.9281	0.0089	0	53	0.0013	1	U	K.TDCLSLPSFENAIPMGEK.K
316	1010.9970	2019.9794	2019.9684	0.0110	0	(58)	0.00066	1	U	R.DTSCVNPPTVQNAYIVSR.Q
317	1010.9972	2019.9798	2019.9684	0.0114	0	86	8.9e-007	1	U	R.DTSCVNPPTVQNAYIVSR.Q
329	552.7704	2207.0523	2207.0429	0.0094	1	42	0.02	1	U	K.IVSSAMEPDREYHFGQAVR.F + Oxidation (M)
330	552.7708	2207.0542	2207.0429	0.0112	1	(3)	1.3e+002	1	U	K.IVSSAMEPDREYHFGQAVR.F + Oxidation (M)
342	754.0085	2259.0037	2258.9902	0.0135	1	(44)	0.0035	1	U	K.TKEEYGHSEVVEYYCNPR.F
343	754.0086	2259.0038	2258.9902	0.0136	1	48	0.0014	1	U	K.TKEEYGHSEVVEYYCNPR.F
344	754.0093	2259.0062	2258.9902	0.0160	1	(30)	0.093	1	U	K.TKEEYGHSEVVEYYCNPR.F
348	1133.0546	2264.0946	2264.0848	0.0098	0	102	1.9e-008	1	U	K.SSIDIENGFISESQYTYALK.E
363	1206.5798	2411.1451	2411.1348	0.0103	0	31	0.21	1	U	K.VSVLCQENYLIQEGEEITCK.D
378	914.1078	2739.3015	2739.2844	0.0171	1	(43)	0.011	1	U	K.VSVLCQENYLIQEGEEITCKDGR.W
379	914.1078	2739.3017	2739.2844	0.0174	1	60	0.0002	1	U	K.VSVLCQENYLIQEGEEITCKDGR.W
380	914.1121	2739.3144	2739.2844	0.0301	1	(38)	0.041	1	U	K.VSVLCQENYLIQEGEEITCKDGR.W
388	732.3400	2925.3307	2925.3127	0.0180	0	38	0.016	1	U	K.SPPEISHGVVAHMSDSYQYGEEVTYK.C + Oxidation (M)

2. TRYP_PIG **Mass:** 25078 **Score:** 845 **Matches:** 28(20) **Sequences:** 9(7) **emPAI:** 3.29
Trypsin OS=Sus scrofa PE=1 SV=1
Check to include this hit in error tolerant search or archive report

Query	Observed	Mr(expt)	Mr(calc)	Delta	Miss	Score	Expect	Rank	Unique	Peptide
120	421.7603	841.5060	841.5022	0.0039	0	(52)	0.0047	1	U	R.VATVSLPR.S
121	421.7605	841.5065	841.5022	0.0044	0	(49)	0.0081	1	U	R.VATVSLPR.S

122	421.7607	841.5068	841.5022	0.0047	0	55	0.0023	1	U	R.VATVSLPR.S
154	523.2873	1044.5600	1044.5564	0.0037	0	68	0.00016	1	U	K.LSSPATLNSR.V
155	523.2874	1044.5602	1044.5564	0.0038	0	(55)	0.0027	1	U	K.LSSPATLNSR.V
156	523.2876	1044.5607	1044.5564	0.0043	0	(50)	0.0096	1	U	K.LSSPATLNSR.V
157	532.2598	1062.5051	1062.5016	0.0035	0	(94)	2.4e-007	1	U	K.APVLSDSSCK.S
158	532.2601	1062.5057	1062.5016	0.0042	0	94	2.3e-007	1	U	K.APVLSDSSCK.S
159	532.2604	1062.5062	1062.5016	0.0047	0	(94)	2.4e-007	1	U	K.APVLSDSSCK.S
281	763.8794	1525.7441	1525.7446	-0.0005	0	(53)	0.0024	1	U	K.SSGSSYPSLLQCLK.A
282	763.8811	1525.7477	1525.7446	0.0031	0	74	1.8e-005	1	U	K.SSGSSYPSLLQCLK.A
331	737.7062	2210.0967	2210.0967	-0.0000	0	(8)	66	2	U	R.LGEHNIDVLEGNEQFINAAK.I
332	737.7098	2210.1077	2210.0967	0.0110	0	(100)	4.2e-008	1	U	R.LGEHNIDVLEGNEQFINAAK.I
333	737.7101	2210.1084	2210.0967	0.0117	0	103	2e-008	1	U	R.LGEHNIDVLEGNEQFINAAK.I
334	1106.0615	2210.1084	2210.0967	0.0117	0	(18)	6.8	1	U	R.LGEHNIDVLEGNEQFINAAK.I
335	1106.0616	2210.1087	2210.0967	0.0120	0	(40)	0.042	1	U	R.LGEHNIDVLEGNEQFINAAK.I
336	737.7102	2210.1087	2210.0967	0.0120	0	(101)	3e-008	1	U	R.LGEHNIDVLEGNEQFINAAK.I
338	1108.0351	2214.0557	2214.0449	0.0107	0	17	6	1	U	K.SSYPGQITGNMICVGFLEGGK.D
350	761.7360	2282.1861	2282.1729	0.0132	0	(56)	0.00096	1	U	K.IITHPNFNGNTLDNDIMLIK.L
352	761.7361	2282.1866	2282.1729	0.0137	0	(56)	0.00089	1	U	K.IITHPNFNGNTLDNDIMLIK.L
356	767.0666	2298.1781	2298.1678	0.0103	0	(32)	0.23	1	U	K.IITHPNFNGNTLDNDIMLIK.L + Oxidation (M)
357	767.0676	2298.1810	2298.1678	0.0132	0	68	5.3e-005	1	U	K.IITHPNFNGNTLDNDIMLIK.L + Oxidation (M)
374	903.1478	2706.4216	2706.4089	0.0127	1	(0)	2.5e+002	1	U	R.IQVRLGEHNIDVLEGNEQFINAAK.I
375	903.1597	2706.4572	2706.4089	0.0484	1	22	1.2	1	U	R.IQVRLGEHNIDVLEGNEQFINAAK.I
400	828.1916	3308.7373	3308.7187	0.0186	1	68	2.9e-005	1	U	K.IITHPNFNGNTLDNDIMLIKLSSPATLNSR.V
401	828.1916	3308.7375	3308.7187	0.0188	1	(54)	0.00059	1	U	K.IITHPNFNGNTLDNDIMLIKLSSPATLNSR.V
403	832.1916	3324.7372	3324.7136	0.0236	1	(30)	0.16	1	U	K.IITHPNFNGNTLDNDIMLIKLSSPATLNSR.V + Oxidation (M)
404	832.1956	3324.7531	3324.7136	0.0395	1	(16)	4.2	1	U	K.IITHPNFNGNTLDNDIMLIKLSSPATLNSR.V + Oxidation (M)

3. K2C1_HUMAN **Mass:** 66170 **Score:** 695 **Matches:** 34(22) **Sequences:** 19(14) **emPAI:** 1.38
Keratin, type II cytoskeletal 1 OS=Homo sapiens GN=KRT1 PE=1 SV=6
Check to include this hit in error tolerant search or archive report

Query	Observed	Mr(expt)	Mr(calc)	Delta	Miss	Score	Expect	Rank	Unique	Peptide
129	437.7555	873.4964	873.4920	0.0044	0	34	0.46	1	U	R.SLVNLGGSK.S
139	487.2715	972.5285	972.5240	0.0045	0	44	0.04	1		K.IEISELNR.V
140	487.2718	972.5290	972.5240	0.0050	0	(44)	0.043	1		K.IEISELNR.V
164	533.2660	1064.5175	1064.5138	0.0037	0	(35)	0.17	1		K.AQYEDIAQK.S
165	533.2664	1064.5182	1064.5138	0.0044	0	41	0.049	1		K.AQYEDIAQK.S
166	533.2665	1064.5185	1064.5138	0.0047	0	(27)	1.1	1		K.AQYEDIAQK.S
167	537.3032	1072.5918	1072.5876	0.0042	1	(21)	7.1	4		R.LRSEIDNVK.K
168	537.3036	1072.5926	1072.5876	0.0050	1	37	0.2	2		R.LRSEIDNVK.K
197	590.3061	1178.5977	1178.5931	0.0046	0	53	0.0031	1		K.YEELQITAGR.H
205	401.2367	1200.6882	1200.6826	0.0056	2	47	0.013	1		R.LRSEIDNVKK.Q
206	601.3514	1200.6882	1200.6826	0.0056	2	(34)	0.25	1		R.LRSEIDNVKK.Q
207	401.2367	1200.6882	1200.6826	0.0056	2	(24)	2.8	1		R.LRSEIDNVKK.Q
231	633.3247	1264.6349	1264.6299	0.0050	0	90	6.3e-007	1		R.TNAENEFVTIK.K
232	633.3252	1264.6358	1264.6299	0.0059	0	(82)	4.3e-006	1		R.TNAENEFVTIK.K
234	639.3611	1276.7076	1276.7027	0.0049	0	61	0.00042	1	U	K.LALDLEIATYR.T
238	651.8638	1301.7130	1301.7078	0.0052	0	74	2.9e-005	1		R.SLDLDSIIAEVK.A
239	436.8920	1307.6541	1307.6469	0.0071	2	(46)	0.016	1		R.NKYEDEINKR.T
240	436.8922	1307.6548	1307.6469	0.0079	2	56	0.0015	1		R.NKYEDEINKR.T
253	670.8405	1339.6665	1339.6619	0.0046	1	(60)	0.00047	1	U	K.SKAEAESLYQSK.Y
254	670.8410	1339.6675	1339.6619	0.0056	1	68	8.8e-005	1	U	K.SKAEAESLYQSK.Y
255	670.8413	1339.6680	1339.6619	0.0061	1	(57)	0.001	1	U	K.SKAEAESLYQSK.Y
256	670.8416	1339.6687	1339.6619	0.0068	1	(62)	0.00036	1	U	K.SKAEAESLYQSK.Y
266	692.3515	1382.6884	1382.6830	0.0054	0	57	0.00096	1	U	K.SLNNQFASFIDK.V
268	697.3725	1392.7305	1392.7249	0.0057	1	(51)	0.0051	1		R.TNAENEFVTIKK.D
269	465.2508	1392.7305	1392.7249	0.0057	1	(28)	0.93	1		R.TNAENEFVTIKK.D
270	697.3733	1392.7320	1392.7249	0.0072	1	71	4.3e-005	1		R.TNAENEFVTIKK.D
275	738.3811	1474.7477	1474.7416	0.0061	0	57	0.0012	1		K.WELLQQVDTSTR.T
276	738.3994	1474.7842	1474.7780	0.0062	0	(91)	5.7e-007	1		R.FLEQQNQVLQTK.W
277	738.3999	1474.7852	1474.7780	0.0073	0	91	4.9e-007	1		R.FLEQQNQVLQTK.W
285	800.4249	1598.8353	1598.8264	0.0089	1	65	0.00018	1	U	K.NKLNDLEDALQQAK.E
315	673.3045	2016.8918	2016.8768	0.0150	1	8	15	1	U	R.LDSELKNMQDMVEDYR.N + 2 Oxidation (M)
327	728.7164	2183.1272	2183.1182	0.0091	2	(13)	23	1	U	K.NKLNDLEDALQQAKEDLAR.L
328	728.7164	2183.1275	2183.1182	0.0093	2	30	0.4	1	U	K.NKLNDLEDALQQAKEDLAR.L
359	777.0620	2328.1642	2328.1669	-0.0027	1	4	1.4e+002	1	U	K.QISNLQQSISDAEQRGENALK.D

4. K1C10_HUMAN **Mass:** 59020 **Score:** 582 **Matches:** 34(22) **Sequences:** 18(14) **emPAI:** 1.49
Keratin, type I cytoskeletal 10 OS=Homo sapiens GN=KRT10 PE=1 SV=6
Check to include this hit in error tolerant search or archive report

Query	Observed	Mr(expt)	Mr(calc)	Delta	Miss	Score	Expect	Rank	Unique	Peptide
111	404.2054	806.3962	806.3923	0.0040	0	62	0.00054	1		R.LAADDFR.L
112	404.2055	806.3964	806.3923	0.0042	0	(31)	0.66	1		R.LAADDFR.L
113	405.2253	808.4361	808.4330	0.0031	0	40	0.075	1		R.LASYLDK.V
142	498.2656	994.5167	994.5123	0.0044	1	33	0.33	1		K.IKEWYEK.H
143	498.2659	994.5172	994.5123	0.0049	1	(21)	5.1	1		K.IKEWYEK.H
144	502.2813	1002.5480	1002.5458	0.0022	1	33	0.57	1		K.SEITELRR.N
145	502.2819	1002.5493	1002.5458	0.0036	1	(32)	0.57	1		K.SEITELRR.N
146	502.2823	1002.5500	1002.5458	0.0042	1	(27)	1.7	1		K.SEITELRR.N
149	516.3045	1030.5944	1030.5910	0.0034	0	(15)	22	1		R.VLDELTLTK.A
150	516.3045	1030.5944	1030.5910	0.0034	0	48	0.01	1		R.VLDELTLTK.A
151	516.3056	1030.5966	1030.5910	0.0056	0	(39)	0.094	1		R.VLDELTLTK.A
162	532.8106	1063.6066	1063.6026	0.0040	1	(46)	0.013	1		R.LASYLDKVR.A
163	532.8112	1063.6079	1063.6026	0.0053	1	51	0.0043	1		R.LASYLDKVR.A
171	545.7668	1089.5190	1089.5237	-0.0047	0	(42)	0.032	1		K.VTMQNLNDR.L
172	545.7710	1089.5274	1089.5237	0.0037	0	72	4.3e-005	1		K.VTMQNLNDR.L
173	545.7710	1089.5275	1089.5237	0.0038	0	(49)	0.0075	1		K.VTMQNLNDR.L
178	555.2510	1108.4875	1108.4825	0.0050	0	(41)	0.019	1		K.DAEAWFNEK.S
179	555.2513	1108.4879	1108.4825	0.0054	0	43	0.013	1		K.DAEAWFNEK.S
221	617.8456	1233.6765	1233.6717	0.0049	1	(64)	0.00026	1	U	R.LKYENEVALR.Q
222	617.8465	1233.6784	1233.6717	0.0067	1	(44)	0.025	1	U	R.LKYENEVALR.Q
223	617.8469	1233.6793	1233.6717	0.0076	1	65	0.0002	1	U	R.LKYENEVALR.Q
258	679.3659	1356.7173	1356.7110	0.0064	1	(23)	3.1	1		R.QSVEADINGLRR.V
259	679.3667	1356.7188	1356.7110	0.0078	1	43	0.031	1		R.QSVEADINGLRR.V
262	683.3308	1364.6470	1364.6320	0.0149	0	53	0.002	1	U	R.SQYEQLAEQNR.K
263	691.3314	1380.6483	1380.6408	0.0075	0	(75)	1.1e-005	1	U	R.ALEESNYELEGK.I
264	691.3317	1380.6488	1380.6408	0.0079	0	80	3.4e-006	1	U	R.ALEESNYELEGK.I
267	695.8456	1389.6767	1389.6736	0.0032	0	47	0.01	1		K.QSLEASLAETEGR.Y
279	747.3743	1492.7341	1492.7270	0.0071	1	50	0.0046	1	U	R.SQYEQLAEQNRK.D
300	899.0135	1796.0125	1796.0043	0.0082	0	76	6.9e-006	1	U	R.NVQALEIELQSQLALK.Q
301	899.0136	1796.0127	1796.0043	0.0084	0	(54)	0.00099	1	U	R.NVQALEIELQSQLALK.Q
313	998.9934	1995.9722	1995.9636	0.0086	0	94	1.6e-007	1	U	K.ELTTEIDNNIEQISSYK.S
337	738.0412	2211.1017	2211.0906	0.0111	1	56	0.001	1	U	K.SKELTTEIDNNIEQISSYK.S
339	742.0547	2223.1422	2223.1344	0.0079	1	(30)	0.39	1	U	K.ADLEMQIESLTEELAYLKK.N
340	742.0558	2223.1457	2223.1344	0.0113	1	35	0.12	1	U	K.ADLEMQIESLTEELAYLKK.N

5. K1C9_HUMAN **Mass:** 62255 **Score:** 359 **Matches:** 16(10) **Sequences:** 10(7) **emPAI:** 0.72
Keratin, type I cytoskeletal 9 OS=Homo sapiens GN=KRT9 PE=1 SV=3
Check to include this hit in error tolerant search or archive report

Query	Observed	Mr(expt)	Mr(calc)	Delta	Miss	Score	Expect	Rank	Unique	Peptide
113	405.2253	808.4361	808.4330	0.0031	0	40	0.075	1		R.LASYLDK.V
185	561.2978	1120.5811	1120.5764	0.0047	0	43	0.04	1	U	R.QEYEQLIAK.N
193	579.3017	1156.5888	1156.5836	0.0051	0	53	0.0027	1	U	R.QGVDADINGLR.Q
204	595.8104	1189.6063	1189.6013	0.0051	0	46	0.018	1	U	R.QVLDNLTMEK.S
208	603.8088	1205.6031	1205.5962	0.0069	0	(39)	0.083	1	U	R.QVLDNLTMEK.S + Oxidation (M)
218	616.8046	1231.5946	1231.5906	0.0041	0	(67)	0.00011	1	U	R.SGGGGGGGLGSGGSIR.S
219	616.8051	1231.5957	1231.5906	0.0051	0	85	1.8e-006	1	U	R.SGGGGGGGLGSGGSIR.S
220	616.8053	1231.5959	1231.5906	0.0054	0	(72)	4e-005	1	U	R.SGGGGGGGLGSGGSIR.S
224	618.2708	1234.5270	1234.5215	0.0056	0	51	0.0012	1	U	R.FSSSSGYGGGSSR.V
246	662.3427	1322.6708	1322.6652	0.0056	1	(22)	3.9	1	U	R.IKFEMEQNLR.Q + Oxidation (M)
247	662.3427	1322.6709	1322.6652	0.0057	1	(21)	5	1	U	R.IKFEMEQNLR.Q + Oxidation (M)
248	662.3431	1322.6716	1322.6652	0.0064	1	40	0.066	1	U	R.IKFEMEQNLR.Q + Oxidation (M)
304	613.3294	1836.9665	1836.9581	0.0084	0	38	0.074	1	U	R.HGVQELEIELQSQLSK.K

369	837.3864	2509.1373	2509.1245	0.0128	0	35	0.038	1	U	K.EIETYHNLLEGGQEDFESSGAGK.I
397	1088.8470	3263.5191	3263.5066	0.0125	0	(65)	4.3e-005	1	U	K.DIENQYETQITQIEHEVSSSGQEVQSSAK.E
398	1088.8484	3263.5233	3263.5066	0.0168	0	69	1.7e-005	1	U	K.DIENQYETQITQIEHEVSSSGQEVQSSAK.E

6. K2C1_RAT **Mass:** 65190 **Score:** 332 **Matches:** 9(7) **Sequences:** 5(4) **emPAI:** 0.30
Keratin, type II cytoskeletal 1 OS=Rattus norvegicus GN=Krt1 PE=2 SV=1
Check to include this hit in error tolerant search or archive report

Query	Observed	Mr(expt)	Mr(calc)	Delta	Miss	Score	Expect	Rank	Unique	Peptide
169	541.8061	1081.5977	1081.5920	0.0057	1	38	0.084	1		K.FASFIDKVR.F
231	633.3247	1264.6349	1264.6299	0.0050	0	90	6.3e-007	1		R.TNAENEFVTIK.K
232	633.3252	1264.6358	1264.6299	0.0059	0	(82)	4.3e-006	1		R.TNAENEFVTIK.K
268	697.3725	1392.7305	1392.7249	0.0057	1	(51)	0.0051	1		R.TNAENEFVTIKK.D
269	465.2508	1392.7305	1392.7249	0.0057	1	(28)	0.93	1		R.TNAENEFVTIKK.D
270	697.3733	1392.7320	1392.7249	0.0072	1	71	4.3e-005	1		R.TNAENEFVTIKK.D
275	738.3811	1474.7477	1474.7416	0.0061	0	57	0.0012	1		K.WELLQQVDTSTR.T
276	738.3994	1474.7842	1474.7780	0.0062	0	(91)	5.7e-007	1		R.FLEQQNQVLQTK.W
277	738.3999	1474.7852	1474.7780	0.0073	0	91	4.9e-007	1		R.FLEQQNQVLQTK.W

7. K22E_HUMAN **Mass:** 65678 **Score:** 295 **Matches:** 13(9) **Sequences:** 9(6) **emPAI:** 0.43
Keratin, type II cytoskeletal 2 epidermal OS=Homo sapiens GN=KRT2 PE=1 SV=2
Check to include this hit in error tolerant search or archive report

Query	Observed	Mr(expt)	Mr(calc)	Delta	Miss	Score	Expect	Rank	Unique	Peptide
139	487.2715	972.5285	972.5240	0.0045	0	44	0.04	1		K.IEISELNR.V
140	487.2718	972.5290	972.5240	0.0050	0	(44)	0.043	1		K.IEISELNR.V
141	497.7895	993.5645	993.5607	0.0038	0	5	1.5e+002	3	U	R.LQGEIAHVK.K
153	521.2798	1040.5451	1040.5502	-0.0051	0	7	98	3		K.VDPEIQNVK.A
169	541.8061	1081.5977	1081.5920	0.0057	1	38	0.084	1		K.FASFIDKVR.F
176	554.2761	1106.5376	1106.5356	0.0020	0	43	0.035	1		K.AQYEEIAQR.S
177	554.2762	1106.5379	1106.5356	0.0023	0	(26)	1.5	1		K.AQYEEIAQR.S
228	627.8098	1253.6051	1253.6001	0.0051	0	66	0.00012	1		R.GFSSGSAVVSGGSR.R
250	665.3694	1328.7243	1328.7187	0.0056	0	(57)	0.0014	1		R.NLDLDSIIAEVK.A
251	665.3696	1328.7245	1328.7187	0.0058	0	82	4.2e-006	1		R.NLDLDSIIAEVK.A
252	668.8613	1335.7080	1335.7034	0.0046	1	50	0.0054	1	U	R.TAAENDFVTLKK.D
276	738.3994	1474.7842	1474.7780	0.0062	0	(91)	5.7e-007	1		R.FLEQQNQVLQTK.W
277	738.3999	1474.7852	1474.7780	0.0073	0	91	4.9e-007	1		R.FLEQQNQVLQTK.W

8. ALBU_HUMAN **Mass:** 71317 **Score:** 188 **Matches:** 14(5) **Sequences:** 10(4) **emPAI:** 0.46
Serum albumin OS=Homo sapiens GN=ALB PE=1 SV=2
Check to include this hit in error tolerant search or archive report

Query	Observed	Mr(expt)	Mr(calc)	Delta	Miss	Score	Expect	Rank	Unique	Peptide
135	464.2527	926.4907	926.4861	0.0046	0	31	0.37	1		K.YLYEIAR.R
137	480.7870	959.5595	959.5552	0.0043	0	46	0.019	1	U	K.FQNALLVR.Y
138	480.7876	959.5607	959.5552	0.0054	0	(35)	0.25	1	U	K.FQNALLVR.Y
147	1013.6035	1012.5962	1012.5917	0.0046	0	27	1.1	1	U	K.LVAASQAALGL.-
186	564.8554	1127.6962	1127.6914	0.0048	1	28	0.53	1	U	K.KQTALVELVK.H
191	575.3143	1148.6140	1148.6077	0.0062	0	65	0.00027	1	U	K.LVNEVTEFAK.T
215	613.8088	1225.6031	1225.5979	0.0052	1	(34)	0.19	1	U	R.FKDLGEENFK.A
216	613.8090	1225.6034	1225.5979	0.0056	1	39	0.062	1	U	R.FKDLGEENFK.A
274	722.3282	1442.6419	1442.6347	0.0072	0	52	0.001	1	U	K.YICENQDSISSK.L
283	516.2724	1545.7955	1545.7894	0.0060	1	16	17	1	U	K.LKECCEKPLLEK.S
286	547.3207	1638.9404	1638.9305	0.0099	1	63	0.00013	1		K.KVPQVSTPTLVEVSR.N
287	547.3209	1638.9409	1638.9305	0.0104	1	(54)	0.001	1		K.KVPQVSTPTLVEVSR.N
318	682.3738	2044.0995	2044.0881	0.0115	0	29	0.43	1	U	K.VFDEFKPLVEEPQNLIK.Q
319	682.3739	2044.0999	2044.0881	0.0119	0	(21)	2.7	1	U	K.VFDEFKPLVEEPQNLIK.Q

Proteins matching the same set of peptides:
ALBU_PONAB **Mass:** 71317 **Score:** 188 **Matches:** 14(5) **Sequences:** 10(4)
Serum albumin OS=Pongo abelii GN=ALB PE=2 SV=1

9. K2C6B_HUMAN **Mass:** 60315 **Score:** 172 **Matches:** 9(6) **Sequences:** 6(4) **emPAI:** 0.40
Keratin, type II cytoskeletal 6B OS=Homo sapiens GN=KRT6B PE=1 SV=5
Check to include this hit in error tolerant search or archive report

Query	Observed	Mr(expt)	Mr(calc)	Delta	Miss	Score	Expect	Rank	Unique	Peptide
169	541.8061	1081.5977	1081.5920	0.0057	1	38	0.084	1		K.FASFIDKVR.F
176	554.2761	1106.5376	1106.5356	0.0020	0	43	0.035	1		K.AQYEEIAQR.S
177	554.2762	1106.5379	1106.5356	0.0023	0	(26)	1.5	1		K.AQYEEIAQR.S
197	590.3061	1178.5977	1178.5931	0.0046	0	53	0.0031	1		K.YEELQITAGR.H
239	436.8920	1307.6541	1307.6469	0.0071	2	(46)	0.016	1		K.NKYEDEINKR.T
240	436.8922	1307.6548	1307.6469	0.0079	2	56	0.0015	1		K.NKYEDEINKR.T
250	665.3694	1328.7243	1328.7187	0.0056	0	(57)	0.0014	1		R.NLDLDSIIAEVK.A
251	665.3696	1328.7245	1328.7187	0.0058	0	82	4.2e-006	1		R.NLDLDSIIAEVK.A
260	679.3731	1356.7316	1356.7249	0.0067	1	38	0.12	1	U	K.NKLEGLEDALQK.A

10. IGHG1_HUMAN **Mass:** 36596 **Score:** 164 **Matches:** 8(5) **Sequences:** 6(4) **emPAI:** 0.58
Ig gamma-1 chain C region OS=Homo sapiens GN=IGHG1 PE=1 SV=1
Check to include this hit in error tolerant search or archive report

Query	Observed	Mr(expt)	Mr(calc)	Delta	Miss	Score	Expect	Rank	Unique	Peptide
119	419.7571	837.4996	837.4960	0.0036	0	43	0.018	1	U	K.ALPAPIEK.T
194	581.3209	1160.6272	1160.6223	0.0049	0	(37)	0.18	1	U	K.NQVSLTCLVK.G
195	581.3210	1160.6275	1160.6223	0.0051	0	40	0.09	1	U	K.NQVSLTCLVK.G
201	593.8296	1185.6445	1185.6394	0.0052	0	63	0.00035	1	U	K.GPSVFPLAPSSK.S
244	661.3450	1320.6755	1320.6708	0.0047	0	63	0.00034	1	U	K.STSGGTAALGCLVK.D
245	661.3453	1320.6760	1320.6708	0.0052	0	(51)	0.0048	1	U	K.STSGGTAALGCLVK.D
292	839.4093	1676.8041	1676.7947	0.0094	0	42	0.024	1	U	K.FNWYVDGVEVHNAK.T
307	937.4683	1872.9221	1872.9146	0.0076	0	10	44	1	U	K.TTPPVLDSDGSFFLYSK.L

11. A2MG_HUMAN **Mass:** 164614 **Score:** 141 **Matches:** 6(6) **Sequences:** 4(4) **emPAI:** 0.11
Alpha-2-macroglobulin OS=Homo sapiens GN=A2M PE=1 SV=2
Check to include this hit in error tolerant search or archive report

Query	Observed	Mr(expt)	Mr(calc)	Delta	Miss	Score	Expect	Rank	Unique	Peptide
209	605.8271	1209.6396	1209.6353	0.0043	0	49	0.0066	1	U	K.LPPNVVEESAR.A
210	605.8271	1209.6397	1209.6353	0.0044	0	(46)	0.014	1	U	K.LPPNVVEESAR.A
229	630.2915	1258.5683	1258.5652	0.0031	0	60	0.00023	1	U	R.VGFYESDVMGR.G
233	638.2900	1274.5655	1274.5602	0.0053	0	(50)	0.0018	1	U	R.VGFYESDVMGR.G + Oxidation (M)
305	923.0198	1844.0251	1844.0295	-0.0044	0	48	0.0054	1	U	R.LLIYAVLPTGDVIGDSAK.Y
308	942.5307	1883.0468	1883.0404	0.0064	0	61	0.00026	1	U	R.VSVQLEASPAFLAVPVEK.E

Proteins matching the same set of peptides:
A2MG_PONAB **Mass:** 164585 **Score:** 141 **Matches:** 6(6) **Sequences:** 4(4)
Alpha-2-macroglobulin OS=Pongo abelii GN=A2M PE=2 SV=1

12. K1C14_HUMAN **Mass:** 51872 **Score:** 121 **Matches:** 9(6) **Sequences:** 5(3) **emPAI:** 0.21

Keratin, type I cytoskeletal 14 OS=Homo sapiens GN=KRT14 PE=1 SV=4

Check to include this hit in error tolerant search or archive report

Query	Observed	Mr(expt)	Mr(calc)	Delta	Miss	Score	Expect	Rank	Unique	Peptide
111	404.2054	806.3962	806.3923	0.0040	0	62	0.00054	1		R.LAADDFR.T
112	404.2055	806.3964	806.3923	0.0042	0	(31)	0.66	1		R.LAADDFR.T
113	405.2253	808.4361	808.4330	0.0031	0	40	0.075	1		R.LASYLDK.V
148	**515.3027**	**1028.5908**	**1028.5866**	**0.0042**	**0**	**42**	**0.057**	**1**		**R.VLDELTLAR.A**
162	532.8106	1063.6066	1063.6026	0.0040	1	(46)	0.013	1		R.LASYLDKVR.A
163	532.8112	1063.6079	1063.6026	0.0053	1	51	0.0043	1		R.LASYLDKVR.A
171	545.7668	1089.5190	1089.5237	-0.0047	0	(42)	0.032	1		K.VTMQNLNDR.L
172	545.7710	1089.5274	1089.5237	0.0037	0	72	4.3e-005	1		K.VTMQNLNDR.L
173	545.7710	1089.5275	1089.5237	0.0038	0	(49)	0.0075	1		K.VTMQNLNDR.L

Proteins matching the same set of peptides:

K1C15_MOUSE **Mass:** 49278 **Score:** 121 **Matches:** 9(6) **Sequences:** 5(3)
Keratin, type I cytoskeletal 15 OS=Mus musculus GN=Krt15 PE=1 SV=2

K1C15_RAT **Mass:** 49011 **Score:** 121 **Matches:** 9(6) **Sequences:** 5(3)
Keratin, type I cytoskeletal 15 OS=Rattus norvegicus GN=Krt15 PE=1 SV=1

K1C16_HUMAN **Mass:** 51578 **Score:** 121 **Matches:** 9(6) **Sequences:** 5(3)
Keratin, type I cytoskeletal 16 OS=Homo sapiens GN=KRT16 PE=1 SV=4

---

13. F13B_HUMAN **Mass:** 77742 **Score:** 55 **Matches:** 1(1) **Sequences:** 1(1) **emPAI:** 0.04

Coagulation factor XIII B chain OS=Homo sapiens GN=F13B PE=1 SV=3

Check to include this hit in error tolerant search or archive report

Query	Observed	Mr(expt)	Mr(calc)	Delta	Miss	Score	Expect	Rank	Unique	Peptide
323	**695.0072**	**2081.9997**	**2081.9840**	**0.0156**	**0**	**55**	**0.001**	1	**U**	**K.VACEEPPFIENGAANLHSK.I**

---

14. F13A_HUMAN **Mass:** 83728 **Score:** 55 **Matches:** 2(1) **Sequences:** 2(1) **emPAI:** 0.04

Coagulation factor XIII A chain OS=Homo sapiens GN=F13A1 PE=1 SV=4

Check to include this hit in error tolerant search or archive report

Query	Observed	Mr(expt)	Mr(calc)	Delta	Miss	Score	Expect	Rank	Unique	Peptide
235	**640.8517**	**1279.6889**	**1279.6846**	**0.0043**	**0**	**55**	**0.0019**	**1**	**U**	**R.LALETALMYGAK.K**
248	662.3431	1322.6716	1322.6864	-0.0148	1	6	1.6e+002	10	U	R.KLIASMSSDSLR.H + Oxidation (M)

---

15. CO4A_HUMAN **Mass:** 194247 **Score:** 39 **Matches:** 1(0) **Sequences:** 1(0) **emPAI:** 0.02

Complement C4-A OS=Homo sapiens GN=C4A PE=1 SV=1

Check to include this hit in error tolerant search or archive report

Query	Observed	Mr(expt)	Mr(calc)	Delta	Miss	Score	Expect	Rank	Unique	Peptide
152	**519.7847**	**1037.5548**	**1037.5506**	**0.0043**	**0**	**39**	**0.075**	1	**U**	**K.DHAVDLIQK.G**

Proteins matching the same set of peptides:

CO4B_HUMAN **Mass:** 194212 **Score:** 39 **Matches:** 1(0) **Sequences:** 1(0)
Complement C4-B OS=Homo sapiens GN=C4B PE=1 SV=1

CO4_RAT **Mass:** 193639 **Score:** 39 **Matches:** 1(0) **Sequences:** 1(0)
Complement C4 OS=Rattus norvegicus GN=C4 PE=1 SV=3

CO4B_MOUSE **Mass:** 194417 **Score:** 39 **Matches:** 1(0) **Sequences:** 1(0)
Complement C4-B OS=Mus musculus GN=C4b PE=1 SV=2

Peptide matches not assigned to protein hits: (no details means no match)

Query	Observed	Mr(expt)	Mr(calc)	Delta	Miss	Score	Expect	Rank	Unique	Peptide
168	537.3036	1072.5926	1072.5764	0.0162	0	46	0.026	1		ETLAALDNVK
167	537.3032	1072.5918	1072.5699	0.0219	1	25	3.2	1		IKMPDLNAR + Oxidation (M)
237	651.8362	1301.6578	1301.6099	0.0479	0	14	23	1		ANEAENEPSTLK
183	557.2814	1112.5482	1112.5172	0.0310	0	14	18	1		GMLDPPQDPK + Oxidation (M)
299	887.9588	1773.9031	1773.8857	0.0174	2	14	23	1		GRVEELGEEIGRESSK
123	846.4449	845.4376	845.4681	-0.0304	0	13	56	1		LMAVVEGK
124	846.4451	845.4379	845.4495	-0.0116	0	13	58	1		DTVVEVGK
331	737.7062	2210.0967	2210.1080	-0.0113	2	12	22	1		DAGRYSRQLLVDDFGVSGQK
408	698.3998	3486.9624	3486.9273	0.0352	1	12	1.8	1		LGIDGLSMGLILLTGFVTTLATLAAWPVTRNTR + Oxidation (M)
153	521.2798	1040.5451	1040.5866	-0.0415	0	12	34	1		PSVGIVIGDGK
141	497.7895	993.5645	993.5396	0.0249	0	10	48	1		LQWSPVHK
367	825.0729	2472.1970	2472.2141	-0.0170	2	9	34	1		ANYGYLLEMPVSRLTSDEMKR
117	416.7496	831.4846	831.4450	0.0396	0	9	1.2e+002	1		EASLASVR
354	762.0638	2283.1696	2283.1470	0.0226	0	9	48	1		LQAIEPSGNYHILHAYCGIK
39	538.3072	537.2999	537.2547	0.0452	0	8	1.1e+002	1		GGYNK
325	719.6873	2156.0401	2156.0597	-0.0196	0	8	50	1		APVSPALASPSSEVSDVSEATR
353	762.0602	2283.1587	2283.1470	0.0117	0	8	64	1		LQAIEPSGNYHILHAYCGIK
182	557.2814	1112.5482	1112.5576	-0.0094	0	7	82	1		EGMYLYLPK
184	557.2820	1112.5494	1112.5574	-0.0080	2	7	89	1		KDLSHRDDK
298	590.5961	1768.7664	1768.7926	-0.0262	2	7	23	1		KEGADRFCMGWAWR
38	538.3070	537.2997	537.2547	0.0450	0	7	1.6e+002	1		GGYNK
423	517.2649	4130.0608	4130.0630	-0.0023	0	6	32	1		VCISTYTTNLGSGPVSVSAVGVLAPHSALAALEDTADYPAR
309	943.0326	1884.0507	1884.0720	-0.0213	0	6	65	1		SLGWVVLLTASEEIIVR
324	719.6838	2156.0295	2156.0763	-0.0467	2	6	74	1		IFENSKPEESSFRARGFR
40	538.3075	537.3002	537.2547	0.0455	0	6	1.7e+002	1		GGYNK
409	601.3165	3601.8556	3601.8549	0.0007	2	6	40	1		APGFGDNRKAMLEDIAVLTAGTVISEEIGLELEK + Oxidation (M)
479	5297.7932	5296.7859	5296.7563	0.0296	2	6	6.7	1		FLIACGAIIKGHGTNVIHIQGVPSLNGCTYSIMPDRIEAGTFLTAAAITK
196	588.3222	1174.6299	1174.5870	0.0429	0	6	2.2e+002	1		DVDETIGWIK
392	630.3292	3146.6094	3146.5661	0.0433	0	6	67	1		DILVLSMGCGNAALQVAGLTSMEAVELAGEK
295	883.8993	1765.7841	1765.7723	0.0117	1	6	42	1		DAVQNCCGISKTEER
371	430.2392	2575.3912	2575.3614	0.0298	1	6	57	1		TGGMSGPAVKPIAVRMVYQVANAVK + 2 Oxidation (M)
427	698.3966	4184.3357	4184.2880	0.0477	2	5	4.8	1		FFLNFVLLVVLAGAIQLGYKHNVHGFIYKHAHDIYK
446	644.3310	4503.2659	4503.2540	0.0119	2	4	36	1		YDDPVIPGQNFALFSFLPTVGAQPDADGVYGFLKIRGTFDR
411	618.2729	3703.5935	3703.6269	-0.0334	0	4	6.3	1		DEGLYENSMIVFMGDHYGISEAHNEAMAEFLGK
125	846.4453	845.4380	845.4681	-0.0301	0	3	5.2e+002	1		AMLVDVAK
464	692.3534	4839.4231	4839.3959	0.0271	2	3	34	1		FSSALSVETFMKSTSIINFNQAALNKTSAAIMELANSEGLHSHSR
373	660.3610	2637.4151	2637.4299	-0.0148	2	3	1.1e+002	1		SIDISSLLLDKLSGESQTVVMKFK
383	412.1727	2878.1578	2878.1237	0.0340	0	3	2.7	1		MFSELINFQNEGHECQCQCGSCK + Oxidation (M)
417	807.4209	4032.0682	4032.0402	0.0281	1	3	71	1		ELILDSMEDVIVSLTDTYAPTRGVEGWDIAALQQGVK
445	747.3739	4478.1999	4478.2344	-0.0345	2	2	61	1		KRGGWCLQVNHLLYWALTMIGFETTILGGYVYNTFNDK
498	5857.7106	5856.7033	5856.7413	-0.0379	0	2	11	1		NDLLLEGFNNYTFLSNGFVPIPAAQDDEMFQETVEAMSIMGFSEEEQLSVLK
426	597.3054	4174.0869	4174.1031	-0.0162	1	2	71	1		FLEELLSPDPQMDFLALSQELEQEEGLTLAQLVEKR + Oxidation (M)
420	579.3024	4048.0657	4048.0300	0.0356	1	2	70	1		YVPFPLRYACEFLMQAFGLQLQMELQLASQLAEK + Oxidation (M)
461	679.3561	4748.4415	4748.4557	-0.0142	2	2	41	1		LVDLATCEIFTIRVHTVEILKVFLNNCPEYLSETIENSLR
458	593.8002	4742.3432	4742.3757	-0.0325	2	2	51	1		DAFVLASLGCNVTMIERSAVAAALLEDGLERAYLNLEIGTWMK + 2 Oxidation (M)
236	641.8614	1281.7081	1281.6718	0.0364	0	2	2.7e+002	1		GVFYGSGIGQLGK
349	1142.1003	2282.1861	2282.1616	0.0244	0	2	2.2e+002	1		QELYNILPSMLNHPDELIK + Oxidation (M)
512	6508.4906	6507.4833	6507.5187	-0.0354	2	2	4.3	1		SIQFHACFNQHMSCVRFLHRTILPGSIANSICQNIEIIILVTLTLLIFILLSVLSK + Oxidation (M)
355	764.3865	2290.1377	2290.1198	0.0179	1	2	2.3e+002	1		EAENMAQRGIGVAASAVPMNFK
407	683.3418	3411.6726	3411.6300	0.0426	2	2	1.2e+002	1		QGGQQLFQKRTDIVSPGGNAYGEEMTATCLR
476	5132.5269	5131.5196	5131.4998	0.0198	2	2	43	1		HKPYAMDRINEYSHIVIGNPEESAGNWKEIFGNEQPIHIEVGTGR
451	574.8019	4590.3571	4590.3906	-0.0335	2	2	47	1		IDILIVVNMFLTGFDSKVLNTLYVDKNLMYHDLIQAYSR + Oxidation (M)
494	5745.0377	5744.0304	5744.0264	0.0040	0	2	14	1		LANPAPLGLMGFGMTTILLNLHNVGYFALDGIILAMGIFYGGIAQIFAGLLEYK + 2 Oxidation (M)
368	416.2561	2491.4928	2491.4486	0.0442	2	2	9.6	1		LLDVAVGELTQIAGQKPQIRKSK
527	1004.9801	8031.7822	8031.7958	-0.0136	2	2	4.7	1		DPQLAAWIEENVTFPCTMVDRIVPAATPETLQEIADQLGVYDPCAIACEPFRQWVIEDNFVNGRPDWDK + Oxidation (M)
442	741.3508	4442.0613	4442.0379	0.0233	2	1	41	1		DKDEYVILSDILGDEDYLGDMDFKVAGTSEGVTALQMDMK + Oxidation (M)
471	631.8065	5046.3939	5046.4157	-0.0218	1	1	44	1		SVQAMLPGADVFPYTIRVVSEITESNGSSSMASVCGASLALMDAGVPMK + Oxidation (M)
65	615.4042	614.3970	614.4115	-0.0146	1	1	1.1e+003	1		AIKVGK
473	637.6797	5093.3796	5093.3887	-0.0091	2	0	32	1		SIANCKWTTLASVGKDIIEQADGIIMPHQWMEGNLPVSSMCAVCK + 3 Oxidation (M)
486	683.3393	5458.6560	5458.6207	0.0353	2	0	44	1		LEVGELAGVELDAMYFGFDVVCRDTLADGARLEIVQRPGQAFCLGCGER
439	4376.3546	4375.3473	4375.3362	0.0111	2	0	37	1		ELGVGFRMTSGPVLAKPGDLAAILTNLEPRDVLFIDEIHR + Oxidation (M)
444	639.3569	4468.4471	4468.4769	-0.0298	0	0	20	1		MDVITIIPPLVVGPFLIPTMPPSLITALSPITGNEAHYSIIK

504	6169.9418	6168.9345	6168.9324	0.0021	0	0	24	1	VSGILDFSDMSFGYYIFELAITIMYMMIESNDPLHAGGYVLAGFQSVIPLTDEEK + 2 Oxidation (M)
416	500.8060	3998.3896	3998.3442	0.0454	2	0	0.98	1	TNITIPQSQRDLITALKATGKPLVLVLMNGRPLALVK + Oxidation (M)
370	854.3721	2560.0946	2560.0889	0.0057	0	0	34	1	SPLMPGCELPVGTCPDMCPAAER + Oxidation (M)
387	970.7859	2909.3360	2909.3395	-0.0035	0	0	1.2e+002	1	EEPYLLYNEVEFGVPYATQGDSYAR
1	412.1689	411.1617							
2	415.2123	414.2050							
3	415.2127	414.2054							
4	415.2127	414.2054							
5	415.2552	414.2479							
6	415.3425	414.3352							
7	419.1679	418.1607							
8	430.2449	429.2376							
9	432.2390	431.2318							
10	432.2392	431.2319							
11	432.2393	431.2320							
12	432.2822	431.2749							
13	433.1282	432.1210							
14	433.1818	432.1745							
15	433.3529	432.3456							
16	444.1174	443.1101							
17	444.1175	443.1102							
18	444.1177	443.1104							
19	444.1205	443.1132							
20	447.1354	446.1281							
21	447.1383	446.1310							
22	456.2811	455.2738							
23	456.2812	455.2740							
24	459.2815	458.2743							
25	474.8332	473.8259							
26	476.3056	475.2983							
27	478.3142	477.3070							
28	500.3073	499.3001							
29	500.3074	499.3001							
30	502.3754	501.3682							
31	520.3342	519.3269							
32	520.3344	519.3271							
33	520.3350	519.3277							
34	520.3353	519.3280							
35	528.3646	527.3574							
36	528.3650	527.3577							
37	531.2688	530.2615							
41	539.3327	538.3255							
42	539.3369	538.3296							
43	546.4021	545.3948							
44	547.3341	546.3268							
45	554.5515	553.5442							
46	562.3088	561.3015							
47	564.3599	563.3527							
48	564.3615	563.3542							
49	564.3617	563.3545							
50	579.2120	578.2047							
51	579.5352	578.5279							
52	586.4176	585.4103							
53	590.4271	589.4199							
54	590.4288	589.4215							
55	591.3583	590.3510							
56	594.3705	593.3632							
57	594.3711	593.3638							
58	601.5411	600.5339							
59	601.5416	600.5343							
60	608.3870	607.3797							
61	608.3874	607.3801							
62	611.3552	610.3479							
63	614.5732	613.5659							
64	615.4041	614.3969							
66	615.4047	614.3974							
67	617.4073	616.4001							
68	617.4073	616.4001							
69	630.4440	629.4368							
70	630.4444	629.4372							
71	638.3916	637.3843							
72	638.4182	637.4109							
73	650.2065	649.1992							
74	652.4140	651.4067							
75	652.4154	651.4082							
76	661.5623	660.5550							
77	661.5627	660.5554							
78	661.5632	660.5559							
79	666.3931	665.3858							
80	666.3941	665.3868							
81	674.4662	673.4589							
82	675.6767	674.6695							
83	675.6770	674.6697							
84	675.6774	674.6701							
85	675.6778	674.6705							
86	675.6779	674.6706							
87	675.6780	674.6707							
88	675.6799	674.6727							
89	675.6802	674.6729							
90	677.6846	676.6773							
91	677.8223	676.8150							
92	678.5896	677.5824							
93	678.5900	677.5827							
94	682.4234	681.4161							
95	717.6250	716.6178							
96	717.6258	716.6185							
97	717.6259	716.6186							
98	718.4963	717.4890							
99	718.4974	717.4901							
100	734.6454	733.6381							
101	734.6528	733.6456							
102	739.5999	738.5926							
103	739.6053	738.5980							

104	739.6068	738.5995
105	739.6075	738.6002
106	739.6087	738.6014
107	752.5419	751.5347
108	752.5479	751.5406
109	752.5486	751.5413
110	762.5226	761.5154
114	412.7551	823.4956
115	412.7552	823.4958
116	416.2476	830.4806
118	832.4919	831.4846
126	428.1780	854.3414
127	430.2269	858.4393
128	433.3529	864.6913
130	448.2156	894.4167
131	456.2777	910.5408
136	950.6375	949.6302
	542.2604	1082.5063
	549.7443	1097.4741
	549.7486	1097.4827
	608.2579	1214.5012
	608.2582	1214.5018
	613.3616	1224.7086
	658.2201	1314.4255
	681.8027	1361.5909
	525.3237	1572.9494
	551.3401	1650.9986
	558.8081	1673.4026
	564.3643	1690.0710
	578.3398	1731.9977
	884.3992	1766.7839
	590.5961	1768.7664
	910.8590	1819.7035
	650.7616	1949.2629
	415.3397	2071.6622
	691.9724	2072.8955
	691.9725	2072.8957
	728.5932	2182.7576
	752.6867	2255.0384
	755.3482	2263.0228
	755.3483	2263.0229
	755.3484	2263.0233
351	1142.1006	2282.1866
	773.3446	2317.0121
	779.3743	2335.1011
	779.3754	2335.1043
	784.7070	2351.0993
	608.3855	2429.5127
	608.3895	2429.5287
	822.3619	2464.0638
	432.2824	2587.6509
	544.3342	2716.6344
	456.2801	2731.6371
	458.3478	2744.0431
	476.3093	2851.8121
	413.2551	2885.7350
	578.3357	2886.6422
	969.4516	2905.3329
	734.6517	2934.5777
	500.2993	2995.7521
	504.3052	3019.7878
393	1054.5083	3160.5031
	528.3678	3164.1630
	636.8362	3179.1445
	402.3541	3210.7747
399	1103.9197	3308.7372
	415.1274	3312.9613
	666.3880	3326.9034
	841.1712	3360.6557
	613.3409	3674.0017
	552.3067	3859.0958
	650.2040	3895.1806
	651.2096	3901.2142
	665.3697	3986.1744
	506.3167	4042.4752
	4044.3511	4043.3438
	678.3128	4063.8331
	4124.3489	4123.3416
	696.4179	4172.4636
	696.4353	4172.5680
	598.8006	4184.5532
	1409.2669	4224.7788
	1409.2673	4224.7800
	605.8052	4233.5853
	1070.4315	4277.6968
	717.6255	4299.7092
	539.3429	4306.6853
	4312.7625	4311.7552
	622.3542	4349.4283
	622.3674	4349.5206
	546.9452	4367.5037
	734.6513	4401.8639
	632.3478	4419.3836
443	4446.4040	4445.3967
447	4520.6009	4519.5936
448	4522.7020	4521.6947
	652.4133	4559.8419
	764.4429	4580.6136
	923.5301	4612.6141
	771.4147	4622.4446
	4634.4112	4633.4039
	4673.7157	4672.7084

456	671.8108	4695.6250
457	789.4759	4730.8119
459	678.5896	4743.0761
460	594.3694	4746.8973
462	681.7781	4765.3956
463	4824.2328	4823.2255
465	695.8495	4863.8952
466	976.4512	4877.2196
467	613.3815	4898.9939
468	5013.6637	5012.6564
469	5015.5015	5014.4942
470	717.8990	5018.2418
472	5065.0211	5064.0138
474	1023.4850	5112.3887
475	854.3732	5120.1952
477	5142.9521	5141.9448
478	5227.9137	5226.9064
480	5335.3510	5334.3437
481	671.3548	5362.7804
482	897.4302	5378.5377
483	674.4617	5387.6355
484	898.9952	5387.9276
485	5456.0335	5455.0262
487	5466.1893	5465.1820
488	696.4351	5563.4224
489	5578.0010	5576.9937
490	5606.3965	5605.3892
491	807.4183	5644.8774
492	941.9191	5645.4708
493	5677.1175	5676.1102
495	5758.5372	5757.5299
496	722.3279	5770.5648
497	5847.9046	5846.8973
499	740.4741	5915.7349
500	5997.6721	5996.6648
501	858.9308	6005.4646
502	6073.9006	6072.8933
503	6105.4958	6104.4885
505	773.3506	6178.7464
506	6225.3263	6224.3190
507	6258.9039	6257.8966
508	6451.9464	6450.9391
509	6460.8104	6459.8031
510	6482.7501	6481.7428
511	6491.9985	6490.9912
513	6569.1780	6568.1707
514	6572.0064	6570.9991
515	6624.4522	6623.4449
516	6700.0001	6698.9928
517	6734.5706	6733.5633
518	6763.4675	6762.4602
519	6836.1292	6835.1219
520	858.9380	6863.4459
521	6903.2409	6902.2336
522	6948.6049	6947.5976
523	6994.2515	6993.2442
524	887.9511	7095.5503
525	950.6145	7596.8581
526	7628.7256	7627.7183
528	8080.1805	8079.1732
529	8134.2703	8133.2630
530	8180.7440	8179.7367
531	8210.0502	8209.0429
532	8724.9338	8723.9265

Search Parameters

Type of search : MS/MS Ion Search
Enzyme : Trypsin
Fixed modifications : Carbamidomethyl (C)
Variable modifications : Oxidation (M)
Mass values : Monoisotopic
Protein Mass : Unrestricted
Peptide Mass Tolerance : ± 0.05 Da
Fragment Mass Tolerance: ± 0.05 Da
Max Missed Cleavages : 2
Instrument type : Default
Number of queries : 532

Mascot Search Results

```
User         : Julian
Email        : julian.langer@mpibp-frankfurt.mpg.de
Search title : Submitted from 140318 842  ChKrettler 1-10  swissprot full T 0.05Da by Mascot Daemon on CCSW010
MS data file : D:\Data\1403\140318\140314 842 ChReinhard   9_Tray01-D5_01_17690.d\140314 842 ChReinhard 9_Tray01-D5_01_17690.mgf
Database     : SwissProt 57.15 (515203 sequences; 181334896 residues)
Timestamp    : 19 Mar 2014  at 09:52:53 GMT
Protein hits : F13A_HUMAN  Coagulation factor XIII A chain   OS=Homo sapiens GN=F13A1 PE=1 SV=4
               F13B_HUMAN  Coagulation factor XIII B chain   OS=Homo sapiens GN=F13B PE=1 SV=3
               TRYP_PIG    Trypsin OS=Sus scrofa PE=1 SV=1
               K2C1_HUMAN  Keratin, type  II cytoskeletal  1 OS=Homo sapiens GN=KRT1 PE=1 SV=6
               K1C9_HUMAN  Keratin, type  I cytoskeletal 9 OS=Homo sapiens GN=KRT9 PE=1 SV=3
               K22E_HUMAN  Keratin, type  II cytoskeletal  2 epidermal OS=Homo sapiens GN=KRT2 PE=1 SV=2
               K1C10_HUMAN Keratin, type  I cytoskeletal 10  OS=Homo sapiens GN=KRT10 PE=1 SV=6
```

Mascot Score Histogram

Ions score is -10*Log(P), where P is the probability that the observed match is a random event.
Individual ions scores > 37 indicate identity or extensive homology (p<0.05).
Protein scores are derived from ions scores as a non-probabilistic basis for ranking protein hits.

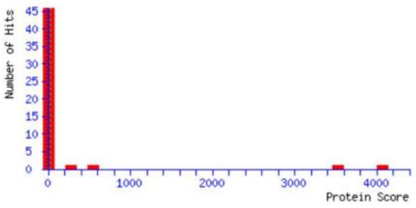

Peptide Summary Report

Format As: Peptide Summary | Help

Significance threshold p<0.05 | Max. number of hits AUTO

Standard scoring | MudPIT scoring | Ions score or expect cut-off 0 | Show sub-sets 0

Show pop-ups | Suppress pop-ups | Sort unassigned Decreasing Score | Require bold red

Select All | Select None | Search Selected | **Error tolerant** | Archive Report

1. F13A_HUMAN **Mass:** 83728 **Score:** 4067 **Matches:** 114(92) **Sequences:** 39(35) **emPAI:** 8.97
Coagulation factor XIII A chain OS=Homo sapiens GN=F13A1 PE=1 SV=4
Check to include this hit in error tolerant search or archive report

Query	Observed	Mr(expt)	Mr(calc)	Delta	Miss	Score	Expect	Rank	Unique	Peptide
124	430.2502	858.4858	858.4811	0.0047	0	(61)	0.00097	1	U	R.LSIQSSPK.C
125	430.2505	858.4865	858.4811	0.0054	0	83	6.1e-006	1	U	R.LSIQSSPK.C
130	435.7630	869.5114	869.5083	0.0031	1	47	0.012	1		R.GNPIKVSR.V
131	435.7636	869.5127	869.5083	0.0044	1	(40)	0.056	1		R.GNPIKVSR.V
132	438.7358	875.4570	875.4535	0.0036	0	(70)	0.00011	1		R.VGSAMVNAK.D
133	438.7358	875.4571	875.4535	0.0037	0	70	0.00011	1		R.VGSAMVNAK.D
134	438.7363	875.4581	875.4535	0.0046	0	(69)	0.00012	1		R.VGSAMVNAK.D
135	439.2154	876.4163	876.4123	0.0040	0	(37)	0.13	1	U	R.AQMDLSGR.G
136	439.2155	876.4164	876.4123	0.0040	0	40	0.069	1	U	R.AQMDLSGR.G
137	439.2156	876.4167	876.4123	0.0043	0	(37)	0.13	1	U	R.AQMDLSGR.G
157	515.7733	1029.5321	1029.5277	0.0044	0	72	6e-005	1	U	R.CGPASVQAIK.H
158	515.7736	1029.5326	1029.5277	0.0049	0	(69)	0.00011	1	U	R.CGPASVQAIK.H
172	558.8105	1115.6065	1115.6009	0.0056	0	(67)	0.00018	1	U	K.KPLNTEGVMK.S
178	566.8073	1131.6001	1131.5958	0.0043	0	(69)	0.00012	1	U	K.KPLNTEGVMK.S + Oxidation (M)
179	566.8078	1131.6009	1131.5958	0.0052	0	74	3.5e-005	1	U	K.KPLNTEGVMK.S + Oxidation (M)
180	566.8078	1131.6011	1131.5958	0.0053	0	(68)	0.00012	1	U	K.KPLNTEGVMK.S + Oxidation (M)
183	576.2540	1150.4934	1150.4891	0.0044	0	(43)	0.0079	1		K.FQEGQEEER.L
184	576.2543	1150.4941	1150.4891	0.0050	0	46	0.0039	1		K.FQEGQEEER.L
190	590.3072	1178.5999	1178.5965	0.0035	0	(72)	4.3e-005	1	U	K.LIASMSSDSLR.H
191	590.3073	1178.6001	1178.5965	0.0036	0	83	3.4e-006	1	U	K.LIASMSSDSLR.H
194	598.3056	1194.5965	1194.5914	0.0051	0	(75)	1.9e-005	1	U	K.LIASMSSDSLR.H + Oxidation (M)
195	598.3056	1194.5966	1194.5914	0.0052	0	(72)	3.2e-005	1	U	K.LIASMSSDSLR.H + Oxidation (M)
223	648.8492	1295.6838	1295.6795	0.0043	0	84	2.3e-006	1		R.LALETALMYGAK.K + Oxidation (M)
224	648.8494	1295.6843	1295.6795	0.0048	0	(82)	3.2e-006	1		R.LALETALMYGAK.K + Oxidation (M)
225	648.8496	1295.6846	1295.6795	0.0051	0	(84)	2.3e-006	1		R.LALETALMYGAK.K + Oxidation (M)
228	654.3554	1306.6962	1306.6915	0.0048	1	61	0.00049	1	U	R.KLIASMSSDSLR.H
239	441.9051	1322.6936	1322.6864	0.0072	1	(57)	0.0012	1	U	R.KLIASMSSDSLR.H + Oxidation (M)
240	663.9187	1325.8228	1325.8170	0.0058	0	(43)	0.0077	1	U	K.STVLTIPEIIIK.V
241	663.9191	1325.8237	1325.8170	0.0068	0	43	0.0072	1	U	K.STVLTIPEIIIK.V
255	683.8797	1365.7449	1365.7405	0.0044	1	70	5.1e-005	1		R.DLFRVEYVIGR.Y
256	456.2556	1365.7449	1365.7405	0.0044	1	(45)	0.016	1		R.DLFRVEYVIGR.Y
289	486.2583	1455.7530	1455.7470	0.0060	0	(68)	8.7e-005	1	U	R.HVYGELDVQIQR.R
290	728.8838	1455.7530	1455.7470	0.0060	0	(79)	6.7e-006	1	U	R.HVYGELDVQIQR.R
291	486.2586	1455.7539	1455.7470	0.0069	0	(92)	3.3e-007	1	U	R.HVYGELDVQIQR.R
292	728.8842	1455.7539	1455.7470	0.0069	0	95	1.6e-007	1	U	R.HVYGELDVQIQR.R
305	763.3966	1524.7786	1524.7712	0.0074	0	82	3.2e-006	1	U	K.ETFDVTLEPLSFK.K
306	777.9139	1553.8133	1553.8065	0.0069	0	(34)	0.2	1	U	R.MYVAVWTPYGVLR.T
308	785.9111	1569.8077	1569.8014	0.0064	0	(40)	0.051	1	U	R.MYVAVWTPYGVLR.T + Oxidation (M)
309	785.9114	1569.8082	1569.8014	0.0069	0	62	0.00034	1	U	R.MYVAVWTPYGVLR.T + Oxidation (M)
320	797.9184	1593.8221	1593.8151	0.0071	1	69	7.4e-005	1		R.VEYVIGRYPQENK.G
321	532.2813	1593.8222	1593.8151	0.0071	1	(45)	0.016	1		R.VEYVIGRYPQENK.G
322	797.9187	1593.8228	1593.8151	0.0077	1	(62)	0.00034	1		R.VEYVIGRYPQENK.G
323	532.2816	1593.8228	1593.8151	0.0077	1	(35)	0.18	1		R.VEYVIGRYPQENK.G
329	822.8692	1643.7238	1643.7171	0.0067	0	(101)	1e-008	1	U	K.QIGGDGMMDITDTYK.F
330	822.8697	1643.7249	1643.7171	0.0078	0	130	1.4e-011	1	U	K.QIGGDGMMDITDTYK.F
345	830.8662	1659.7179	1659.7120	0.0059	0	(86)	2.3e-007	1	U	K.QIGGDGMMDITDTYK.F + Oxidation (M)
346	830.8666	1659.7186	1659.7120	0.0066	0	(101)	7.5e-009	1	U	K.QIGGDGMMDITDTYK.F + Oxidation (M)
359	844.4707	1686.9268	1686.9192	0.0076	0	(64)	0.00018	1	U	K.GTYIPVPIVSELQSGK.W
360	844.4710	1686.9274	1686.9192	0.0082	0	(69)	6.3e-005	1	U	K.GTYIPVPIVSELQSGK.W
361	844.4710	1686.9274	1686.9192	0.0082	0	69	6.2e-005	1	U	K.GTYIPVPIVSELQSGK.W
362	564.6190	1690.8351	1690.8275	0.0077	0	(86)	1.4e-006	1		K.DGTHVVENVDATHIGK.L
363	564.6195	1690.8367	1690.8275	0.0092	0	88	7.5e-007	1		K.DGTHVVENVDATHIGK.L
394	883.9197	1765.8248	1765.8193	0.0056	0	(69)	4e-005	1	U	R.SNVDMDFEVENAVLGK.D
395	883.9199	1765.8253	1765.8193	0.0061	0	(83)	1.7e-006	1	U	R.SNVDMDFEVENAVLGK.D
399	594.6645	1780.9717	1780.9611	0.0106	2	51	0.003	1	U	K.KETFDVTLEPLSFKK.E
401	891.9178	1781.8210	1781.8142	0.0069	0	(97)	5.8e-008	1	U	R.SNVDMDFEVENAVLGK.D + Oxidation (M)
402	891.9185	1781.8224	1781.8142	0.0082	0	121	2.3e-010	1	U	R.SNVDMDFEVENAVLGK.D + Oxidation (M)
408	602.6567	1804.9484	1804.9407	0.0077	0	44	0.023	1	U	R.NVWVHLDGPGVTRPMK.K
409	602.6571	1804.9495	1804.9407	0.0089	0	(43)	0.027	1	U	R.NVWVHLDGPGVTRPMK.K
412	607.3176	1818.9310	1818.9224	0.0085	1	62	0.00031	1		K.KDGTHVVENVDATHIGK.L
413	607.3180	1818.9321	1818.9224	0.0096	1	(54)	0.0022	1		K.KDGTHVVENVDATHIGK.L
414	455.7403	1818.9321	1818.9224	0.0097	1	(59)	0.00063	1		K.KDGTHVVENVDATHIGK.L
417	607.9882	1820.9427	1820.9356	0.0071	0	(21)	3.9	1	U	R.NVWVHLDGPGVTRPMK.K + Oxidation (M)
418	607.9889	1820.9449	1820.9356	0.0093	0	(24)	1.9	1	U	R.NVWVHLDGPGVTRPMK.K + Oxidation (M)
438	982.9804	1963.9462	1963.9404	0.0058	0	68	5.8e-005	1		R.YGQCWVFAGVFNTFLR.C

440	496.0145	1980.0289	1980.0177	0.0112	2	39	0.051	1	U	K.VDHHTDKYENNKLIVR.R
441	496.0152	1980.0319	1980.0177	0.0142	2	(37)	0.086	1	U	K.VDHHTDKYENNKLIVR.R
476	536.7529	2142.9826	2142.9719	0.0107	2	(32)	0.12	1	U	R.WDTNKVDHHTDKYENNK.L
477	715.3348	2142.9826	2142.9719	0.0107	2	36	0.046	1	U	R.WDTNKVDHHTDKYENNK.L
480	725.0122	2172.0149	2172.0045	0.0104	1	73	1.1e-005	1	U	R.SNVDMDFEVENAVLGKDFK.L + Oxidation (M)
500	777.0615	2328.1626	2328.1525	0.0101	0	(69)	4.3e-005	1	U	R.EEYVLNDIGVIFYGEVNDIK.T
501	1165.0886	2328.1626	2328.1525	0.0101	0	174	1.3e-015	1	U	R.EEYVLNDIGVIFYGEVNDIK.T
502	1165.0891	2328.1636	2328.1525	0.0111	0	(162)	2.6e-014	1	U	R.EEYVLNDIGVIFYGEVNDIK.T
503	777.0618	2328.1637	2328.1525	0.0111	0	(57)	0.00067	1	U	R.EEYVLNDIGVIFYGEVNDIK.T
514	805.0648	2412.1725	2412.1631	0.0094	1	(63)	0.00016	1		K.FQEGQEEERLALETALMYGAK.K
515	805.0649	2412.1730	2412.1631	0.0099	1	(46)	0.0073	1		K.FQEGQEEERLALETALMYGAK.K
516	805.0650	2412.1731	2412.1631	0.0101	1	(59)	0.00043	1		K.FQEGQEEERLALETALMYGAK.K
521	810.3948	2428.1626	2428.1580	0.0046	1	66	6.9e-005	1		K.FQEGQEEERLALETALMYGAK.K + Oxidation (M)
522	810.3965	2428.1676	2428.1580	0.0096	1	(61)	0.00021	1		K.FQEGQEEERLALETALMYGAK.K + Oxidation (M)
523	810.3972	2428.1697	2428.1580	0.0117	1	(46)	0.0074	1		K.FQEGQEEERLALETALMYGAK.K + Oxidation (M)
528	1278.0696	2554.1247	2554.1145	0.0103	0	(62)	4.1e-005	1	U	R.SWSYGQFEDGILDTCLYVMDR.A
529	1278.0697	2554.1249	2554.1145	0.0104	0	70	6.5e-006	1	U	R.SWSYGQFEDGILDTCLYVMDR.A
530	1286.0652	2570.1159	2570.1094	0.0065	0	(34)	0.021	1	U	R.SWSYGQFEDGILDTCLYVMDR.A + Oxidation (M)
531	1286.0659	2570.1172	2570.1094	0.0078	0	(13)	2.5	1	U	R.SWSYGQFEDGILDTCLYVMDR.A + Oxidation (M)
532	1286.0673	2570.1200	2570.1094	0.0106	0	(34)	0.024	1	U	R.SWSYGQFEDGILDTCLYVMDR.A + Oxidation (M)
539	1301.6637	2601.3128	2601.3035	0.0094	0	(25)	1	1	U	R.AVPPNNSNAAEDDLPTVELQGVVPR.G
540	868.1116	2601.3129	2601.3035	0.0094	0	72	2.4e-005	1	U	R.AVPPNNSNAAEDDLPTVELQGVVPR.G
541	868.1121	2601.3145	2601.3035	0.0110	0	(34)	0.15	1	U	R.AVPPNNSNAAEDDLPTVELQGVVPR.G
542	1301.6648	2601.3151	2601.3035	0.0117	0	(25)	1	1	U	R.AVPPNNSNAAEDDLPTVELQGVVPR.G
543	868.1149	2601.3228	2601.3035	0.0193	0	(68)	5.5e-005	1	U	R.AVPPNNSNAAEDDLPTVELQGVVPR.G
544	1301.6687	2601.3228	2601.3035	0.0193	0	(25)	1.1	1	U	R.AVPPNNSNAAEDDLPTVELQGVVPR.G
548	872.1086	2613.3040	2613.2962	0.0078	1	(63)	0.00015	1	U	K.EREEYVLNDIGVIFYGEVNDIK.T
550	872.1100	2613.3081	2613.2962	0.0118	1	(92)	1.9e-007	1	U	K.EREEYVLNDIGVIFYGEVNDIK.T
551	872.1102	2613.3087	2613.2962	0.0125	1	94	1.3e-007	1	U	K.EREEYVLNDIGVIFYGEVNDIK.T
585	681.3403	2721.3319	2721.3194	0.0125	0	28	0.44	1	U	R.EIRPNSTVQWEEVCRPWVSGHR.K
586	681.3408	2721.3343	2721.3194	0.0149	0	(27)	0.55	1	U	R.EIRPNSTVQWEEVCRPWVSGHR.K
593	920.1462	2757.4169	2757.4046	0.0123	1	79	3.4e-006	1	U	R.RAVPPNNSNAAEDDLPTVELQGVVPR.G
594	920.1467	2757.4183	2757.4046	0.0137	1	(55)	0.00088	1	U	R.RAVPPNNSNAAEDDLPTVELQGVVPR.G
595	920.1467	2757.4183	2757.4046	0.0137	1	(51)	0.0022	1	U	R.RAVPPNNSNAAEDDLPTVELQGVVPR.G
596	920.1474	2757.4203	2757.4046	0.0158	1	(71)	2e-005	1	U	R.RAVPPNNSNAAEDDLPTVELQGVVPR.G
612	1007.8625	3020.5656	3020.5430	0.0227	0	(15)	7.4	1	U	K.EAVLIQAGEYMGQLLEQASLHFFVTAR.I
614	1013.1904	3036.5495	3036.5379	0.0116	0	36	0.068	1	U	K.EAVLIQAGEYMGQLLEQASLHFFVTAR.I + Oxidation (M)
615	1013.1921	3036.5543	3036.5379	0.0164	0	(26)	0.7	1	U	K.EAVLIQAGEYMGQLLEQASLHFFVTAR.I + Oxidation (M)
617	1021.1233	3060.3481	3060.3335	0.0146	0	75	1.4e-006	1	U	R.NPETDTYILFNPWCEDDAVYLDNEK.E
618	1531.1813	3060.3481	3060.3335	0.0146	0	(36)	0.0095	1	U	R.NPETDTYILFNPWCEDDAVYLDNEK.E
619	1531.1815	3060.3484	3060.3335	0.0150	0	(12)	2.9	1	U	R.NPETDTYILFNPWCEDDAVYLDNEK.E
638	1116.1723	3345.4950	3345.4772	0.0179	1	(44)	0.0019	1	U	R.NPETDTYILFNPWCEDDAVYLDNEKER.E
639	1116.1726	3345.4959	3345.4772	0.0188	1	65	1.7e-005	1	U	R.NPETDTYILFNPWCEDDAVYLDNEKER.E
647	1138.5304	3412.5694	3412.5517	0.0176	0	(128)	1.3e-011	1	U	R.IVTNYFSAHDNDANLQMDIFLEEDGNVNSK.L
648	1138.5309	3412.5708	3412.5517	0.0190	0	(119)	1.1e-010	1	U	R.IVTNYFSAHDNDANLQMDIFLEEDGNVNSK.L
649	1138.5312	3412.5716	3412.5517	0.0199	0	131	7.2e-012	1	U	R.IVTNYFSAHDNDANLQMDIFLEEDGNVNSK.L
651	1143.8613	3428.5621	3428.5467	0.0154	0	(88)	1e-007	1	U	R.IVTNYFSAHDNDANLQMDIFLEEDGNVNSK.L + Oxidation (M)
652	1143.8624	3428.5654	3428.5467	0.0187	0	(95)	2.2e-008	1	U	R.IVTNYFSAHDNDANLQMDIFLEEDGNVNSK.L + Oxidation (M)
669	1252.9454	3755.8144	3755.7995	0.0149	0	30	0.13	1	U	K.DDEGVLVGSWDNIYAYGVPPSAWTGSVDILLEYR.S

2. F13B_HUMAN **Mass:** 77742 **Score:** 3442 **Matches:** 103(87) **Sequences:** 36(31) **emPAI:** 6.70

Coagulation factor XIII B chain OS=Homo sapiens GN=F13B PE=1 SV=3

Check to include this hit in error tolerant search or archive report

Query	Observed	Mr(expt)	Mr(calc)	Delta	Miss	Score	Expect	Rank	Unique	Peptide
105	757.3910	756.3837	756.3806	0.0031	0	(16)	10	2	U	K.WTEPPK.C
106	757.3913	756.3840	756.3806	0.0034	0	27	0.8	1	U	K.WTEPPK.C
107	757.3918	756.3845	756.3806	0.0039	0	(26)	0.81	1	U	K.WTEPPK.C
109	405.2036	808.3927	808.3902	0.0026	0	(44)	0.028	1	U	K.YPLCTR.K
110	405.2037	808.3928	808.3902	0.0027	0	47	0.014	1	U	K.YPLCTR.K
127	431.2523	860.4900	860.4868	0.0032	1	50	0.0087	1	U	R.GQLKYPR.C
128	431.2525	860.4904	860.4868	0.0036	1	(39)	0.11	1	U	R.GQLKYPR.C
129	431.2528	860.4911	860.4868	0.0043	1	(38)	0.15	1	U	R.GQLKYPR.C
169	552.2843	1102.5540	1102.5515	0.0025	0	(36)	0.21	1	U	K.GMCTSPPLIK.H
170	552.2850	1102.5555	1102.5515	0.0041	0	(34)	0.27	1	U	K.GMCTSPPLIK.H
171	552.2852	1102.5558	1102.5515	0.0043	0	(37)	0.16	1	U	K.GMCTSPPLIK.H
175	560.2820	1118.5494	1118.5464	0.0030	0	52	0.0045	1	U	K.GMCTSPPLIK.H + Oxidation (M)
176	565.7704	1129.5263	1129.5226	0.0037	0	59	0.00048	1	U	R.CNEYYLLR.G
177	565.7708	1129.5270	1129.5226	0.0044	0	(56)	0.00087	1	U	R.CNEYYLLR.G
196	598.8052	1195.5959	1195.5913	0.0046	0	65	0.00016	1	U	R.IAQYYYTFK.S
197	598.8057	1195.5967	1195.5913	0.0054	0	(65)	0.00017	1	U	R.IAQYYYTFK.S
204	610.3318	1218.6491	1218.6431	0.0060	0	63	0.00039	1	U	R.CPPPPLPINSK.I
205	610.3320	1218.6493	1218.6431	0.0063	0	(58)	0.0012	1	U	R.CPPPPLPINSK.I
206	611.8192	1221.6238	1221.6176	0.0062	1	(46)	0.017	1	U	R.GEVKYPLCTR.K
207	408.2152	1221.6238	1221.6176	0.0062	1	54	0.0028	1	U	R.GEVKYPLCTR.K
208	611.8193	1221.6241	1221.6176	0.0065	1	(41)	0.047	1	U	R.GEVKYPLCTR.K
209	408.2153	1221.6241	1221.6176	0.0065	1	(51)	0.0053	1	U	R.GEVKYPLCTR.K
211	617.7957	1233.5769	1233.5740	0.0029	0	(37)	0.084	1	U	K.SFYFPMSIDK.K
212	617.7961	1233.5777	1233.5740	0.0037	0	48	0.006	1	U	K.SFYFPMSIDK.K
215	625.7940	1249.5735	1249.5689	0.0046	0	(45)	0.0098	1	U	K.SFYFPMSIDK.K + Oxidation (M)
216	625.7942	1249.5738	1249.5689	0.0049	0	(39)	0.043	1	U	K.SFYFPMSIDK.K + Oxidation (M)
229	439.5736	1315.6990	1315.6925	0.0065	0	(63)	0.00037	1	U	R.LIENGYFHPVK.Q
230	658.8568	1315.6990	1315.6925	0.0065	0	(65)	0.00026	1	U	R.LIENGYFHPVK.Q
231	658.8569	1315.6992	1315.6925	0.0067	0	77	1.4e-005	1	U	R.LIENGYFHPVK.Q
232	439.5737	1315.6992	1315.6925	0.0067	0	(62)	0.00045	1	U	R.LIENGYFHPVK.Q
238	661.3440	1320.6734	1320.6674	0.0060	0	61	0.00056	1	U	R.QSTLSYQEPLR.T
243	673.8092	1345.6039	1345.5972	0.0066	1	(61)	0.00015	1	U	R.CEDGKWTEPPK.C
244	449.5419	1345.6039	1345.5972	0.0066	1	(30)	0.23	1	U	R.CEDGKWTEPPK.C
245	673.8097	1345.6048	1345.5972	0.0076	1	67	4e-005	1	U	R.CEDGKWTEPPK.C
246	449.5422	1345.6048	1345.5972	0.0076	1	(48)	0.0031	1	U	R.CEDGKWTEPPK.C
251	454.8987	1361.6742	1361.6689	0.0053	1	(52)	0.0035	1	U	K.SFYFPMSIDKK.L
252	681.8444	1361.6742	1361.6689	0.0053	1	73	3.3e-005	1	U	K.SFYFPMSIDKK.L
253	681.8448	1361.6750	1361.6689	0.0061	1	(44)	0.023	1	U	K.SFYFPMSIDKK.L
260	689.8401	1377.6656	1377.6639	0.0017	1	(42)	0.032	1	U	K.SFYFPMSIDKK.L + Oxidation (M)
261	689.8418	1377.6691	1377.6639	0.0052	1	(43)	0.029	1	U	K.SFYFPMSIDKK.L + Oxidation (M)
271	468.8771	1403.6094	1403.6041	0.0054	0	(62)	8.1e-005	1	U	R.CFDHHFLEGSR.E
272	702.8120	1403.6094	1403.6041	0.0054	0	(58)	0.00019	1	U	R.CFDHHFLEGSR.E
273	702.8131	1403.6117	1403.6041	0.0076	0	68	2.2e-005	1	U	R.CFDHHFLEGSR.E
274	468.8778	1403.6117	1403.6041	0.0076	0	(50)	0.0013	1	U	R.CFDHHFLEGSR.E
278	472.5851	1414.7334	1414.7279	0.0055	0	(50)	0.0065	1	U	K.VLHGDLIDFVCK.Q
279	708.3740	1414.7334	1414.7279	0.0055	0	(89)	9e-007	1	U	K.VLHGDLIDFVCK.Q
280	708.3740	1414.7334	1414.7279	0.0055	0	101	5.4e-008	1	U	K.VLHGDLIDFVCK.Q
281	472.5851	1414.7334	1414.7279	0.0055	0	(59)	0.00078	1	U	K.VLHGDLIDFVCK.Q
283	711.8674	1421.7202	1421.7150	0.0051	1	(56)	0.0016	1	U	R.QSTLSYQEPLRT.-
284	711.8678	1421.7210	1421.7150	0.0060	1	60	0.00061	1	U	R.QSTLSYQEPLRT.-
298	745.4046	1488.7946	1488.7871	0.0076	1	(63)	0.00029	1	U	R.NRCPPPPLPINSK.I
299	497.2722	1488.7947	1488.7871	0.0076	1	(49)	0.0085	1	U	R.NRCPPPPLPINSK.I
300	745.4049	1488.7953	1488.7871	0.0082	1	65	0.00019	1	U	R.NRCPPPPLPINSK.I
315	532.2595	1593.7568	1593.7497	0.0071	1	(88)	6.3e-007	1	U	K.IYYNGDKVTYACK.S
316	797.8857	1593.7568	1593.7497	0.0071	1	(109)	5.1e-009	1	U	K.IYYNGDKVTYACK.S
317	797.8858	1593.7570	1593.7497	0.0073	1	115	1.2e-009	1	U	K.IYYNGDKVTYACK.S
318	532.2596	1593.7570	1593.7497	0.0073	1	(73)	2.3e-005	1	U	K.IYYNGDKVTYACK.S
319	532.2597	1593.7574	1593.7497	0.0077	1	(62)	0.00026	1	U	K.IYYNGDKVTYACK.S
347	834.3760	1666.7374	1666.7297	0.0077	0	77	2.9e-006	1	U	K.VQYECATGYYTAGGK.K
364	566.2772	1695.8098	1695.8138	-0.0040	0	(45)	0.012	1	U	K.CTKPDLSNGYISDVK.L
365	848.9122	1695.8098	1695.8138	-0.0040	0	(57)	0.00084	1	U	K.CTKPDLSNGYISDVK.L
366	566.2806	1695.8199	1695.8138	0.0062	0	(75)	1.3e-005	1	U	K.CTKPDLSNGYISDVK.L
367	566.2815	1695.8227	1695.8138	0.0089	0	89	5e-007	1	U	K.CTKPDLSNGYISDVK.L
368	848.9186	1695.8227	1695.8138	0.0089	0	(76)	1.2e-005	1	U	K.CTKPDLSNGYISDVK.L
373	854.9082	1707.8018	1707.7927	0.0092	0	94	1.2e-007	1	U	K.LSFFCLAGYTTESGR.Q
374	854.9089	1707.8031	1707.7927	0.0105	0	(70)	3.5e-005	1	U	K.LSFFCLAGYTTESGR.Q
396	884.9640	1767.9135	1767.9043	0.0092	0	97	1.1e-007	1	U	R.GDTYPAELYITGSILR.M
397	884.9643	1767.9141	1767.9043	0.0098	0	(91)	3.9e-007	1	U	R.GDTYPAELYITGSILR.M

404	898.4231	1794.8317	1794.8247	0.0070	1	4	97	1	U	K.VQYECATGYYTAGGKK.T
48	608.9801	1823.9185	1823.9087	0.0097	1	69	6.1e-005	1	U	K.KCTKPDLSNGYISDVK.L
420	608.9810	1823.9210	1823.9087	0.0123	1	(57)	0.00096	1	U	K.KCTKPDLSNGYISDVK.L
429	637.6277	1909.8612	1909.8516	0.0096	1	96	4.3e-008	1	U	K.DKVQYECATGYYTAGGK.K
430	955.9385	1909.8625	1909.8516	0.0109	1	(68)	2.8e-005	1	U	K.DKVQYECATGYYTAGGK.K
431	637.6281	1909.8626	1909.8516	0.0109	1	(80)	1.8e-006	1	U	K.DKVQYECATGYYTAGGK.K
432	956.9569	1911.8992	1911.8924	0.0067	0	110	2.9e-009	1	U	K.TEEVECLTYGWSLTPK.C
433	956.9573	1911.9001	1911.8924	0.0077	0	(106)	8e-009	1	U	K.TEEVECLTYGWSLTPK.C
445	681.0064	2039.9975	2039.9874	0.0101	1	(61)	0.00032	1	U	K.KTEEVECLTYGWSLTPK.C
446	1021.0060	2039.9975	2039.9874	0.0101	1	(11)	28	1	U	K.KTEEVECLTYGWSLTPK.C
447	681.0065	2039.9978	2039.9874	0.0104	1	(58)	0.00066	1	U	K.KTEEVECLTYGWSLTPK.C
448	681.0073	2040.0000	2039.9874	0.0126	1	(63)	0.0002	1	U	K.KTEEVECLTYGWSLTPK.C
449	1021.0073	2040.0000	2039.9874	0.0126	1	68	5.7e-005	1	U	K.KTEEVECLTYGWSLTPK.C
450	692.3186	2073.9341	2073.9248	0.0093	1	67	2.7e-005	1	U	R.GKWTLPPECVENNENCK.H
451	692.3191	2073.9354	2073.9248	0.0106	1	(51)	0.001	1	U	R.GKWTLPPECVENNENCK.H
452	692.3192	2073.9357	2073.9248	0.0109	1	(42)	0.008	1	U	R.GKWTLPPECVENNENCK.H
457	695.0052	2081.9938	2081.9840	0.0098	0	60	0.00031	1	U	K.VACEEPPFIENGAANLHSK.I
458	695.0054	2081.9944	2081.9840	0.0104	0	(53)	0.0017	1	U	K.VACEEPPFIENGAANLHSK.I
459	695.0065	2081.9976	2081.9840	0.0136	0	(52)	0.002	1	U	K.VACEEPPFIENGAANLHSK.I
471	713.3493	2137.0260	2137.0150	0.0110	2	86	7.4e-007	1	U	K.VKDKVQYECATGYYTAGGK.K
472	713.3498	2137.0276	2137.0150	0.0126	2	(77)	6.8e-006	1	U	K.VKDKVQYECATGYYTAGGK.K
473	535.2642	2137.0276	2137.0150	0.0126	2	(49)	0.0041	1	U	K.VKDKVQYECATGYYTAGGK.K
482	730.9993	2189.9761	2189.9721	0.0040	2	12	7	1	U	R.CEDGKWTEPPKCIEGQEK.V
489	1139.0604	2276.1063	2276.1107	-0.0043	0	(28)	0.52	1	U	K.QGYDLSPLTPLSELSVQCNR.G
490	1139.0678	2276.1211	2276.1107	0.0104	0	74	1.6e-005	1	U	K.QGYDLSPLTPLSELSVQCNR.G
576	899.7378	2696.1915	2696.1807	0.0109	1	(85)	1.9e-007	1	U	K.TTGGKDEEVVQCLSDGWSSQPTCR.K
577	899.7385	2696.1936	2696.1807	0.0129	1	(81)	4.9e-007	1	U	K.TTGGKDEEVVQCLSDGWSSQPTCR.K
578	899.7393	2696.1960	2696.1807	0.0153	1	100	6.2e-009	1	U	K.TTGGKDEEVVQCLSDGWSSQPTCR.K
579	899.7401	2696.1985	2696.1807	0.0178	1	(86)	1.9e-007	1	U	K.TTGGKDEEVVQCLSDGWSSQPTCR.K
580	899.7418	2696.2034	2696.1807	0.0227	1	(78)	1.1e-006	1	U	K.TTGGKDEEVVQCLSDGWSSQPTCR.K
598	921.1121	2760.3144	2760.3541	-0.0398	0	(87)	4.9e-007	1	U	K.HPPVVMNGAVADGILASYATGSSVEYR.C
601	926.4605	2776.3596	2776.3490	0.0105	0	(90)	2.5e-007	1	U	K.HPPVVMNGAVADGILASYATGSSVEYR.C + Oxidation (M)
602	926.4617	2776.3633	2776.3490	0.0142	0	109	3.3e-009	1	U	K.HPPVVMNGAVADGILASYATGSSVEYR.C + Oxidation (M)
641	1127.1646	3378.4720	3378.4627	0.0093	0	11	2.3	1	U	R.EAYCLDGMWTTPPLCLEPCTLSFTEMEK.N
668	1242.5616	3724.6630	3724.6410	0.0220	1	12	2.6	1	U	K.LSFFCLAGYTTESGRQEEQTTCTTEGWSPEPR.C

3. TRYP_PIG **Mass:** 25078 **Score:** 492 **Matches:** 14(12) **Sequences:** 6(6) **emPAI:** 1.88

Trypsin OS=Sus scrofa PE=1 SV=1

Check to include this hit in error tolerant search or archive report

Query	Observed	Mr(expt)	Mr(calc)	Delta	Miss	Score	Expect	Rank	Unique	Peptide
116	421.7600	841.5053	841.5022	0.0032	0	54	0.0028	1	U	R.VATVSLPR.S
117	421.7600	841.5055	841.5022	0.0033	0	(51)	0.005	1	U	R.VATVSLPR.S
161	523.2873	1044.5600	1044.5564	0.0037	0	49	0.011	1	U	K.LSSPATLNSR.V
162	523.2880	1044.5614	1044.5564	0.0050	0	(41)	0.071	1	U	K.LSSPATLNSR.V
163	532.2604	1062.5063	1062.5016	0.0048	0	94	2.4e-007	1	U	K.APVLSDSSCK.S
164	532.2605	1062.5064	1062.5016	0.0049	0	(72)	3.7e-005	1	U	K.APVLSDSSCK.S
484	737.7093	2210.1062	2210.0967	0.0095	0	104	1.4e-008	1	U	R.LGEHNIDVLEGNEQFINAAK.I
485	737.7099	2210.1080	2210.0967	0.0113	0	(97)	7.4e-008	1	U	R.LGEHNIDVLEGNEQFINAAK.I
491	761.7343	2282.1812	2282.1729	0.0083	0	(52)	0.0022	1	U	K.IITHPNFNGNTLDNDIMLIK.L
492	761.7346	2282.1819	2282.1729	0.0090	0	74	1.6e-005	1	U	K.IITHPNFNGNTLDNDIMLIK.L
494	761.7352	2282.1837	2282.1729	0.0108	0	(62)	0.00023	1	U	K.IITHPNFNGNTLDNDIMLIK.L
496	767.0671	2298.1796	2298.1678	0.0118	0	(48)	0.0054	1	U	K.IITHPNFNGNTLDNDIMLIK.L + Oxidation (M)
634	828.1907	3308.7339	3308.7187	0.0152	1	55	0.0005	1	U	K.IITHPNFNGNTLDNDIMLIKLSSPATLNSR.V
637	832.1907	3324.7339	3324.7136	0.0203	1	(21)	1.4	1	U	K.IITHPNFNGNTLDNDIMLIKLSSPATLNSR.V + Oxidation (M)

4. K2C1_HUMAN **Mass:** 66170 **Score:** 287 **Matches:** 8(7) **Sequences:** 7(6) **emPAI:** 0.43

Keratin, type II cytoskeletal 1 OS=Homo sapiens GN=KRT1 PE=1 SV=6

Check to include this hit in error tolerant search or archive report

Query	Observed	Mr(expt)	Mr(calc)	Delta	Miss	Score	Expect	Rank	Unique	Peptide
151	487.2714	972.5283	972.5240	0.0043	0	43	0.05	1		K.IEISELNR.V
226	651.8641	1301.7136	1301.7078	0.0057	0	82	3.9e-006	1	U	R.SLDLDSIIAEVK.A
227	651.8642	1301.7138	1301.7078	0.0060	0	(60)	0.0006	1	U	R.SLDLDSIIAEVK.A
242	670.8413	1339.6680	1339.6619	0.0061	1	80	5e-006	1	U	K.SKAEAESLYQSK.Y
264	692.3513	1382.6881	1382.6830	0.0051	0	74	1.9e-005	1	U	K.SLNNQFASFIDK.V
269	697.3731	1392.7317	1392.7249	0.0068	1	63	0.0003	1	U	R.TNAENEFVTIKK.D
296	738.3966	1474.7786	1474.7780	0.0006	0	33	0.33	1		R.FLEQQNQVLQTK.W
378	858.9339	1715.8533	1715.8438	0.0095	0	76	1.3e-005	1	U	K.QISNLQQSISDAEQR.G

Proteins matching the same set of peptides:

K2C1_PANTR **Mass:** 65621 **Score:** 287 **Matches:** 8(7) **Sequences:** 7(6)

Keratin, type II cytoskeletal 1 OS=Pan troglodytes GN=KRT1 PE=2 SV=1

5. K1C9_HUMAN **Mass:** 62255 **Score:** 54 **Matches:** 3(1) **Sequences:** 3(1) **emPAI:** 0.11

Keratin, type I cytoskeletal 9 OS=Homo sapiens GN=KRT9 PE=1 SV=3

Check to include this hit in error tolerant search or archive report

Query	Observed	Mr(expt)	Mr(calc)	Delta	Miss	Score	Expect	Rank	Unique	Peptide
165	533.2513	1064.4880	1064.4920	-0.0040	0	20	4.2	1	U	K.STMQELNSR.L
213	618.2713	1234.5280	1234.5215	0.0065	0	57	0.00026	1	U	R.FSSSGYGGGSSR.V
495	767.0521	2298.1344	2298.1123	0.0221	1	6	95	1	U	K.SDLEMQYETLQEELMALKK.N

6. K22E_HUMAN **Mass:** 65678 **Score:** 41 **Matches:** 3(1) **Sequences:** 3(1) **emPAI:** 0.11

Keratin, type II cytoskeletal 2 epidermal OS=Homo sapiens GN=KRT2 PE=1 SV=2

Check to include this hit in error tolerant search or archive report

Query	Observed	Mr(expt)	Mr(calc)	Delta	Miss	Score	Expect	Rank	Unique	Peptide
151	487.2714	972.5283	972.5240	0.0043	0	43	0.05	1		K.IEISELNR.V
268	464.5678	1390.6815	1390.6728	0.0087	1	23	2.7	1	U	R.SKEEAEALYHSK.Y
296	738.3966	1474.7786	1474.7780	0.0006	0	33	0.33	1		R.FLEQQNQVLQTK.W

7. K1C10_HUMAN **Mass:** 59020 **Score:** 26 **Matches:** 2(0) **Sequences:** 2(0) **emPAI:** 0.06

Keratin, type I cytoskeletal 10 OS=Homo sapiens GN=KRT10 PE=1 SV=6

Check to include this hit in error tolerant search or archive report

Query	Observed	Mr(expt)	Mr(calc)	Delta	Miss	Score	Expect	Rank	Unique	Peptide
152	502.2830	1002.5514	1002.5458	0.0056	1	12	55	1	U	K.SEITELRR.N
405	899.0126	1796.0107	1796.0043	0.0064	0	26	0.76	1	U	R.NVQALEIELQSQLALK.Q

Peptide matches not assigned to protein hits: (no details means no match)

Query	Observed	Mr(expt)	Mr(calc)	Delta	Miss	Score	Expect	Rank	Unique	Peptide
254	682.8921	1363.7696	1363.7235	0.0461	0	20	3.9	1		YTEEGEIGILIK
174	559.3114	1116.6082	1116.5849	0.0234	0	20	8.1	1		EPISMVNLSK
235	659.3488	1316.6830	1316.6711	0.0119	0	19	8.1	1		LLETVEDEELK
105	757.3910	756.3837	756.4242	-0.0405	0	18	5.4	1		SRPELR
324	532.5878	1594.7415	1594.7450	-0.0035	1	16	7.5	1		YLYDGFMSRAQTK + Oxidation (M)
312	787.4002	1572.7859	1572.7678	0.0181	1	16	14	1		AIVASENNMSRTHK + Oxidation (M)
156	515.7712	1029.5279	1029.5091	0.0188	0	16	22	1		IDQSNLLNN

154	512.2792	1022.5439	1022.5549	-0.0110	1	14	23	1	EAFLKYPR
181	567.3005	1132.5865	1132.5546	0.0319	0	14	33	1	QEVIGNQAMK + Oxidation (M)
398	887.9554	1773.8962	1773.8857	0.0105	2	14	23	1	GRVEELGEEIGRESSK
286	483.9267	1448.7583	1448.7524	0.0059	1	13	30	1	WAREADLNHIPK
148	471.7648	941.5151	941.4791	0.0360	2	13	26	1	GPRDADRR
113	418.1658	834.3170	834.3654	-0.0484	0	13	3.5	1	VEGSCQR
218	639.8372	1277.6598	1277.6550	0.0048	2	13	43	1	KVCRWDSLSK
112	418.1658	834.3170	834.3654	-0.0484	0	12	4.1	1	VEGSCQR
114	835.4680	834.4607	834.4712	-0.0105	1	12	57	1	RVYEIR
667	617.8473	3701.0399	3701.0080	0.0319	2	12	2.6	1	QVQILAEKLLADNIVPFFPETLTKYGDLELQR
355	840.4048	1678.7949	1678.7686	0.0263	0	11	26	1	QVVQSEDSPFDSDVK
123	429.7334	857.4523	857.4971	-0.0448	1	11	67	1	VPVKSNSK
294	734.8318	1467.6491	1467.6594	-0.0103	0	11	14	1	GEMCLDLMLMQK
155	512.2799	1022.5452	1022.5549	-0.0097	1	10	58	1	EAFLKYPR
185	583.2932	1164.5718	1164.5775	-0.0057	1	10	59	1	EVNRYLDEK
198	601.8176	1201.6207	1201.6277	-0.0070	0	10	74	1	VNKPMLGHYK + Oxidation (M)
338	414.6972	1654.7599	1654.7120	0.0479	1	10	24	1	KFMDQHPEMDFSK + Oxidation (M)
288	483.9268	1448.7585	1448.7260	0.0325	0	10	64	1	AVSVGDGFEVVSQR
234	659.3487	1316.6828	1316.6936	-0.0108	1	10	71	1	LIEASAKVTDDR
182	574.8060	1147.5974	1147.5655	0.0319	2	10	87	1	KNKSECSPAK
411	910.3774	1818.7403	1818.7885	-0.0482	1	10	2.7	1	YMKCGIGVCGSCNLGK + Oxidation (M)
199	601.8179	1201.6213	1201.6277	-0.0064	0	10	83	1	VNKPMLGHYK + Oxidation (M)
375	570.9539	1709.8398	1709.8737	-0.0339	1	9	51	1	QPDWPAIADRLSDVK
342	829.3632	1656.7119	1656.7092	0.0027	1	9	12	1	LQEMMRIMCEER + 2 Oxidation (M)
266	463.2017	1386.5833	1386.5730	0.0103	0	9	11	1	NPFVMMMDSGAR + 2 Oxidation (M)
120	846.4455	845.4382	845.4131	0.0251	0	9	1.4e+002	1	DEAVGDLK
407	601.3145	1800.9217	1800.8795	0.0422	2	9	66	1	KRGYDDLNDFFGNLK
313	526.2787	1575.8142	1575.8079	0.0063	1	8	91	1	MFKAELNDPNILR + Oxidation (M)
146	469.7182	937.4218	937.4658	-0.0439	1	8	50	1	REGIGYPF
160	521.2726	1040.5307	1040.5614	-0.0308	0	8	74	1	TPEVALNAAR
335	551.9651	1652.8735	1652.8886	-0.0150	2	8	75	1	TDPSYRKIALSFQK
147	471.7582	941.5017	941.4930	0.0087	1	8	89	1	KDPELANR
343	829.3636	1656.7126	1656.7092	0.0034	1	8	17	1	LQEMMRIMCEER + 2 Oxidation (M)
122	429.7280	857.4415	857.4178	0.0238	0	8	1.3e+002	1	AHSAGLMR + Oxidation (M)
354	840.4040	1678.7934	1678.8097	-0.0162	1	8	63	1	RNALFADMQQGSDVK
275	703.3539	1404.6932	1404.6820	0.0112	0	7	1.1e+002	1	NQGISYSQLMHK
756	5892.9603	5891.9530	5891.9482	0.0048	2	7	7.5	1	MNIGQILETHLGWVAKSGWNIDGNPEWAVNLPEELRHAQPNQIVSTPVFDGAK + Oxidation (M)
287	725.3865	1448.7585	1448.7524	0.0061	1	7	1.3e+002	1	IQWHGKDNQVPK
465	1062.0387	2122.0629	2122.0994	-0.0364	2	7	82	1	GVTKVKPCSTKFTFHTER
327	546.5903	1636.7490	1636.7297	0.0193	1	7	55	1	KCVSCQNTNPTSNK
262	690.8773	1379.7400	1379.7271	0.0129	0	7	1.2e+002	1	VFGIQNFSPMLK
464	705.0095	2112.0068	2112.0422	-0.0354	2	7	61	1	MSAKELIDAFNGVREFNR + Oxidation (M)
391	580.9546	1739.8420	1739.8730	-0.0310	0	7	84	1	EFYELAQTLQTELR
518	608.0392	2428.1278	2428.1342	-0.0064	0	6	42	1	MINQANNQVNFSEQQFVHHK + Oxidation (M)
337	552.2593	1653.7561	1653.7345	0.0217	0	6	53	1	AGEQVTYTCATYYK
574	446.7346	2674.3642	2674.3948	-0.0305	2	6	71	1	MNAIFVANKPAGMSSNHFLGRLKR + Oxidation (M)
118	846.4450	845.4377	845.4494	-0.0117	0	6	2.8e+002	1	AEEIGLSK
263	690.8776	1379.7406	1379.7482	-0.0076	2	6	1.3e+002	1	MKINKYVEDLK
37	559.3020	558.2948	558.3125	-0.0178	0	6	3.4e+002	1	LRPSS
599	921.4582	2761.3528	2761.3911	-0.0383	2	6	67	1	HGFIMAHLPAGSAARFDFQKYIER
650	1139.5564	3415.6473	3415.6419	0.0053	2	6	41	1	GIVGGKAGEELEIDVTFPEDYHNEDLAGKDAK
428	947.9109	1893.8073	1893.7588	0.0485	1	6	18	1	TDGYRWCGTTEDYDR
310	524.5910	1570.7511	1570.7160	0.0351	0	6	94	1	FSNFPDLIMMDGGK
463	699.6765	2096.0078	2096.0071	0.0007	0	5	91	1	DGMAIDPTGKPFAFEIMTR
519	608.0398	2428.1303	2428.1342	-0.0040	0	5	57	1	MINQANNQVNFSEQQFVHHK + Oxidation (M)
498	772.3634	2314.0685	2314.1185	-0.0499	0	5	56	1	MSLSSDDMTSALFLPKPISSR + 2 Oxidation (M)
692	745.3901	4466.2971	4466.3389	-0.0418	2	5	24	1	SMILPDMVGVTIAVHNGKNHIPVLVNENMVGHKLGEFAITR + Oxidation (M)
564	441.7276	2644.3216	2644.3088	0.0129	2	5	1e+002	1	AMMANVDELNKSIQSLTKYTIDK + 2 Oxidation (M)
328	546.9195	1637.7366	1637.7257	0.0109	0	5	72	1	GAVAGCGVFFGNDDPR
415	607.9501	1820.8286	1820.8628	-0.0342	0	5	66	1	MSRPHFQFETDLAAR + Oxidation (M)
119	846.4451	845.4379	845.4858	-0.0479	1	5	3.8e+002	1	ISEIEKK
454	693.6790	2078.0153	2078.0102	0.0051	0	5	1.2e+002	1	QTSSAYDAPAAAIAGMVDAIR
715	617.7968	4934.3159	4934.3253	-0.0094	2	5	16	1	GDAVLHPMGWDAFGLPAENAAIARNVDPGDWTDQNIAQMRAQLDR + 2 Oxidation (M)
282	473.5494	1417.6264	1417.6586	-0.0322	2	5	54	1	DERTNKEHDFK
734	763.3850	5336.6438	5336.6585	-0.0147	2	5	19	1	YLTGKEIEVDAICDGETVVIPGIMEHIERAGVHSGDSIAVYPPQTLTEK
737	5443.8428	5442.8355	5442.8531	-0.0176	0	5	9.9	1	DIATVLQNLKPDVALVMLHGKPGEDGTIQGVLETLGIPYSHSGVLSSSLAMR + Oxidation (M)
187	1165.5972	1164.5899	1164.5887	0.0012	2	5	2.4e+002	1	ERYRGEDLK
711	606.2907	4842.2672	4842.2543	0.0129	0	5	16	1	GTNIDALNDALHGVDALYTYEPNLLLTMCYADCVPIYFYSEK
475	536.5276	2142.0811	2142.0422	0.0389	2	4	1.4e+002	1	QSRMQFSSLAVNVRGSGMR + 2 Oxidation (M)
466	1062.0419	2122.0692	2122.0511	0.0181	2	4	1.5e+002	1	KNGKIAVGGDGQVSMGNTVMK + 2 Oxidation (M)
233	439.9015	1316.6828	1316.6823	0.0004	0	4	2.7e+002	1	LLETINVSGEDK
186	583.2934	1164.5722	1164.5999	-0.0278	2	4	2.5e+002	1	QKGQTERYR
680	1049.5360	4194.1151	4194.0799	0.0352	2	4	50	1	FPQKHYQMSVLESLLINSTVGRVLAMDLDEGVNAEMK + 2 Oxidation (M)
139	456.7064	911.3982	911.4209	-0.0227	0	4	94	1	SHNIDNGR
678	686.3510	4112.0623	4112.0142	0.0481	2	4	53	1	CVPGLVLMAPSDENELRHMLHTSLQHDGPAALRYPR + 2 Oxidation (M)
236	439.9016	1316.6830	1316.6823	0.0006	0	4	3e+002	1	LLETINVSGEDK
250	680.8160	1359.6174	1359.5725	0.0450	0	4	1e+002	1	ASAAEQGMEEHGK + Oxidation (M)
563	880.1219	2637.3438	2637.3796	-0.0358	2	4	1.4e+002	1	EKKVDGVVSAGNTGAVMSSALLYLGR + Oxidation (M)
557	1308.6752	2615.3358	2615.3402	-0.0044	1	3	1.5e+002	1	LGDGLDPDSVSLASVTALTSNVSNKR
403	892.4951	1782.9757	1782.9363	0.0395	2	3	1.9e+002	1	KTGEALYKLASSESATK
348	557.2658	1668.7755	1668.8206	-0.0451	1	3	1.4e+002	1	IKQEPDDDLTEAAPK
479	719.6877	2156.0412	2156.0597	-0.0185	0	3	1.6e+002	1	APVSPALASPSSEVSDVSEATR
750	1414.9197	5655.6497	5655.6142	0.0355	2	3	16	1	QSIANNDATKASEDYVDANNDKQTAYNNAVAAAETIINANSNPEMNPSTITQK + Oxidation (M)
644	567.2804	3397.6389	3397.6130	0.0259	0	3	94	1	SSLPLIVSDPTSQQQSNMSSLSPMQSTGTSFR
672	638.3431	3824.0152	3824.0176	-0.0024	2	3	51	1	LETLTACGGISNKEIVKLVADDPIGLRPDMTASIAR
340	552.5939	1654.7599	1654.7872	-0.0273	1	3	1.3e+002	1	GITDDMKSFQSAIDK
222	647.8351	1293.6557	1293.6751	-0.0194	0	3	3.4e+002	1	ASYICNILNVK
661	907.9124	3627.6206	3627.6677	-0.0471	2	3	19	1	GGMGVDLALDRVPMREEGMTPYELMLSESQER + 2 Oxidation (M)
434	646.3047	1935.8924	1935.9373	-0.0449	2	3	1.2e+002	1	YAVFEIEQHDMRRAR + Oxidation (M)
406	601.3143	1800.9210	1800.8728	0.0482	2	3	2.7e+002	1	RDHTDNPSQRVSHPR
168	549.7581	1097.5016	1097.5506	-0.0490	0	3	1.8e+002	1	QFSDYGLIR
486	1130.5553	2259.0960	2259.1247	-0.0287	2	3	1.7e+002	1	ICEMLIPKMSKQAINHMAL + 2 Oxidation (M)
339	552.5939	1654.7599	1654.7872	-0.0274	1	3	1.4e+002	1	GITDDMKSFQSAIDK
621	1022.1200	3063.3381	3063.3457	-0.0076	1	3	20	1	LWEASGHWDNYRSHMFIAEVEDEDK
509	791.3519	2371.0339	2371.0155	0.0184	1	3	43	1	NMVETSDGLEASENEKEVSCK + Oxidation (M)
173	559.2922	1116.5698	1116.5928	-0.0230	0	2	4.4e+002	1	QSFTSIIHGK
462	699.6748	2096.0027	2096.0208	-0.0181	0	2	1.7e+002	1	ALEECGLPAAAVQAIESPDR
670	540.2863	3774.9535	3774.9105	0.0431	0	2	75	1	AYADEALPIGLNQTISQPFTVAYQTALLGVQPDDR
717	624.8339	4990.6132	4990.6123	0.0009	2	2	22	1	DPCGIFGLVLTPTRELAIQIAEQFTALGANMNIRVALIYGGVDMVK + Oxidation (M)
453	693.6786	2078.0139	2078.0540	-0.0401	1	2	2.2e+002	1	TQVGEMTVMVKDFHLLSK + Oxidation (M)
556	1308.6733	2615.3321	2615.3528	-0.0207	2	2	2e+002	1	QSRADRYVVEATSGSSLNPGLRPR
554	872.7832	2615.3277	2615.3609	-0.0331	2	2	2e+002	1	EAVANKGPVSVGVDARHPSFFLYR
677	1020.7324	4078.9007	4078.8750	0.0256	1	2	45	1	LVLEEEAWAAANDFEDLMLTDQVYMHRDMLPEPR + 2 Oxidation (M)
520	608.0411	2428.1353	2428.1342	0.0010	0	2	1.3e+002	1	MINQANNQVNFSEQQFVHHK + Oxidation (M)
767	6508.4503	6507.4430	6507.4498	-0.0067	2	2	6	1	GLFRVTIDGITDQFSHQKLIITPNHVSFLDGALLALFLPIKPVFAVYSNITESWYMR + Oxidation (M)
444	1019.4439	2036.8733	2036.9084	-0.0351	0	2	41	1	IHQMNCFDFLDQVENK
553	1308.6711	2615.3277	2615.3528	-0.0251	2	2	2.1e+002	1	QSRADRYVVEATSGSSLNPGLRPR
745	5624.5669	5623.5596	5623.5727	-0.0131	2	2	9.3	1	TQLAVEMYDVAKKLCSFCEGLVHDEHLQHQGWAAIMANLEDCSNSYQK
390	580.9544	1739.8414	1739.8301	0.0113	0	2	2.6e+002	1	ASFEAAQFAVTPQAMR + Oxidation (M)
325	533.2286	1596.6641	1596.7103	-0.0462	2	2	44	1	GEFHMNYDRERK + Oxidation (M)
456	694.3349	2079.9829	2080.0324	-0.0496	0	2	1.9e+002	1	EEQPEVNLTITAVDGGSPPK
149	945.4178	944.4105	944.4563	-0.0458	0	1	1.3e+002	1	SLSQEPER
738	682.8856	5455.0268	5455.0734	-0.0466	2	1	2.5	1	LAWAVGLGTIPAGLAGLLLFHDIIATHLRSAQVIAMATIGFAVLLLFADVSGKR
610	588.2986	2936.4568	2936.4953	-0.0385	1	1	2e+002	1	DADALDAMAAAVIVERWLSAPEQATLLP
640	560.7827	3358.6527	3358.6730	-0.0203	2	1	1.6e+002	1	GTKGFVTNVLESRAPEFDVFYSCGPLPMLK
555	872.7847	2615.3321	2615.3806	-0.0485	2	1	2.6e+002	1	VLDAAVAGKITDVDVLKAQFEEER
85	692.0411	2073.1015	2073.1140	-0.0125	1	1	3.2e+002	1	LTMVELGKLVGLSSPSAAER + Oxidation (M)
597	920.7976	2759.3711	2759.3490	0.0222	1	1	2.4e+002	1	EVRSDNTVMWLAAWVEHIQNSFK
357	563.2473	1686.7201	1686.6953	0.0249	1	1	68	1	IDKHMYHDMYMR + 3 Oxidation (M)
425	624.9426	1871.8059	1871.8550	-0.0492	1	1	74	1	SGAWYSYNEERLGQGR
358	563.2480	1686.7221	1686.7382	-0.0161	0	1	76	1	LQEDCMTIWYNSK
762	777.9003	6215.1442	6215.1316	0.0126	2	1	26	1	VQFNSVQELVQHYMEVNDGLCNLLIAPCTIMKPQTLGLAKDAWEISRSSITLER
249	454.2131	1359.6174	1359.6419	-0.0245	0	1	2.1e+002	1	SSPPPVFSDNGTR
508	1185.5316	2369.0487	2369.0747	-0.0260	1	1	78	1	SGFKSTHEFVTCFGDSIDAHK
701	572.7818	4574.1964	4574.2403	-0.0440	2	1	72	1	VAGALQAGVAADCVVVGCDSMLFIDGGLCGKPGSADAALRQWRR
419	608.2846	1821.8320	1821.8423	-0.0104	0	0	2.1e+002	1	LVCSITDGIPAQDMMR + Oxidation (M)

729	662.2280	5289.7658	5289.7320	0.0338	2	0	35	1	GAIENGNYIGLALMIITSLIAMFSLFRIFFVMYLGNENKGEAIVFNK + 3 Oxidation (M)
757	5918.5668	5917.5595	5917.5816	-0.0221	1	0	2.5	1	VPPPPETPAQEEMETETEASAPQGKEQDDTAAMLADFIDCPPDDEKPPPATEPDS
730	884.9725	5303.7912	5303.7773	0.0139	2	0	29	1	VLALYNQHNPGASAAPCCVPQVLEPLPIVYYVGRKPKVEQLSNMIVR + Oxidation (M)
558	872.7859	2615.3358	2615.3853	-0.0495	2	0	3.1e+002	1	ENDLRLLKILLLNQSCDFQQR
336	827.4440	1652.8735	1652.8352	0.0384	2	0	4.7e+002	1	MVHGRMVHGRSVSGK + Oxidation (M)
510	794.7002	2381.0787	2381.0634	0.0153	2	0	1.2e+002	1	EQMMNHSMSSGSGSLRTNQKR + Oxidation (M)
293	734.8316	1467.6486	1467.6594	-0.0107	0	0	1.8e+002	1	GEMCLDLMLMQK
1	415.1272	414.1200							
2	415.2122	414.2049							
3	415.2126	414.2054							
4	415.2150	414.2077							
5	415.3358	414.3285							
6	415.3430	414.3357							
7	419.1685	418.1612							
8	419.1704	418.1631							
9	432.2395	431.2322							
10	432.2398	431.2326							
11	432.2411	431.2338							
12	433.1815	432.1742							
13	441.9034	440.8961							
14	444.1175	443.1102							
15	444.1176	443.1103							
16	444.1179	443.1106							
17	444.1197	443.1125							
18	447.1376	446.1303							
19	453.7869	452.7796							
20	455.3364	454.3291							
21	474.8329	473.8257							
22	500.3071	499.2999							
23	500.3071	499.2999							
24	500.3080	499.3007							
25	502.3747	501.3674							
26	503.3084	502.3011							
27	506.3205	505.3132							
28	515.7740	514.7668							
29	520.3346	519.3273							
30	526.5211	525.5138							
31	528.3649	527.3576							
32	529.3691	528.3618							
33	537.3007	536.2934							
34	542.2262	541.2189							
35	545.7720	544.7648							
36	550.3436	549.3363							
38	563.2464	562.2391							
39	579.5356	578.5283							
40	579.5363	578.5290							
41	579.5363	578.5291							
42	579.5366	578.5294							
43	586.4190	585.4117							
44	590.4284	589.4211							
45	601.5421	600.5348							
46	601.5422	600.5349							
47	601.5426	600.5353							
49	610.8280	609.8207							
50	613.3639	612.3566							
51	615.4045	614.3972							
52	615.4046	614.3973							
53	615.8424	614.8351							
54	617.4079	616.4006							
55	617.4079	616.4006							
56	630.4433	629.4360							
57	630.4441	629.4368							
58	634.4554	633.4481							
59	648.8466	647.8393							
60	650.2075	649.2002							
61	661.5622	660.5550							
62	661.5629	660.5556							
63	661.5657	660.5584							
64	663.4552	662.4479							
65	663.4565	662.4492							
66	674.4704	673.4632							
67	674.4728	673.4655							
68	675.6771	674.6698							
69	675.6773	674.6700							
70	675.6776	674.6703							
71	675.6778	674.6705							
72	675.6782	674.6710							
73	675.6783	674.6710							
74	675.6785	674.6712							
75	675.6877	674.6804							
76	677.6848	676.6775							
77	677.6850	676.6777							
78	677.8702	676.8629							
79	678.5891	677.5818							
80	678.5903	677.5830							
81	678.5903	677.5831							
82	683.5448	682.5376							
83	683.5452	682.5379							
84	683.5459	682.5387							
86	717.6217	716.6144							
87	717.6252	716.6179							
88	717.6263	716.6190							
89	717.6334	716.6261							
90	718.4965	717.4892							
91	720.4322	719.4249							
92	724.5821	723.5749							
93	732.4788	731.4715							
94	734.6505	733.6432							

95	734.6549	733.6476
96	739.6038	738.5965
97	739.6054	738.5981
98	739.6059	738.5986
99	739.6062	738.5989
100	739.6068	738.5995
101	739.6080	738.6008
102	752.5484	751.5412
103	752.5484	751.5412
104	752.5485	751.5413
108	762.5236	761.5163
111	809.4837	808.4764
115	419.2028	836.3911
121	850.5762	849.5690
126	861.3980	860.3907
138	453.7888	905.5630
140	458.1871	914.3596
141	934.8811	933.8739
142	934.8817	933.8744
143	934.8820	933.8748
144	934.8821	933.8748
145	468.6670	935.3194
150	481.6903	961.3661
153	1021.9463	1020.9390
159	516.7903	1031.5660
166	542.2304	1082.4463
167	1089.0036	1087.9963
188	585.2690	1168.5234
189	585.2732	1168.5318
192	592.2288	1182.4431
193	593.2646	1184.5146
200	602.2671	1202.5196
201	602.7111	1203.4077
202	610.2520	1218.4894
203	610.2523	1218.4899
210	617.2661	1232.5177
214	625.2656	1248.5166
217	421.5593	1261.6561
219	640.7227	1279.4309
220	640.7229	1279.4312
221	647.8332	1293.6519
237	659.5611	1317.1076
247	678.3212	1354.6278
248	680.7456	1359.4767
257	457.2087	1368.6043
258	457.2116	1368.6129
259	688.3028	1374.5910
265	692.8467	1383.6788
267	694.2989	1386.5833
270	467.1800	1398.5181
276	703.7953	1405.5761
277	706.3245	1410.6344
285	481.5392	1441.5959
295	735.3620	1468.7095
297	740.8858	1479.7570
301	749.8105	1497.6064
302	500.5694	1498.6864
303	503.8966	1508.6679
304	507.9380	1520.7921
307	519.5425	1555.6058
311	524.5913	1570.7520
314	789.8538	1577.6931
326	539.3395	1614.9967
331	549.8982	1646.6729
332	827.2773	1652.5400
333	551.8539	1652.5400
334	827.2774	1652.5402
341	553.2446	1656.7119
344	553.2448	1656.7126
349	837.3595	1672.7045
350	558.5760	1672.7061
351	837.3607	1672.7069
352	558.5762	1672.7069
353	420.5741	1678.2674
356	842.3912	1682.7678
369	849.3264	1696.6382
370	566.5534	1696.6382
371	849.3272	1696.6398
372	566.5539	1696.6398
376	572.2513	1713.7322
377	572.2514	1713.7323
379	860.7602	1719.5058
380	574.1759	1719.5058
381	860.7615	1719.5084
382	574.1767	1719.5084
383	574.1790	1719.5151
384	576.2139	1725.6198
385	578.2441	1731.7106
386	578.8472	1733.5198
387	867.7672	1733.5198
388	580.8927	1739.6562
389	870.9271	1739.8397
392	582.2568	1743.7487
393	583.9186	1748.7339
400	891.4932	1780.9717
410	907.4194	1812.8242

416	911.4216	1820.8286
421	609.9451	1826.8135
422	612.3028	1833.8865
423	612.5862	1834.7369
424	612.9738	1835.8996
426	940.9448	1879.8751
427	947.5717	1893.1288
435	648.6821	1943.0244
436	649.8647	1946.5724
437	655.2219	1962.6438
439	983.4152	1964.8158
442	666.6662	1996.9769
443	679.3681	2035.0823
455	693.8827	2078.6262
460	698.6452	2092.9137
461	698.6455	2092.9146
467	709.6311	2125.8716
468	711.3176	2130.9309
469	712.6420	2134.9043
470	712.6425	2134.9056
474	1069.8241	2137.6336
478	718.0211	2151.0415
481	726.0036	2174.9888
483	731.6703	2191.9890
487	755.3478	2263.0216
488	755.6826	2264.0261
493	1142.0982	2282.1819
497	1154.5155	2307.0165
499	772.3636	2314.0690
504	777.3530	2329.0371
505	777.3531	2329.0374
506	588.3564	2349.3966
507	787.6534	2359.9384
511	794.7005	2381.0797
512	1202.0148	2402.0151
513	1202.0153	2402.0161
517	808.6669	2422.9790
524	819.3177	2454.9312
525	819.3182	2454.9328
526	838.8584	2513.5535
527	848.9144	2543.7214
533	861.0525	2580.1356
534	649.0214	2592.0564
535	865.0284	2592.0635
536	865.0318	2592.0735
537	649.3592	2593.4077
538	1299.0601	2596.1056
545	870.0207	2607.0404
546	870.0208	2607.0404
547	870.0210	2607.0411
549	1307.6613	2613.3080
552	1307.6616	2613.3087
559	875.3525	2623.0356
560	875.3527	2623.0362
561	878.6912	2633.0517
562	528.3475	2636.7010
565	885.7506	2654.2300
566	885.7508	2654.2305
567	664.5649	2654.2305
568	885.7513	2654.2321
569	444.1181	2658.6651
570	889.7482	2666.2228
571	889.7483	2666.2230
572	534.3164	2666.5457
573	890.4234	2668.2483
575	539.3255	2691.5913
581	904.4034	2710.1884
582	904.4109	2710.2109
583	904.4118	2710.2135
584	908.1179	2721.3319
587	908.1187	2721.3343
588	910.4182	2728.2329
589	685.3352	2737.3119
590	917.3763	2749.1070
591	688.2840	2749.1071
592	688.2859	2749.1144
600	925.7955	2774.3646
603	465.1729	2784.9938
604	558.5808	2787.8675
605	465.6700	2787.9766
606	703.5911	2810.3354
607	964.4588	2890.3546
608	973.0385	2916.0936
609	734.6526	2934.5814
611	594.3667	2966.7972
613	433.1333	3024.8823
616	764.4428	3053.7421
620	1532.6763	3063.3381
622	622.3639	3106.7831
623	1038.7616	3113.2630
624	1038.7618	3113.2637
625	1038.7622	3113.2649
626	1054.2949	3159.8629
627	790.9730	3159.8629
628	639.8226	3194.0765
629	807.4274	3225.6807

630	539.3123	3229.8301
631	652.4039	3256.9832
632	412.1702	3289.3036
633	550.3446	3296.0239
635	1103.9186	3308.7339
636	1104.5881	3310.7426
642	1127.6316	3379.8728
643	1130.4886	3388.4439
645	850.6107	3398.4139
646	1133.8119	3398.4139
653	1144.8088	3431.4047
654	867.3785	3465.4847
655	1156.1689	3465.4848
656	867.3788	3465.4861
657	871.3766	3481.4773
658	871.3766	3481.4773
659	871.3770	3481.4787
660	602.2552	3607.4875
662	908.4217	3629.6577
663	728.8854	3639.3909
664	458.1804	3657.3852
665	458.3491	3658.7347
666	615.3027	3685.7728
671	945.4029	3777.5823
673	549.7635	3841.2936
674	641.7648	3844.5451
675	654.3844	3920.2629
676	664.9176	3983.4619
679	829.7123	4143.5250
681	525.3223	4194.5200
682	606.3218	4237.2017
683	539.7436	4309.8904
684	618.8141	4324.6480
685	546.4017	4363.1556
686	4372.5604	4371.5531
687	4395.7969	4394.7896
688	1099.9611	4395.8154
689	4403.2149	4402.2076
690	4436.0738	4435.0665
691	1113.1875	4448.7211
693	1506.6376	4516.8909
694	1506.6378	4516.8915
695	1506.6388	4516.8947
696	4525.8975	4524.8902
697	650.2075	4544.4015
698	910.8874	4549.4006
699	652.4277	4559.9432
700	1143.4591	4569.8072
702	1145.4620	4577.8188
703	4621.9247	4620.9174
704	1156.6861	4622.7153
705	1172.7422	4686.9397
706	1172.7434	4686.9443
707	791.8249	4744.9059
708	4755.7686	4754.7613
709	680.7352	4758.0956
710	1200.6303	4798.4920
712	613.3357	4898.6278
713	615.4080	4915.2059
714	822.8533	4931.0764
716	1234.7352	4934.9117
718	714.3810	4993.6159
719	5006.3432	5005.3359
720	5007.9433	5006.9360
721	5008.0722	5007.0649
722	628.2752	5018.1436
723	628.7731	5022.1269
724	840.4031	5036.3748
725	634.4362	5067.4313
726	846.8609	5075.1217
727	644.2350	5145.8217
728	5202.7784	5201.7711
731	664.4191	5307.2947
732	665.3866	5315.0348
733	5320.2097	5319.2024
735	5384.5644	5383.5571
736	904.4694	5420.7727
739	911.4251	5462.5070
740	5504.5833	5503.5760
741	689.8060	5510.3898
742	695.6564	5557.1928
743	5578.0198	5577.0125
744	5618.3189	5617.3116
746	5640.6723	5639.6650
747	1414.9169	5655.6386
748	1414.9190	5655.6468
749	1414.9190	5655.6468
751	1414.9198	5655.6501
752	1414.9201	5655.6513
753	5663.2262	5662.2189
754	974.3722	5840.1893
755	734.6525	5869.1615
758	5933.6447	5932.6374
759	747.3746	5970.9384
760	6026.7211	6025.7138
761	6037.3821	6036.3748

	763	777.9137	6215.2516
☑	764	791.3681	6322.8868
☑	765	1069.5008	6410.9614
☑	766	6423.0990	6422.0917
☑	768	6624.4769	6623.4696
☑	769	6682.9020	6681.8947
☑	770	842.3891	6731.0542
☑	771	6857.9300	6856.9227
☑	772	6861.3746	6860.3673
☑	773	6877.8593	6876.8520
☑	774	983.4628	6877.1886
☑	775	1157.8445	6941.0233
☑	776	868.6352	6941.0233
☑	777	7086.0692	7085.0619
☑	778	7127.8785	7126.8712
☑	779	1038.4815	7262.3197
☑	780	908.9807	7263.7876
☑	781	7273.2353	7272.2280
☑	782	7287.4409	7286.4336
☑	783	1243.8963	7457.3343
☑	784	1248.5663	7485.3542
☑	785	1278.0685	7662.3675
☑	786	7739.5286	7738.5213
☑	787	8055.4159	8054.4086
☑	788	1155.0568	8078.3468
☑	789	8877.3495	8876.3422
☑	790	9132.7700	9131.7627
☑	791	9532.3817	9531.3744
☑	792	10909.9918	10908.9845
☑	793	11564.9140	11563.9067

Search Parameters

Type of search	: MS/MS Ion Search
Enzyme	: Trypsin
Fixed modifications	: Carbamidomethyl (C)
Variable modifications	: Oxidation (M)
Mass values	: Monoisotopic
Protein Mass	: Unrestricted
Peptide Mass Tolerance	: ± 0.05 Da
Fragment Mass Tolerance	: ± 0.05 Da
Max Missed Cleavages	: 2
Instrument type	: Default
Number of queries	: 793

Mascot: http://www.matrixscience.com/

Mascot Search Results

User : Julian
Email : julian.langer@mpibp-frankfurt.mpg.de
Search title : Submitted from 140318 842 ChKrettler 1-10 swissprot full T 0.05Da by Mascot Daemon on CCSW010
MS data file : D:\Data\1403\140318\140314 842 ChReinhard 10_Tray01-D6_01_17691.d\140314 842 ChReinhard 10_Tray01-D6_01_17691.mgf
Database : SwissProt 57.15 (515203 sequences; 181334896 residues)
Timestamp : 19 Mar 2014 at 09:53:27 GMT
Protein hits :
ALBU_HUMAN Serum albumin OS=Homo sapiens GN=ALB PE=1 SV=2
F13A_HUMAN Coagulation factor XIII A chain OS=Homo sapiens GN=F13A1 PE=1 SV=4
K2C1_HUMAN Keratin, type II cytoskeletal 1 OS=Homo sapiens GN=KRT1 PE=1 SV=6
TRYP_PIG Trypsin OS=Sus scrofa PE=1 SV=1
F13B_HUMAN Coagulation factor XIII B chain OS=Homo sapiens GN=F13B PE=1 SV=3
K1C9_HUMAN Keratin, type I cytoskeletal 9 OS=Homo sapiens GN=KRT9 PE=1 SV=3
K1C10_HUMAN Keratin, type I cytoskeletal 10 OS=Homo sapiens GN=KRT10 PE=1 SV=6
K22E_HUMAN Keratin, type II cytoskeletal 2 epidermal OS=Homo sapiens GN=KRT2 PE=1 SV=2
A1BG_HUMAN Alpha-1B-glycoprotein OS=Homo sapiens GN=A1BG PE=1 SV=3
K1C14_HUMAN Keratin, type I cytoskeletal 14 OS=Homo sapiens GN=KRT14 PE=1 SV=4
HEMO_HUMAN Hemopexin OS=Homo sapiens GN=HPX PE=1 SV=2
K1M1_SHEEP Keratin, type I microfibrillar 48 kDa, component 8C-1 OS=Ovis aries PE=1 SV=2
K2M3_SHEEP Keratin, type II microfibrillar, component 5 OS=Ovis aries PE=1 SV=1
CO4A_HUMAN Complement C4-A OS=Homo sapiens GN=C4A PE=1 SV=1

Mascot Score Histogram

Ions score is -10*Log(P), where P is the probability that the observed match is a random event.
Individual ions scores > 38 indicate identity or extensive homology (p<0.05).
Protein scores are derived from ions scores as a non-probabilistic basis for ranking protein hits.

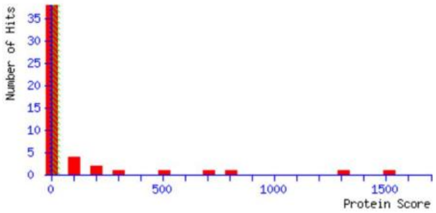

Peptide Summary Report

Format As: Peptide Summary — Help

Significance threshold p< 0.05 — Max. number of hits AUTO

Standard scoring — MudPIT scoring — Ions score or expect cut-off 0 — Show sub-sets 0

Show pop-ups — Suppress pop-ups — Sort unassigned Decreasing Score — Require bold red

Select All — Select None — Search Selected — Error tolerant — Archive Report

1. ALBU_HUMAN **Mass:** 71317 **Score:** 1513 **Matches:** 84(44) **Sequences:** 38(23) **emPAI:** 3.34
Serum albumin OS=Homo sapiens GN=ALB PE=1 SV=2
Check to include this hit in error tolerant search or archive report

Query	Observed	Mr(expt)	Mr(calc)	Delta	Miss	Score	Expect	Rank	Unique	Peptide
127	772.4416	771.4344	771.4313	0.0031	0	27	2.7	1		K.AACLLPK.L
128	772.4422	771.4349	771.4313	0.0036	0	(21)	9.7	1		K.AACLLPK.L
129	789.4750	788.4677	788.4644	0.0033	0	(35)	0.29	1		K.LVTDLTK.V
130	789.4750	788.4678	788.4644	0.0034	0	54	0.0035	1		K.LVTDLTK.V
152	438.2495	874.4845	874.5025	-0.0179	1	(24)	4.4	1		R.LSQRFPK.A
153	438.2498	874.4851	874.5025	-0.0174	1	(13)	53	3		R.LSQRFPK.A
154	438.2518	874.4890	874.5025	-0.0135	1	(23)	6.3	1		R.LSQRFPK.A
155	438.2586	874.5027	874.5025	0.0003	1	33	0.49	1		R.LSQRFPK.A
159	440.7256	879.4367	879.4338	0.0029	0	(29)	1	1	U	K.AEFAEVSK.L
160	440.7262	879.4379	879.4338	0.0041	0	63	0.00036	1	U	K.AEFAEVSK.L
161	880.4452	879.4379	879.4338	0.0041	0	(19)	8.6	1	U	K.AEFAEVSK.L
162	440.7263	879.4380	879.4338	0.0042	0	(40)	0.076	1	U	K.AEFAEVSK.L
166	464.2524	926.4902	926.4861	0.0041	0	35	0.15	1		K.YLYEIAR.R
167	927.4981	926.4908	926.4861	0.0047	0	(34)	0.18	1		K.YLYEIAR.R
168	464.2527	926.4908	926.4861	0.0047	0	(32)	0.29	1		K.YLYEIAR.R
169	467.2652	932.5159	932.5113	0.0046	0	31	0.75	1		K.LCTVATLR.E
172	470.7295	939.4444	939.4410	0.0035	0	(30)	0.46	1	U	K.DDNPNLPR.L
173	470.7301	939.4456	939.4410	0.0047	0	33	0.19	1	U	K.DDNPNLPR.L
174	480.7870	959.5595	959.5552	0.0042	0	49	0.01	1	U	K.FQNALLVR.Y
178	492.7498	983.4850	983.4811	0.0039	0	38	0.061	1	U	K.TYETTLEK.C
183	500.8075	999.6004	999.5964	0.0039	0	55	0.0019	1		K.QTALVELVK.H
185	500.8078	999.6011	999.5964	0.0047	0	(46)	0.017	1		K.QTALVELVK.H
189	507.3055	1012.5965	1012.5917	0.0049	0	14	23	2	U	K.LVAASQAALGL.-
190	509.2734	1016.5322	1016.5291	0.0031	0	32	0.61	1		K.SLHTLFGDK.L
216	537.7763	1073.5380	1073.5353	0.0027	1	(46)	0.021	1	U	K.LDELRDEGK.A
217	537.7770	1073.5394	1073.5353	0.0042	1	47	0.014	1	U	K.LDELRDEGK.A
218	537.7773	1073.5401	1073.5353	0.0048	1	(40)	0.079	1	U	K.LDELRDEGK.A
219	538.2543	1074.4941	1074.4917	0.0025	0	10	38	1		R.NECFLQHK.D
232	564.8553	1127.6961	1127.6914	0.0047	1	(49)	0.0039	1		K.KQTALVELVK.H
233	564.8555	1127.6964	1127.6914	0.0050	1	73	1.9e-005	1		K.KQTALVELVK.H
238	569.7539	1137.4932	1137.4907	0.0026	0	(44)	0.0073	1		K.CCTESLVNR.R
239	569.7546	1137.4946	1137.4907	0.0039	0	53	0.001	1		K.CCTESLVNR.R
241	571.3480	1140.6815	1140.6866	-0.0051	1	28	0.78	1	U	K.KLVAASQAALGL.-
242	571.3521	1140.6896	1140.6866	0.0029	1	(25)	1.2	1	U	K.KLVAASQAALGL.-
244	575.3136	1148.6127	1148.6077	0.0050	0	(84)	3.4e-006	1		K.LVNEVTEFAK.T
245	575.3137	1148.6129	1148.6077	0.0052	0	84	3.2e-006	1		K.LVNEVTEFAK.T
264	613.8093	1225.6041	1225.5979	0.0063	1	(39)	0.065	1	U	R.FKDLGEENFK.A
265	613.8096	1225.6047	1225.5979	0.0068	1	(76)	1.1e-005	1	U	R.FKDLGEENFK.A
266	409.5422	1225.6047	1225.5979	0.0068	1	(38)	0.081	1	U	R.FKDLGEENFK.A
267	409.5423	1225.6049	1225.5979	0.0070	1	(20)	5.2	1	U	R.FKDLGEENFK.A
268	613.8097	1225.6049	1225.5979	0.0070	1	77	1e-005	1	U	R.FKDLGEENFK.A
297	671.8237	1341.6327	1341.6275	0.0053	0	94	1.3e-007	1	U	K.AVMDDFAAFVEK.C
298	671.8244	1341.6342	1341.6275	0.0067	0	(89)	4.2e-007	1	U	K.AVMDDFAAFVEK.C
302	679.8208	1357.6271	1357.6224	0.0047	0	(86)	7.3e-007	1	U	K.AVMDDFAAFVEK.C + Oxidation (M)
303	679.8220	1357.6294	1357.6224	0.0070	0	(87)	5.4e-007	1	U	K.AVMDDFAAFVEK.C + Oxidation (M)
304	686.2890	1370.5635	1370.5595	0.0040	0	(59)	9.1e-005	1	U	K.AAFTECCQAADK.A
305	686.2894	1370.5643	1370.5595	0.0048	0	64	3.2e-005	1	U	K.AAFTECCQAADK.A
321	722.3276	1442.6407	1442.6347	0.0060	0	80	1.8e-006	1	U	K.YICENQDSISSK.L
324	489.9545	1466.8417	1466.8358	0.0060	1	43	0.015	1		R.RHPDYSVVLLLR.L
325	489.9547	1466.8422	1466.8358	0.0064	1	(35)	0.093	1		R.RHPDYSVVLLLR.L
334	516.2718	1545.7937	1545.7894	0.0042	1	(56)	0.0017	1	U	K.LKECCEKPLLEK.S

335	516.2720	1545.7941	1545.7894	0.0047	1	(57)	0.0012	1	U	K.LKECCEKPLLEK.S
336	516.2725	1545.7958	1545.7894	0.0063	1	67	0.00013	1	U	K.LKECCEKPLLEK.S
337	776.8047	1551.5948	1551.5905	0.0044	0	29	0.012	1	U	K.CCAAADPHECYAK.V
341	812.4005	1622.7865	1622.7803	0.0062	0	53	0.0023	1	U	K.DVFLGMFLYEYAR.R
342	547.3200	1638.9381	1638.9305	0.0076	1	(65)	7.8e-005	1		K.KVPQVSTPTLVEVSR.N
343	547.3201	1638.9385	1638.9305	0.0080	1	75	8.5e-006	1		K.KVPQVSTPTLVEVSR.N
349	829.3841	1656.7536	1656.7453	0.0082	0	75	7.9e-006	1	U	K.QNCELFEQLGEYK.F
357	572.2722	1713.7949	1713.7893	0.0056	1	(18)	3.8	1		K.QEPERNECFLQHK.D
358	572.2732	1713.7979	1713.7893	0.0086	1	28	0.44	1		K.QEPERNECFLQHK.D
379	637.6515	1909.9327	1909.9244	0.0083	0	(51)	0.0027	1	U	R.RPCFSALEVDETYVPK.E
380	955.9746	1909.9347	1909.9244	0.0103	0	(22)	2.6	1	U	R.RPCFSALEVDETYVPK.E
381	637.6522	1909.9347	1909.9244	0.0103	0	62	0.00023	1	U	R.RPCFSALEVDETYVPK.E
384	666.3180	1995.9321	1995.9221	0.0100	1	(11)	19	1	U	R.NECFLQHKDDNPNLPR.L
385	666.3186	1995.9340	1995.9221	0.0119	1	33	0.14	1	U	R.NECFLQHKDDNPNLPR.L
389	682.3727	2044.0962	2044.0881	0.0081	0	(56)	0.001	1	U	K.VFDEFKPLVEEPQNLIK.Q
390	1023.0563	2044.0981	2044.0881	0.0100	0	(21)	3	1	U	K.VFDEFKPLVEEPQNLIK.Q
391	682.3733	2044.0981	2044.0881	0.0100	0	56	0.00092	1	U	K.VFDEFKPLVEEPQNLIK.Q
392	682.3736	2044.0989	2044.0881	0.0108	0	(50)	0.0036	1	U	K.VFDEFKPLVEEPQNLIK.Q
408	754.0163	2259.0270	2259.0154	0.0116	0	36	0.034	1	U	K.EFNAETFTFHADICTLSEK.E
434	647.0384	2584.1245	2584.1105	0.0141	1	7	12	1	U	K.VHTECCHGDLLECADDRADLAK.Y
446	659.8155	2635.2330	2635.2197	0.0133	2	(27)	0.36	1	U	K.QEPERNECFLQHKDDNPNLPR.L
447	659.8160	2635.2349	2635.2197	0.0151	2	(21)	1.3	1	U	K.QEPERNECFLQHKDDNPNLPR.L
448	659.8163	2635.2361	2635.2197	0.0163	2	31	0.14	1	U	K.QEPERNECFLQHKDDNPNLPR.L
449	663.3266	2649.2774	2649.2567	0.0207	0	13	13	1	U	R.LVRPEVDVMCTAFHDNEETFLK.K
464	699.3475	2793.3608	2793.3466	0.0142	1	39	0.033	1	U	R.LVRPEVDVMCTAFHDNEETFLKK.Y + Oxidation (M)
465	559.6813	2793.3700	2793.3466	0.0234	1	(29)	0.33	1	U	R.LVRPEVDVMCTAFHDNEETFLKK.Y + Oxidation (M)
466	699.3498	2793.3700	2793.3466	0.0234	1	(32)	0.2	1	U	R.LVRPEVDVMCTAFHDNEETFLKK.Y + Oxidation (M)
473	992.1229	2973.3468	2973.3372	0.0096	0	(72)	5.6e-006	1	U	K.SHCIAEVENDEMPADLPSLAADFVESK.D
474	992.1236	2973.3490	2973.3372	0.0118	0	74	3.6e-006	1	U	K.SHCIAEVENDEMPADLPSLAADFVESK.D
475	992.1281	2973.3625	2973.3372	0.0253	0	(69)	1.2e-005	1	U	K.SHCIAEVENDEMPADLPSLAADFVESK.D
476	992.1318	2973.3734	2973.3372	0.0362	0	(54)	0.00047	1	U	K.SHCIAEVENDEMPADLPSLAADFVESK.D
477	997.4566	2989.3479	2989.3321	0.0158	0	(53)	0.00039	1	U	K.SHCIAEVENDEMPADLPSLAADFVESK.D + Oxidation (M)
478	997.4571	2989.3494	2989.3321	0.0173	0	(59)	9.9e-005	1	U	K.SHCIAEVENDEMPADLPSLAADFVESK.D + Oxidation (M)

Proteins matching the same set of peptides:

ALBU_PONAB **Mass:** 71317 **Score:** 1513 **Matches:** 84(44) **Sequences:** 38(23)

Serum albumin OS=Pongo abelii GN=ALB PE=2 SV=1

---

2. F13A_HUMAN **Mass:** 83728 **Score:** 1332 **Matches:** 53(35) **Sequences:** 26(20) **emPAI:** 1.97

Coagulation factor XIII A chain OS=Homo sapiens GN=F13A1 PE=1 SV=4

Check to include this hit in error tolerant search or archive report

Query	Observed	Mr(expt)	Mr(calc)	Delta	Miss	Score	Expect	Rank	Unique	Peptide
137	418.2388	834.4631	834.4599	0.0032	0	33	0.4	1	U	R.VEYVIGR.Y
138	418.2392	834.4638	834.4599	0.0039	0	(31)	0.63	1	U	R.VEYVIGR.Y
147	430.2497	858.4848	858.4811	0.0038	0	49	0.016	1	U	R.LSIQSSPK.C
150	435.7816	869.5486	869.5083	0.0403	1	15	13	1	U	R.GNPIKVSR.V
156	439.2148	876.4151	876.4123	0.0028	0	48	0.012	1	U	R.AQMDLSGR.G
157	439.2148	876.4151	876.4123	0.0028	0	(32)	0.4	1	U	R.AQMDLSGR.G
158	439.2154	876.4162	876.4123	0.0039	0	(22)	3.8	1	U	R.AQMDLSGR.G
192	515.7731	1029.5316	1029.5277	0.0039	0	(58)	0.0012	1	U	R.CGPASVQAIK.H
193	515.7731	1029.5316	1029.5277	0.0039	0	65	0.00027	1	U	R.CGPASVQAIK.H
226	558.8090	1115.6035	1115.6009	0.0027	0	(54)	0.0036	1	U	K.KPLNTEGVMK.S
227	558.8092	1115.6038	1115.6009	0.0029	0	(39)	0.11	1	U	K.KPLNTEGVMK.S
228	558.8095	1115.6044	1115.6009	0.0036	0	59	0.0012	1	U	K.KPLNTEGVMK.S
234	566.8065	1131.5985	1131.5958	0.0027	0	(38)	0.13	1	U	K.KPLNTEGVMK.S + Oxidation (M)
235	566.8069	1131.5993	1131.5958	0.0035	0	(56)	0.0021	1	U	K.KPLNTEGVMK.S + Oxidation (M)
236	566.8070	1131.5994	1131.5958	0.0036	0	(58)	0.0013	1	U	K.KPLNTEGVMK.S + Oxidation (M)
237	566.8074	1131.6002	1131.5958	0.0044	0	(38)	0.13	1	U	K.KPLNTEGVMK.S + Oxidation (M)
250	598.3061	1194.5976	1194.5914	0.0062	0	47	0.013	1	U	K.LIASMSSDSLR.H + Oxidation (M)
279	640.8529	1279.6912	1279.6846	0.0066	0	(83)	2.9e-006	1	U	R.LALETALMYGAK.K
282	648.8496	1295.6847	1295.6795	0.0052	0	86	1.2e-006	1	U	R.LALETALMYGAK.K + Oxidation (M)
290	663.9179	1325.8211	1325.8170	0.0042	0	(22)	0.93	1	U	K.STVLTIPEIIIK.V
291	663.9180	1325.8214	1325.8170	0.0044	0	40	0.016	1	U	K.STVLTIPEIIIK.V
322	728.8785	1455.7425	1455.7470	-0.0045	0	14	20	1	U	R.HVYGELDVQIQR.R
331	763.3984	1524.7822	1524.7712	0.0110	0	68	8.1e-005	1	U	K.ETFDVTLEPLSFK.K
344	822.8776	1643.7407	1643.7171	0.0236	0	52	0.0013	1	U	K.QIGGDGMMDITDTYK.F
345	827.4451	1652.8757	1652.8661	0.0095	1	43	0.025	1	U	K.ETFDVTLEPLSFKK.E
351	564.6190	1690.8352	1690.8275	0.0077	0	(35)	0.15	1	U	K.DGTHVVENVDATHIGK.L
352	564.6193	1690.8362	1690.8275	0.0087	0	63	0.00026	1	U	K.DGTHVVENVDATHIGK.L
367	883.9212	1765.8279	1765.8193	0.0086	0	77	5.6e-006	1	U	R.SNVDMDFEVENAVLGK.D
372	607.3173	1818.9300	1818.9224	0.0075	1	(58)	0.00091	1	U	K.KDGTHVVENVDATHIGK.L
373	607.3175	1818.9308	1818.9224	0.0084	1	65	0.00018	1	U	K.KDGTHVVENVDATHIGK.L
374	607.9892	1820.9457	1820.9356	0.0101	0	30	0.51	1	U	R.NVWVHLDGPGVTRPMK.K + Oxidation (M)
418	1165.0865	2328.1584	2328.1525	0.0059	0	(101)	2.6e-008	1	U	R.EEYVLNDIGVIFYGEVNDIK.T
419	1165.0872	2328.1598	2328.1525	0.0073	0	124	1.6e-010	1	U	R.EEYVLNDIGVIFYGEVNDIK.T
424	805.0628	2412.1666	2412.1631	0.0036	1	(39)	0.034	1	U	K.FQEGQEEERLALETALMYGAK.K
425	805.0633	2412.1681	2412.1631	0.0050	1	45	0.01	1	U	K.FQEGQEEERLALETALMYGAK.K
426	810.3976	2428.1709	2428.1580	0.0129	1	(28)	0.45	1	U	K.FQEGQEEERLALETALMYGAK.K + Oxidation (M)
427	810.3996	2428.1771	2428.1580	0.0191	1	(37)	0.062	1	U	K.FQEGQEEERLALETALMYGAK.K + Oxidation (M)
430	1278.0696	2554.1247	2554.1145	0.0103	0	(22)	0.47	1	U	R.SWSYGQFEDGILDTCLYVMDR.A
431	1278.0708	2554.1270	2554.1145	0.0125	0	24	0.3	1	U	R.SWSYGQFEDGILDTCLYVMDR.A
435	868.1105	2601.3096	2601.3035	0.0062	0	(43)	0.018	1	U	R.AVPPNNSNAAEDDLPTVELQGVVPR.G
438	868.1127	2601.3162	2601.3035	0.0128	0	57	0.00077	1	U	R.AVPPNNSNAAEDDLPTVELQGVVPR.G
440	868.1139	2601.3200	2601.3035	0.0165	0	(56)	0.00092	1	U	R.AVPPNNSNAAEDDLPTVELQGVVPR.G
441	872.1087	2613.3043	2613.2962	0.0081	1	71	2.4e-005	1	U	K.EREEYVLNDIGVIFYGEVNDIK.T
442	872.1092	2613.3057	2613.2962	0.0095	1	(68)	5.6e-005	1	U	K.EREEYVLNDIGVIFYGEVNDIK.T
443	872.1095	2613.3068	2613.2962	0.0105	1	(66)	8e-005	1	U	K.EREEYVLNDIGVIFYGEVNDIK.T
444	872.1118	2613.3135	2613.2962	0.0172	1	(46)	0.0087	1	U	K.EREEYVLNDIGVIFYGEVNDIK.T
458	681.3407	2721.3335	2721.3194	0.0141	0	(24)	1.2	1	U	R.EIRPNSTVQWEEVCRPWVSGHR.K
459	681.3422	2721.3396	2721.3194	0.0202	0	25	0.97	1	U	R.EIRPNSTVQWEEVCRPWVSGHR.K
461	920.1480	2757.4223	2757.4046	0.0177	1	64	0.00012	1	U	R.RAVPPNNSNAAEDDLPTVELQGVVPR.G
482	1021.1225	3060.3458	3060.3335	0.0123	0	44	0.0016	1	U	R.NPETDTYILFNPWCEDDAVYLDNEK.E
508	1138.5294	3412.5664	3412.5517	0.0147	0	117	1.5e-010	1	U	R.IVTNYFSAHDNDANLQMDIFLEEDGNVNSK.L
509	1138.5300	3412.5682	3412.5517	0.0164	0	(88)	1.3e-007	1	U	R.IVTNYFSAHDNDANLQMDIFLEEDGNVNSK.L
511	1143.8612	3428.5618	3428.5467	0.0152	0	(57)	0.00015	1	U	R.IVTNYFSAHDNDANLQMDIFLEEDGNVNSK.L + Oxidation (M)

---

3. K2C1_HUMAN **Mass:** 66170 **Score:** 769 **Matches:** 30(19) **Sequences:** 18(12) **emPAI:** 1.26

Keratin, type II cytoskeletal 1 OS=Homo sapiens GN=KRT1 PE=1 SV=6

Check to include this hit in error tolerant search or archive report

Query	Observed	Mr(expt)	Mr(calc)	Delta	Miss	Score	Expect	Rank	Unique	Peptide
151	437.7549	873.4952	873.4920	0.0033	0	45	0.035	1	U	R.SLVNLGGSK.S
175	487.2711	972.5276	972.5240	0.0036	0	(38)	0.16	1		K.IEISELNR.V
176	487.2713	972.5281	972.5240	0.0041	0	44	0.04	1		K.IEISELNR.V
181	500.2286	998.4426	998.4379	0.0048	0	14	8.9	1	U	K.DVDGAYMTK.V
196	517.2642	1032.5139	1032.5087	0.0052	0	73	4.3e-005	1	U	R.TLLEGEESR.M
211	533.2657	1064.5169	1064.5138	0.0031	0	32	0.4	1	U	K.AQYEDIAQK.S
212	533.2660	1064.5174	1064.5138	0.0036	0	(28)	0.83	1	U	K.AQYEDIAQK.S
214	537.3020	1072.5894	1072.5876	0.0017	1	(25)	3.2	2	U	R.LRSEIDNVK.K
215	537.3020	1072.5895	1072.5876	0.0018	1	34	0.36	1	U	R.LRSEIDNVK.K
240	571.2658	1140.5171	1140.5121	0.0050	0	34	0.11	1		R.DYQELMNTK.L
247	590.3058	1178.5971	1178.5931	0.0039	0	(53)	0.0037	1		K.YEELQITAGR.H

248	590.3065	1178.5984	1178.5931	0.0052	0	77	1.3e-005	1		K.YEELQITAGR.H
253	401.2365	1200.6876	1200.6826	0.0050	2	34	0.24	1	U	R.LRSEIDNVKK.Q
254	401.2365	1200.6876	1200.6826	0.0050	2	(27)	1.3	1	U	R.LRSEIDNVKK.Q
277	639.3615	1276.7085	1276.7027	0.0058	0	(65)	0.00017	1	U	K.LALDLEIATYR.T
278	639.3617	1276.7089	1276.7027	0.0062	0	65	0.00016	1	U	K.LALDLEIATYR.T
283	650.7708	1299.5271	1299.5224	0.0048	0	44	0.0024	1	U	K.NMQDMVEDYR.N
286	436.8912	1307.6517	1307.6469	0.0048	2	54	0.0025	1		R.NKYEDEINKR.T
294	670.8405	1339.6664	1339.6619	0.0045	1	64	0.00019	1	U	K.SKAEAESLYQSK.Y
295	670.8406	1339.6667	1339.6619	0.0048	1	(59)	0.00058	1	U	K.SKAEAESLYQSK.Y
296	670.8408	1339.6671	1339.6619	0.0052	1	(60)	0.00056	1	U	K.SKAEAESLYQSK.Y
300	679.3534	1356.6923	1356.6885	0.0039	0	50	0.0062	1	U	K.LNDLEDALQQAK.E
309	697.3728	1392.7310	1392.7249	0.0062	1	78	9.7e-006	1	U	R.TNAENEFVTIKK.D
310	697.3730	1392.7315	1392.7249	0.0066	1	(72)	3.7e-005	1	U	R.TNAENEFVTIKK.D
326	738.3997	1474.7848	1474.7780	0.0068	0	117	1.2e-009	1		R.FLEQQNQVLQTK.W
327	738.3999	1474.7851	1474.7780	0.0072	0	(109)	7.8e-009	1		R.FLEQQNQVLQTK.W
340	800.4232	1598.8318	1598.8264	0.0055	1	24	2.1	1	U	K.NKLNDLEDALQQAK.E
359	858.9325	1715.8505	1715.8438	0.0067	0	(92)	3.4e-007	1	U	K.QISNLQQSISDAEQR.G
360	858.9333	1715.8520	1715.8438	0.0082	0	(22)	3.2	1	U	K.QISNLQQSISDAEQR.G
361	858.9334	1715.8523	1715.8438	0.0085	0	103	2.4e-008	1	U	K.QISNLQQSISDAEQR.G

4. TRYP_PIG **Mass:** 25078 **Score:** 683 **Matches:** 22(18) **Sequences:** 7(6) **emPAI:** 2.76

Trypsin OS=Sus scrofa PE=1 SV=1

Check to include this hit in error tolerant search or archive report

Query	Observed	Mr(expt)	Mr(calc)	Delta	Miss	Score	Expect	Rank	Unique	Peptide
140	421.7601	841.5056	841.5022	0.0034	0	54	0.0029	1	U	R.VATVSLPR.S
142	421.7601	841.5057	841.5022	0.0035	0	(54)	0.003	1	U	R.VATVSLPR.S
143	421.7603	841.5060	841.5022	0.0039	0	(52)	0.0046	1	U	R.VATVSLPR.S
199	523.2871	1044.5596	1044.5564	0.0033	0	(52)	0.006	1	U	K.LSSPATLNSR.V
200	523.2874	1044.5602	1044.5564	0.0038	0	(50)	0.0096	1	U	K.LSSPATLNSR.V
201	523.2879	1044.5613	1044.5564	0.0050	0	53	0.0053	1	U	K.LSSPATLNSR.V
207	532.2588	1062.5031	1062.5016	0.0016	0	(47)	0.011	1	U	K.APVLSDSSCK.S
208	532.2600	1062.5055	1062.5016	0.0040	0	(94)	2.4e-007	1	U	K.APVLSDSSCK.S
209	532.2605	1062.5064	1062.5016	0.0048	0	94	2.2e-007	1	U	K.APVLSDSSCK.S
400	1106.0608	2210.1070	2210.0967	0.0102	0	(7)	76	1	U	R.LGEHNIDVLEGNEQFINAAK.I
401	737.7096	2210.1070	2210.0967	0.0102	0	(93)	1.9e-007	1	U	R.LGEHNIDVLEGNEQFINAAK.I
403	737.7099	2210.1078	2210.0967	0.0110	0	(79)	4.7e-006	1	U	R.LGEHNIDVLEGNEQFINAAK.I
404	737.7102	2210.1086	2210.0967	0.0119	0	104	1.7e-008	1	U	R.LGEHNIDVLEGNEQFINAAK.I
411	761.7337	2282.1792	2282.1729	0.0063	0	(60)	0.00035	1	U	K.IITHPNFNGNTLDNDIMLIK.L
412	761.7352	2282.1838	2282.1729	0.0109	0	75	1.2e-005	1	U	K.IITHPNFNGNTLDNDIMLIK.L
414	761.7354	2282.1843	2282.1729	0.0115	0	(63)	0.0002	1	U	K.IITHPNFNGNTLDNDIMLIK.L
416	767.0684	2298.1833	2298.1678	0.0155	0	(44)	0.015	1	U	K.IITHPNFNGNTLDNDIMLIK.L + Oxidation (M)
456	903.1484	2706.4235	2706.4089	0.0146	1	33	0.12	1	U	R.IQVRLGEHNIDVLEGNEQFINAAK.I
457	677.6159	2706.4343	2706.4089	0.0254	1	(22)	1.3	1	U	R.IQVRLGEHNIDVLEGNEQFINAAK.I
499	828.1913	3308.7359	3308.7187	0.0173	1	70	1.7e-005	1	U	K.IITHPNFNGNTLDNDIMLIKLSSPATLNSR.V
500	828.1915	3308.7370	3308.7187	0.0183	1	(54)	0.00063	1	U	K.IITHPNFNGNTLDNDIMLIKLSSPATLNSR.V
502	832.1932	3324.7438	3324.7136	0.0302	1	(23)	0.83	1	U	K.IITHPNFNGNTLDNDIMLIKLSSPATLNSR.V + Oxidation (M)

5. F13B_HUMAN **Mass:** 77742 **Score:** 456 **Matches:** 26(14) **Sequences:** 18(11) **emPAI:** 0.92

Coagulation factor XIII B chain OS=Homo sapiens GN=F13B PE=1 SV=3

Check to include this hit in error tolerant search or archive report

Query	Observed	Mr(expt)	Mr(calc)	Delta	Miss	Score	Expect	Rank	Unique	Peptide
134	405.2033	808.3921	808.3902	0.0020	0	19	7.6	1	U	K.YPLCTR.K
148	431.2527	860.4909	860.4868	0.0041	1	26	2.3	1	U	R.GQLKYPR.C
222	552.2849	1102.5552	1102.5515	0.0037	0	(37)	0.15	1	U	K.GMCTSPPLIK.H
229	560.2814	1118.5483	1118.5464	0.0019	0	38	0.098	1	U	K.GMCTSPPLIK.H + Oxidation (M)
251	598.8039	1195.5931	1195.5913	0.0018	0	60	0.00053	1	U	R.IAQYYYTFK.S
252	598.8057	1195.5968	1195.5913	0.0055	0	(59)	0.00075	1	U	R.IAQYYYTFK.S
259	610.3310	1218.6473	1218.6431	0.0043	0	38	0.12	1	U	R.CPPPPLPINSK.I
261	611.8189	1221.6232	1221.6176	0.0056	1	13	38	1	U	R.GEVKYPLCTR.K
288	439.5734	1315.6983	1315.6925	0.0059	0	62	0.00047	1	U	R.LIENGYFHPVK.Q
289	658.8564	1315.6983	1315.6925	0.0059	0	(44)	0.031	1	U	R.LIENGYFHPVK.Q
77	658.8572	1315.6997	1315.6925	0.0073	0	(33)	0.39	1	U	R.LIENGYFHPVK.Q
312	468.8776	1403.6111	1403.6041	0.0070	0	37	0.027	1	U	R.CFDHHFLEGSR.E
313	468.8778	1403.6116	1403.6041	0.0075	0	(31)	0.11	1	U	R.CFDHHFLEGSR.E
314	708.3736	1414.7326	1414.7279	0.0047	0	95	2.2e-007	1	U	K.VLHGDLIDFVCK.Q
315	472.5848	1414.7326	1414.7279	0.0047	0	(30)	0.6	1	U	K.VLHGDLIDFVCK.Q
317	708.3743	1414.7340	1414.7279	0.0062	0	(22)	4.1	1	U	K.VLHGDLIDFVCK.Q
339	532.2597	1593.7573	1593.7497	0.0076	1	30	0.45	1	U	K.IYYNGDKVTYACK.S
353	566.2810	1695.8211	1695.8138	0.0074	0	46	0.012	1	U	K.CTKPDLSNGYISDVK.L
356	854.9063	1707.7979	1707.7927	0.0053	0	53	0.0015	1	U	K.LSFFCLAGYTTESGR.Q
362	574.2765	1719.8076	1719.7999	0.0078	0	57	0.00062	1	U	K.SGYLLHGSNEITCNR.G
368	884.9621	1767.9097	1767.9043	0.0054	0	53	0.0025	1	U	R.GDTYPAELYITGSILR.M
388	681.0069	2039.9989	2039.9874	0.0115	1	12	24	1	U	K.KTEEVECLTYGWSLTPK.C
394	695.0030	2081.9872	2081.9840	0.0032	0	(57)	0.00059	1	U	K.VACEEPPFIENGAANLHSK.I
395	695.0050	2081.9933	2081.9840	0.0093	0	69	3.9e-005	1	U	K.VACEEPPFIENGAANLHSK.I
455	899.7388	2696.1945	2696.1807	0.0139	1	59	9.4e-005	1	U	K.TTGKDEEVVQCLSDGWSSQPTCR.K
462	921.1190	2760.3351	2760.3541	-0.0190	0	40	0.03	1	U	K.HPPVVMNGAVADGILASYATGSSVEYR.C

6. K1C9_HUMAN **Mass:** 62255 **Score:** 272 **Matches:** 14(8) **Sequences:** 10(5) **emPAI:** 0.63

Keratin, type I cytoskeletal 9 OS=Homo sapiens GN=KRT9 PE=1 SV=3

Check to include this hit in error tolerant search or archive report

Query	Observed	Mr(expt)	Mr(calc)	Delta	Miss	Score	Expect	Rank	Unique	Peptide
164	449.2116	896.4087	896.4062	0.0025	0	42	0.02	1	U	R.MTLDDFR.I
210	533.2550	1064.4954	1064.4920	0.0033	0	38	0.069	1	U	K.STMQELNSR.L
230	561.2981	1120.5817	1120.5764	0.0053	0	38	0.12	1	U	R.QEYEQLIAK.N
246	579.3009	1156.5872	1156.5836	0.0036	0	64	0.00025	1	U	R.QGVDADINGLR.Q
249	595.8031	1189.5917	1189.6013	-0.0096	0	26	1.7	1	U	R.QVLDNLTMEK.S
255	603.8075	1205.6005	1205.5962	0.0043	0	(23)	3.3	1	U	R.QVLDNLTMEK.S + Oxidation (M)
269	616.8045	1231.5945	1231.5906	0.0040	0	71	4.1e-005	1	U	R.SGGGGGGGLGSGGSIR.S
270	616.8047	1231.5949	1231.5906	0.0044	0	(55)	0.0018	1	U	R.SGGGGGGGLGSGGSIR.S
273	618.2699	1234.5253	1234.5215	0.0039	0	66	3e-005	1	U	R.FSSSSGYGGGSSR.V
274	618.2700	1234.5255	1234.5215	0.0040	0	(58)	0.0002	1	U	R.FSSSSGYGGGSSR.V
275	618.2701	1234.5257	1234.5215	0.0043	0	(61)	0.00011	1	U	R.FSSSSGYGGGSSR.V
285	654.3451	1306.6756	1306.6703	0.0053	1	28	0.97	1	U	R.IKFEMEQNLR.Q
428	837.3824	2509.1254	2509.1245	0.0009	0	43	0.0047	1	U	K.EIETYHNLLEGGQEDFESSGAGK.I
496	1088.8476	3263.5211	3263.5066	0.0145	0	6	28	1	U	K.DIENQYETQITQIEHEVSSSGQEVQSSAK.E

7. K1C10_HUMAN **Mass:** 59020 **Score:** 249 **Matches:** 15(8) **Sequences:** 11(7) **emPAI:** 0.49

Keratin, type I cytoskeletal 10 OS=Homo sapiens GN=KRT10 PE=1 SV=6

Check to include this hit in error tolerant search or archive report

Query	Observed	Mr(expt)	Mr(calc)	Delta	Miss	Score	Expect	Rank	Unique	Peptide
132	404.2051	806.3957	806.3923	0.0034	0	(29)	1.2	1		R.LAADDFR.L
133	404.2052	806.3959	806.3923	0.0037	0	50	0.0087	1		R.LAADDFR.L
180	498.2652	994.5158	994.5123	0.0034	1	21	4.9	1		K.IKEWYEK.H
187	502.2824	1002.5503	1002.5458	0.0045	1	20	8.3	2		K.SEITELRR.N

188	502.2844	1002.5542	1002.5458	0.0084	1	(15)	31	1		K.SEITELRR.N
271	617.8459	1233.6772	1233.6717	0.0055	1	(49)	0.0088	1		R.LKYENEVALR.Q
272	617.8461	1233.6776	1233.6717	0.0059	1	65	0.00021	1		R.LKYENEVALR.Q
301	679.3660	1356.7175	1356.7110	0.0066	1	7	1.3e+002	1		R.QSVEADINGLRR.V
307	691.3322	1380.6499	1380.6408	0.0090	0	75	1.3e-005	1		R.ALEESNYELEGK.I
308	695.8458	1389.6770	1389.6736	0.0035	0	55	0.0015	1	U	K.QSLEASLAETEGR.Y
329	747.3725	1492.7305	1492.7270	0.0035	1	45	0.015	1	U	R.SQYEQLAEQNRK.D
330	498.5847	1492.7322	1492.7270	0.0053	1	(13)	26	1	U	R.SQYEQLAEQNRK.D
355	854.3927	1706.7708	1706.7649	0.0059	0	77	4.2e-006	1	U	K.GSLGGGFSSGGFSGGSFSR.G
371	899.0132	1796.0119	1796.0043	0.0076	0	58	0.00043	1	U	R.NVQALEIELQSQLALK.Q
405	738.0352	2211.0838	2211.0906	-0.0068	1	0	3.2e+002	1	U	K.SKELTTEIDNNIEQISSYK.S

---

8. K22E_HUMAN **Mass:** 65678 **Score:** 234 **Matches:** 6(5) **Sequences:** 4(4) **emPAI:** 0.23
Keratin, type II cytoskeletal 2 epidermal OS=Homo sapiens GN=KRT2 PE=1 SV=2
Check to include this hit in error tolerant search or archive report

Query	Observed	Mr(expt)	Mr(calc)	Delta	Miss	Score	Expect	Rank	Unique	Peptide
175	487.2711	972.5276	972.5240	0.0036	0	(38)	0.16	1		K.IEISELNR.V
176	487.2713	972.5281	972.5240	0.0041	0	44	0.04	1		K.IEISELNR.V
292	665.3692	1328.7239	1328.7187	0.0052	0	45	0.019	1		R.NLDLDSIIAEVK.A
323	730.9061	1459.7977	1459.7922	0.0054	0	69	8.5e-005	1	U	K.VDLLNQEIEFLK.V
326	738.3997	1474.7848	1474.7780	0.0068	0	117	1.2e-009	1		R.FLEQQNQVLQTK.W
327	738.3999	1474.7851	1474.7780	0.0072	0	(109)	7.8e-009	1		R.FLEQQNQVLQTK.W

---

9. A1BG_HUMAN **Mass:** 54809 **Score:** 82 **Matches:** 3(2) **Sequences:** 3(2) **emPAI:** 0.13
Alpha-1B-glycoprotein OS=Homo sapiens GN=A1BG PE=1 SV=3
Check to include this hit in error tolerant search or archive report

Query	Observed	Mr(expt)	Mr(calc)	Delta	Miss	Score	Expect	Rank	Unique	Peptide
149	435.7701	869.5256	869.5222	0.0034	0	61	0.00042	1	U	K.LLELTGPK.S
276	619.3284	1236.6423	1236.6390	0.0033	0	9	88	1	U	R.LETPDFQLFK.N
306	686.8540	1371.6935	1371.6895	0.0040	0	57	0.0012	1	U	K.HQFLLTGDTQGR.Y

---

10. K1C14_HUMAN **Mass:** 51872 **Score:** 67 **Matches:** 4(3) **Sequences:** 3(3) **emPAI:** 0.21
Keratin, type I cytoskeletal 14 OS=Homo sapiens GN=KRT14 PE=1 SV=4
Check to include this hit in error tolerant search or archive report

Query	Observed	Mr(expt)	Mr(calc)	Delta	Miss	Score	Expect	Rank	Unique	Peptide
132	404.2051	806.3957	806.3923	0.0034	0	(29)	1.2	1		R.LAADDFR.T
133	404.2052	806.3959	806.3923	0.0037	0	50	0.0087	1		R.LAADDFR.T
191	515.3011	1028.5876	1028.5866	0.0010	0	53	0.0048	1		R.VLDELTLAR.A
284	651.3325	1300.6504	1300.6510	-0.0006	0	41	0.049	1		R.ALEEANADLEVK.I

Proteins matching the same set of peptides:
K1C15_HUMAN **Mass:** 49395 **Score:** 67 **Matches:** 4(3) **Sequences:** 3(3)
Keratin, type I cytoskeletal 15 OS=Homo sapiens GN=KRT15 PE=1 SV=2
K1C16_HUMAN **Mass:** 51578 **Score:** 67 **Matches:** 4(3) **Sequences:** 3(3)
Keratin, type I cytoskeletal 16 OS=Homo sapiens GN=KRT16 PE=1 SV=4
K1C19_POTTR **Mass:** 43860 **Score:** 67 **Matches:** 4(3) **Sequences:** 3(3)
Keratin, type I cytoskeletal 19 OS=Potorous tridactylus GN=KRT19 PE=2 SV=1
K1C42_MOUSE **Mass:** 50444 **Score:** 67 **Matches:** 4(3) **Sequences:** 3(3)
Keratin, type I cytoskeletal 42 OS=Mus musculus GN=Krt42 PE=1 SV=1

---

11. HEMO_HUMAN **Mass:** 52385 **Score:** 64 **Matches:** 3(1) **Sequences:** 3(1) **emPAI:** 0.14
Hemopexin OS=Homo sapiens GN=HPX PE=1 SV=2
Check to include this hit in error tolerant search or archive report

Query	Observed	Mr(expt)	Mr(calc)	Delta	Miss	Score	Expect	Rank	Unique	Peptide
135	407.2244	812.4343	812.4327	0.0016	0	5	1.5e+002	1	U	R.LHIMAGR.R + Oxidation (M)
243	571.8226	1141.6307	1141.6244	0.0063	0	32	0.39	1	U	R.QGHNSVFLIK.G
260	610.8088	1219.6030	1219.5986	0.0045	0	58	0.001	1	U	K.NFPSPVDAAFR.Q

Proteins matching the same set of peptides:
HEMO_PONAB **Mass:** 52323 **Score:** 64 **Matches:** 3(1) **Sequences:** 3(1)
Hemopexin OS=Pongo abelii GN=HPX PE=2 SV=1

---

12. K1M1_SHEEP **Mass:** 48070 **Score:** 55 **Matches:** 6(3) **Sequences:** 5(3) **emPAI:** 0.23
Keratin, type I microfibrillar 48 kDa, component 8C-1 OS=Ovis aries PE=1 SV=2
Check to include this hit in error tolerant search or archive report

Query	Observed	Mr(expt)	Mr(calc)	Delta	Miss	Score	Expect	Rank	Unique	Peptide
132	404.2051	806.3957	806.3923	0.0034	0	(29)	1.2	1		K.LAADDFR.T
133	404.2052	806.3959	806.3923	0.0037	0	50	0.0087	1		K.LAADDFR.T
182	500.2943	998.5741	998.5760	-0.0019	0	41	0.052	1	U	R.LVVQIDNAK.L
223	555.2689	1108.5232	1108.5189	0.0043	0	24	1.9	2	U	R.DVEEWYIR.Q
328	739.8556	1477.6966	1477.6896	0.0070	0	38	0.056	1	U	R.DSLENTLTETEAR.Y
377	917.9986	1833.9827	1833.9697	0.0130	0	16	11	1	U	R.TVNALEVELQAQHNLR.D

---

13. K2M3_SHEEP **Mass:** 56418 **Score:** 47 **Matches:** 2(0) **Sequences:** 2(0) **emPAI:** 0.13
Keratin, type II microfibrillar, component 5 OS=Ovis aries PE=1 SV=1
Check to include this hit in error tolerant search or archive report

Query	Observed	Mr(expt)	Mr(calc)	Delta	Miss	Score	Expect	Rank	Unique	Peptide
177	490.2667	978.5188	978.5134	0.0053	0	39	0.099	1	U	R.ISPGYSVTR.T
197	519.7478	1037.4811	1037.4778	0.0033	0	32	0.3	1	U	K.AQYDDIASR.S

---

14. CO4A_HUMAN **Mass:** 194247 **Score:** 39 **Matches:** 1(0) **Sequences:** 1(0) **emPAI:** 0.02
Complement C4-A OS=Homo sapiens GN=C4A PE=1 SV=1
Check to include this hit in error tolerant search or archive report

Query	Observed	Mr(expt)	Mr(calc)	Delta	Miss	Score	Expect	Rank	Unique	Peptide
333	771.4181	1540.8216	1540.8097	0.0119	0	39	0.067	1	U	K.VLSLAQEQVGGSPEK.L

Proteins matching the same set of peptides:

CO4B_HUMAN **Mass:** 194212 **Score:** 39 **Matches:** 1(0) **Sequences:** 1(0)
Complement C4-B OS=Homo sapiens GN=C4B PE=1 SV=1

Peptide matches not assigned to protein hits: (no details means no match)

Query	Observed	Mr(expt)	Mr(calc)	Delta	Miss	Score	Expect	Rank	Unique	Peptide
136	416.7495	831.4844	831.4814	0.0030	0	35	0.31	1		SLSLGSLR
214	537.3020	1072.5894	1072.6128	-0.0234	0	28	1.7	1		AIITSLDNVK
223	555.2689	1108.5232	1108.5400	-0.0169	0	26	1	1		ITEESVEFR
187	502.2824	1002.5503	1002.5709	-0.0206	2	22	6.6	1		SEALAKGLKS
202	523.7810	1045.5474	1045.5655	-0.0181	0	18	13	1		IIGTATDEVK
153	438.2498	874.4851	874.4912	-0.0061	0	18	18	1		LNGSLFPK
81	673.3806	672.3733	672.3919	-0.0185	0	15	42	1		GLSVAAR
145	846.4452	845.4379	845.4243	0.0136	0	15	38	1		VTDVNGNK
204	523.7819	1045.5493	1045.5590	-0.0097	0	14	32	1		LPMVTNLSR + Oxidation (M)
189	507.3055	1012.5965	1012.6029	-0.0064	1	14	23	1		EKAGALVAVR
144	846.4450	845.4378	845.4495	-0.0117	0	13	60	1		DTVVEVGK
415	762.0643	2283.1710	2283.1470	0.0240	0	12	21	1		LQAIEPSGNYHILHAYCGIK
370	887.9568	1773.8990	1773.8857	0.0134	2	12	35	1		GRVEELGEEIGRESSK
469	480.7791	2878.6308	2878.5837	0.0471	2	12	3.4	1		EIITSIRIGTYISVKEMDNISILVR + Oxidation (M)
366	883.8985	1765.7824	1765.8226	-0.0402	1	11	10	1		KMEQESIDQLCELK + Oxidation (M)
281	645.8818	1289.7491	1289.7456	0.0035	2	11	32	1		IFGTKNDRIVK
203	523.7816	1045.5487	1045.5404	0.0083	1	11	79	1		LREGTLDDK
316	472.5853	1414.7340	1414.7542	-0.0202	1	10	60	1		GDNRVHGHVVAVR
410	755.3486	2263.0240	2263.0474	-0.0233	1	10	13	1		QLGCHFNNDSELVSCLRSK
198	521.2819	1040.5492	1040.5801	-0.0308	2	9	72	1		KMKIGPSHK + Oxidation (M)
47	540.2985	539.2912	539.3067	-0.0156	0	8	69	1		PQAPK
256	605.8032	1209.5918	1209.5924	-0.0007	0	8	80	1		MITASSPHNPR
506	426.2213	3401.7124	3401.6826	0.0298	2	8	31	1		LGWGHGDQEIFSTKEMIEAFDLPAIGRSAAR
224	555.2987	1108.5829	1108.5876	-0.0047	0	8	94	1		GNIHEDLLAK
507	487.2696	3403.8364	3403.8020	0.0344	2	7	17	1		LGEKDLQIQLAISLHAVDDELRSELIPMNK + Oxidation (M)
490	530.7835	3178.6576	3178.6339	0.0238	1	7	36	1		GFFGEHGGLFVSETLIPALQELADAYKAAK
491	530.7852	3178.6677	3178.6339	0.0338	1	6	42	1		GFFGEHGGLFVSETLIPALQELADAYKAAK
221	549.7449	1097.4753	1097.4634	0.0119	0	6	49	1		DMSIMQWR + 2 Oxidation (M)
332	763.8981	1525.7816	1525.7671	0.0145	1	6	1.2e+002	1		HRDAIQALEAAMGK + Oxidation (M)
584	651.8702	5206.9032	5206.9389	-0.0356	1	6	1.1	1		VTKYWHSLSNLVASLLNSIQSIASLLLLFLFIVIFGLLGMQVFGGR + Oxidation (M)
564	665.3522	4650.4141	4650.3745	0.0396	1	6	15	1		ALAETTTVDIVTGADVVEYYAGLATAIEGIQLPLRESSFFYTR
522	610.3269	3655.9178	3655.8820	0.0358	2	6	31	1		LNLASRLPSYFVRPDLGPKMYNAYGLITAEDR + Oxidation (M)
346	827.9468	1653.8790	1653.8872	-0.0082	1	6	1.2e+002	1		LLLHRNEIDSIMGK + Oxidation (M)
225	555.7525	1109.4904	1109.4957	-0.0054	1	5	81	1		SAQGMGKMER + Oxidation (M)
573	617.7923	4934.2805	4934.3253	-0.0448	2	5	13	1		GDAVLHPMGWDAFGLPAENAAIARNVDPGDWTDQNIAQMRAQLDR + 2 Oxidation (M)
421	779.3725	2335.0957	2335.0970	-0.0013	1	5	78	1		CTHSSSIEDLALGRCMEIIK + Oxidation (M)
486	622.3514	3106.7206	3106.7665	-0.0460	2	4	24	1		MRLSVIILAIFMISLFIMAAFLFFKR + Oxidation (M)
582	5102.5540	5101.5467	5101.5396	0.0072	2	4	23	1		GFTGLQGLPGPPGPSGDQGTSGPAGPSGPRGPPGPVGPSGKDGSNGIPGPIGPPGPR
481	610.3280	3046.6036	3046.6273	-0.0237	2	4	71	1		LIPEVINKEMENATKDIFPLQNVHVR
544	844.4700	4217.3136	4217.3368	-0.0232	1	4	9.4	1		AVNGFIRIWIMAALPIAISTAALLILLMFSCMLGPLLR + 3 Oxidation (M)
546	708.3530	4244.0742	4244.0387	0.0355	2	4	47	1		ALDDMFKATGHQNAYFPLFIPMSFLEKEAEHVEGFAK + Oxidation (M)
69	622.3678	621.3605	621.3156	0.0450	0	4	3.3e+002	1		GIMSSK
417	767.3897	2299.1472	2299.1042	0.0430	1	4	1.5e+002	1		KEAFEALESIMLNEEFGSAGK
231	563.7890	1125.5634	1125.5706	-0.0072	0	4	2e+002	1		ILTNFDFEK
433	430.2517	2575.4665	2575.4486	0.0180	2	4	26	1		VIEFPAPGIADRQAVKQPLQTGIK
83	675.3333	674.3260	674.3170	0.0090	0	4	4.9e+002	1		MNAPAR + Oxidation (M)
605	5918.5712	5917.5639	5917.5816	-0.0177	1	3	1.2	1		VPPPPETPAQEEMETETEASAPQGKEQDDTAAMLADFIDCPPDDEKPPPATEPDS
348	552.5938	1654.7595	1654.7957	-0.0363	1	3	1.1e+002	1		AQERMNQSHQSLAR
579	844.9728	5063.7928	5063.8234	-0.0306	2	3	3.5	1		LQLTKNLGKISGLIIGILILTTGIAAAVGIAASAGFDVSATGLQQGDAESAR
111	722.3264	721.3191	721.3395	-0.0204	0	3	2.2e+002	1		YLANQN
386	670.3190	2007.9351	2007.9497	-0.0147	1	3	1.2e+002	1		NLAESLDSRISQGDFNDK
510	571.2913	3421.7040	3421.6711	0.0328	0	3	1e+002	1		TNYFDAQMSSSTQNGSSLSQLVSIIIATFSPK
503	559.3135	3349.8371	3349.7882	0.0489	2	3	35	1		GTVDVVVTEGFTGNIALKTAEGTAKQIAQYIR
572	4916.7752	4915.7679	4915.7323	0.0356	1	3	1.8	1		MLKEVRPAVVSLLALTMITGLAYPLAVTGLATVLFPYQAQGSLVER + Oxidation (M)
588	663.3339	5298.6131	5298.6024	0.0107	2	2	32	1		SLGIQTAMLTGDNQDAAMSTQEQLENALDIVHSELLPQDKARIIDDFK + Oxidation (M)
515	579.3007	3469.7602	3469.7140	0.0463	2	2	1.1e+002	1		MMFPKVATEHVVPWAGLRPMMPNMMPRVGK + 2 Oxidation (M)
407	747.3720	2239.0943	2239.0977	-0.0034	2	2	2.1e+002	1		GGSEKMAIAIGKMIAEDPSFR + 2 Oxidation (M)
531	651.8657	3905.1506	3905.1812	-0.0306	2	2	20	1		RTSELIPLCHPLAIAGVTLELEPDAALPGIRIAATVK
146	846.4464	845.4391	845.4681	-0.0290	0	2	7e+002	1		LMAVVEGK
347	552.5930	1654.7572	1654.8049	-0.0478	1	2	1.5e+002	1		EKLEPNPEESQDIK
445	526.2826	2626.3765	2626.3901	-0.0136	1	2	1.9e+002	1		SHASNVGIATAMKAAGKPAAAGFLLEK + Oxidation (M)
597	5469.8427	5468.8354	5468.8218	0.0136	0	2	23	1		QQQIQQQQQHHQPPPLLQPQPQFWVQPQHPNHIPQPMAQLPILSK
406	738.0443	2211.1112	2211.0850	0.0262	2	1	3.1e+002	1		DDIIRMMVGREITQMFPK + 2 Oxidation (M)
534	813.3901	4061.9143	4061.9640	-0.0497	2	1	68	1		LELNSIGSQEDRADYRTALVAFLEQHIDVLDEDCK
603	5742.9858	5741.9785	5741.9827	-0.0042	2	1	24	1		LQPDLTDEEVLLLSMRDMNIAKLTSVDAPLFNAIVQDLFPNIELPVIDYGK + Oxidation (M)
575	622.3368	4970.6364	4970.6314	0.0049	1	1	29	1		TPEMITLEALTALVESGADAAQLLLPVAAGLSDFAQITLDATLAARFR + Oxidation (M)
397	700.0120	2097.0143	2097.0201	-0.0058	1	1	3.2e+002	1		VCTGYRFEGQELETLPAK
485	516.3045	3091.7832	3091.7548	0.0284	0	1	19	1		MNTIIGLILAYLLGSIPTGLWIGQIFFK
436	1301.6621	2601.3096	2601.2659	0.0437	1	1	3e+002	1		DAGFARANWQVMSGGIVALHSGWR + Oxidation (M)
520	725.3836	3621.8815	3621.9215	-0.0400	2	1	1.3e+002	1		IPEDITRILVPLIDLDISSFYTTVMIDTEKR + Oxidation (M)
205	527.2009	1052.3873	1052.4345	-0.0473	0	0	16	1		YNSACPEGR
376	609.9464	1826.8173	1826.8469	-0.0296	0	0	1.5e+002	1		QPIDPNQEITEICDR
563	663.9272	4640.4397	4640.4557	-0.0160	2	0	47	1		GAKATDIVVIVVAADDGVMPQTIEAIQHAQASEAPIIIAVNKMDK
601	5729.7763	5728.7690	5728.7957	-0.0267	2	0	37	1		WVPYFSAVETIINLTQDPDDLVSFKAMDVCANHKIEESVAYLSSIIDDVR + Oxidation (M)
470	480.7851	2878.6670	2878.6433	0.0238	2	0	20	1		LTFDPAEDIAAPFILKQARPKVAVLR
574	829.3614	4970.1249	4970.1476	-0.0227	2	0	3.8	1		MPSAQPFHHFAPRYSEMGCAGMQMRDAHEENPESILDEHVQR + 3 Oxidation (M)
1	402.2353	401.2281								
2	402.3577	401.3504								
3	412.1691	411.1618								
4	412.1694	411.1621								
5	412.7557	411.7484								
6	415.2128	414.2055								
7	415.2130	414.2057								
8	415.2145	414.2072								
9	415.2549	414.2476								
10	415.3427	414.3354								
11	415.3429	414.3357								
12	418.3902	417.3829								
13	419.1682	418.1610								
14	426.3195	425.3122								
15	430.2448	429.2375								
16	432.2389	431.2316								
17	432.2391	431.2318								
18	432.2392	431.2319								
19	432.2818	431.2745								
20	432.2822	431.2749								
21	432.2830	431.2757								
22	433.1285	432.1212								
23	433.1806	432.1733								
24	444.1141	443.1068								
25	444.1173	443.1100								
26	444.1176	443.1103								
27	444.1177	443.1104								
28	444.1179	443.1106								

29	444.1179	443.1107
30	447.1393	446.1320
31	456.2819	455.2746
32	476.3081	475.3009
33	484.3866	483.3793
34	500.3076	499.3003
35	500.3077	499.3005
36	500.3087	499.3014
37	500.3831	499.3759
38	502.3740	501.3667
39	503.3079	502.3006
40	504.3037	503.2964
41	520.3344	519.3272
42	520.3349	519.3276
43	525.3227	524.3155
44	528.3644	527.3571
45	528.3657	527.3584
46	539.3334	538.3261
48	544.3350	543.3278
49	546.3989	545.3917
50	546.4023	545.3951
51	548.3309	547.3236
52	578.3403	577.3331
53	579.2160	578.2088
54	579.5355	578.5282
55	579.5364	578.5291
56	579.5364	578.5291
57	579.5366	578.5294
58	586.4196	585.4124
59	590.4290	589.4217
60	606.3338	605.3265
61	607.5679	606.5606
62	608.3872	607.3799
63	614.5729	613.5656
64	615.4051	614.3978
65	615.4053	614.3980
66	615.4057	614.3984
67	617.4079	616.4007
68	617.4083	616.4010
70	625.9693	624.9620
71	630.4461	629.4389
72	630.4462	629.4389
73	638.1649	637.1576
74	638.3981	637.3908
75	638.4108	637.4036
76	650.2069	649.1996
78	661.5632	660.5559
79	661.5632	660.5559
80	661.5636	660.5564
82	675.3281	674.3208
84	675.6618	674.6545
85	675.6774	674.6701
86	675.6774	674.6701
87	675.6780	674.6707
88	675.6780	674.6707
89	675.6780	674.6708
90	675.6785	674.6713
91	675.6786	674.6714
92	675.6800	674.6727
93	675.6855	674.6782
94	677.6848	676.6776
95	677.6854	676.6781
96	678.4820	677.4747
97	678.5842	677.5769
98	678.5898	677.5825
99	678.5920	677.5847
100	683.5453	682.5380
101	683.5455	682.5383
102	683.5456	682.5383
103	696.4402	695.4329
104	714.3241	713.3168
105	717.5425	716.5352
106	717.6301	716.6229
107	717.6315	716.6243
108	718.4981	717.4908
109	718.4982	717.4909
110	720.4349	719.4276
112	732.4788	731.4716
113	734.6488	733.6415
114	734.6503	733.6430
115	734.6509	733.6437
116	739.6049	738.5976
117	739.6050	738.5977
118	739.6052	738.5980
119	739.6057	738.5984
120	739.6075	738.6002
121	739.6079	738.6006
122	740.4663	739.4590
123	752.5488	751.5415
124	752.5491	751.5418
125	752.5495	751.5422
126	762.5237	761.5165

131	806.5503	805.5430
139	835.4711	834.4638
141	842.5130	841.5057
163	884.0956	883.0884
165	918.4879	917.4806
170	934.8809	933.8736
171	934.8812	933.8740
179	984.4923	983.4850
184	1000.6084	999.6011
	501.7842	1001.5538
	516.7017	1031.3888
	516.7052	1031.3958
206	1053.3952	1052.3879
	534.2648	1066.5150
	549.7447	1097.4748
	608.2585	1214.5025
	608.2593	1214.5041
	613.3611	1224.7077
	613.3645	1224.7144
	645.8814	1289.7483
	439.1024	1314.2852
	446.2574	1335.7504
	678.3268	1354.6390
	698.7826	1395.5506
	717.8918	1433.7690
	481.5385	1441.5936
	481.5395	1441.5968
	528.3672	1582.0796
	560.9509	1679.8310
	848.9179	1695.8211
	870.9270	1739.8393
	580.9537	1739.8394
	441.3256	1761.2734
	590.5956	1768.7651
	456.2824	1821.1005
	476.3070	1901.1988
	655.2908	1962.8506
	655.2908	1962.8506
	677.8645	2030.5716
	415.2516	2071.2217
	696.4387	2086.2943
	550.1402	2196.5317
	734.6458	2200.9155
402	1106.0612	2210.1077
	755.3479	2263.0218
413	1142.0992	2282.1838
420	1166.0925	2330.1705
	794.4313	2380.2722
	804.9904	2411.9495
	638.3865	2549.5171
	854.4041	2560.1906
437	1301.6654	2601.3162
439	1301.6655	2601.3164
	884.0997	2649.2774
	885.7513	2654.2320
	889.7488	2666.2247
	446.2567	2671.4964
	536.3142	2676.5347
	545.7701	2723.8141
	697.4274	2785.6806
	562.7947	2808.9372
	564.3471	2816.6992
	415.1258	2898.8297
	594.1511	2965.7191
479	1000.7914	2999.3523
	608.3897	3036.9121
483	1531.1802	3060.3458
	516.3028	3091.7729
487	1038.7567	3113.2484
	526.2766	3151.6156
	453.7807	3169.4141
	456.2874	3186.9607
	534.2419	3199.4080
	648.8508	3239.2178
	653.4463	3262.1953
	660.8841	3299.3840
	1103.9192	3308.7359
	416.2436	3321.8906
	561.2997	3361.7547
	679.8049	3393.9879
	432.2757	3450.1477
	867.3781	3465.4831
	867.3790	3465.4868
	579.5301	3471.1371
	497.0205	3472.0926
	696.4423	3477.1751
	711.8573	3554.2501
	608.3877	3644.2828
	460.2670	3674.0779
	528.2980	3691.0350
	3737.1174	3736.1101
	539.3335	3768.2838

	633.3220	3793.8880
	763.8716	3814.3216
	763.8766	3814.3467
	3881.4919	3880.4846
	562.2972	3929.0295
	507.3018	4050.3562
	509.2621	4066.0384
	679.3633	4070.1364
	4097.3706	4096.3633
	4111.5889	4110.5816
	4137.4019	4136.3946
	695.8441	4169.0207
	4180.6198	4179.6125
	527.2003	4209.5443
	527.2032	4209.5670
	848.8375	4239.1513
	608.3853	4251.6462
	4292.4647	4291.4574
	717.6251	4299.7069
	717.7699	4300.5758
	736.4370	4412.5783
	632.3741	4419.5679
	632.8513	4422.9080
	634.4570	4434.1479
	740.4582	4436.7055
	4452.4125	4451.4052
	566.3002	4522.3430
	756.4234	4532.4964
	756.4300	4532.5363
	651.2013	4551.3581
	652.3893	4559.6742
	663.4533	4637.1218
	590.1545	4713.1780
	674.4811	4714.3165
	4736.2038	4735.1965
	800.4212	4796.4835
	691.3387	4832.3201
	692.3599	4839.4681
	608.3814	4858.9930
	832.4924	4988.9110
	5039.4929	5038.4856
	632.3387	5050.6511
580	5070.1876	5069.1803
	638.3498	5098.7399
583	5117.9694	5116.9621
585	5222.7974	5221.7901
586	5261.9716	5260.9643
	883.9111	5297.4228
	885.3718	5306.1873
	5318.0546	5317.0473
	887.9785	5321.8273
	890.4115	5336.4251
	5345.0843	5344.0770
	764.4656	5344.2085
	899.0048	5387.9852
	5462.8137	5461.8064
	802.4250	5609.9239
	5647.6501	5646.6428
	5677.6682	5676.6609
	955.9700	5729.7763
	844.4875	5904.3615
606	6062.2605	6061.2532
607	6096.6204	6095.6131
	883.8928	6180.1987
	6313.4377	6312.4304
	6460.6320	6459.6247
	6513.6818	6512.6745
	6592.1910	6591.1837
	6618.1763	6617.1690
	6624.4984	6623.4911
	6643.5077	6642.5004
	6653.7948	6652.7875
	6734.6994	6733.6921
	6968.0027	6966.9954
	7017.3864	7016.3791
	885.3742	7074.9356
	7247.7937	7246.7864
	7270.6964	7269.6891
	7291.8846	7290.8773
	7351.9738	7350.9665
	920.0040	7351.9738
	7378.9385	7377.9312
	7905.3602	7904.3529
	7955.7108	7954.7035
	7988.6195	7987.6122
	8082.6824	8081.6751
	8279.1311	8278.1238
	8839.4573	8838.4500

Search Parameters

```
Type of search          : MS/MS Ion Search
Enzyme                  : Trypsin
Fixed modifications     : Carbamidomethyl (C)
Variable modifications  : Oxidation (M)
Mass values             : Monoisotopic
Protein Mass            : Unrestricted
Peptide Mass Tolerance  : ± 0.05 Da
Fragment Mass Tolerance : ± 0.05 Da
Max Missed Cleavages    : 2
Instrument type         : Default
Number of queries       : 632
```

Mascot: http://www.matrixscience.com/
